# Asymmetric reproductive interference: The consequences of cross‐pollination on reproductive success in sexual–apomictic populations of *Potentilla puberula* (Rosaceae)

**DOI:** 10.1002/ece3.3684

**Published:** 2017-11-28

**Authors:** Christoph Dobeš, Susanne Scheffknecht, Yulia Fenko, Dagmar Prohaska, Christina Sykora, Karl Hülber

**Affiliations:** ^1^ Department of Forest Genetics Austrian Research Centre for Forests Vienna Austria; ^2^ Department of Pharmacognosy and Pharmacobotany University of Vienna Vienna Austria; ^3^ Division of Conservation Biology, Vegetation Ecology and Landscape Ecology Department of Botany and Biodiversity Research University of Vienna Vienna Austria; ^4^ Vienna Institute for Nature Conservation and Analyses Vienna Austria

**Keywords:** apomixis, crossing barrier, genomic endosperm balance, pollen, polyploidy, selfing

## Abstract

Apomixis evolves from a sexual background and usually is linked to polyploidization. Pseudogamous gametophytic apomicts, which require a fertilization to initiate seed development, of various ploidy levels frequently co‐occur with their lower‐ploid sexual ancestors, but the stability of such mixed populations is affected by reproductive interferences mediated by cross‐pollination. Thereby, reproductive success of crosses depends on the difference in ploidy levels of mating partners, that is, on tolerance of deviation from the balanced ratio of maternal versus paternal genomes. Quality of pollen can further affect reproductive success in intercytotype pollinations. Cross‐fertilization, however, can be avoided by selfing which may be induced upon pollination with mixtures of self‐ and cross‐pollen (i.e., mentor effects). We tested for reproductive compatibility of naturally co‐occurring tetraploid sexuals and penta‐ to octoploid apomicts in the rosaceous species *Potentilla puberula* by means of controlled crosses. We estimated the role of selfing as a crossing barrier and effects of self‐ and cross‐pollen quality as well as maternal: paternal genomic ratios in the endosperm on reproductive success. Cross‐fertilization of sexuals by apomicts was not blocked by selfing, and seed set was reduced in hetero‐ compared to homoploid crosses. Thereby, seed set was negatively related to deviations from balanced parental genomic ratios in the endosperm. In contrast, seed set in the apomictic cytotypes was not reduced in hetero‐ compared to homoploid crosses. Thus, apomictic cytotypes either avoided intercytotype cross‐fertilization through selfing, tolerated intercytotype cross‐fertilizations without negative effects on reproductive success, or even benefitted from higher pollen quality in intercytotype pollinations. Our experiment provides evidence for asymmetric reproductive interference, in favor of the apomicts, with significantly reduced seed set of sexuals in cytologically mixed populations, whereas seed set in apomicts was not affected. Incompleteness of crossing barriers further indicated at least partial losses of a parental genomic endosperm balance requirement.

## 1 INTRODUCTION

Variation in chromosome number is an important cytogenetic phenomenon in plant speciation and diversification. Three major types can be distinguished: dysploidy (variability of base chromosome number), aneuploidy (deviation from a multiple of the base chromosome number), and polyploidy (addition of whole chromosome sets). In particular, the latter one is regarded as a major source of intraspecific variation in numerous plant species (e.g., Dobeš & Vitek, 2000; Duchoslav, Šafářová, & Krahulec, 2010; Ehrendorfer, 1980; Stebbins & Dawe, 1987; Trávníček et al., 2011). Intraspecific ploidy variation is frequently geographically structured ranging from allopatry (Lihová & Marhold, 2003; Mráz, Chrtek, & Šíngliarová, 2009) via parapatry of cytotypes (e.g., Keeler, 1992; Lauterbrunner, 1979) to the widespread occurrence of cytologically mixed and fully sympatric populations (Kao, 2008; Keeler, 2004; Marhold et al., 2010).

Polyploidization is commonly accompanied by changes in the reproductive system like the breakdown of self‐incompatibility (e.g., Barrett, 1988) or sometimes by the evolution of apomixis (i.e., asexual reproduction via seeds: Asker & Jerling, 1992; Carman, 1997). A major variant of apomixis is gametophytic apomixis, in which the female gametophyte or embryo sac is still functional, and which is common in the Asteraceae, Poaceae, Ranunculaceae, and Rosaceae (Asker & Jerling, 1992; Carman, 1997). In most cases, the ancestral sexuals are diploid thus giving rise to sexual diploid–apomictic polyploid contrasts (e.g., Bayer, 1997; Cosendai, Rodewald, & Hörandl, 2011; Hojsgaard, Schegg, Valis, Martinez, & Quarin, 2008), although reproductive differentiation at the polyploid level also exists (Dobeš, Milosevic, et al., 2013; Rotreklová, Krahulcová, Vanková, Peckert, & Mráz, 2002; Savidan, Carman, & Dresselhaus, 2001).

The interaction of ploidy and mode of reproduction is of high relevance for the ecological and spatial distribution of cytotypes. Three principal factors drive the distribution of both sexual and apomictic cytotypes from the geographic to the population scale: migration (Cosendai, Wagner, Ladinig, Rosche, & Hörandl, 2013; Dobeš, Mitchell‐Olds, & Koch, 2004; Parisod, Holderegger, & Brochmann, 2010), habitat preferences (e.g., Bayer, Purdy, & Lededyk, 1991; Meirmans, Calame, Bretagnolle, Felber, & Nijs, 1999; Sonnleitner et al., 2010), and reproductive interference among cytotypes (e.g., Baack, 2004; Stewart‐Cox, Britton, & Mogie, 2005; Van Dijk, Hartog, & Can Wilke, 1992).

Reproductive interference has been defined as any negative effect of interspecific sexual interaction on fitness (see Kyogoku, 2015 for a review). In plants, reproductive interference is mediated via cross‐pollination and is known to affect the distribution of cytotypes (e.g., Hardy, Loose, Vekemans, & Meerts, 2001; Kay, 1969; Van Dijk et al., 1992), promoted via the minority cytotype exclusion principle (Levin, 1975). Negative effects on the frequency of both sexual and apomictic cytotypes can be expected if intercytotype pollen transfers in sexual–apomictic systems yield less fit offspring compared to intracytotype pollinations. Susceptibility to exclusion, however, differs for sexual and apomictic cytotypes for reasons pertaining to the cytology of seed formation: In pure sexual species, reciprocal cross‐fertilization of ploidy‐differentiated cytotypes is possible and such intercytotype cross‐fertilizations change the ploidy of the progeny (i.e., the embryo) as well as the ploidy of the endosperm. The cytologically transformed progeny is lost to the population of the sexuals. Such intimate and direct loss of progeny is not possible under apomictic reproduction due to autonomous development of the embryo. Cross‐fertilization affects the endosperm only and only in apomicts which require a fertilization event to initiate endosperm formation and seed development (i.e., pseudogamous gametophytic apomicts; in contrast to autonomous gametophytic apomicts in which endosperm develops without a precedent fertilization: Hörandl, 1992; Rutishauser, 1969. For convenience, we refer henceforward to pseudogamous gametophytic apomixis as apomixis). Consequently, negative effects on seed set resulting from cross‐fertilization are restricted in apomicts to developmental disturbances of the endosperm and these effects may rather be gradual than absolute. Moreover, the cytology of endosperm development in apomicts differs from that in sexuals with potential consequences for the sensitivity of the endosperm to ploidy changes caused by cross‐fertilization as detailed in the following paragraph.

Reduction in female fertility was observed in experimental interploidy crosses of sexuals and found to be related to the imbalance in the number of parental genomes in the endosperm (e.g., Lin, 1984; Scott, Spielman, Bailey, & Dickinson, 1988). The endosperm of sexual angiosperms is typically triploid carrying two maternal (*m*) genomes and one paternal (*p*) genome (Rutishauser, 1969). Violation of this 2*m*:1*p* genomic ratio is often entailing abnormal endosperm development and, thus, decreased seed vitality or seed abortion (e.g., citations in Haig & Westoby, 1991; Lin, 1984; Nishiyama & Inomata, 1966). The sensitivity of the endosperm to parental genomic ratios deviating from the 2*m*:1*p* ratio has been related to genomic imprinting (Haig & Westoby, 1991; Kinoshita, 2007), an epigenetic mechanism that results in parent‐of‐origin dependent gene expression. Parental genomic endosperm balance is also of particular interest in apomicts. The involvement of two unreduced polar nuclei in the endosperm (Rutishauser, 1969) doubles the maternal genomic contribution leading to a deviation from the 2*m*:1*p* genomic ratio. Normal endosperm development (Koltunow & Grossniklaus, 2003) is secured by reestablishing the normal parental genomic ratio through doubling the paternal genomic contribution to the endosperm resulting in a 4*m*:2*p* ratio (Dobeš, Koch, & Sharbel, 2006; Rutishauser, 1954), involvement of only one polar nucleus in endosperm formation (Savidan, 1975; Warmke, 1954), or, hypothetically, in halving the number of imprintable maternal genes expressed in the polar nuclei enabling endosperm with 4*m*:1*p* ratios to develop (Talent, 2009). Theoretically, regarding numbers of involved parental genomes, apomicts which adapted endosperm formation via these strategies can also tolerate cross‐fertilization by their sexual counterparts as long as they are homoploid (which, however, is the exception). Alternatively, apomicts may tolerate deviating parental genomic ratios in the endosperm (e.g., Grimanelli, Hernández, Perotti, & Savidan, 1997; Quarin, 1999; Šarhanová, Vašut, Dancák, Bureš, & Trávníček, 2012), a strategy which should provide them with an advantage in case of cross‐fertilization by cytotypes of differing ploidy including sexuals.

An additional factor potentially influencing reproductive success upon intercytotype pollinations in sexual–apomictic complexes is the quality of cross‐pollen. Although apomictic individuals maintain functional pollen, it is often less viable than pollen from sexual counterparts (Dobeš, Milosevic, et al., 2013; Hörandl, Dobeš, & Lambrou, 1997). As a consequence, heteroploid crosses can result in reduced seed set and offspring vitality in sexuals (Britton & Mogie, 2001).

Selfing is an effective barrier against cross‐fertilization in sexual plants as well as apomicts—we use the term “selfing” to refer to both double fertilization in sexuals and fertilization of the polar nuclei/the central cell only in apomicts. Most apomicts are self‐compatible (Hörandl, 2010), which allows them to avoid or reduce intercytotype fertilizations and hence genomic imbalances. Furthermore, selfing potentially eliminates the minority cytotype problem and provides reproductive assurance independent from pollinators and mating partners. In contrast, the sexual ancestors of apomicts are usually self‐incompatible outcrossers (Asker & Jerling, 1992). Nevertheless, sexuals may escape negative effects of cross‐fertilization by apomicts in mixed populations by so‐called mentor effects (i.e., induced selfing). Mentor effects refer to the blocking of cross‐fertilization and promotion of selfing by mixtures of self‐ and cross‐pollen deposited on the stigma of otherwise self‐incompatible individuals. The importance of mentor effects was demonstrated in crosses among sexual pollen recipients and apomictic pollen donors (Hörandl & Temsch, 2009; Mráz, 2003).

Patterns of cytotype distribution may be explained by reproductive interference with the reproductive incompatibility of cytotypes driving spatial avoidance and compatibility allowing a mixture of cytotypes. We addressed this hypothesis by cross‐pollinating naturally co‐occurring sexual and apomictic individuals comprising five ploidy levels in the model system *Potentilla puberula* (Rosaceae) in a common garden experiment. We quantified reproductive success (seed set and germination rate) and inferred paternal genomic contributions to and parental genomic ratios in the endosperm from a flow cytometric seed screen (FCSS) to address the following questions: (1) Do differences in the ploidy of crossing partners, the reproductive mode of pollen receptors, and/or pollen quality of donors affect the reproductive success? (2) Do sexuals and/or apomicts avoid cross‐fertilization in intercytotype pollinations through (induced) selfing? (3) Specifically, does reproductive success decrease in case of intercytotype cross‐fertilizations with increasing deviation from the 2*m*:1*p* genomic ratio and the 4*m*: 2*p* or 4*m*:1*p* genomic ratios in the endosperm of sexually and apomictically derived seeds, respectively? As methodological prerequisite, we established reproductive mode (sexual vs. apomictic) and self‐compatibility of individuals.

## 2 MATERIAL AND METHODS

### 2.1 The study system

**Figure 1:**
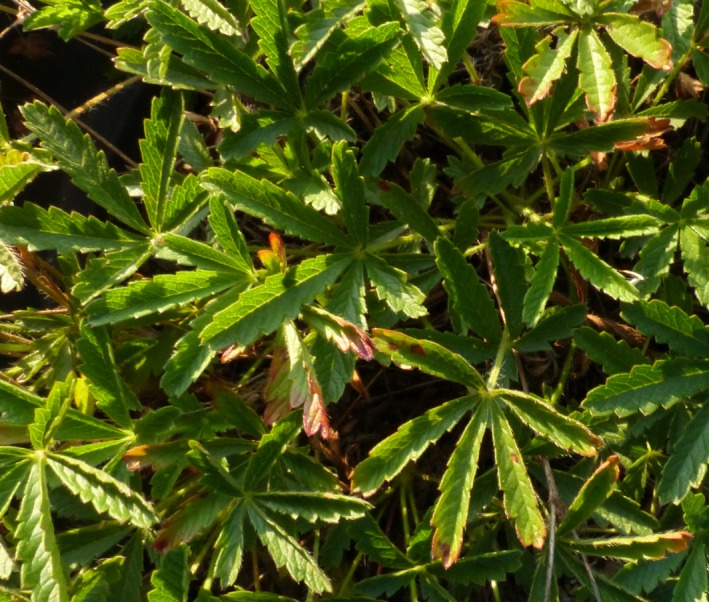
The study system *Potentilla puberula*, a hemicryptophte of typically xeric mountainous habitats

*Potentilla puberula* Krašan (= *Potentilla pusilla* Host: Soják, 2010; Figure 1) constitutes a suitable model to study the consequences of reproductive interference for co‐existence of reproductively differentiated cytotypes. The species comprises tetraploids being almost exclusively sexual and self‐incompatible and penta‐ to octoploids which are preferentially apomictic (Dobeš, Milosevic, et al., 2013; Prohaska, 2013). A screen of 269 populations along a latitudinal transect through the Eastern European Alps revealed about every second population to be cytologically mixed (i.e., inhabited by 2–5 cytotypes in various combinations). Nevertheless, the presence of tetraploids in a population was negatively related to the presence of penta‐ to octoploids and vice versa. In contrast, the occurrences of penta‐ to octoploids were hardly related to each other (Hülber, Scheffknecht, & Dobeš, 2013).

*Potentilla puberula* is assumed to be of allopolyploid origin with tetraploids (Soják, 2010) constituting the lowest ploidy level (Dobeš, 1999; Dobeš, Milosevic, et al., 2013). Functionality of the gametophytic SI system suggested that the tetraploids are functional diploids (Dobeš, Milosevic, et al., 2013). The cytotypes, however, are genetically barely differentiated (Paule, Scherbantin, & Dobeš, 2012) suggesting an intraspecific origin. Two polar nuclei contribute to the endosperm of both sexually and apomictically derived seed and either one or two of the sperm in the latter (Dobeš, Milosevic, et al., 2013). Pollen is mostly meiotically reduced in *P. puberula* irrespective of reproductive mode and ploidy of individuals (Christoph Dobeš and Christina Sykora unpublished research). Hence, the following parental genomic ratios in the endosperm constitute the normal condition in this system: 2*m:*1*p* in the tetraploid sexual cytotype with *m* and *p* representing two chromosome sets (half the number of chromosome sets present in the maternal genome), and 4*m:*1*p* or 4*m:*2*p* in the apomictic cytotypes with *m* and *p* representing half the number of their five, six, seven, and eight chromosome sets, respectively.

### 2.2 Plant material

The study is based on 133 individuals of *P. puberula* representing 11 populations from East Tyrol, Austria. Populations were selected from a pool of 50 populations previously screened for ploidy variation using flow cytometry and DAPI‐stained leaf samples within this area (Scheffknecht, Hülber, Moser, & Dobeš, 2012) under the criteria to contain each class of cytotype (tetraploid sexuals, and penta‐ and heptaploids apomicts) with a frequency of >10% in a population and that all cytotypes are included in the study. Applying these criteria, hexa‐ and octoploids were too rare in populations with sexuals to be included (but criteria were met by these two cytotypes in pure apomictic populations). Per population, 7–22 individuals covering 2–5 ploidy levels were selected (Table 1). Individuals were genotyped beyond this study using eight microsatellite loci developed for the *Potentilla* core group (markers PMS001079, PMS001080, PMS001082, PMS001193, PMS001292, PMS001476, PMS001862, PMS002118 (Dobeš & Scheffknecht, 2012). Plants were cultivated in the experimental garden of the Department of Pharmacognosy, University of Vienna, and grown in pots (14 cm in diameter) using a substrate composed of six parts ground soil, two parts of bark humus (Ríndenhumus, Kranzinger, Straßwalchen, Austria), and two parts of quartz sand.

**Table 1:** General description of the 11 studied populations of *Potentilla puberula* from East Tyrol, Austria. Coordinates are provided in WGS84 standard. 4*x*, 5*x*, 6*x*, 7*x,* and 8*x* refer to tetra‐, penta‐, hexa‐, hepta‐, and octoploidy, respectively. Reproductive mode indicates the dominant reproductive mode of seed formation of cytotypes observed in the respective population

Population	Longitude; latitude	Ploidy	Reproductive mode	*N* individuals	*N* genotypes
Raut	12.57448E; 46.78112N	4*x*	Sexual	5	5
		5*x*	Apomictic	5	1
Zabernig	12.51920E; 47.00467N	4*x*	Sexual	5	5
		5*x*	Apomictic	5	4
		7*x*	Apomictic	3	3
Groder	12.33275E; 47.01883N	4*x*	Sexual	5	5
		5*x*	Apomictic	5	4
Erlbach	12.36964E; 46.74653N	5*x*	Apomictic	5	3
		7*x*	Apomictic	5	3
		8*x*	Apomictic	5	2
Lana	12.63190E; 46.98575N	5*x*	Apomictic	5	4
		6*x*	Apomictic	5	5
Stein	12.52672E; 47.02757N	5*x*	Apomictic	10	5
		7*x*	Apomictic	3	3
		8*x*	Apomictic	5	1
Innervillgraten	12.36085E; 46.81183N	4*x*	Apomictic	5	2
		5*x*	Apomictic	5	3
		6*x*	Apomictic	5	3
		7*x*	Apomictic	3	2
		8*x*	Apomictic	4	2
Virgen	12.45868E; 47.00545N	5*x*	Apomictic	4	1
		7*x*	Apomictic	5	1
Arnig	12.63231E; 46.98451N	5*x*	Apomictic	5	1
		6*x*	Apomictic	2	1
Schrottendorf	12.67375E; 46.79172N	5*x*	Apomictic	5	1
		6*x*	Apomictic	3	1
		8*x*	Apomictic	1	1
Oberburgfrieden	12.71367E; 46.79808N	5*x*	Apomictic	5	1
		6*x*	Apomictic	5	3

### 2.3 Pollen quality

Flowers were collected in late balloon stage and immediately fixed in Carnoy's solution (six parts ethanol: three chloroform: one acetic acid). A single anther per individual was used to estimate pollen quality using a Nikon Optiphot light microscope (Nikon, Tokyo, Japan) and bright‐field illumination. Quality of pollen was established in determining the percentage of morphologically normally developed pollen grains (i.e., of regular circular or roundish form; compared to deformed pollen grains of irregular or ellipsoid form) among 104–192 inspected. This parameter was shown to closely correlate (Pearson *r* ^*2*^ of .99, *p* < .001, *N *=* *373) with the vitality of pollen, that is, stainability using Peterson's vitality stain (Peterson, Slovin, & Chen, 2010) in *P. puberula* (cf. Dobeš, Milosevic, et al., 2013). Analyses were performed at 100‐fold magnification. We tested for differences in pollen quality among ploidy levels by means of logistic regressions using the proportion of viable pollen grains as response and ploidy level as a categorical predictor. Number of individuals was used as weighting factor, because proportions of normal pollen were pooled over individuals for each cytotype within populations. In regression analyses using treatment contrasts, categorical predictors like ploidy allow for pairwise comparisons only with a predefined baseline level. Thus, it was necessary to re‐fit the model using different cytotypes as baseline levels; that is, each cytotype was compared to the remaining ones in a separate model. An inflation of Type I errors due to multiple comparisons was avoided by applying a Bonferroni correction of *p* values. Analyses were performed using R (R Development Core Team 2011).

### 2.4 Crossing experiments

A controlled ex situ crossing experiment was carried out in spring 2012 in the experimental garden of the Department of Pharmacognosy. Flowers were bagged a few days before anthesis using bridal veil as this material has the least effect on the microclimate of the bagged flowers (Wyatt, Boyles, & Derda, 1992). At stigma, maturity flowers were pollinated by gently rotating a mature flower of the pollen donor over all recipient's stigmas and anthers, thereby simultaneously depositing mixtures of donor‐ and self‐pollen onto all stigmas of the multipistillate flowers. Three treatments were applied to each individual: (1) selfing (pollination of flowers with pollen from the same individual), (2) homoploid crosses (reciprocal pollination of individuals of the same cytotype), (3) heteroploid crosses (reciprocal pollination of individuals of different cytotype). Each treatment was applied to one flower per individual. Selfings were performed to estimate the degree of self‐compatibility. Homo‐ and heteroploid crosses were performed to estimate the compatibility of cytotypes, that is, to test for effects of difference in ploidy of crossing partners on reproductive success. Crosses were performed among all possible pairwise combinations of individuals within a population (see Appendix S1 for details); that is, in the homoploid crosses, each individual was crossed with all other individuals of the same cytotype and different genotype present in a particular population as well as with all individuals of different cytotype present in this population in the heteroploid crosses. Tetraploids from population Innervillgraten which turned out to be apomictic were excluded from further analyses.

Seed set was estimated by counting the ovules (actually, we counted the ovaries each containing one ovule) per flower (two flowers per individual) using a stereoscopic microscope (Nikon SMZ‐U, Nikon, Japan). At maturity, the number of developed seeds (actually fruitlets each usually containing a single seed; however, for convenience, we consistently use the term seed both when referring to fruitlets and isolated seeds used in the FCSS) was assessed and seed: ovule ratios calculated for each flower. Depending on the number of obtained seeds and the number of seeds destructively analyzed in the FCSS, 1–20 seeds per flower were sown in sterilized Neuhaus N3 substrate (Klasmann‐Deilmann, Geeste, Germany) from the 28th to 30th of May 2013 in a temperate greenhouse of the Department of Pharmacognosy. Germination was recorded on a weekly basis. Germination rate was calculated as the percentage of sown seeds developing cotyledons or into later stages within 12 weeks.

Seed set of homoploid crosses was compared to those in heteroploid crosses (test for crossability of cytotypes) and those of selfings (test for self‐compatibility/self‐incompatibility) using generalized linear mixed models (GLMMs) for both sexual (tetraploid) individuals and individuals reproducing via apomixis (penta‐ to octoploid; only two apomictically reproducing tetraploids were available and hence excluded from the test). For the homo‐ and heteroploid crosses, we related seed set to the ploidy of the pollen donor, the pollen quality of donor and recipient (i.e., self‐pollen), and the interaction between ploidy and donor‐pollen quality. We assumed the proportion of seeds per flower to be a binomially distributed random variable and, thus, applied a logit‐link function. To consider potential autocorrelation of values derived from individuals originating from the same population and flowers of the same individual, we included random effect intercepts for populations and individuals nested in population. Analyses were performed separately for each ploidy level using the function glmer of the library lme4 (Bates, Mächler, & Bolker, 2011) in R (R Development Core Team 2011).

### 2.5 Establishment of reproductive mode and calculation of parental genomic contributions to and parental genomic ratios in the endosperm

The reproductive origin and parental genomic contributions to the endosperm of a subset of 1,900 seeds obtained in the crosses were inferred using FCSS, performed separately for each seed. One to 11 seeds, depending on the number of available seeds, were randomly drawn per flower. Additionally, 93 seeds obtained from 11 tetraploid individuals used in the crossing experiment were screened for the purpose to establish their reproductive mode. The FCSS protocol followed Dobeš, Lückl, Hülber, and Paule (2013). *Pisum sativum* cv. Kleine Rheinländerin (Greilhuber & Ebert, 1994) and a strain of *Lathyrus tuberosus* (Fabaceae) co‐chopped with the sample served as internal standards. DAPI (4′,6‐diamidino‐2‐phenylindole) was used as DNA‐selective stain. The embryo: standard fluorescence ratio and the peak index (i.e., the endosperm: embryo fluorescence ratio) were calculated from the means of the corresponding fluorescence signals.

We distinguish between sexuality (i.e., involving female meiosis and the zygotic origin of the embryo) and apomixis (i.e., parthenogenesis in combination with female apomeiosis) according to Dobeš, Lückl, et al. (2013). Individuals analyzed for at least 10 seeds and forming ≥90 % of these seeds either via sexuality or apomixis were classified as sexuals and apomicts, respectively.

The maternal and paternal genomic contributions to the endosperm were calculated from the embryo and endosperm ploidies according to Dobeš, Lückl, et al. (2013) as follows: The maternal genomic contribution to the endosperm is *2 × (ploidy of the endosperm − ploidy of the embryo)* for sexually derived seeds and *2 × ploidy of the embryo* for seeds with parthenogenetically (the term refers to development of the egg cell without fertilization as in apomictically derived embryos) derived embryos. The paternal genomic contribution was computed as 2 × *ploidy of the embryo − ploidy of the endosperm* for seeds with sexually derived embryos and as *ploidy of the endosperm − 2 × ploidy of the embryo* for seeds with parthenogenetically derived embryos. The ploidy of apomictically derived embryos thereby was equaled with that of the maternal plant (based on the assumption that apomictic progeny recovers the maternal genome). The ploidy of sexually derived embryos was determined in using as a reference for the tetraploids averaged embryo: standard ratios of embryos derived via regular sexuality (homoploid crosses only) and for the higher ploid cytotypes averaged values of apomictically derived embryos for each cytotype. The ploidy of the endosperm was inferred from the mean fluorescence intensity of the endosperm signal relative to that of the embryo (i.e., the peak index). Estimates of paternal and maternal genomic contributions were used to calculate *m*:*p* genomic ratios in the endosperm. *m* and *p* are expressed either in units of *n* or *x*. We used, according to Greilhuber (2005), *n* (the haplophasic chromosome number) to indicate the number of holoploid genomes (i.e., the whole chromosome complement with chromosome number *n*), and *x* (the chromosome number of the monoploid genome) when referring to the number of chromosome sets (i.e., the generative ploidy).

### 2.6 Selfing versus intercytotype cross‐fertilization and relating parental genomic endosperm balance to reproductive success

**Figure 2:**
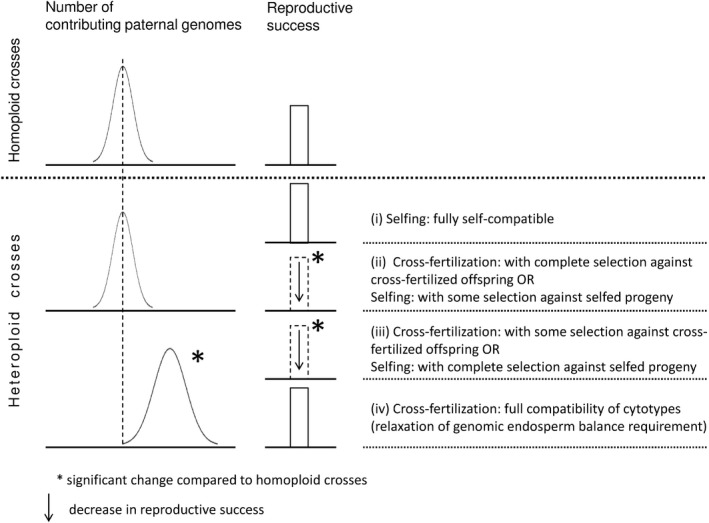
Assessment of the mode of mating (cross‐fertilization versus induced selfing) in heteroploid crosses of apomictic high polyploids upon self‐incompatible, tetraploid sexuals without emasculation based on the presence/absence of changes in the paternal genomic contribution to the embryos (and the endosperm) and changes in reproductive success compared to (tetraploid) homoploid crosses. Intercytotype cross‐fertilization will lead to a change in the paternal genomic contribution, whereas no change is indicative of progeny derived from selfing. (i) No differences neither in the paternal genomic contribution nor the reproductive success indicate full self‐compatibility (i.e., selfing of tetraploids), whereas (iv) an increase in the number of parental genomes without reduction in the reproductive success suggests full compatibility of cytotypes (i.e., relaxation of genomic endosperm balance requirement). In contrast, a reduction in reproductive success indicates selection against selfed and/or cross‐fertilized progeny (leading to abortion of seeds), that is, entails an ambiguous inference: (ii) reduced reproductive success, but no change in parental genomes indicates either complete selection against cross‐fertilized progeny (i.e., only selfed progeny developed into seeds) or selfing occurred and some selfed progeny was lost due to inbreeding depression. In contrast, (iii) change in the paternal genomic contribution accompanied by a reduction in reproductive success either indicates some selection against cross‐fertilized progeny or complete selection against selfed progeny

We inferred the origin of seeds obtained in heteroploid crosses by comparing the numbers of paternal genomes contributing to the endosperm (and embryo) and the reproductive success to those in homoploid crosses. By combining these two parameters, we define four scenarios outlined in Figure 2 which allow to distinguish whether seeds originated from selfing*,* intercytotype cross‐fertilization or mixed matings. To test these scenarios, we defined Δ *p*, the deviation of the observed number of paternal genomes (*p*) in the endosperm from their number in endosperms with balanced parental genomic ratios (which are 2*m*:1*p* in sexuals and 4*m*:2*p* in apomicts). We calculated Δ *p* as *p*_observed_ − 1*p* for sexually derived seeds and *p*_observed_ − 2*p* for apomictically derived seeds, with *p* expressed as the multiple of the holoploid maternal genome. To account for Talent's (2009) model, which predicts co‐occurrence of balanced 4*m*:1*p* and 4*m*:2*p* genomic ratios in the same individual, we defined, in addition, for apomictically derived seeds Δ *p*_min_ = min(|1*p *− *p*_observed_|, |2*p *− *p*_observed_|), that is, the lower absolute deviation of *p*_observed_ from 1*p* and 2*p* (e.g., for a *p*_observed_ of 1.1 the absolute deviation from 1*p* and 2p is 0.1 and 0.9, respectively; the value becomes Δ *p*_min_ = 0.1). We related, separately for each ploidy level of the pollen recipient, Δ *p* and Δ *p*_min_, averaged for each flower, to reproductive success by means of GLMMs applying the Laplacian approximation to estimate the model coefficients. Due to the proportional character of reproductive success (both variables are rates), we used the canonical logit‐link function. We used mixed models instead of simple logistic regressions to account for two potential sources of dependence within the data; that is, single pollen recipients were pollinated with pollen of up to four pollen donors, and pollen donors covered three and five ploidy levels for sexual and apomictically reproducing pollen recipients, respectively. We accounted for this dependence by estimating random‐effects intercept terms for each pollen recipient and each paternal cytotype. Analyses were performed using the glmer function (see the previous section).

We tested for mentor effects in the (self‐incompatible) tetraploid sexuals by comparing the paternal genomic contributions to embryos observed in heteroploid crosses (using penta‐ and heptaploid pollen donors) to that in homoploid treatments (homoploid crosses and selfings). A seed obtained in a heteroploid cross whose paternal genomic contribution had a probability of 95% to come from outside the range of values observed for homoploid treatments (defined by the mean ± 2 × standard deviation of these values) was regarded to have originated from intercytotype cross‐fertilization. In the apomicts, we could not apply this approach because either one or two (usually reduced) sperm contribute to the endosperm in *P. puberula* often resulting in a bimodal distribution of paternal genomic contributions. Instead, we tested for shifts in the distribution of paternal genomic contribution in hetero‐ compared to homoploid crosses based on Δ *p*_min_ using the Kolmogorov–Smirnov test as implemented in Statistica 6.1 (StatSoft, Inc. 2002; for better comparability of results, we included also the sexual tetraploids in this test). Differences significant at *p* < .05 were interpreted as indication for the occurrence of cross‐fertilization. Note that—for presumably methodological reasons (see Results for explanation)—observed paternal genomic contributions to the endosperm in homoploid treatments were lower than theoretically expected for both sexually and apomictically derived seeds (Student's t test implemented in Statistica 6.1: *p* < .001 for both comparisons).

## 3 RESULTS

### 3.1 Performance of the FCSS

A clear fluorescence signal for the embryo and endosperm was obtained for 1,804 seeds representing 90.5 % of the morphologically well‐developed seeds subjected to FCSS. The number of embryo nuclei counted per sample, and the coefficient of variation (CV) of embryo peaks ranged between 296 and 3,350 (mean 1,839 ± 331 *SD*) and 3.12–7.37 (5.17 ± 0.73), respectively. Corresponding values for the endosperm were 32–532 (111 ± 42.2) and 1.70–7.21 (3.94 ± 0.72). For details on single measurements, see Appendix S2.

**Figure 3:**
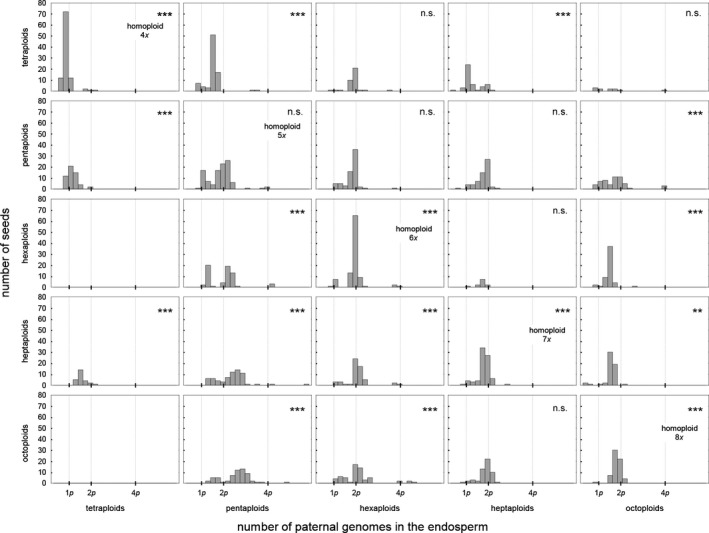
Number of paternal genomes *p* in the endosperm (expressed as the multiple of the holoploid genome of the pollen recipient) of seeds obtained in hetero‐ and homoploid crosses of *Potentilla puberula*. Seeds obtained in crosses upon tetraploid pollen recipients were derived by sexuality, those obtained in the other crosses originated from apomixis. Significant deviation of the observed paternal genomic contribution to the endosperm in the homoploid treatments from the values theoretically expected (1*p* for sexual, 1*p* and 2*p* for apomicts) due to inherent systematic methodological error is indicated. Analogously deviation of the paternal genomic contribution observed for the heteroploid treatments from the values observed in the homoploid crosses is indicated. Pollen recipients are represented by the *x*‐axis; pollen donors by the *y*‐axis. Tetraploids were crossed with tetra‐, penta‐, and heptaploids only. **p < .01, ***p < .001

We observed lower paternal genomic contributions than expected by theory in the tetra‐, hexa‐, hepta‐, and octoploid homoploid crosses (Figure 3). However, we assume that these deviations are artifacts inherent to the applied flow cytometric technique and the way the paternal genomic contribution is calculated. For example, peak indices for sexually derived seeds were raised in average (1.54 ± 0.03 *SD*) compared to 1.50 expected (Matzk, Meister, & Schubert, 2000). This rise may have various causes including suboptimal performance of the flow cytometer, tissue‐specific differential expression of secondary metabolites (e.g., polyphenolics), and/or DNA degeneration owing to shriveled tissues in embryo and endosperm. For sexually derived seeds, the paternal genomic contribution calculates as *2 × embryo ploidy − endosperm ploidy*. As the observed endosperm ploidies were slightly raised compared to the theoretically expected values (endosperm ploidy = embryo ploidy *×* peak index), the estimates for the paternal genomic contribution in turn decreased: for example, in a seed with a tetraploid embryo and an observed peak index of 1.54 the paternal genomic contribution calculates as 2 × 4*x *− 6.16*x* [=4*x* × 1.54] = 1.84*x* (instead of 2*x* expected). Although the difference between the observed and expected peak indices was a moderate 2.67% [=((1.54/1.50)* *− 1) × 100], this deviation is significant here since for mathematical reasons it decreased three times this value the paternal genomic contribution [−8.01% = ((1.84/2.00)* *− 1) × 100) in the given example] (Dobeš, Lückl, et al., 2013).

### 3.2 Reproductive modes

Regular sexuality and apomixis were the main reproductive modes observed for 321 (i.e., 18.6%) and 1,402 seeds (81.4%), respectively. For 81 seeds, irregular reproductive modes including the parthenogenetic development of meiotically reduced egg cells and fertilization of unreduced egg cells were observed (Appendix S2). The majority of individuals (analyzed for at least 10 seeds) showed one predominant pathway of seed production. Twelve and 37 of 58 individuals were sexual and apomictic, respectively. Sexual individuals were exclusively tetraploid, whereas apomicts were of all ploidy levels, although apomixis at the tetraploid level was only rarely observed. This trend of separation of reproductive modes in *P. puberula* on the level of individuals was also evident for those additional 59 individuals analyzed for two to nine seeds, 52 (88.1%) of which formed seeds either via sexuality or apomixis (disregarding rare and aberrant modes).

### 3.3 Self‐compatibility of individuals

**Figure 4:**
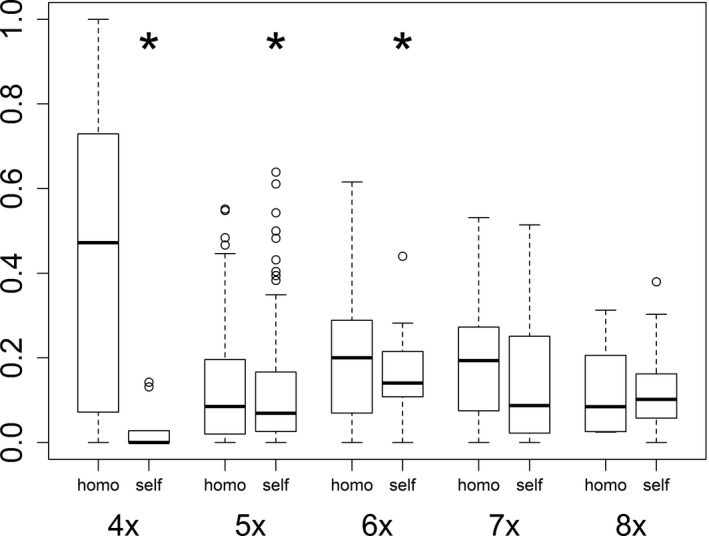
Seed set of *Potentilla puberula* derived from a common garden crossing experiment. 4*x*, 5*x*, 6*x*, 7*x,* and 8*x* refer to the ploidy level of the pollen recipients (tetra‐ to octoploid). Asterisks indicate significant differences in selfed individuals (self) from homoploid crosses (homo). Sample size was 3 populations/12 individuals for tetraploids, 11/59 for pentaploids, for 5/20 hexaploids, for 5/19 heptaploids, and 4/15 for octoploids. Boxes span the range between the 25th and 75th percentile with indicated median, and whiskers extend to 1.5‐fold the interquartile range. Outliers are represented by open circles

Seed set derived from selfed tetraploid sexuals was marginal and significantly lower compared to homoploid crosses (Figure 4, Appendix S3) indicating a high degree of self‐incompatibility. Seed set in selfings of penta‐ and hexaploid apomicts was significantly lower compared to homoploid crosses although still considerable, and in the range of homoploid crosses for hepta‐ and octoploids (Figure 4, Appendix S3).

### 3.4 Pollen quality

Pollen quality varied widely among individuals for all cytotypes: 10.0%–96.0% (median 66.8%) for tetraploid individuals, 0.0%–92.6% (59.4%; penta‐), 11.0%–85.9% (77.2%; hexa‐), 26.2%–89.5% (82.5%; hepta‐), and 0.0–84.4 (65.5%; octoploids). Pollen quality differed significantly among cytotypes (*p* < .001 for all pairwise comparisons; Appendix S4) with highest quality detected in heptaploids, followed by hexa‐, tetra‐, penta‐, and octoploids.

### 3.5 Crossability of cytotypes

Tetraploids had higher, while pentaploids had lower seed set in the homoploid compared to all heteroploid crosses. The effects were explained by both pollen quality and the ploidy of the pollen donor (Table 2). Donor‐pollen quality was positively correlated with seed set in the pentaploids. The correlation was stronger in the homo‐ compared to the heteroploid crosses as seen from the significantly negative interference between ploidy and donor‐pollen quality (negative partial coefficients) for the hexa‐ to octoploids, which describes the slope of the regression for these cytotypes relative to the pentaploids (the model intercept). Paradoxically, pollen quality of donors was negatively correlated with seed set for the tetraploids. This relation mainly applied to the homoploid crosses as partial coefficients were strongly positive for the heteroploid (penta‐ and hexaploid pollen donors) crosses.

**Table 2:** Fixed‐effect coefficients of binomial generalized linear mixed models relating seed set of sexual (tetraploids) and apomictic (penta‐ to octoploids) cytotypes to the ploidy of the crossing partner and the pollen quality of both, the pollen donor and recipient (self‐pollen) in crossings of *Potentilla puberula*

	Coef ± *SE*	*z*–Value	*p*–value
Tetraploids	*N *=* *118, groups = 3/12		
Intercept (homoploid crosses)	0.675 ± 0.797	0.846	.397
Pentaploids	−4.830 ± 0.403	−11.992	**<.001**
Heptaploids	−6.823 ± 1.902	−3.587	**<.001**
Donor‐pollen quality	−2.878 ± 0.391	−7.360	**<.001**
Self‐pollen quality	1.553 ± 1.005	1.545	.122
Pentaploids × donor‐pollen quality	5.598 ± 0.549	10.198	**<.001**
Heptaploids × donor‐pollen quality	8.390 ± 2.274	3.689	**<.001**
Pentaploids	*N *=* *429, groups = 11/59		
Intercept (homoploid crosses)	−2.176 ± 0.327	−6.652	**<.001**
Tetraploids	0.458 ± 0.232	1.972	**.049**
Hexaploids	2.432 ± 0.246	9.878	**<.001**
Heptaploids	1.166 ± 0.272	4.278	**<.001**
Octoploids	0.486 ± 0.202	2.402	**.016**
Donor‐pollen quality	0.921 ± 0.293	3.143	**.002**
Self‐pollen quality	0.031 ± 0.436	0.071	.944
Tetraploids × donor‐pollen quality	−0.588 ± 0.376	−1.566	.117
Hexaploids × donor‐pollen quality	−3.349 ± 0.381	−8.790	**<.001**
Heptaploids × donor‐pollen quality	−1.118 ± 0.389	−2.874	**.004**
Octoploids × donor‐pollen quality	−0.878 ± 0.364	−2.412	**.016**
Hexaploids	*N *=* *201, groups = 5/20		
Intercept (homoploid crosses)	−0.832 ± 0.685	−1.214	.225
Tetraploids	−0.551 ± 0.350	−1.575	.115
Pentaploids	−0.441 ± 0.250	−1.764	.078
Heptaploids	−0.400 ± 0.409	−0.978	.328
Octoploids	0.102 ± 0.248	0.411	.681
Donor‐pollen quality	0.103 ± 0.299	0.344	.731
Self‐pollen quality	−1.147 ± 0.896	−1.280	.201
Tetraploids × donor‐pollen quality	1.689 ± 1.384	1.220	.222
Pentaploids × donor‐pollen quality	0.036 ± 0.365	0.099	.921
Heptaploids × donor‐pollen quality	0.760 ± 0.562	1.354	.176
Octoploids × donor‐pollen quality	0.431 ± 0.563	0.766	.444
Heptaploids	*N *=* *171, groups = 5/19		
Intercept (homoploid crosses)	−2.352 ± 1.033	−2.277	**.023**
Tetraploids	−0.589 ± 0.666	−0.886	.376
Pentaploids	−0.870 ± 0.659	−1.320	.187
Hexaploids	−0.980 ± 1.216	−0.806	.420
Octoploids	−0.391 ± 0.634	−0.616	.538
Donor‐pollen quality	−1.305 ± 0.789	−1.654	.098
Self‐pollen quality	1.784 ± 1.034	1.726	.084
Tetraploids × donor‐pollen quality	0.139 ± 0.865	0.160	.873
Pentaploids × donor‐pollen quality	0.710 ± 0.846	0.840	.401
Hexaploids × donor‐pollen quality	1.062 ± 1.644	0.646	.518
Octoploids × donor‐pollen quality	0.768 ± 0.846	0.907	.364
Octoploids	*N *=* *141, groups = 4/15		
Intercept (homoploid crosses)	−1.616 ± 0.299	−5.410	**<.001**
Tetraploids	−0.080 ± 0.660	−0.121	.904
Pentaploids	−0.234 ± 0.299	−0.783	.434
Hexaploids	0.079 ± 0.595	0.132	.895
Heptaploids	−0.571 ± 0.447	−1.279	.201
Donor‐pollen quality	−0.191 ± 0.465	−0.411	.681
Self‐pollen quality	−0.252 ± 0.393	−0.641	.521
Tetraploids × donor‐pollen quality	−4.385 ± 3.712	−1.181	.237
Pentaploids × donor‐pollen quality	0.139 ± 0.544	0.255	.799
Hexaploids × donor‐pollen quality	0.149 ± 0.876	0.171	.865
Heptaploids × donor‐pollen quality	1.305 ± 0.676	1.930	.054

*p*‐Values given in bold indicate significant differences in heteroploid crosses compared to homoploid crosses (representing the model intercept). Calculations were taken separately for each ploidy level of the pollen recipient. *N* represents the number of pollinated flowers. Groups refer to the number of populations and the number of pollen recipients (individuals) nested within populations. “coef” is the partial coefficients.

In comparison, seed set did not significantly differ between homo‐ and heteroploid crosses in the hexa‐ to octoploids (Table 2) and donor‐pollen quality had no significant influence on this parameter in these crosses. Self‐pollen quality, finally, was unrelated to seed set in any of the five cytotypes.

### 3.6 Selfing versus cross‐fertilization in heteroploid crosses

**Figure 5:**
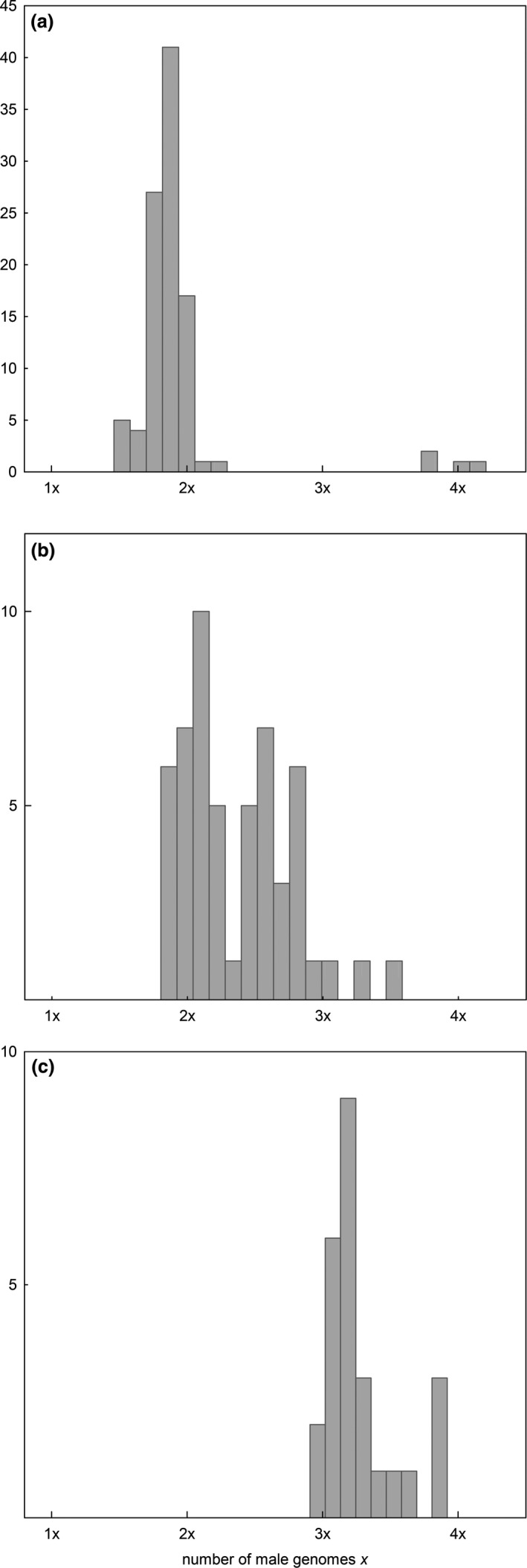
Number of paternal monoploid genomes *x* in the endosperm of seeds obtained from crosses of tetraploid sexual pollen recipients (*N *=* *209) with tetra‐ (a), penta‐ (b), and heptaploid pollen donors (c)

Crosses upon sexuals: the paternal genomic contribution to the endosperm (and to the embryo which receives the same contribution) in the homoploid treatments of the tetraploid sexuals varied between 1.46*x* and 2.21*x* (mean = 1.84*x*;* N *=* *96) with an additional peak ranging from 3.87*x* to 4.31*x* (mean = 4.07*x*;* N *=* *4) (Figure 5a). The first peak corresponds to contributions by meiotically reduced sperm; the second one signifies the contribution by apomeiotically formed sperm and involved four of five analyzed seeds originating from a single flower and was excluded from further analyses. 64.8 % and 97.9 % of the seeds obtained in the heteroploid crosses upon the sexual tetraploids using penta‐ (*N *=* *54, with an observed paternal genomic contribution of 1.80*x*–3.59*x*: Figure 5b) and heptaploid (*N *=* *48, 1.99*x*–3.92*x*: Figure 5c) pollen donors, respectively, had a likelihood of ≤5% to originate from the distribution of paternal genomic contributions observed for the homoploid tetraploid treatments. The result indicates that this progeny likely arose from intercytotype cross‐fertilization. For the remaining seeds obtained in the crosses with pentaploids, we could not distinguish between selfing and cross‐fertilization, possibly to due insufficient discrimination of paternal contributions of penta‐ from those of tetraploids. Intercytotype cross‐fertilization was also evident from the significantly higher Δ *p* in the heteroploid crosses compared to the homoploid treatments (Figures 3; Kolmogorov–Smirnov test; *p* < 0.001 for crosses with both the penta‐ and heptaploid pollen donors). As a consequence of intercytotype cross‐fertilization, the ploidy of the progeny of the sexual tetraploids was on average higher compared to their mothers, indicating the cytological transformation of sexuals by apomicts.

Crosses upon apomicts: Δ *p*_min_ observed for 10 of the 16 performed heteroploid crosses significantly differed from the corresponding value observed in the homoploid treatments, indicating intercytotype cross‐fertilization. Cross‐fertilization only was indicated in all heteroploid crosses upon pentaploids. In contrast, out of the four heteroploid crosses performed upon each apomictic cytotype selfing was inferred for one cross each upon hexa‐ and octoploids and three crosses upon heptaploids (Figure 3). Selfing is suggested by estimates of seed set and Δ *p*_min_ obtained in the heteroploid crosses both being nonsignificantly different from the corresponding values recorded in the homoploid crosses (scenario i in Figure 2).

### 3.7 Parental genomic ratios in the endosperm and their relation to reproductive success

**Figure 6:**
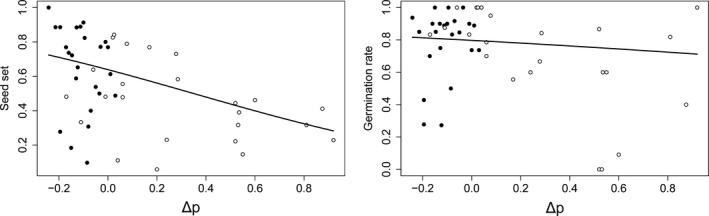
Examination of the requirement for a balanced maternal: paternal genomic ratio in the endosperm of seeds produced by tetraploid sexual individuals of *Potentilla puberula*. Generalized linear mixed models revealed a significant (*p* < .001) and nonsignificant (*p* = .570) relation between seed set and germination rate, respectively, and Δ *p* (averaged for each flower), that is, the deviation of the observed number of parental genomes (*p*) in the endosperm from their number in endosperms with balanced parental genomic ratios (2*m*:1*p*). Black and white dots illustrate flowers subjected to homoploid and heteroploid crosses, respectively

**Figure 7:**
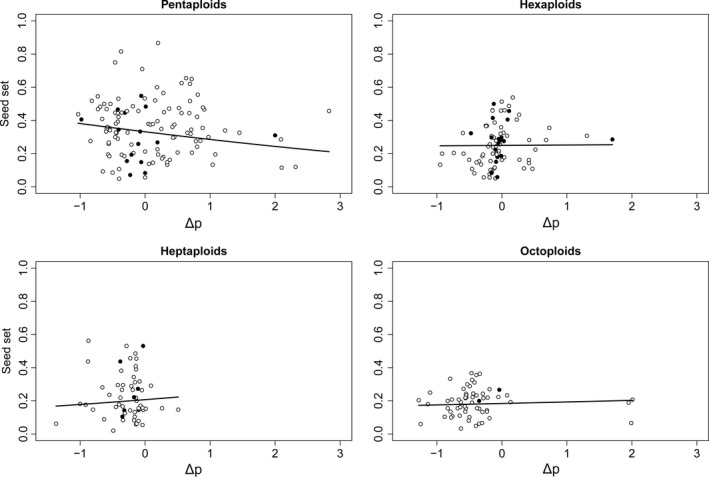
Examination of the requirement for a balanced maternal: paternal genomic ratio in the endosperm of apomictically derived seeds in four cytotypes of *Potentilla puberula*. Generalized linear mixed models revealed a significant relation between seed set and Δ *p* (averaged for each flower), that is, the deviation of the observed number of parental genomes in the endosperm from the balanced number (4*m*:2*p*), for pentaploids (*p* = .002), but not for hexa‐, hepta‐, and octoploids (*p* = .915, .364 and .518). Headings indicate the ploidy level of the pollen recipient. Black and white dots illustrate flowers subjected to homoploid and heteroploid crosses, respectively

Seed set was negatively associated with Δ *p* for tetraploid sexuals (Figure 6) as well as the pentaploids (Figure 7). The effect was stronger in the tetraploids and significant for both cytotypes in the GLMMs (Appendix S5). In contrast, seed set in crosses upon the hexa‐ to octoploid cytotypes was independent from Δ *p* (Figure 7). The same pattern was detected for Δ *p*_min_ (Appendix S5). There was generally no significant relation of Δ *p* (or Δ *p*_min_) to germination rate (Appendix S5).

## 4 DISCUSSION

The genus *Potentilla* in its current taxonomic circumscription (Eriksson, Hibbs, Yoder, Delwiche, & Donoghue, 2003; Paule & Soják, 2009; Potter et al., 2007; Soják, 2008) expresses two principal modes of seed formation, regular sexuality and pseudogamous gametophytic apomixis (Dobeš et al., 2015). The origin of apomicts from sexual relatives is connected to polyploidization of the genome of apomicts. We foremost aimed to answer whether mating among reproductively differentiated cytotypes in *P. puberula* exert reproductive interference on either of these cytotypes and whether negative impacts on reproductive success can be avoided through selfing. We further hypothesized that negative impacts may result from imbalanced parental genomic ratios in the endosperm of sexually and apomictically derived seeds as well as poor pollen quality of donors.

We found indication for unidirectional reproductive interference (Kyogoku, 2015) of the apomictic cytotypes on the sexual one. Tetraploid sexuals suffered decreased seed set upon intercytotype pollinations, a response not recorded for the penta‐ to octoploid apomictic pollen recipients (Table 3). Our data further implied that sexuals likely do not escape fertilization by apomicts through selfing, that is, that mentor effects played no or only a limited role as barrier to gene flow from the apomictic cytotypes toward the sexuals: In agreement with Dobeš, Milosevic, et al. (2013), sexuals were self‐incompatible (Figure 4, Appendix S3), a condition which was maintained upon application of heteroploid pollen loads. The height of the paternal genomic contribution to still obtained seeds in heteroploid crosses upon sexuals using pollen of penta‐ and heptaploids (Table 2) implied that they were likely derived from cross‐fertilization (Figure 5). Our results are in line with Hörandl and Temsch (2009), who observed weak mentor effects in heteroploid crosses of sexual and pseudogamous cytotypes of *Ranunculus auricomus* (Ranunculaceae).

**Table 3:** Characterization of the reproductive system of the five intraspecific ploidy cytotypes recorded for *Potentilla puberula*

Ploidy	Reproductive mode	Breeding system		Association of reduced seed set and imbalanced genomic ratios	Reduced seed set in heteroploid crosses
Tetraploid	Sexual	SI	Cross‐fertilization	Yes	Yes
Pentaploid	Apomictic	SC	Cross‐fertilization	Yes	No^a^
Hexaploid	Apomictic	SC	Mixed	No^a^	No^a^
Heptaploid	Apomictic	SC	Selfing	No^a^	No^a^
Octoploid	Apomictic	SC	Mixed	No^a^	No^a^

SI, self‐incompatible; SC, self‐compatible.

Reproductive mode refers to the prevailing mode of seed production, sexuality or pseudogamous gametophytic apomixis. Breeding system summarizes whether cytotypes are self‐incompatible (SI) or self‐compatible (SC) and the dominant mode of mating upon application of heteroploid pollen on the stigmas of nonemasculated flowers. Reduced seed set in heteroploid crosses refers to comparisons with homoploid crosses.

a Nonsignificant.

We uncovered a more complex mating pattern for the apomictic cytotypes. The paternal genomic contribution to the endosperm in the pentaploids closely reflected the ploidy of the pollen donor (Figure 3), that is, largely matched the values expected for cross‐fertilized endosperm. In contrast, in the hexa‐ to octoploids, selfing occurred aside of cross‐fertilization by heteroploid pollen donors. The flow cytometric data hence suggest that the apomicts formed seeds in the heteroploid crosses either from selfing, cross‐fertilization, or a mixture of both but importantly without significant negative overall effects on seed set.

Seed set of tetraploid sexuals and apomictic pentaploids of *P. puberula* decreased with deviation from the balanced parental genomic ratio. The negative correlation of seed set with Δ *p* (i.e., the deviation of the observed number of paternal genomes *p* in the endosperm from their number in endosperms with balanced parental genomic ratios; Figures 6 and 7) and Δ *p*_min_ (the deviation either closer to 1*p* or 2*p*, respectively; Appendix S5) would agree with the requirement of balanced numbers of parental genomes in the endosperm of both sexuals (Johnston, den Nijs, Peloquin, & Hanneman, 1980) and apomicts (Haig & Westoby, 1991; Talent, 2009). Nevertheless, a considerable number of vital and germinable cross‐fertilized seeds were still formed, suggesting that the sensitivity against unbalanced parental genomic ratios (i.e., specifically genomic imprinting), usually considered a crossing barrier (Johnston et al., 1980), is not absolute in individuals of both reproductive modes. In addition, the decrease in seed set in the hetero‐ compared to the homoploid crosses inferred for the tetraploid sexuals (Table 2) may be alternatively explained by early selection against sired selfed progeny instead of selection against cross‐fertilized progeny (scenario iii in Figure 2). Furthermore, we observed no significant relation between deviation from balanced parental genomic ratios in the endosperm of seeds from the apomictic hexa‐ to octoploid cytotypes.

Unbalanced parental genomic ratios have been repeatedly observed in pseudogamous apomicts, notably in the Hypericaceae, *Hypericum* (Barcaccia et al., 2006), the Poaceae, *Paspalum* (Cáceres, Matzk, Busti, Pupilli, & Arcioni, 2001) and *Tripsacum* (Grimanelli et al., 1997), and the Rosaceae, *Crataegus* (Talent & Dickinson, 2007), *Potentilla* (Dobeš, Lückl, et al., 2013), and *Rubus* (Šarhanová et al., 2012). The multiplicity of parental genomic ratios in the endosperm of apomictically derived seeds observed for these taxa evidenced a relaxation of the parental genomic endosperm balance requirement, but the effect of parental genomic ratios in the endosperm on reproductive success has rarely been quantified for pseudogamous apomicts. Quarin (1999) suggested, based on the lack of a significant difference in reproductive success between heteroploid crosses and selfings, that endosperm formation in *Paspalum notatum* (Poaceae) is independent of the ploidy of the pollen donor in apomictically derived seeds. However, reproductive success was significantly higher in homoploid crosses, indicating negative effects caused by differences in the ploidy of crossing partners. Hence, the existence of some degree of a parental genomic endosperm balance requirement could not—in agreement with our results—be discounted.

Relaxation of the parental genomic endosperm balance requirement in sexual *P. puberula* furthermore meets the postulate that lineages able to modify endosperm development are predisposed to develop apomixis (Grimanelli et al., 1997; Mogie, 1992; Richards, 1997). Several examples favoring this hypothesis are available (Bayer, 1997; Cosendai et al., 2011; Paule, Sharbel, & Dobeš, 2011). Specifically, our data agree with results obtained by Grimanelli et al. (1997), who found endosperms with 4*m*:1*p* genomic ratios in sexual individuals of tetraploid *Tripsacum dactyloides* (Poaceae) and 8*m:*1*p* or 8*m*:2*p* genomic ratios in the conspecific apomicts to be frequent. The authors explained the 4*m*:1*p* genomic ratio by the loss of sensitivity to imprinting.

Despite a negative effect of unbalanced parental genomic ratios in the endosperm on seed set, pentaploids did not suffer reduced seed set in hetero‐ compared to homoploid crosses. Instead, homoploid pentaploid crosses yielded the lowest seed set among apomictic cytotypes (Table 2). In addition to genomic imprinting, pollen quality is a known extrinsic factor influencing reproductive success (Britton & Mogie, 2001; Chacoff, García, & Obeso, 2008; Knight et al., 2005; Larson & Barrett, 2000). The pollen quality of donors was significantly positively related to seed set in crosses upon apomictic pentaploids (but not in other apomicts). This appears a plausible result given inferred predominant outcrossing and the relatively poor quality of the pollen of pentaploids (second to last among the studied cytotypes). The effect of pollen quality on seed set was particularly strong in the homoploid crosses (Table 2) and may explain the observed low seed set.

Inference of the role of pollen quality on seed set in the crosses upon the tetraploid sexuals is more complicated than for apomicts because only penta‐ and heptaploids co‐occurred with sexual tetraploids in our study area in significant numbers to allow carrying outcrossing experiments. Poor pollen quality in *Potentilla* is linked to disturbances of the male meiosis indicated by irregular chromosome pairing, laggards, sticking chromosome bridges, microcyte formation, or degeneration of nuclei (e.g., Asker, 1970; Czapik, 1975; Müntzing, 1928), irregularities particularly expected in odd ploids (Dawe, 1998). Hence, pollen quality rather than parental genomic imbalances may explain the observed reduction in seed set in hetero‐ compared to homoploid crosses upon sexuals. However, pollen quality was highest in heptaploid *P. puberula* and lowest in the odd ploid octoploids. We therefore do not assume that effects on seed set in the heteroploid crosses upon sexuals can be explained by poor pollen quality of odd ploids alone, an interpretation which accords with the significant role of ploidy levels of crossing partners on seed set found in the GLMM analysis (Table 2).

The production of healthy seeds (germination rates did not decrease with Δ *p*: Appendix S5) derived from cross‐fertilization suggests that apomicts potentially usurp progeny of sexuals. Cross‐fertilization may lead to the reproductive transformation of sexuals by apomicts as apomixis is known to be transmitted by pollen (Asker, 1980; Grimanelli, Leblanc, Perotti, & Grossniklaus, 2001; Ozias‐Akins & Van Dijk, 2007). In case that such intercytotype offspring is vital and fertile, reproductive (and cytological) transformation can speed up replacement of sexuals by apomicts, although the actual outcome of competition among reproductive modes depends on a series of factors as rates of penetrance of apomixis, the male and female fitness of cytotypes, pollen and seed dispersal abilities, existence of crossing barriers, or starting frequencies of cytotypes in the population (Britton & Mogie, 2001; Joshi & Moddy, 1995; Mogie, 2011), conditions which need to be established simultaneously for a concrete situation.

In summary, we observed contrasting effects of reproductive interference on seed set in *P. puberula*: negative effects for the sexual tetraploids; positive net effects for the apomictic pentaploids; and nonsignificant effects on the hexa‐ to octoploid apomicts. Net effects applied to all apomictic cytotypes irrespective of cytology and reproductive mode of the pollen donor. These results have potential implications for the co‐existence of cytotypes: Tetraploid sexuals suffer reduced fertility from the presence of apomicts due to cross‐pollination. Moreover, the production of healthy seeds derived from cross‐fertilization suggests that apomicts may usurp the sexual's progeny potentially involving the reproductive transformation of sexuals by apomicts. Whether even ploid apomicts exert analogous negative effects on sexuals as their odd ploid counterparts remains an open question, as our study design and numbers of available individuals in reproductively mixed populations did not allow to include these apomictic cytotypes in crosses upon sexuals. However, the data on pollen quality of cytotypes do not suggest that even ploid apomicts would perform significantly better as pollen donors than odd ploids. At least the low frequencies in natural population of the latter (5.4% hexa‐ and 3.7% octoploids compared to 63.7% penta‐ and 7.6% heptaploids within the study area) do not indicate that they play a key role in shaping the distribution of the sexuals.

In contrast to sexuals, the self‐compatible apomictic cytotypes either avoided intercytotype cross‐fertilization by selfing, were insensitive to intercytotype cross‐fertilization, or may even have benefitted from higher quality of heteroploid cross‐pollen compared to self‐pollen. The inferred asymmetrical reproductive interference might drive displacement of sexuals by apomicts and may explain the observed mutual avoidance of sexual and apomictic cytotypes in *P. puberula*. An analogous conclusion was recently drawn by Hersh, Grimm, and Whitton (2016) in a study of sexual and apomictic North American *Crepis* species (Asteraceae).

Rates of cross‐pollination and cross‐fertilization may considerably differ between an experimental study and natural populations. Indeed, cross‐fertilization of sexuals by apomicts in the field was negligible in cytologically mixed populations of *P. puberula* in East Tyrol (Dobeš, Milosevic, et al., 2013). Although seed set depended on differences in the ploidy of crossing partners and pollen quality of donors in the ex situ experiment, considerable numbers of (germinable) seeds originated from cross‐fertilization of sexuals by apomicts. Consequently, almost lack of intercytotype cross‐fertilizations in the field study cannot be explained by these factors alone and mechanisms preventing cross‐fertilization of sexual by apomicts likely are active in natural populations of *P. puberula*. For instance, pollen precedence may explain the difference. Pollen precedence was found to modify results observed in single‐source crossings. In *Centaurea* (Asteraceae), intercytotype cross‐fertilizations observed in single‐source crossings of a di‐ and tetraploid species were suppressed by experimental pollinations with a mixture of pollen of both species indicating that homoploid pollen takes precedence over heteroploid pollen (Koutecky, Badurova, Štech, Kosnar, & Karásek, 2011). Intercytotype hybrids were almost absent in natural mixed populations of *Centaurea*, an observation explained by this mechanism. Although we consider our results an important parameter to understand the dynamics of cytologically and reproductively mixed populations, additional factors influencing reproductive success and governing reproductive interference need to be considered in order to predict the relative success and fate of cytotypes. In particular, the actual degree of intercytotype cross‐fertilization depends on factors like the overlap in flowering time among cytotypes, the activity and the specificity of pollen vectors, the possible occurrence of pollen precedence upon mixed pollinations, or the spatial arrangement and frequency of cytotypes within populations. In addition, difference in the fitness of cytotypes in terms of female fertility and vigor of progeny and adults as well as the fitness and the reproductive mode of hybrid offspring will modify the success of cytotypes in mixed populations.

## CONFLICT OF INTEREST

The authors declare that they have no competing interests.

## AUTHORS’ CONTRIBUTIONS

CD conceived and designed research. SS, YF, DP, and CS conducted experiments. CD, YF, and KH analyzed data. CD and KH wrote the manuscript. All authors read and approved the manuscript.
