# Supplementary material for: Asymmetric reproductive interference: The consequences of cross‐pollination on reproductive success in sexual–apomictic populations of Potentilla puberula (Rosaceae)
Source: Ecol Evol. 2017 Nov 28;8(1):365–81. doi: 10.1002/ece3.3684 (PMC5756837; doi:10.1002/ece3.3684)
Supplement: Supplementary file 1 [file ECE3-8-365-s001.docx]

**Online Resource 1** Descriptive statistics of a controlled *ex situ* crossing experiment carried out on 133 individuals of *Potentilla puberula* representing 11 populations from East Tyrol, Austria. For each “pollen recipient” the “population” name, “individual” number, generative “ploidy” *x* and “pollen quality” are provided. “Treatment” differentiates between homoploid crosses (*IA*), selfings (*S*), and heteroploid crosses (*IE*). “Seed set” and “germination rate” are the mean of values for the single manipulations observed for a treatment.

| **population** | **individual** | **ploidy** | **treatment** | ***N* manipulations** | **seed set [%]** | **germination rate [%]** | **pollen quality [%]** |
| --- | --- | --- | --- | --- | --- | --- | --- |
| Raut | 1 | 5 | S | 1 | 0.00 | na | 92.56 |
|  | 1 | 5 | IE | 5 | 30.00 | na | 92.56 |
|  | 5 | 4 | S | 1 | 0.00 | na | 91.39 |
|  | 5 | 4 | IE | 5 | 47.27 | 86.54 | 91.39 |
|  | 5 | 4 | IA | 4 | 63.64 | na | 91.39 |
|  | 8 | 4 | S | 1 | 2.78 | na | 86.71 |
|  | 8 | 4 | IE | 5 | 35.00 | na | 86.71 |
|  | 8 | 4 | IA | 3 | 59.26 | 88.33 | 86.71 |
|  | 12 | 5 | S | 1 | 39.47 | 76.90 | 86.11 |
|  | 12 | 5 | IE | 5 | 46.32 | 64.66 | 86.11 |
|  | 16 | 4 | S | 1 | 0.00 | na | 83.94 |
|  | 16 | 4 | IE | 5 | 46.96 | na | 83.94 |
|  | 16 | 4 | IA | 4 | 51.09 | 61.00 | 83.94 |
|  | 31 | 4 | S | 1 | 13.16 | na | 87.18 |
|  | 31 | 4 | IA | 4 | 26.32 | 80.83 | 87.18 |
|  | 31 | 4 | IE | 5 | 60.53 | 88.34 | 87.18 |
|  | 37 | 5 | S | 1 | 37.89 | 55.60 | 84.43 |
|  | 37 | 5 | IE | 5 | 21.47 | na | 84.43 |
|  | 46 | 5 | IE | 5 | 17.21 | 83.80 | 85.40 |
|  | 46 | 5 | S | 1 | 6.98 | 0.00 | 85.40 |
|  | 47 | 4 | IE | 5 | 85.00 | 89.98 | 79.73 |
|  | 47 | 4 | S | 1 | 5.00 | na | 79.73 |
|  | 47 | 4 | IA | 4 | 67.50 | 70.05 | 79.73 |
|  | 48 | 5 | S | 1 | 63.89 | 85.00 | 71.35 |
|  | 48 | 5 | IE | 5 | 39.44 | 70.66 | 71.35 |
| Zabernig | 1 | 4 | S | 1 | 2.78 | na | 56.32 |
|  | 1 | 4 | IE | 8 | 13.89 | na | 56.32 |
|  | 1 | 4 | IA | 4 | 35.42 | 80.08 | 56.32 |
|  | 3 | 5 | IE | 8 | 8.33 | na | 61.15 |
|  | 3 | 5 | S | 1 | 0.00 | na | 61.15 |
|  | 3 | 5 | IA | 3 | 4.32 | 100.00 | 61.15 |
|  | 6 | 5 | S | 1 | 0.00 | na | 36.24 |
|  | 6 | 5 | IE | 8 | 9.15 | na | 36.24 |
|  | 6 | 5 | IA | 3 | 2.44 | na | 36.24 |
|  | 8 | 4 | IE | 8 | 15.44 | na | 44.30 |
|  | 8 | 4 | IA | 4 | 45.59 | na | 44.30 |
|  | 8 | 4 | S | 1 | 0.00 | na | 44.30 |
|  | 14 | 4 | IE | 8 | 11.81 | na | 75.94 |
|  | 14 | 4 | IA | 4 | 25.27 | 75.00 | 75.94 |
|  | 14 | 4 | S | 1 | 17.58 | 87.50 | 75.94 |
|  | 17 | 4 | IA | 4 | 42.45 | na | 44.27 |
|  | 17 | 4 | S | 1 | 0.00 | na | 44.27 |
|  | 17 | 4 | IE | 8 | 28.77 | 64.80 | 44.27 |
|  | 22 | 4 | IA | 4 | 29.88 | 60.40 | 95.97 |
|  | 22 | 4 | IE | 8 | 21.34 | na | 95.97 |
|  | 22 | 4 | S | 1 | 0.00 | na | 95.97 |
|  | 26 | 7 | IA | 2 | 47.69 | 87.50 | 84.62 |
|  | 26 | 7 | IE | 9 | 31.45 | na | 84.62 |
|  | 28 | 7 | IE | 10 | 19.45 | na | 75.86 |
|  | 28 | 7 | S | 1 | 27.27 | na | 75.86 |
|  | 28 | 7 | IA | 2 | 24.55 | 70.00 | 75.86 |
|  | 32 | 7 | IE | 10 | 12.35 | 46.31 | 89.08 |
|  | 32 | 7 | S | 1 | 11.76 | 100.00 | 89.08 |
|  | 32 | 7 | IA | 2 | 22.06 | 68.75 | 89.08 |
|  | 33 | 5 | S | 1 | 0.00 | na | 66.47 |
|  | 33 | 5 | IE | 8 | 39.31 | 91.55 | 66.47 |
|  | 33 | 5 | IA | 4 | 17.81 | 88.75 | 66.47 |
|  | 41 | 5 | S | 1 | 0.00 | na | 65.38 |
|  | 41 | 5 | IA | 4 | 24.11 | 88.78 | 65.38 |
|  | 41 | 5 | IE | 8 | 18.30 | na | 65.38 |
|  | 42 | 5 | IE | 8 | 20.26 | 80.33 | 74.23 |
|  | 42 | 5 | S | 1 | 5.17 | 100.00 | 74.23 |
|  | 42 | 5 | IA | 4 | 19.83 | 95.83 | 74.23 |
|  | 302 | 5 | S | 1 | 9.52 | 100.00 | na |
|  | 302 | 5 | IE | 5 | 25.71 | 64.76 | na |
|  | 302 | 5 | IA | 2 | 11.90 | 0.00 | na |
| Groder | 5 | 5 | S | 1 | 0.00 | na | 67.02 |
|  | 5 | 5 | IE | 5 | 9.00 | na | 67.02 |
|  | 5 | 5 | IA | 4 | 20.63 | 74.18 | 67.02 |
|  | 6 | 5 | IE | 5 | 10.23 | na | 50.32 |
|  | 6 | 5 | S | 1 | 0.00 | na | 50.32 |
|  | 6 | 5 | IA | 3 | 1.55 | na | 50.32 |
|  | 9 | 4 | IE | 5 | 3.23 | na | 78.71 |
|  | 9 | 4 | IA | 4 | 18.55 | na | 78.71 |
|  | 9 | 4 | S | 1 | 0.00 | na | 78.71 |
|  | 12 | 4 | IA | 4 | 30.28 | na | 42.68 |
|  | 12 | 4 | S | 1 | 0.00 | na | 42.68 |
|  | 12 | 4 | IE | 5 | 7.32 | na | 42.68 |
|  | 17 | 5 | S | 1 | 3.13 | 0.00 | 47.88 |
|  | 17 | 5 | IA | 4 | 14.84 | na | 47.88 |
|  | 17 | 5 | IE | 5 | 35.63 | 76.32 | 47.88 |
|  | 18 | 4 | IA | 4 | 0.00 | na | 80.38 |
|  | 18 | 4 | IE | 5 | 0.00 | na | 80.38 |
|  | 18 | 4 | S | 1 | 0.00 | na | 80.38 |
|  | 22 | 4 | IA | 4 | 55.24 | 79.53 | 83.87 |
|  | 22 | 4 | S | 1 | 0.00 | na | 83.87 |
|  | 22 | 4 | IE | 4 | 41.20 | 59.75 | 83.87 |
|  | 33 | 4 | IA | 4 | 65.00 | 50.70 | 77.27 |
|  | 33 | 4 | S | 1 | 14.29 | na | 77.27 |
|  | 33 | 4 | IE | 5 | 18.86 | na | 77.27 |
|  | 49 | 5 | IE | 5 | 23.23 | na | 52.99 |
|  | 49 | 5 | S | 1 | 3.23 | 100.00 | 52.99 |
|  | 49 | 5 | IA | 4 | 20.16 | na | 52.99 |
| Erlbach | 5 | 8 | IA | 4 | 5.49 | na | 72.88 |
|  | 5 | 8 | S | 1 | 4.88 | na | 72.88 |
|  | 5 | 8 | IE | 10 | 2.93 | na | 72.88 |
|  | 6 | 7 | IE | 10 | 19.33 | na | 89.52 |
|  | 6 | 7 | IA | 4 | 9.80 | na | 89.52 |
|  | 6 | 7 | S | 1 | 50.42 | 87.50 | 89.52 |
|  | 12 | 5 | S | 1 | 27.78 | 11.10 | 71.03 |
|  | 12 | 5 | IA | 1 | 33.33 | 100.00 | 71.03 |
|  | 12 | 5 | IE | 10 | 17.78 | na | 71.03 |
|  | 13 | 7 | IA | 2 | 9.76 | na | 63.70 |
|  | 13 | 7 | IE | 8 | 14.94 | na | 63.70 |
|  | 13 | 7 | S | 1 | 4.88 | 50.00 | 63.70 |
|  | 14 | 7 | IA | 2 | 7.29 | 100.00 | 78.57 |
|  | 14 | 7 | S | 1 | 10.42 | 80.00 | 78.57 |
|  | 14 | 7 | IE | 10 | 10.00 | na | 78.57 |
|  | 15 | 5 | IE | 9 | 38.52 | 62.44 | 63.08 |
|  | 15 | 5 | S | 1 | 0.00 | na | 63.08 |
|  | 15 | 5 | IA | 1 | 46.67 | 61.50 | 63.08 |
|  | 19 | 8 | IE | 10 | 16.88 | na | na |
|  | 19 | 8 | IA | 1 | 31.25 | na | na |
|  | 19 | 8 | S | 1 | 22.92 | 33.30 | na |
|  | 20 | 5 | S | 1 | 34.48 | 40.00 | 70.80 |
|  | 20 | 5 | IE | 10 | 46.90 | na | 70.80 |
|  | 21 | 5 | S | 1 | 15.00 | 50.00 | 48.13 |
|  | 21 | 5 | IE | 10 | 22.50 | 60.78 | 48.13 |
|  | 22 | 8 | IE | 8 | 18.43 | na | na |
|  | 22 | 8 | IA | 1 | 2.68 | 0.00 | na |
|  | 22 | 8 | S | 1 | 10.72 | 50.00 | na |
|  | 23 | 7 | S | 1 | 0.00 | na | 84.21 |
|  | 23 | 7 | IA | 4 | 10.61 | na | 84.21 |
|  | 23 | 7 | IE | 8 | 15.91 | 94.86 | 84.21 |
|  | 24 | 8 | IE | 10 | 15.31 | na | na |
|  | 24 | 8 | S | 1 | 18.37 | 66.70 | na |
|  | 24 | 8 | IA | 1 | 6.12 | 33.30 | na |
|  | 26 | 8 | IE | 10 | 17.88 | na | 50.78 |
|  | 26 | 8 | S | 1 | 27.27 | 80.00 | 50.78 |
|  | 26 | 8 | IA | 1 | 21.21 | 14.30 | 50.78 |
|  | 28 | 5 | IE | 10 | 18.37 | na | 87.07 |
|  | 28 | 5 | IA | 2 | 12.79 | 75.00 | 87.07 |
|  | 28 | 5 | S | 1 | 18.60 | 100.00 | 87.07 |
|  | 32 | 7 | IA | 2 | 4.65 | na | 72.22 |
|  | 32 | 7 | S | 1 | 27.91 | 100.00 | 72.22 |
|  | 32 | 7 | IE | 10 | 7.44 | na | 72.22 |
| Lana | 4 | 5 | S | 1 | 15.48 | 75.00 | 29.81 |
|  | 4 | 5 | IE | 5 | 4.95 | na | 29.81 |
|  | 4 | 5 | IA | 3 | 13.42 | na | 29.81 |
|  | 6 | 5 | IA | 4 | 1.08 | na | 73.87 |
|  | 6 | 5 | IE | 5 | 11.18 | na | 73.87 |
|  | 6 | 5 | S | 1 | 10.75 | na | 73.87 |
|  | 17 | 6 | IE | 5 | 0.00 | na | 82.76 |
|  | 17 | 6 | S | 1 | 0.00 | na | 82.76 |
|  | 17 | 6 | IA | 4 | 0.00 | na | 82.76 |
|  | 20 | 5 | IE | 5 | 10.29 | 0.00 | 51.46 |
|  | 20 | 5 | S | 1 | 54.29 | 78.90 | 51.46 |
|  | 20 | 5 | IA | 3 | 17.14 | na | 51.46 |
|  | 23 | 6 | IA | 4 | 13.82 | 22.90 | 77.59 |
|  | 23 | 6 | IE | 5 | 15.26 | na | 77.59 |
|  | 23 | 6 | S | 1 | 13.16 | 40.00 | 77.59 |
|  | 29 | 5 | IA | 4 | 18.57 | na | 67.26 |
|  | 29 | 5 | IE | 5 | 18.86 | na | 67.26 |
|  | 29 | 5 | S | 1 | 11.43 | 100.00 | 67.26 |
|  | 33 | 6 | IE | 4 | 12.98 | 20.83 | 75.18 |
|  | 33 | 6 | S | 1 | 21.37 | 14.30 | 75.18 |
|  | 33 | 6 | IA | 4 | 19.85 | 30.48 | 75.18 |
|  | 37 | 5 | S | 1 | 0.00 | na | 59.59 |
|  | 37 | 5 | IA | 4 | 3.81 | na | 59.59 |
|  | 37 | 5 | IE | 5 | 13.09 | na | 59.59 |
|  | 41 | 6 | IA | 4 | 28.77 | 67.95 | 11.03 |
|  | 41 | 6 | S | 1 | 13.21 | 28.60 | 11.03 |
|  | 41 | 6 | IE | 5 | 18.11 | 59.16 | 11.03 |
|  | 46 | 6 | IE | 5 | 13.58 | na | 46.15 |
|  | 46 | 6 | S | 1 | 0.00 | na | 46.15 |
|  | 46 | 6 | IA | 4 | 19.32 | na | 46.15 |
| Stein | 4 | 5 | S | 1 | 11.27 | 100.00 | 41.67 |
|  | 4 | 5 | IE | 5 | 39.44 | 66.94 | 41.67 |
|  | 5 | 5 | IE | 5 | 29.44 | 37.12 | 68.94 |
|  | 5 | 5 | S | 1 | 16.67 | 33.30 | 68.94 |
|  | 7 | 5 | IE | 5 | 23.74 | 26.84 | 56.05 |
|  | 7 | 5 | S | 1 | 15.38 | 50.00 | 56.05 |
|  | 8 | 5 | S | 1 | 26.09 | 75.00 | 56.02 |
|  | 8 | 5 | IE | 5 | 32.17 | 58.22 | 56.02 |
|  | 9 | 5 | IE | 5 | 40.00 | 79.74 | 24.26 |
|  | 9 | 5 | S | 1 | 0.00 | na | 24.26 |
|  | 14 | 8 | S | 1 | 6.35 | 0.00 | 77.44 |
|  | 14 | 8 | IE | 5 | 7.94 | 60.66 | 77.44 |
|  | 15 | 8 | S | 1 | 11.85 | 62.50 | 81.40 |
|  | 15 | 8 | IE | 5 | 11.26 | 56.04 | 81.40 |
|  | 16 | 8 | IE | 5 | 11.15 | na | 65.15 |
|  | 16 | 8 | S | 1 | 15.38 | 16.70 | 65.15 |
|  | 17 | 8 | S | 1 | 9.43 | 40.00 | 65.93 |
|  | 17 | 8 | IE | 5 | 21.13 | 42.52 | 65.93 |
|  | 19 | 8 | IE | 5 | 18.31 | 44.06 | 84.38 |
|  | 19 | 8 | S | 1 | 1.69 | 0.00 | 84.38 |
|  | 22 | 5 | IA | 4 | 5.00 | na | 38.03 |
|  | 22 | 5 | IE | 3 | 9.52 | na | 38.03 |
|  | 22 | 5 | S | 1 | 0.00 | na | 38.03 |
|  | 27 | 5 | S | 1 | 29.79 | 64.30 | 74.05 |
|  | 27 | 5 | IE | 3 | 5.67 | na | 74.05 |
|  | 27 | 5 | IA | 4 | 10.11 | na | 74.05 |
|  | 28 | 7 | S | 1 | 2.63 | 0.00 | 85.98 |
|  | 28 | 7 | IE | 5 | 10.00 | na | 85.98 |
|  | 28 | 7 | IA | 2 | 13.16 | 8.35 | 85.98 |
|  | 29 | 7 | S | 1 | 0.00 | na | 82.48 |
|  | 29 | 7 | IA | 2 | 14.49 | 60.70 | 82.48 |
|  | 29 | 7 | IE | 4 | 10.87 | na | 82.48 |
|  | 30 | 5 | S | 1 | 0.00 | na | na |
|  | 30 | 5 | IA | 4 | 25.81 | na | na |
|  | 30 | 5 | IE | 3 | 39.78 | 96.67 | na |
|  | 38 | 5 | IE | 3 | 15.71 | na | 30.37 |
|  | 38 | 5 | IA | 4 | 3.21 | na | 30.37 |
|  | 38 | 5 | S | 1 | 4.29 | 66.70 | 30.37 |
|  | 45 | 5 | IA | 4 | 4.55 | 0.00 | 58.75 |
|  | 45 | 5 | S | 1 | 4.55 | 100.00 | 58.75 |
|  | 45 | 5 | IE | 3 | 12.88 | 80.00 | 58.75 |
|  | 49 | 7 | IA | 2 | 0.69 | na | 85.00 |
|  | 49 | 7 | S | 1 | 0.00 | na | 85.00 |
|  | 49 | 7 | IE | 5 | 5.28 | na | 85.00 |
| Innervillgraten | 1 | 6 | IE | 16 | 18.75 | 66.10 | 81.82 |
|  | 1 | 6 | IA | 2 | 14.29 | 66.65 | 81.82 |
|  | 1 | 6 | S | 1 | 8.16 | na | 81.82 |
|  | 2 | 6 | S | 1 | 23.08 | 88.90 | 62.86 |
|  | 2 | 6 | IA | 2 | 16.67 | 83.35 | 62.86 |
|  | 2 | 6 | IE | 15 | 25.98 | na | 62.86 |
|  | 5 | 6 | S | 1 | 11.11 | 80.00 | 77.21 |
|  | 5 | 6 | IA | 2 | 18.89 | 100.00 | 77.21 |
|  | 5 | 6 | IE | 16 | 18.06 | na | 77.21 |
|  | 7 | 6 | S | 1 | 9.01 | 100.00 | 85.42 |
|  | 7 | 6 | IA | 4 | 17.12 | 88.88 | 85.42 |
|  | 7 | 6 | IE | 13 | 17.05 | 90.12 | 85.42 |
|  | 11 | 5 | IE | 12 | 30.30 | 34.71 | 51.56 |
|  | 11 | 5 | S | 1 | 18.18 | 100.00 | 51.56 |
|  | 11 | 5 | IA | 1 | 14.55 | 33.30 | 51.56 |
|  | 14 | 4 | S | 1 | 4.35 | na | 26.32 |
|  | 14 | 4 | IE | 13 | 37.29 | na | 26.32 |
|  | 14 | 4 | IA | 2 | 13.04 | na | 26.32 |
|  | 15 | 4 | S | 1 | 4.76 | na | 14.89 |
|  | 15 | 4 | IE | 15 | 54.29 | 81.55 | 14.89 |
|  | 15 | 4 | IA | 1 | 9.52 | 100.00 | 14.89 |
|  | 20 | 8 | IE | 6 | 10.54 | na | na |
|  | 20 | 8 | S | 1 | 6.12 | 33.30 | na |
|  | 23 | 7 | IE | 13 | 14.53 | na | 76.69 |
|  | 23 | 7 | IA | 1 | 22.22 | 55.60 | 76.69 |
|  | 25 | 5 | IE | 15 | 31.72 | 65.02 | 80.88 |
|  | 25 | 5 | S | 1 | 6.90 | 100.00 | 80.88 |
|  | 25 | 5 | IA | 2 | 43.10 | 47.90 | 80.88 |
|  | 27 | 8 | IE | 13 | 21.88 | na | 23.97 |
|  | 27 | 8 | S | 1 | 22.22 | 80.00 | 23.97 |
|  | 27 | 8 | IA | 1 | 20.00 | 25.00 | 23.97 |
|  | 29 | 5 | IE | 10 | 12.22 | na | 57.50 |
|  | 29 | 5 | S | 1 | 16.67 | 100.00 | 57.50 |
|  | 29 | 5 | IA | 3 | 4.63 | na | 57.50 |
|  | 32 | 6 | S | 1 | 2.65 | 0.00 | 58.33 |
|  | 32 | 6 | IE | 14 | 28.99 | na | 58.33 |
|  | 32 | 6 | IA | 4 | 37.80 | na | 58.33 |
|  | 36 | 5 | IA | 2 | 23.73 | 56.25 | 62.94 |
|  | 36 | 5 | IE | 15 | 22.37 | 55.38 | 62.94 |
|  | 36 | 5 | S | 1 | 6.78 | 0.00 | 62.94 |
|  | 37 | 7 | IE | 13 | 8.33 | na | 85.71 |
|  | 37 | 7 | S | 1 | 2.08 | 100.00 | 85.71 |
|  | 38 | 7 | IE | 9 | 9.84 | na | 40.94 |
|  | 38 | 7 | IA | 1 | 14.29 | 60.00 | 40.94 |
|  | 38 | 7 | S | 1 | 0.00 | na | 40.94 |
|  | 41 | 8 | IA | 1 | 26.67 | 0.00 | na |
|  | 41 | 8 | S | 1 | 16.67 | 40.00 | na |
|  | 41 | 8 | IE | 15 | 18.22 | na | na |
|  | 43 | 4 | IE | 16 | 56.72 | na | 27.47 |
|  | 43 | 4 | IA | 1 | 2.50 | 100.00 | 27.47 |
|  | 44 | 8 | S | 1 | 13.51 | 0.00 | 6.96 |
|  | 44 | 8 | IE | 14 | 11.97 | na | 6.96 |
|  | 44 | 8 | IA | 2 | 6.76 | 37.50 | 6.96 |
|  | 45 | 5 | IE | 13 | 13.68 | na | 5.22 |
|  | 45 | 5 | S | 1 | 0.00 | na | 5.22 |
|  | 45 | 5 | IA | 4 | 15.00 | 69.75 | 5.22 |
|  | 47 | 4 | S | 1 | 5.19 | na | 19.69 |
|  | 47 | 4 | IA | 1 | 2.60 | 100.00 | 19.69 |
|  | 47 | 4 | IE | 15 | 38.10 | na | 19.69 |
|  | 48 | 4 | IE | 15 | 50.00 | 85.94 | 10.00 |
|  | 48 | 4 | IA | 1 | 8.82 | 100.00 | 10.00 |
|  | 48 | 4 | S | 1 | 5.88 | na | 10.00 |
| Virgen | 2 | 7 | IE | 4 | 6.45 | na | 85.83 |
|  | 2 | 7 | S | 1 | 29.03 | na | 85.83 |
|  | 4 | 7 | IE | 4 | 26.14 | 70.68 | 75.97 |
|  | 4 | 7 | S | 1 | 36.36 | 84.60 | 75.97 |
|  | 6 | 5 | S | 1 | 5.88 | 100.00 | 38.60 |
|  | 6 | 5 | IE | 5 | 45.88 | 88.98 | 38.60 |
|  | 13 | 7 | IE | 4 | 1.92 | na | 26.17 |
|  | 13 | 7 | S | 1 | 7.69 | 33.30 | 26.17 |
|  | 17 | 7 | S | 1 | 16.16 | 33.30 | 86.51 |
|  | 17 | 7 | IE | 3 | 6.73 | na | 86.51 |
|  | 22 | 5 | S | 1 | 3.94 | 0.00 | 58.48 |
|  | 22 | 5 | IE | 5 | 25.25 | 59.52 | 58.48 |
|  | 24 | 5 | S | 1 | 32.50 | 100.00 | 36.00 |
|  | 24 | 5 | IE | 5 | 32.00 | 86.58 | 36.00 |
|  | 26 | 7 | IE | 4 | 15.57 | 90.83 | 81.65 |
|  | 26 | 7 | S | 1 | 37.74 | 53.30 | 81.65 |
|  | 28 | 5 | IE | 5 | 23.20 | na | 59.72 |
|  | 28 | 5 | S | 1 | 6.00 | 33.30 | 59.72 |
| Arnig | 2 | 5 | S | 1 | 20.15 | 100.00 | 20.00 |
|  | 2 | 5 | IE | 2 | 25.19 | na | 20.00 |
|  | 6 | 5 | IE | 2 | 26.42 | 80.00 | 9.03 |
|  | 6 | 5 | S | 1 | 3.77 | 100.00 | 9.03 |
|  | 9 | 5 | S | 1 | 7.77 | 25.00 | 41.82 |
|  | 9 | 5 | IE | 2 | 29.13 | 41.65 | 41.82 |
|  | 10 | 5 | IE | 1 | 32.73 | 27.80 | 50.33 |
|  | 12 | 5 | IE | 1 | 14.55 | 85.70 | 59.20 |
|  | 12 | 5 | S | 1 | 1.82 | na | 59.20 |
|  | 14 | 6 | S | 1 | 44.00 | 81.80 | 78.29 |
|  | 14 | 6 | IE | 5 | 16.80 | na | 78.29 |
|  | 16 | 6 | S | 1 | 21.95 | 28.60 | 68.46 |
|  | 16 | 6 | IE | 5 | 22.44 | 72.00 | 68.46 |
| Schrottendorf | 2 | 8 | IE | 8 | 17.00 | na | 53.08 |
|  | 2 | 8 | S | 1 | 38.00 | 0.00 | 53.08 |
|  | 4 | 5 | S | 1 | 4.55 | 33.30 | 48.80 |
|  | 4 | 5 | IE | 4 | 19.70 | 47.50 | 48.80 |
|  | 5 | 6 | IE | 6 | 18.84 | 77.65 | 80.42 |
|  | 5 | 6 | S | 1 | 21.74 | 60.00 | 80.42 |
|  | 10 | 6 | S | 1 | 28.21 | 100.00 | 85.93 |
|  | 10 | 6 | IE | 6 | 15.81 | na | 85.93 |
|  | 11 | 5 | S | 1 | 0.00 | na | 30.87 |
|  | 11 | 5 | IE | 2 | 24.42 | 48.80 | 30.87 |
|  | 13 | 5 | IE | 4 | 16.43 | 71.98 | 35.59 |
|  | 13 | 5 | S | 1 | 14.29 | 50.00 | 35.59 |
|  | 21 | 5 | S | 1 | 21.95 | 87.50 | 12.59 |
|  | 21 | 5 | IE | 4 | 18.90 | 62.38 | 12.59 |
|  | 29 | 5 | IE | 4 | 14.86 | 65.08 | 33.15 |
|  | 29 | 5 | S | 1 | 0.00 | na | 33.15 |
|  | 30 | 6 | S | 1 | 10.81 | 57.10 | 44.77 |
|  | 30 | 6 | IE | 6 | 7.43 | na | 44.77 |
| Oberburgfrieden | 6 | 5 | IE | 5 | 10.90 | na | 68.18 |
|  | 6 | 5 | S | 1 | 39.83 | 86.70 | 68.18 |
|  | 8 | 5 | S | 1 | 7.55 | 100.00 | 85.27 |
|  | 8 | 5 | IE | 5 | 15.09 | 86.12 | 85.27 |
|  | 13 | 6 | IE | 5 | 7.01 | na | 78.17 |
|  | 13 | 6 | IA | 4 | 9.49 | na | 78.17 |
|  | 13 | 6 | S | 1 | 18.98 | 100.00 | 78.17 |
|  | 14 | 5 | IE | 5 | 10.00 | na | na |
|  | 14 | 5 | S | 1 | 0.00 | na | na |
|  | 17 | 5 | IE | 5 | 7.91 | na | 73.79 |
|  | 17 | 5 | S | 1 | 0.00 | na | 73.79 |
|  | 19 | 5 | S | 1 | 9.30 | 100.00 | 77.86 |
|  | 19 | 5 | IE | 5 | 5.58 | na | 77.86 |
|  | 23 | 6 | S | 1 | 19.44 | 60.00 | 67.97 |
|  | 23 | 6 | IA | 3 | 12.96 | 26.67 | 67.97 |
|  | 23 | 6 | IE | 5 | 13.33 | 36.88 | 67.97 |
|  | 24 | 6 | IA | 3 | 13.79 | na | 81.60 |
|  | 24 | 6 | S | 1 | 0.00 | na | 81.60 |
|  | 24 | 6 | IE | 5 | 6.44 | na | 81.60 |
|  | 27 | 6 | S | 1 | 13.79 | 33.30 | 66.24 |
|  | 27 | 6 | IE | 5 | 4.83 | na | 66.24 |
|  | 27 | 6 | IA | 3 | 20.69 | 44.43 | 66.24 |
|  | 28 | 6 | IE | 5 | 6.20 | na | 84.56 |
|  | 28 | 6 | S | 1 | 16.90 | 60.00 | 84.56 |
|  | 28 | 6 | IA | 3 | 28.17 | 70.80 | 84.56 |
