# Supplementary material for: Asymmetric reproductive interference: The consequences of cross‐pollination on reproductive success in sexual–apomictic populations of Potentilla puberula (Rosaceae)
Source: Ecol Evol. 2017 Nov 28;8(1):365–81. doi: 10.1002/ece3.3684 (PMC5756837; doi:10.1002/ece3.3684)
Supplement: Supplementary file 2 [file ECE3-8-365-s002.docx]

**Online Resource 2** Descriptive statistics of the flow cytometric measurement of 1804 seeds from five ploidy cytotypes of *Potentilla puberula*. Seeds were obtained in two *ex situ* crossing experiments performed to infer the relation of the genomic constitution of the endosperm to reproductive success (experiment performed in 2012) and to provide additional data on the reproductive mode of maternal plants (experiment performed in 2013). For each “pollen recipient” and “pollen donor” used in the crosses the “individual” number (the first two digits signify the population) and generative “ploidy x” is provided. “Treatment” differentiates between homoploid crosses (*IA*), selfings (*S*), and heteroploid crosses (*IE*). “Standard” is the internal biological standard (*P* = *Pisum sativum* cv. Kleine Rheinländerin: Greilhuber & Ebert, 1994, *L* = *Lathyrus tuberosus*). “Count”, “mean” and “cv” are the number of particles registered, the mean fluorescence, and the variation coefficient, respectively, calculated for the internal “standard”, the “embryo”, and the “endosperm”. The “peak index” (i.e. endosperm : embryo ratio) was calculated from the respective fluorescence means. The “reproductive mode” of the seed is provided as *S* = regular sexuality, the combination of female meiosis and the zygotic origin of the embryo, *A* = apomixis the combination of female apomeiosis and parthenogenesis, and the minor modes *AF* = female apomeiosis and fertilization of the egg cell, *HP* = haploid parthenogenesis, apomixis involving contribution of one (*A1*) and three (*A3*) polar nuclei to the endosperm, and *AS* = apomixis preceded by a somatic duplication of the maternal genome in the embryo sac. No further statistics are provided for rare reproductive modes. The “paternal genomic contribution” is the number of genomes contributed to the endosperm calculated as *x* and *p*, *p* is expressed as the multiple of the holoploid maternal genome (*n*).

| crossing experiment | pollen recipient | | pollen  donor | | treatment | standard | standard | | | embryo | | | endosperm | | | peak index | reproductive mode | embtyo ploidy [*x*] | paternal genomic contribution | paternal genomic contribution *p* [*n*] |
| --- | --- | --- | --- | --- | --- | --- | --- | --- | --- | --- | --- | --- | --- | --- | --- | --- | --- | --- | --- | --- |
|  | individual | ploidy [*x*] | individual | ploidy [*x*] |  |  | count | mean | cv | count | mean | cv | count | mean | cv |  |  |  |  |  |
| 2012 | 09_01 | 5 | 09_08 | 4 | IE | P | 403 | 609.94 | 2.05 | 1775 | 107.81 | 4.81 | 140 | 287.68 | 4.54 | 0.18 | A | 5.00 | 3.34 | 1.34 |
| 2012 | 09_01 | 5 | 09_16 | 4 | IE | P | 412 | 627.91 | 2.70 | 1789 | 113.62 | 4.55 | 125 | 317.93 | 3.14 | 0.18 | A | 5.00 | 3.99 | 1.60 |
| 2012 | 09_01 | 5 | 09_16 | 4 | IE | P | 710 | 597.16 | 2.51 | 1392 | 106.03 | 4.81 | 113 | 300.89 | 4.19 | 0.18 | A | 5.00 | 4.19 | 1.68 |
| 2012 | 09_01 | 5 | 09_16 | 4 | IE | P | 422 | 600.98 | 2.42 | 1702 | 106.83 | 4.82 | 163 | 299.80 | 3.51 | 0.18 | A | 5.00 | 4.03 | 1.61 |
| 2012 | 09_01 | 5 | 09_16 | 4 | IE | P | 567 | 606.90 | 2.07 | 1669 | 108.48 | 5.26 | 126 | 308.12 | 4.01 | 0.18 | A | 5.00 | 4.20 | 1.68 |
| 2012 | 09_01 | 5 | 09_16 | 4 | IE | P | 326 | 600.26 | 2.47 | 1497 | 108.01 | 4.91 | 112 | 266.34 | 4.53 | 0.18 | A | 5.00 | 2.33 | 0.93 |
| 2012 | 09_01 | 5 | 09_16 | 4 | IE | P | 547 | 600.72 | 2.29 | 1414 | 105.75 | 5.18 | 116 | 259.03 | 4.92 | 0.18 | A | 5.00 | 2.25 | 0.90 |
| 2012 | 09_01 | 5 | 09_16 | 4 | IE | P | 423 | 600.09 | 2.29 | 1689 | 103.63 | 4.98 | 116 | 249.83 | 3.80 | 0.17 | A | 5.00 | 2.05 | 0.82 |
| 2012 | 09_01 | 5 | 09_16 | 4 | IE | P | 483 | 604.73 | 2.10 | 1593 | 108.09 | 5.39 | 108 | 268.45 | 4.17 | 0.18 | A | 5.00 | 2.42 | 0.97 |
| 2012 | 09_01 | 5 | 09_16 | 4 | IE | P | 546 | 591.86 | 2.67 | 1668 | 104.09 | 5.38 | 96 | 260.24 | 4.74 | 0.18 | A | 5.00 | 2.50 | 1.00 |
| 2012 | 09_01 | 5 | 09_16 | 4 | IE | P | 335 | 590.29 | 2.27 | 1400 | 106.12 | 5.75 | 130 | 293.25 | 4.48 | 0.18 | A | 5.00 | 3.82 | 1.53 |
| 2012 | 09_01 | 5 | 09_46 | 5 | IA | P | 261 | 605.41 | 2.19 | 1948 | 109.01 | 4.87 | 136 | 326.95 | 3.13 | 0.18 | A | 5.00 | 5.00 | 2.00 |
| 2012 | 09_01 | 5 | 09_47 | 4 | IE | P | 323 | 603.74 | 2.47 | 2028 | 107.61 | 3.94 | 131 | 296.74 | 3.00 | 0.18 | A | 5.00 | 3.79 | 1.52 |
| 2012 | 09_01 | 5 | 09_47 | 4 | IE | P | 469 | 594.61 | 2.40 | 1888 | 104.05 | 4.82 | 81 | 292.23 | 3.27 | 0.17 | A | 5.00 | 4.04 | 1.62 |
| 2012 | 09_01 | 5 | 09_47 | 4 | IE | P | 450 | 601.33 | 2.00 | 423 | 104.34 | 4.25 | 125 | 274.29 | 4.25 | 0.17 | A | 5.00 | 3.14 | 1.26 |
| 2012 | 09_01 | 5 | 09_47 | 4 | IE | P | 453 | 603.17 | 2.60 | 1616 | 106.10 | 4.88 | 97 | 296.45 | 3.95 | 0.18 | A | 5.00 | 3.97 | 1.59 |
| 2012 | 09_01 | 5 | 09_47 | 4 | IE | P | 358 | 605.89 | 2.80 | 1650 | 106.15 | 5.11 | 121 | 295.03 | 4.72 | 0.18 | A | 5.00 | 3.90 | 1.56 |
| 2012 | 09_01 | 5 | 09_47 | 4 | IE | P | 323 | 593.11 | 2.17 | 1642 | 103.92 | 4.56 | 130 | 289.28 | 3.68 | 0.18 | A | 5.00 | 3.92 | 1.57 |
| 2012 | 09_01 | 5 | 09_47 | 4 | IE | P | 486 | 603.96 | 2.02 | 1579 | 106.23 | 4.81 | 123 | 294.14 | 4.43 | 0.18 | A | 5.00 | 3.84 | 1.54 |
| 2012 | 09_01 | 5 | 09_47 | 4 | IE | P | 262 | 600.86 | 2.05 | 1824 | 106.42 | 4.78 | 91 | 295.09 | 3.31 | 0.18 | A | 5.00 | 3.86 | 1.55 |
| 2012 | 09_01 | 5 | 09_47 | 4 | IE | P | 559 | 604.25 | 2.44 | 1488 | 108.06 | 5.28 | 93 | 263.42 | 4.82 | 0.18 | A | 5.00 | 2.19 | 0.88 |
| 2012 | 09_01 | 5 | 09_47 | 4 | IE | P | 463 | 611.82 | 1.97 | 1791 | 106.92 | 5.17 | 142 | 298.69 | 3.76 | 0.17 | A | 5.00 | 3.97 | 1.59 |
| 2012 | 09_05 | 4 | 09_01 | 5 | IE | P | 235 | 596.36 | 2.21 | 2063 | 93.54 | 5.47 | 151 | 144.54 | 4.29 | 0.16 | S | 4.31 | 1.96 | 0.98 |
| 2012 | 09_05 | 4 | 09_05 | 4 | S | P | 225 | 606.12 | 1.97 | 1949 | 84.39 | 5.64 | 204 | 130.62 | 4.71 | 0.14 | S | 3.83 | 1.73 | 0.87 |
| 2012 | 09_05 | 4 | 09_12 | 5 | IE | P | 153 | 596.36 | 1.54 | 1695 | 89.51 | 5.97 | 199 | 134.54 | 5.22 | 0.15 | S | 4.13 | 2.05 | 1.03 |
| 2012 | 09_05 | 4 | 09_31 | 4 | IA | P | 190 | 606.60 | 1.93 | 2048 | 92.23 | 5.13 | 140 | 143.22 | 3.68 | 0.15 | S | 4.18 | 1.87 | 0.93 |
| 2012 | 09_05 | 4 | 09_31 | 4 | IA | P | 184 | 601.57 | 1.92 | 2176 | 87.43 | 5.68 | 196 | 133.83 | 3.97 | 0.15 | S | 4.00 | 1.88 | 0.94 |
| 2012 | 09_05 | 4 | 09_31 | 4 | IA | P | 220 | 602.50 | 1.44 | 2126 | 86.49 | 5.76 | 159 | 131.69 | 4.39 | 0.14 | S | 3.95 | 1.88 | 0.94 |
| 2012 | 09_05 | 4 | 09_31 | 4 | IA | P | 176 | 603.05 | 1.96 | 2007 | 88.74 | 5.25 | 201 | 137.56 | 4.49 | 0.15 | S | 4.05 | 1.82 | 0.91 |
| 2012 | 09_05 | 4 | 09_31 | 4 | IA | P | 166 | 595.18 | 1.83 | 2232 | 85.93 | 5.82 | 160 | 133.11 | 3.86 | 0.14 | S | 3.97 | 1.79 | 0.90 |
| 2012 | 09_05 | 4 | 09_31 | 4 | IA | P | 99 | 598.87 | 2.24 | 2190 | 87.64 | 5.62 | 206 | 133.27 | 4.80 | 0.15 | S | 4.02 | 1.93 | 0.96 |
| 2012 | 09_05 | 4 | 09_31 | 4 | IA | P | 327 | 603.49 | 3.10 | 1969 | 85.21 | 7.45 | 140 | 127.86 | 3.87 | 0.14 | S | 3.88 | 1.94 | 0.97 |
| 2012 | 09_05 | 4 | 09_31 | 4 | IA | P | 157 | 595.26 | 1.91 | 2254 | 85.98 | 5.53 | 180 | 130.32 | 4.51 | 0.14 | S | 3.97 | 1.92 | 0.96 |
| 2012 | 09_05 | 4 | 09_31 | 4 | IA | P | 135 | 597.13 | 1.78 | 2128 | 86.79 | 5.33 | 161 | 132.60 | 4.50 | 0.15 | S | 4.00 | 1.89 | 0.94 |
| 2012 | 09_05 | 4 | 09_37 | 5 | IE | P | 270 | 607.57 | 1.72 | 2047 | 89.46 | 5.92 | 164 | 136.70 | 4.38 | 0.15 | S | 4.05 | 1.91 | 0.96 |
| 2012 | 09_05 | 4 | 09_47 | 4 | IA | P | 239 | 605.21 | 2.01 | 2120 | 88.67 | 5.35 | 156 | 137.14 | 4.51 | 0.15 | S | 4.03 | 1.83 | 0.91 |
| 2012 | 09_05 | 4 | 09_47 | 4 | IA | P | 161 | 603.76 | 2.48 | 2091 | 88.32 | 5.87 | 162 | 137.84 | 4.34 | 0.15 | S | 4.02 | 1.77 | 0.88 |
| 2012 | 09_05 | 4 | 09_47 | 4 | IA | P | 157 | 593.43 | 2.16 | 1983 | 84.41 | 5.87 | 204 | 137.28 | 5.43 | 0.14 | S | 3.91 | 1.46 | 0.73 |
| 2012 | 09_05 | 4 | 09_47 | 4 | IA | P | 171 | 598.94 | 1.93 | 2087 | 86.31 | 6.03 | 202 | 140.35 | 5.06 | 0.14 | S | 3.96 | 1.48 | 0.74 |
| 2012 | 09_05 | 4 | 09_47 | 4 | IA | P | 188 | 603.25 | 2.37 | 1667 | 88.06 | 5.18 | 152 | 136.16 | 4.30 | 0.15 | S | 4.01 | 1.82 | 0.91 |
| 2012 | 09_05 | 4 | 09_47 | 4 | IA | P | 224 | 605.40 | 2.00 | 2174 | 87.44 | 5.05 | 154 | 137.15 | 4.80 | 0.14 | S | 3.97 | 1.71 | 0.86 |
| 2012 | 09_05 | 4 | 09_47 | 4 | IA | P | 200 | 607.28 | 2.19 | 1943 | 90.55 | 5.19 | 175 | 140.69 | 3.63 | 0.15 | S | 4.10 | 1.83 | 0.91 |
| 2012 | 09_05 | 4 | 09_47 | 4 | IA | P | 184 | 603.40 | 2.03 | 1622 | 87.11 | 5.60 | 207 | 133.42 | 4.43 | 0.14 | S | 3.97 | 1.86 | 0.93 |
| 2012 | 09_05 | 4 | 09_47 | 4 | IA | P | 145 | 602.63 | 2.27 | 2113 | 88.81 | 5.55 | 188 | 139.57 | 4.50 | 0.15 | S | 4.05 | 1.74 | 0.87 |
| 2012 | 09_05 | 4 | 09_47 | 4 | IA | P | 107 | 596.67 | 2.17 | 1978 | 87.91 | 6.07 | 129 | 138.51 | 4.46 | 0.15 | S | 4.05 | 1.72 | 0.86 |
| 2012 | 09_05 | 4 | 09_48 | 5 | IE | P | 175 | 614.25 | 2.69 | 2180 | 95.75 | 5.12 | 194 | 142.23 | 3.82 | 0.16 | S | 4.29 | 2.21 | 1.10 |
| 2013 | 09_05 | 4 | 14_24 | 8 | IE | P | 560 | 611.01 | 2.12 | 438 | 113.68 | 3.61 | 53 | 161.04 | 3.61 | 0.186 | S | 5.12 |  |  |
| 2013 | 09_05 | 4 | 14_24 | 8 | IE | P | 946 | 622.40 | 2.10 | 901 | 115.12 | 3.66 | 116 | 161.09 | 3.43 | 0.185 | S | 5.09 |  |  |
| 2013 | 09_05 | 4 | 14_24 | 8 | IE | P | 773 | 641.36 | 2.33 | 1196 | 139.18 | 3.74 | 111 | 185.60 | 2.55 | 0.217 | S | 5.97 |  |  |
| 2013 | 09_05 | 4 | 14_24 | 8 | IE | P | 303 | 624.15 | 1.78 | 198 | 129.92 | 3.44 | 45 | 173.51 | 2.57 | 0.208 | S | 5.72 |  |  |
| 2013 | 09_05 | 4 | 27_04 | 7 | IE | P | 524 | 679.20 | 2.82 | 993 | 119.19 | 3.74 | 63 | 171.50 | 3.68 | 0.175 | S | 4.83 |  |  |
| 2013 | 09_05 | 4 | 27_04 | 7 | IE | P | 342 | 674.12 | 2.72 | 728 | 98.86 | 3.24 | 101 | 149.05 | 3.25 | 0.147 | S | 4.03 |  |  |
| 2013 | 09_05 | 4 | 27_04 | 7 | IE | P | 479 | 666.09 | 2.31 | 693 | 130.19 | 3.34 | 75 | 183.55 | 4.30 | 0.195 | S | 5.37 |  |  |
| 2013 | 09_05 | 4 | 27_04 | 7 | IE | P | 529 | 677.75 | 2.64 | 637 | 128.88 | 3.64 | 87 | 180.20 | 3.39 | 0.190 | S | 5.23 |  |  |
| 2012 | 09_08 | 4 | 09_08 | 4 | S | P | 130 | 603.00 | 1.94 | 2085 | 92.81 | 5.39 | 199 | 137.06 | 4.04 | 0.15 | S | 4.23 | 2.21 | 1.11 |
| 2012 | 09_08 | 4 | 09_16 | 4 | IA | P | 232 | 607.55 | 2.44 | 2218 | 88.35 | 5.53 | 165 | 135.23 | 3.81 | 0.15 | S | 4.00 | 1.88 | 0.94 |
| 2012 | 09_08 | 4 | 09_16 | 4 | IA | P | 288 | 607.92 | 1.99 | 2033 | 86.32 | 5.52 | 187 | 132.60 | 4.32 | 0.14 | S | 3.90 | 1.81 | 0.91 |
| 2012 | 09_08 | 4 | 09_16 | 4 | IA | P | 213 | 614.41 | 2.20 | 2218 | 86.14 | 5.64 | 179 | 133.73 | 4.04 | 0.14 | S | 3.85 | 1.73 | 0.86 |
| 2012 | 09_08 | 4 | 09_31 | 4 | IA | P | 365 | 619.21 | 3.20 | 1907 | 93.89 | 5.58 | 142 | 141.76 | 3.98 | 0.15 | S | 4.17 | 2.04 | 1.02 |
| 2012 | 09_08 | 4 | 09_31 | 4 | IA | P | 241 | 612.61 | 2.52 | 2000 | 88.71 | 5.87 | 161 | 133.72 | 4.81 | 0.14 | S | 3.98 | 1.96 | 0.98 |
| 2012 | 09_08 | 4 | 09_37 | 5 | IE | P | 237 | 606.71 | 2.09 | 1780 | 87.21 | 5.51 | 185 | 132.13 | 3.81 | 0.14 | S | 3.95 | 1.92 | 0.96 |
| 2012 | 09_08 | 4 | 09_46 | 5 | IE | P | 352 | 599.58 | 2.07 | 1718 | 102.48 | 5.00 | 218 | 145.82 | 3.80 | 0.17 | S | 4.70 | 2.71 | 1.36 |
| 2012 | 09_08 | 4 | 09_46 | 5 | IE | P | 290 | 598.90 | 2.59 | 1857 | 100.85 | 5.04 | 196 | 146.52 | 4.78 | 0.17 | S | 4.63 | 2.53 | 1.27 |
| 2013 | 09_08 | 4 | 17_16 | 8 | IE | P | 475 | 623.57 | 1.95 | 1028 | 125.64 | 3.54 | 139 | 175.52 | 3.68 | 0.201 | S | 5.54 |  |  |
| 2013 | 09_08 | 4 | 17_16 | 8 | IE | P | 174 | 614.96 | 1.81 | 476 | 125.41 | 3.31 | 62 | 171.43 | 3.40 | 0.204 | S | 5.61 |  |  |
| 2013 | 09_08 | 4 | 17_16 | 8 | IE | P | 556 | 622.00 | 1.87 | 1303 | 127.08 | 3.36 | 149 | 174.97 | 3.17 | 0.204 | S | 5.62 |  |  |
| 2013 | 09_08 | 4 | 27_02 | 7 | IE | P | 308 | 628.21 | 1.74 | 937 | 122.75 | 3.41 | 151 | 172.28 | 3.38 | 0.195 | S | 5.37 |  |  |
| 2013 | 09_08 | 4 | 27_02 | 7 | IE | P | 228 | 623.81 | 1.95 | 1531 | 108.36 | 3.36 | 93 | 155.35 | 3.34 | 0.174 | S | 4.78 |  |  |
| 2013 | 09_08 | 4 | 27_02 | 7 | IE | P | 438 | 616.69 | 2.01 | 1368 | 114.52 | 3.57 | 157 | 162.21 | 3.31 | 0.186 | S | 5.11 |  |  |
| 2013 | 09_08 | 4 | 27_02 | 7 | IE | P | 467 | 623.73 | 1.83 | 1826 | 111.88 | 3.17 | 114 | 160.84 | 4.02 | 0.179 | S | 4.93 |  |  |
| 2013 | 09_08 | 4 | 37_14 | 6 | IE | P | 775 | 627.60 | 2.19 | 1344 | 107.80 | 3.80 | 106 | 155.39 | 2.87 | 0.172 | S | 4.72 |  |  |
| 2013 | 09_08 | 4 | 37_14 | 6 | IE | P | 742 | 631.29 | 2.05 | 1019 | 109.33 | 3.22 | 111 | 159.03 | 2.57 | 0.173 | S | 4.76 |  |  |
| 2013 | 09_08 | 4 | 37_14 | 6 | IE | P | 998 | 630.07 | 1.80 | 981 | 104.24 | 3.08 | 95 | 150.89 | 3.10 | 0.165 | S | 4.55 |  |  |
| 2013 | 09_08 | 4 | 37_14 | 6 | IE | P | 880 | 631.55 | 1.67 | 1291 | 109.36 | 3.60 | 117 | 156.92 | 3.57 | 0.173 | S | 4.76 |  |  |
| 2012 | 09_12 | 5 | 09_05 | 4 | IE | P | 353 | 603.43 | 1.86 | 1798 | 107.76 | 5.49 | 143 | 293.74 | 3.59 | 0.18 | A | 5.00 | 3.63 | 1.45 |
| 2012 | 09_12 | 5 | 09_08 | 4 | IE | P | 357 | 601.85 | 1.97 | 2025 | 106.13 | 5.21 | 125 | 296.47 | 4.29 | 0.18 | A | 5.00 | 3.97 | 1.59 |
| 2012 | 09_12 | 5 | 09_12 | 5 | S | P | 238 | 606.38 | 2.82 | 2184 | 106.92 | 6.05 | 133 | 315.93 | 3.80 | 0.18 | A | 5.00 | 4.77 | 1.91 |
| 2012 | 09_12 | 5 | 09_12 | 5 | S | P | 385 | 598.75 | 2.45 | 2088 | 106.31 | 5.75 | 172 | 326.75 | 3.90 | 0.18 | A | 5.00 | 5.37 | 2.15 |
| 2012 | 09_12 | 5 | 09_31 | 4 | IE | P | 445 | 593.29 | 2.54 | 1892 | 104.05 | 6.03 | 103 | 293.27 | 4.76 | 0.18 | A | 5.00 | 4.09 | 1.64 |
| 2012 | 09_12 | 5 | 09_37 | 5 | IA | P | 187 | 600.86 | 2.14 | 2260 | 103.95 | 5.74 | 146 | 284.67 | 3.98 | 0.17 | A | 5.00 | 3.69 | 1.48 |
| 2012 | 09_12 | 5 | 09_47 | 4 | IE | P | 365 | 597.76 | 2.11 | 2049 | 105.74 | 5.43 | 150 | 297.66 | 4.20 | 0.18 | A | 5.00 | 4.08 | 1.63 |
| 2012 | 09_16 | 4 | 09_31 | 4 | IA | P | 229 | 612.05 | 2.40 | 2147 | 89.37 | 5.62 | 191 | 136.44 | 4.56 | 0.15 | S | 4.01 | 1.90 | 0.95 |
| 2012 | 09_16 | 4 | 09_37 | 5 | IE | P | 227 | 596.03 | 2.17 | 2183 | 85.76 | 5.16 | 197 | 128.24 | 4.87 | 0.14 | S | 3.96 | 2.00 | 1.00 |
| 2012 | 09_16 | 4 | 09_47 | 4 | IA | P | 107 | 609.92 | 2.03 | 2358 | 86.66 | 5.75 | 187 | 133.37 | 4.22 | 0.14 | S | 3.91 | 1.80 | 0.90 |
| 2012 | 09_16 | 4 | 09_47 | 4 | IA | P | 209 | 597.71 | 2.27 | 1899 | 83.76 | 5.54 | 165 | 128.11 | 4.16 | 0.14 | S | 3.85 | 1.81 | 0.91 |
| 2012 | 09_16 | 4 | 09_47 | 4 | IA | P | 309 | 603.07 | 1.82 | 2056 | 85.73 | 5.75 | 183 | 131.40 | 4.51 | 0.14 | S | 3.91 | 1.83 | 0.91 |
| 2012 | 09_16 | 4 | 09_48 | 5 | IE | P | 300 | 600.16 | 1.96 | 1729 | 92.29 | 5.60 | 232 | 137.01 | 4.67 | 0.15 | S | 4.23 | 2.18 | 1.09 |
| 2013 | 09_16 | 4 | 17_19 | 8 | IE | P | 654 | 608.55 | 3.77 | 625 | 133.43 | 3.81 | 97 | 176.56 | 3.81 | 0.219 | S | 6.03 |  |  |
| 2013 | 09_16 | 4 | 17_19 | 8 | IE | P | 327 | 597.05 | 2.76 | 316 | 129.60 | 3.88 | 59 | 175.57 | 3.90 | 0.217 | S | 5.97 |  |  |
| 2013 | 09_16 | 4 | 17_19 | 8 | IE | P | 486 | 590.37 | 3.53 | 500 | 112.90 | 4.15 | 65 | 159.25 | 4.37 | 0.191 | S | 5.26 |  |  |
| 2013 | 09_16 | 4 | 17_19 | 8 | IE | P | 592 | 587.68 | 3.07 | 632 | 123.39 | 3.86 | 68 | 167.19 | 4.14 | 0.210 | S | 5.77 |  |  |
| 2013 | 09_16 | 4 | 17_19 | 8 | IE | P | 759 | 605.62 | 2.99 | 872 | 119.29 | 3.30 | 86 | 166.89 | 4.64 | 0.197 | S | 5.42 |  |  |
| 2013 | 09_16 | 4 | 37_05 | 6 | IE | P | 424 | 579.84 | 1.87 | 1074 | 105.10 | 3.44 | 128 | 153.06 | 4.19 | 0.181 | S | 4.98 |  |  |
| 2013 | 09_16 | 4 | 37_05 | 6 | IE | P | 499 | 595.91 | 1.70 | 1688 | 105.21 | 3.61 | 174 | 152.24 | 3.77 | 0.177 | S | 4.85 |  |  |
| 2013 | 09_16 | 4 | 37_05 | 6 | IE | P | 369 | 594.07 | 1.84 | 1231 | 105.40 | 3.44 | 99 | 151.85 | 3.41 | 0.177 | S | 4.88 |  |  |
| 2012 | 09_31 | 4 | 09_01 | 5 | IE | P | 626 | 594.27 | 2.22 | 1316 | 84.95 | 5.36 | 138 | 129.61 | 4.22 | 0.14 | S | 3.93 | 1.86 | 0.93 |
| 2012 | 09_31 | 4 | 09_01 | 5 | IE | P | 498 | 593.54 | 2.13 | 916 | 89.59 | 5.04 | 156 | 133.94 | 5.59 | 0.15 | S | 4.15 | 2.10 | 1.05 |
| 2012 | 09_31 | 4 | 09_01 | 5 | IE | P | 557 | 604.28 | 2.24 | 1107 | 88.32 | 4.45 | 135 | 132.58 | 4.07 | 0.15 | S | 4.02 | 2.00 | 1.00 |
| 2012 | 09_31 | 4 | 09_01 | 5 | IE | P | 908 | 594.62 | 2.81 | 1317 | 84.06 | 5.17 | 153 | 129.10 | 4.94 | 0.14 | S | 3.89 | 1.80 | 0.90 |
| 2012 | 09_31 | 4 | 09_01 | 5 | IE | P | 940 | 587.73 | 1.98 | 1079 | 88.74 | 5.22 | 137 | 132.60 | 4.14 | 0.15 | S | 4.15 | 2.10 | 1.05 |
| 2012 | 09_31 | 4 | 09_01 | 5 | IE | P | 697 | 595.17 | 2.14 | 1201 | 101.15 | 4.49 | 168 | 147.73 | 4.88 | 0.17 | S | 4.67 | 2.52 | 1.26 |
| 2012 | 09_31 | 4 | 09_01 | 5 | IE | P | 847 | 589.85 | 2.10 | 1111 | 93.93 | 4.99 | 117 | 139.89 | 3.88 | 0.16 | S | 4.38 | 2.24 | 1.12 |
| 2012 | 09_31 | 4 | 09_01 | 5 | IE | P | 885 | 603.78 | 2.60 | 1365 | 111.81 | 5.75 | 213 | 161.35 | 5.03 | 0.19 | S | 5.09 | 2.84 | 1.42 |
| 2012 | 09_31 | 4 | 09_01 | 5 | IE | P | 517 | 591.58 | 3.19 | 1451 | 94.54 | 6.77 | 168 | 144.91 | 4.89 | 0.16 | S | 4.39 | 2.05 | 1.03 |
| 2012 | 09_31 | 4 | 09_01 | 5 | IE | P | 855 | 591.66 | 2.72 | 1121 | 105.44 | 5.07 | 137 | 155.89 | 3.99 | 0.18 | S | 4.90 | 2.56 | 1.28 |
| 2012 | 09_31 | 4 | 09_08 | 4 | IA | P | 265 | 595.97 | 2.31 | 2092 | 83.63 | 6.35 | 171 | 128.75 | 4.76 | 0.14 | S | 3.86 | 1.78 | 0.89 |
| 2012 | 09_31 | 4 | 09_12 | 5 | IE | P | 614 | 607.47 | 2.40 | 1431 | 93.78 | 4.98 | 137 | 140.99 | 4.45 | 0.15 | S | 4.24 | 2.11 | 1.05 |
| 2012 | 09_31 | 4 | 09_12 | 5 | IE | P | 586 | 599.42 | 3.11 | 1196 | 98.55 | 5.72 | 135 | 140.51 | 3.39 | 0.16 | S | 4.52 | 2.60 | 1.30 |
| 2012 | 09_31 | 4 | 09_12 | 5 | IE | P | 595 | 608.45 | 2.34 | 1571 | 90.32 | 5.87 | 141 | 136.47 | 4.88 | 0.15 | S | 4.08 | 2.00 | 1.00 |
| 2012 | 09_31 | 4 | 09_12 | 5 | IE | P | 454 | 613.07 | 2.13 | 1809 | 93.14 | 5.14 | 186 | 142.62 | 4.43 | 0.15 | S | 4.18 | 1.96 | 0.98 |
| 2012 | 09_31 | 4 | 09_12 | 5 | IE | P | 588 | 607.66 | 2.37 | 1493 | 93.08 | 5.16 | 177 | 140.57 | 4.37 | 0.15 | S | 4.21 | 2.06 | 1.03 |
| 2012 | 09_31 | 4 | 09_12 | 5 | IE | P | 568 | 616.95 | 2.25 | 1467 | 106.45 | 5.05 | 135 | 157.73 | 3.52 | 0.17 | S | 4.74 | 2.46 | 1.23 |
| 2012 | 09_31 | 4 | 09_12 | 5 | IE | P | 758 | 604.78 | 2.29 | 1214 | 109.09 | 5.38 | 153 | 157.01 | 3.72 | 0.18 | S | 4.96 | 2.78 | 1.39 |
| 2012 | 09_31 | 4 | 09_12 | 5 | IE | P | 480 | 606.40 | 2.24 | 1265 | 108.86 | 5.75 | 222 | 156.43 | 4.13 | 0.18 | S | 4.94 | 2.78 | 1.39 |
| 2012 | 09_31 | 4 | 09_12 | 5 | IE | P | 853 | 606.22 | 2.40 | 1258 | 102.43 | 5.94 | 156 | 149.36 | 3.78 | 0.17 | S | 4.65 | 2.52 | 1.26 |
| 2012 | 09_31 | 4 | 09_12 | 5 | IE | P | 739 | 608.76 | 2.66 | 1218 | 93.55 | 5.92 | 142 | 145.57 | 4.13 | 0.15 | S | 4.23 | 1.88 | 0.94 |
| 2012 | 09_31 | 4 | 09_31 | 4 | S | P | 281 | 605.75 | 2.55 | 1311 | 138.17 | 5.22 | 271 | 183.67 | 3.88 | 0.23 | S | 6.27 | 4.21 | 2.10 |
| 2012 | 09_31 | 4 | 09_31 | 4 | S | P | 378 | 608.45 | 1.96 | 1376 | 132.73 | 4.82 | 260 | 175.03 | 3.63 | 0.22 | S | 6.00 | 4.09 | 2.04 |
| 2012 | 09_31 | 4 | 09_31 | 4 | S | P | 230 | 610.14 | 1.99 | 1501 | 170.33 | 4.00 | 135 | 260.75 | 3.03 | 0.28 | AF | 7.68 |  |  |
| 2012 | 09_31 | 4 | 09_31 | 4 | S | P | 297 | 611.32 | 1.91 | 1447 | 131.92 | 5.04 | 237 | 178.91 | 4.65 | 0.22 | S | 5.93 | 3.82 | 1.91 |
| 2012 | 09_31 | 4 | 09_31 | 4 | S | P | 358 | 614.42 | 1.63 | 1585 | 131.66 | 4.78 | 208 | 178.91 | 3.71 | 0.21 | S | 5.89 | 3.78 | 1.89 |
| 2012 | 09_31 | 4 | 09_31 | 4 | S | P | 214 | 609.95 | 1.90 | 2247 | 86.91 | 5.43 | 226 | 130.58 | 5.06 | 0.14 | S | 3.92 | 1.95 | 0.97 |
| 2012 | 09_31 | 4 | 09_47 | 4 | IA | P | 287 | 596.98 | 2.36 | 1929 | 86.76 | 5.47 | 180 | 133.85 | 4.52 | 0.15 | S | 4.00 | 1.83 | 0.91 |
| 2013 | 09_31 | 4 | 14_26 | 8 | IE | P | 651 | 525.81 | 3.45 | 1111 | 101.89 | 3.76 | 109 | 142.95 | 3.72 | 0.194 | S | 5.33 |  |  |
| 2013 | 09_31 | 4 | 14_26 | 8 | IE | P | 612 | 533.40 | 2.64 | 906 | 75.81 | 3.64 | 92 | 116.90 | 3.08 | 0.142 | S | 3.91 |  |  |
| 2013 | 09_31 | 4 | 14_26 | 8 | IE | P | 970 | 531.74 | 2.65 | 1512 | 74.15 | 3.95 | 113 | 113.93 | 3.95 | 0.139 | S | 3.83 |  |  |
| 2013 | 09_31 | 4 | 17_29 | 7 | IE | P | 651 | 551.68 | 3.02 | 669 | 106.67 | 3.59 | 131 | 150.21 | 3.44 | 0.193 | S | 5.32 |  |  |
| 2013 | 09_31 | 4 | 17_29 | 7 | IE | P | 648 | 546.23 | 2.76 | 866 | 101.37 | 3.29 | 73 | 141.54 | 3.40 | 0.186 | S | 5.10 |  |  |
| 2013 | 09_31 | 4 | 17_29 | 7 | IE | P | 600 | 532.19 | 2.98 | 918 | 100.67 | 3.52 | 110 | 139.08 | 3.23 | 0.189 | S | 5.20 |  |  |
| 2013 | 09_31 | 4 | 41_30 | 6 | IE | P | 802 | 585.08 | 3.38 | 1525 | 101.75 | 3.97 | 129 | 146.38 | 3.79 | 0.174 | S | 4.78 |  |  |
| 2013 | 09_31 | 4 | 41_30 | 6 | IE | P | 550 | 571.93 | 2.83 | 879 | 97.97 | 3.49 | 85 | 141.71 | 4.13 | 0.171 | S | 4.71 |  |  |
| 2013 | 09_31 | 4 | 41_30 | 6 | IE | P | 648 | 564.49 | 3.12 | 1114 | 101.20 | 3.88 | 73 | 147.55 | 4.37 | 0.179 | S | 4.93 |  |  |
| 2013 | 09_31 | 4 | 41_30 | 6 | IE | P | 541 | 556.44 | 2.91 | 745 | 98.00 | 3.71 | 99 | 140.95 | 4.90 | 0.176 | S | 4.84 |  |  |
| 2012 | 09_37 | 5 | 09_08 | 4 | IE | P | 341 | 606.44 | 2.03 | 2023 | 108.07 | 6.13 | 119 | 299.30 | 3.89 | 0.18 | A | 5.00 | 3.85 | 1.54 |
| 2012 | 09_37 | 5 | 09_37 | 5 | S | P | 290 | 598.66 | 2.52 | 1705 | 103.87 | 6.14 | 91 | 263.90 | 3.81 | 0.17 | A | 5.00 | 2.70 | 1.08 |
| 2012 | 09_37 | 5 | 09_37 | 5 | S | P | 271 | 592.37 | 2.60 | 1680 | 103.78 | 5.40 | 77 | 265.67 | 4.00 | 0.18 | A | 5.00 | 2.80 | 1.12 |
| 2012 | 09_37 | 5 | 09_46 | 5 | IA | P | 487 | 595.97 | 2.44 | 1690 | 105.37 | 5.96 | 153 | 301.65 | 5.01 | 0.18 | A | 5.00 | 4.31 | 1.73 |
| 2012 | 09_37 | 5 | 09_46 | 5 | IA | P | 379 | 594.55 | 2.04 | 1579 | 104.57 | 5.87 | 144 | 299.53 | 3.87 | 0.18 | A | 5.00 | 4.32 | 1.73 |
| 2012 | 09_37 | 5 | 09_47 | 4 | IE | P | 364 | 602.32 | 2.10 | 1906 | 106.07 | 5.46 | 104 | 260.24 | 4.29 | 0.18 | A | 5.00 | 2.27 | 0.91 |
| 2012 | 09_37 | 5 | 09_47 | 4 | IE | P | 348 | 607.32 | 1.75 | 1957 | 106.34 | 5.43 | 151 | 300.08 | 4.32 | 0.18 | A | 5.00 | 4.11 | 1.64 |
| 2012 | 09_46 | 5 | 09_05 | 4 | IE | P | 549 | 606.82 | 2.37 | 1628 | 151.51 | 4.83 | 95 | 262.21 | 4.80 | 0.25 | AF | 6.93 |  |  |
| 2012 | 09_46 | 5 | 09_12 | 5 | IA | P | 323 | 610.41 | 2.61 | 1950 | 153.62 | 4.77 | 163 | 268.59 | 5.55 | 0.25 | AF | 6.99 |  |  |
| 2012 | 09_47 | 4 | 09_01 | 5 | IE | P | 173 | 598.44 | 2.01 | 1895 | 103.52 | 5.29 | 176 | 148.33 | 3.80 | 0.17 | S | 4.76 | 2.70 | 1.35 |
| 2012 | 09_47 | 4 | 09_05 | 4 | IA | P | 205 | 604.50 | 1.62 | 1958 | 85.53 | 6.06 | 226 | 130.90 | 4.54 | 0.14 | S | 3.89 | 1.83 | 0.91 |
| 2012 | 09_47 | 4 | 09_16 | 4 | IA | P | 161 | 613.40 | 2.71 | 2231 | 89.18 | 6.11 | 198 | 135.89 | 4.61 | 0.15 | S | 4.00 | 1.90 | 0.95 |
| 2012 | 09_47 | 4 | 09_16 | 4 | IA | P | 167 | 594.85 | 2.45 | 1890 | 83.00 | 6.41 | 209 | 125.56 | 5.00 | 0.14 | S | 3.84 | 1.87 | 0.93 |
| 2012 | 09_47 | 4 | 09_16 | 4 | IA | P | 180 | 595.64 | 2.83 | 1929 | 85.22 | 6.92 | 213 | 131.50 | 5.08 | 0.14 | S | 3.93 | 1.80 | 0.90 |
| 2012 | 09_47 | 4 | 09_37 | 5 | IE | P | 169 | 594.77 | 2.42 | 2480 | 86.33 | 6.03 | 260 | 131.04 | 4.73 | 0.15 | S | 3.99 | 1.92 | 0.96 |
| 2012 | 09_47 | 4 | 09_47 | 4 | S | P | 197 | 612.89 | 2.97 | 1661 | 95.66 | 6.43 | 261 | 142.31 | 4.62 | 0.16 | S | 4.29 | 2.20 | 1.10 |
| 2012 | 09_48 | 5 | 09_12 | 5 | IA | P | 205 | 605.49 | 3.10 | 2086 | 110.79 | 5.82 | 128 | 342.76 | 4.27 | 0.18 | A | 5.00 | 5.47 | 2.19 |
| 2012 | 09_48 | 5 | 09_12 | 5 | IA | P | 192 | 594.12 | 2.53 | 2129 | 105.05 | 5.23 | 139 | 328.61 | 4.31 | 0.18 | A | 5.00 | 5.64 | 2.26 |
| 2012 | 09_48 | 5 | 09_12 | 5 | IA | P | 206 | 596.13 | 2.22 | 2111 | 107.16 | 5.45 | 142 | 313.77 | 6.10 | 0.18 | A | 5.00 | 4.64 | 1.86 |
| 2012 | 09_48 | 5 | 09_12 | 5 | IA | P | 213 | 597.74 | 2.60 | 1989 | 107.19 | 5.10 | 134 | 329.76 | 4.43 | 0.18 | A | 5.00 | 5.38 | 2.15 |
| 2012 | 09_48 | 5 | 09_12 | 5 | IA | P | 205 | 598.68 | 2.23 | 1696 | 107.90 | 5.68 | 104 | 318.35 | 4.55 | 0.18 | A | 5.00 | 4.75 | 1.90 |
| 2012 | 09_48 | 5 | 09_12 | 5 | IA | P | 228 | 605.64 | 2.23 | 1894 | 108.81 | 5.38 | 138 | 326.28 | 4.32 | 0.18 | A | 5.00 | 4.99 | 2.00 |
| 2012 | 09_48 | 5 | 09_12 | 5 | IA | P | 197 | 599.36 | 2.68 | 1926 | 106.72 | 5.10 | 104 | 275.12 | 3.94 | 0.18 | A | 5.00 | 2.89 | 1.16 |
| 2012 | 09_48 | 5 | 09_12 | 5 | IA | P | 238 | 605.44 | 2.41 | 1945 | 109.94 | 5.15 | 102 | 278.77 | 3.58 | 0.18 | A | 5.00 | 2.68 | 1.07 |
| 2012 | 09_48 | 5 | 09_12 | 5 | IA | P | 167 | 590.45 | 2.53 | 1263 | 106.84 | 5.55 | 77 | 308.55 | 3.76 | 0.18 | A | 5.00 | 4.44 | 1.78 |
| 2012 | 09_48 | 5 | 09_12 | 5 | IA | P | 218 | 598.74 | 2.55 | 2025 | 107.92 | 5.23 | 116 | 310.93 | 4.14 | 0.18 | A | 5.00 | 4.41 | 1.76 |
| 2012 | 09_48 | 5 | 09_16 | 4 | IE | P | 342 | 599.27 | 2.39 | 1944 | 106.21 | 5.40 | 135 | 294.28 | 3.58 | 0.18 | A | 5.00 | 3.85 | 1.54 |
| 2012 | 09_48 | 5 | 09_37 | 5 | IA | P | 227 | 596.30 | 3.73 | 2407 | 104.65 | 5.13 | 86 | 311.11 | 4.32 | 0.18 | A | 5.00 | 4.86 | 1.95 |
| 2012 | 09_48 | 5 | 09_37 | 5 | IA | P | 235 | 589.83 | 2.23 | 1965 | 107.41 | 5.10 | 143 | 275.60 | 3.76 | 0.18 | A | 5.00 | 2.83 | 1.13 |
| 2012 | 09_48 | 5 | 09_37 | 5 | IA | P | 262 | 592.00 | 2.54 | 2099 | 109.21 | 6.11 | 147 | 292.09 | 4.74 | 0.18 | A | 5.00 | 3.37 | 1.35 |
| 2012 | 09_48 | 5 | 09_37 | 5 | IA | P | 459 | 598.92 | 2.21 | 1835 | 110.76 | 5.24 | 134 | 337.85 | 4.99 | 0.18 | A | 5.00 | 5.25 | 2.10 |
| 2012 | 09_48 | 5 | 09_37 | 5 | IA | P | 243 | 600.20 | 2.52 | 1950 | 111.59 | 5.27 | 140 | 345.57 | 5.12 | 0.19 | A | 5.00 | 5.48 | 2.19 |
| 2012 | 09_48 | 5 | 09_37 | 5 | IA | P | 226 | 586.61 | 1.97 | 2062 | 106.30 | 5.24 | 95 | 265.59 | 3.49 | 0.18 | A | 5.00 | 2.49 | 1.00 |
| 2012 | 09_48 | 5 | 09_37 | 5 | IA | P | 142 | 586.35 | 2.07 | 2312 | 106.44 | 5.00 | 147 | 318.27 | 3.84 | 0.18 | A | 5.00 | 4.95 | 1.98 |
| 2012 | 09_48 | 5 | 09_37 | 5 | IA | P | 181 | 588.88 | 1.98 | 2302 | 107.74 | 5.87 | 88 | 285.86 | 3.82 | 0.18 | A | 5.00 | 3.27 | 1.31 |
| 2012 | 09_48 | 5 | 09_37 | 5 | IA | P | 214 | 593.52 | 2.43 | 2183 | 110.95 | 5.66 | 83 | 283.25 | 4.99 | 0.19 | A | 5.00 | 2.76 | 1.11 |
| 2012 | 09_48 | 5 | 09_37 | 5 | IA | P | 182 | 597.31 | 1.95 | 2034 | 108.67 | 4.97 | 89 | 329.13 | 4.15 | 0.18 | A | 5.00 | 5.14 | 2.06 |
| 2012 | 09_48 | 5 | 09_47 | 4 | IE | P | 407 | 600.08 | 2.38 | 1948 | 106.58 | 5.12 | 145 | 297.60 | 3.87 | 0.18 | A | 5.00 | 3.96 | 1.58 |
| 2012 | 10_01 | 4 | 10_01 | 4 | S | P | 273 | 604.65 | 2.03 | 1851 | 87.30 | 6.90 | 164 | 130.78 | 4.91 | 0.14 | S | 3.97 | 1.99 | 1.00 |
| 2012 | 10_01 | 4 | 10_01 | 4 | S | P | 284 | 608.99 | 2.37 | 1929 | 87.99 | 6.44 | 193 | 132.84 | 4.95 | 0.14 | S | 3.97 | 1.95 | 0.97 |
| 2012 | 10_01 | 4 | 10_01 | 4 | S | P | 333 | 604.84 | 2.45 | 2033 | 86.82 | 6.91 | 222 | 134.36 | 4.35 | 0.14 | S | 3.95 | 1.79 | 0.89 |
| 2012 | 10_01 | 4 | 10_01 | 4 | S | P | 237 | 603.94 | 3.67 | 2092 | 94.68 | 6.18 | 185 | 145.02 | 5.06 | 0.16 | S | 4.31 | 2.02 | 1.01 |
| 2012 | 10_01 | 4 | 10_01 | 4 | S | P | 313 | 597.96 | 2.24 | 1903 | 85.89 | 6.48 | 187 | 130.69 | 4.73 | 0.14 | S | 3.95 | 1.89 | 0.94 |
| 2012 | 10_01 | 4 | 10_01 | 4 | S | P | 285 | 602.75 | 2.00 | 1752 | 87.54 | 7.34 | 240 | 133.30 | 5.08 | 0.15 | S | 3.99 | 1.91 | 0.95 |
| 2012 | 10_01 | 4 | 10_17 | 4 | IA | P | 142 | 593.92 | 2.17 | 2235 | 83.72 | 6.00 | 155 | 127.06 | 5.64 | 0.14 | S | 3.88 | 1.87 | 0.93 |
| 2012 | 10_01 | 4 | 10_17 | 4 | IA | P | 123 | 593.78 | 2.64 | 2156 | 79.84 | 6.09 | 129 | 127.46 | 3.52 | 0.13 | S | 3.70 | 1.49 | 0.75 |
| 2012 | 10_01 | 4 | 10_26 | 7 | IE | P | 426 | 616.47 | 2.65 | 1665 | 120.56 | 6.14 | 204 | 168.45 | 5.04 | 0.20 | S | 5.38 | 3.24 | 1.62 |
| 2012 | 10_01 | 4 | 10_32 | 7 | IE | P | 345 | 609.09 | 2.17 | 1838 | 112.51 | 4.88 | 207 | 157.68 | 4.54 | 0.18 | S | 5.08 | 3.04 | 1.52 |
| 2012 | 10_01 | 4 | 10_32 | 7 | IE | P | 235 | 599.66 | 2.88 | 1720 | 109.79 | 5.52 | 185 | 152.84 | 4.12 | 0.18 | S | 5.03 | 3.06 | 1.53 |
| 2012 | 10_01 | 4 | 10_33 | 5 | IE | P | 153 | 596.42 | 2.49 | 2292 | 88.37 | 5.94 | 165 | 131.59 | 4.80 | 0.15 | S | 4.07 | 2.08 | 1.04 |
| 2013 | 10_01 | 4 | 14_14 | 7 | IE | P | 204 | 593.47 | 1.72 | 618 | 113.32 | 3.16 | 54 | 158.20 | 3.33 | 0.191 | S | 5.25 |  |  |
| 2013 | 10_01 | 4 | 14_14 | 7 | IE | P | 411 | 594.27 | 1.68 | 1015 | 122.10 | 3.25 | 60 | 168.69 | 3.28 | 0.205 | S | 5.65 |  |  |
| 2013 | 10_01 | 4 | 14_14 | 7 | IE | P | 282 | 596.18 | 1.63 | 667 | 115.99 | 3.11 | 49 | 162.71 | 3.68 | 0.195 | S | 5.35 |  |  |
| 2013 | 10_01 | 4 | 15_23 | 6 | IE | P | 201 | 588.24 | 1.52 | 597 | 102.68 | 2.73 | 65 | 146.23 | 3.07 | 0.175 | S | 4.80 |  |  |
| 2013 | 10_01 | 4 | 15_23 | 6 | IE | P | 375 | 583.46 | 1.65 | 819 | 102.15 | 2.77 | 66 | 146.65 | 2.94 | 0.175 | S | 4.81 |  |  |
| 2013 | 10_01 | 4 | 17_14 | 8 | IE | P | 452 | 593.49 | 1.98 | 601 | 122.44 | 3.10 | 85 | 164.58 | 3.53 | 0.206 | S | 5.67 |  |  |
| 2013 | 10_01 | 4 | 17_14 | 8 | IE | P | 802 | 594.49 | 1.66 | 918 | 115.59 | 3.36 | 96 | 156.94 | 3.12 | 0.194 | S | 5.35 |  |  |
| 2013 | 10_01 | 4 | 17_14 | 8 | IE | P | 518 | 584.01 | 1.80 | 476 | 116.08 | 3.02 | 53 | 160.23 | 3.14 | 0.199 | S | 5.47 |  |  |
| 2013 | 10_01 | 4 | 17_14 | 8 | IE | P | 635 | 585.10 | 1.44 | 690 | 112.91 | 2.79 | 55 | 153.68 | 2.27 | 0.193 | S | 5.31 |  |  |
| 2012 | 10_03 | 5 | 10_17 | 4 | IE | P | 262 | 600.96 | 2.10 | 2239 | 105.42 | 4.51 | 96 | 299.70 | 3.90 | 0.18 | A | 5.00 | 4.21 | 1.69 |
| 2012 | 10_03 | 5 | 10_17 | 4 | IE | P | 234 | 591.79 | 1.95 | 2189 | 103.08 | 4.87 | 68 | 262.78 | 3.99 | 0.17 | A | 5.00 | 2.75 | 1.10 |
| 2012 | 10_03 | 5 | 10_17 | 4 | IE | P | 205 | 593.58 | 2.34 | 2422 | 104.56 | 4.89 | 101 | 293.27 | 3.89 | 0.18 | A | 5.00 | 4.02 | 1.61 |
| 2012 | 10_03 | 5 | 10_17 | 4 | IE | P | 319 | 587.64 | 2.73 | 2125 | 91.18 | 5.16 | 178 | 141.54 | 4.10 | 0.16 | S | 4.31 | 1.93 | 0.77 |
| 2012 | 10_03 | 5 | 10_17 | 4 | IE | P | 221 | 599.75 | 2.67 | 2430 | 106.79 | 4.69 | 114 | 300.02 | 3.54 | 0.18 | A | 5.00 | 4.05 | 1.62 |
| 2012 | 10_03 | 5 | 10_17 | 4 | IE | P | 250 | 598.00 | 2.03 | 2183 | 105.35 | 4.65 | 127 | 299.04 | 3.25 | 0.18 | A | 5.00 | 4.19 | 1.68 |
| 2012 | 10_03 | 5 | 10_17 | 4 | IE | P | 255 | 596.15 | 2.58 | 2466 | 105.79 | 4.76 | 85 | 396.46 | 3.87 | 0.18 | A | 5.00 | 8.74 | 3.50 |
| 2012 | 10_03 | 5 | 10_17 | 4 | IE | P | 218 | 587.39 | 2.45 | 2441 | 103.48 | 4.81 | 123 | 287.02 | 6.04 | 0.18 | A | 5.00 | 3.87 | 1.55 |
| 2012 | 10_03 | 5 | 10_17 | 4 | IE | P | 334 | 594.41 | 2.60 | 2005 | 87.02 | 5.54 | 177 | 133.89 | 4.14 | 0.15 | S | 4.06 | 1.88 | 0.75 |
| 2012 | 10_03 | 5 | 10_17 | 4 | IE | P | 346 | 600.10 | 2.06 | 1914 | 205.02 | 3.73 | 54 | 286.98 | 4.65 | 0.34 | AF | 9.48 |  |  |
| 2012 | 10_06 | 5 | 10_01 | 4 | IE | P | 105 | 599.34 | 1.76 | 2419 | 108.27 | 5.61 | 108 | 293.91 | 2.86 | 0.18 | A | 5.00 | 3.57 | 1.43 |
| 2012 | 10_06 | 5 | 10_17 | 4 | IE | P | 144 | 593.37 | 2.33 | 2159 | 105.25 | 5.71 | 108 | 294.39 | 3.46 | 0.18 | A | 5.00 | 3.99 | 1.59 |
| 2012 | 10_08 | 4 | 10_01 | 4 | IA | P | 132 | 602.92 | 1.94 | 2082 | 88.64 | 6.18 | 174 | 135.98 | 4.84 | 0.15 | S | 4.04 | 1.88 | 0.94 |
| 2012 | 10_08 | 4 | 10_06 | 5 | IE | P | 86 | 601.38 | 1.95 | 1622 | 91.89 | 7.70 | 210 | 133.69 | 5.39 | 0.15 | S | 4.20 | 2.29 | 1.15 |
| 2012 | 10_08 | 4 | 10_17 | 4 | IA | P | 146 | 595.33 | 2.00 | 2303 | 88.84 | 6.16 | 139 | 133.78 | 5.30 | 0.15 | S | 4.10 | 2.03 | 1.01 |
| 2012 | 10_08 | 4 | 10_17 | 4 | IA | P | 145 | 591.19 | 2.07 | 2007 | 87.19 | 6.45 | 178 | 134.73 | 4.35 | 0.15 | S | 4.06 | 1.84 | 0.92 |
| 2012 | 10_08 | 4 | 10_32 | 7 | IE | P | 118 | 616.23 | 2.65 | 1233 | 129.29 | 5.79 | 532 | 170.73 | 5.19 | 0.21 | S | 5.77 | 3.92 | 1.96 |
| 2012 | 10_08 | 4 | 10_32 | 7 | IE | P | 237 | 601.47 | 2.50 | 2193 | 117.22 | 5.61 | 254 | 161.74 | 5.00 | 0.19 | S | 5.36 | 3.32 | 1.66 |
| 2013 | 10_08 | 4 | 17_15 | 8 | IE | P | 459 | 588.57 | 1.79 | 747 | 102.55 | 3.21 | 92 | 146.51 | 3.41 | 0.174 | S | 4.79 |  |  |
| 2013 | 10_08 | 4 | 17_15 | 8 | IE | P | 354 | 586.54 | 1.95 | 418 | 122.72 | 3.34 | 76 | 168.39 | 3.42 | 0.209 | S | 5.75 |  |  |
| 2013 | 10_08 | 4 | 17_15 | 8 | IE | P | 268 | 584.36 | 1.76 | 733 | 112.54 | 3.12 | 46 | 160.20 | 2.67 | 0.193 | S | 5.30 |  |  |
| 2013 | 10_08 | 4 | 17_15 | 8 | IE | P | 240 | 576.62 | 1.95 | 473 | 113.05 | 2.95 | 48 | 154.82 | 2.72 | 0.196 | S | 5.39 |  |  |
| 2013 | 10_08 | 4 | 17_15 | 8 | IE | P | 422 | 628.80 | 2.17 | 1032 | 130.37 | 3.35 | 118 | 175.29 | 3.12 | 0.207 | S | 5.70 |  |  |
| 2013 | 10_08 | 4 | 17_15 | 8 | IE | P | 337 | 625.39 | 1.88 | 1340 | 119.74 | 3.75 | 134 | 168.08 | 3.72 | 0.191 | S | 5.26 |  |  |
| 2013 | 10_08 | 4 | 17_15 | 8 | IE | P | 503 | 605.91 | 1.88 | 1181 | 124.05 | 3.48 | 140 | 166.87 | 3.96 | 0.205 | S | 5.63 |  |  |
| 2012 | 10_17 | 4 | 10_01 | 4 | IA | P | 152 | 598.61 | 2.35 | 1222 | 86.87 | 6.48 | 143 | 134.48 | 4.27 | 0.15 | S | 3.99 | 1.80 | 0.90 |
| 2012 | 10_17 | 4 | 10_01 | 4 | IA | P | 380 | 605.53 | 2.95 | 1892 | 89.20 | 5.95 | 180 | 138.95 | 5.76 | 0.15 | S | 4.05 | 1.79 | 0.90 |
| 2012 | 10_17 | 4 | 10_01 | 4 | IA | P | 256 | 604.28 | 2.48 | 2128 | 88.44 | 5.47 | 166 | 134.19 | 4.78 | 0.15 | S | 4.02 | 1.94 | 0.97 |
| 2012 | 10_17 | 4 | 10_01 | 4 | IA | P | 311 | 608.88 | 2.99 | 2085 | 90.13 | 5.61 | 194 | 138.78 | 5.06 | 0.15 | S | 4.07 | 1.87 | 0.94 |
| 2012 | 10_17 | 4 | 10_01 | 4 | IA | P | 289 | 597.31 | 2.49 | 2139 | 85.91 | 5.67 | 149 | 133.42 | 5.27 | 0.14 | S | 3.95 | 1.77 | 0.88 |
| 2012 | 10_17 | 4 | 10_01 | 4 | IA | P | 307 | 601.78 | 2.65 | 1919 | 87.85 | 5.69 | 149 | 135.57 | 5.02 | 0.15 | S | 4.01 | 1.83 | 0.92 |
| 2012 | 10_17 | 4 | 10_01 | 4 | IA | P | 302 | 603.26 | 2.49 | 2088 | 88.32 | 5.97 | 194 | 135.26 | 5.45 | 0.15 | S | 4.03 | 1.89 | 0.94 |
| 2012 | 10_17 | 4 | 10_01 | 4 | IA | P | 163 | 601.79 | 2.58 | 1301 | 89.89 | 5.68 | 135 | 141.73 | 4.75 | 0.15 | S | 4.11 | 1.74 | 0.87 |
| 2012 | 10_17 | 4 | 10_01 | 4 | IA | P | 274 | 601.21 | 2.64 | 2212 | 87.64 | 5.74 | 142 | 135.18 | 4.38 | 0.15 | S | 4.01 | 1.83 | 0.92 |
| 2012 | 10_17 | 4 | 10_01 | 4 | IA | P | 341 | 599.94 | 2.66 | 2100 | 87.09 | 5.90 | 173 | 135.45 | 4.84 | 0.15 | S | 3.99 | 1.78 | 0.89 |
| 2012 | 10_17 | 4 | 10_08 | 4 | IA | P | 394 | 603.36 | 2.83 | 1787 | 89.02 | 6.15 | 167 | 133.97 | 4.68 | 0.15 | S | 4.06 | 2.01 | 1.00 |
| 2012 | 10_17 | 4 | 10_08 | 4 | IA | P | 416 | 588.24 | 2.48 | 1583 | 86.97 | 5.58 | 176 | 134.49 | 5.41 | 0.15 | S | 4.07 | 1.84 | 0.92 |
| 2012 | 10_17 | 4 | 10_08 | 4 | IA | P | 358 | 590.77 | 2.36 | 1867 | 87.53 | 6.29 | 163 | 133.41 | 3.53 | 0.15 | S | 4.07 | 1.94 | 0.97 |
| 2012 | 10_17 | 4 | 10_08 | 4 | IA | P | 310 | 602.81 | 2.40 | 1806 | 88.56 | 5.29 | 212 | 136.83 | 4.84 | 0.15 | S | 4.04 | 1.84 | 0.92 |
| 2012 | 10_17 | 4 | 10_08 | 4 | IA | P | 348 | 597.59 | 2.51 | 2015 | 87.51 | 5.79 | 169 | 133.36 | 4.95 | 0.15 | S | 4.03 | 1.92 | 0.96 |
| 2012 | 10_17 | 4 | 10_08 | 4 | IA | P | 260 | 591.32 | 3.01 | 2217 | 88.76 | 5.92 | 223 | 136.00 | 4.68 | 0.15 | S | 4.13 | 1.93 | 0.97 |
| 2012 | 10_17 | 4 | 10_08 | 4 | IA | P | 306 | 608.88 | 2.79 | 2032 | 89.76 | 5.79 | 205 | 137.34 | 4.92 | 0.15 | S | 4.05 | 1.90 | 0.95 |
| 2012 | 10_17 | 4 | 10_08 | 4 | IA | P | 348 | 596.82 | 2.50 | 1772 | 84.79 | 5.63 | 168 | 136.98 | 5.08 | 0.14 | S | 3.91 | 1.50 | 0.75 |
| 2012 | 10_17 | 4 | 10_08 | 4 | IA | P | 421 | 599.61 | 2.36 | 1727 | 88.97 | 6.09 | 199 | 134.92 | 4.61 | 0.15 | S | 4.08 | 1.97 | 0.99 |
| 2012 | 10_17 | 4 | 10_08 | 4 | IA | P | 250 | 595.70 | 2.29 | 1432 | 87.13 | 6.04 | 148 | 132.27 | 4.96 | 0.15 | S | 4.02 | 1.94 | 0.97 |
| 2012 | 10_17 | 4 | 10_26 | 7 | IE | P | 301 | 591.35 | 2.20 | 1703 | 113.49 | 4.86 | 200 | 157.21 | 3.78 | 0.19 | S | 5.28 | 3.24 | 1.62 |
| 2012 | 10_17 | 4 | 10_33 | 5 | IE | P | 121 | 594.07 | 2.28 | 1833 | 96.80 | 5.95 | 209 | 140.06 | 4.98 | 0.16 | S | 4.48 | 2.48 | 1.24 |
| 2013 | 10_17 | 4 | 17_17 | 8 | IE | P | 340 | 591.66 | 1.95 | 619 | 131.41 | 3.53 | 80 | 177.44 | 3.24 | 0.222 | S | 6.11 |  |  |
| 2013 | 10_17 | 4 | 17_17 | 8 | IE | P | 489 | 590.10 | 2.14 | 556 | 115.12 | 2.76 | 73 | 159.22 | 3.49 | 0.195 | S | 5.36 |  |  |
| 2013 | 10_17 | 4 | 17_17 | 8 | IE | P | 405 | 585.46 | 1.94 | 806 | 115.28 | 3.41 | 39 | 168.63 | 3.38 | 0.197 | S | 5.41 |  |  |
| 2013 | 10_17 | 4 | 17_17 | 8 | IE | P | 416 | 584.41 | 1.94 | 483 | 119.89 | 3.07 | 52 | 164.64 | 3.65 | 0.205 | S | 5.64 |  |  |
| 2013 | 10_17 | 4 | 18_01 | 6 | IE | P | 288 | 595.88 | 4.37 | 998 | 111.64 | 4.28 | 101 | 157.61 | 4.28 | 0.187 | S | 5.15 |  |  |
| 2013 | 10_17 | 4 | 18_01 | 6 | IE | P | 311 | 600.12 | 3.70 | 1070 | 109.75 | 3.58 | 94 | 159.66 | 3.51 | 0.183 | S | 5.03 |  |  |
| 2013 | 10_17 | 4 | 18_01 | 6 | IE | P | 267 | 599.85 | 4.71 | 933 | 112.78 | 3.70 | 62 | 161.79 | 3.71 | 0.188 | S | 5.17 |  |  |
| 2013 | 10_17 | 4 | 18_01 | 6 | IE | P | 273 | 595.28 | 3.75 | 886 | 112.53 | 3.18 | 65 | 161.62 | 2.93 | 0.189 | S | 5.20 |  |  |
| 2012 | 10_22 | 4 | 10_01 | 4 | IA | P | 172 | 598.09 | 1.98 | 2198 | 86.55 | 5.64 | 167 | 132.20 | 4.33 | 0.14 | S | 3.98 | 1.88 | 0.94 |
| 2012 | 10_22 | 4 | 10_01 | 4 | IA | P | 149 | 598.30 | 2.11 | 2261 | 85.80 | 5.73 | 169 | 130.35 | 4.66 | 0.14 | S | 3.94 | 1.90 | 0.95 |
| 2012 | 10_22 | 4 | 10_08 | 4 | IA | P | 233 | 599.49 | 2.58 | 2022 | 86.92 | 6.38 | 171 | 129.80 | 4.61 | 0.14 | S | 3.99 | 2.02 | 1.01 |
| 2012 | 10_22 | 4 | 10_22 | 4 | S | P | 65 | 608.29 | 2.88 | 2081 | 88.85 | 7.12 | 265 | 135.30 | 5.07 | 0.15 | S | 4.02 | 1.92 | 0.96 |
| 2012 | 10_22 | 4 | 10_26 | 7 | IE | P | 237 | 602.67 | 2.37 | 1807 | 122.12 | 5.10 | 225 | 168.50 | 4.22 | 0.20 | S | 5.57 | 3.46 | 1.73 |
| 2012 | 10_22 | 4 | 10_26 | 7 | IE | P | 448 | 600.19 | 2.20 | 2002 | 116.70 | 5.12 | 234 | 162.75 | 3.88 | 0.19 | S | 5.35 | 3.24 | 1.62 |
| 2012 | 10_22 | 4 | 10_26 | 7 | IE | P | 132 | 598.93 | 2.58 | 1005 | 132.35 | 5.52 | 136 | 185.92 | 3.88 | 0.22 | S | 6.08 | 3.62 | 1.81 |
| 2012 | 10_22 | 4 | 10_26 | 7 | IE | P | 198 | 597.39 | 2.33 | 2004 | 114.24 | 5.18 | 222 | 160.79 | 5.57 | 0.19 | S | 5.26 | 3.12 | 1.56 |
| 2012 | 10_22 | 4 | 10_26 | 7 | IE | P | 302 | 593.73 | 2.38 | 1578 | 120.23 | 6.06 | 329 | 170.34 | 5.09 | 0.20 | S | 5.57 | 3.25 | 1.62 |
| 2012 | 10_22 | 4 | 10_26 | 7 | IE | P | 453 | 602.83 | 2.25 | 1328 | 130.93 | 5.03 | 248 | 175.96 | 4.59 | 0.22 | S | 5.97 | 3.92 | 1.96 |
| 2012 | 10_22 | 4 | 10_26 | 7 | IE | P | 267 | 606.02 | 2.10 | 2027 | 116.11 | 5.49 | 232 | 161.98 | 4.34 | 0.19 | S | 5.27 | 3.19 | 1.59 |
| 2012 | 10_22 | 4 | 10_26 | 7 | IE | P | 124 | 603.78 | 2.06 | 1663 | 114.17 | 4.80 | 135 | 160.20 | 3.81 | 0.19 | S | 5.20 | 3.10 | 1.55 |
| 2012 | 10_22 | 4 | 10_26 | 7 | IE | P | 296 | 602.62 | 2.56 | 1753 | 120.12 | 4.98 | 212 | 168.21 | 4.66 | 0.20 | S | 5.48 | 3.29 | 1.64 |
| 2012 | 10_22 | 4 | 10_26 | 7 | IE | P | 263 | 607.31 | 1.99 | 1840 | 119.68 | 5.34 | 214 | 168.55 | 4.93 | 0.20 | S | 5.42 | 3.21 | 1.60 |
| 2012 | 10_22 | 4 | 10_28 | 7 | IE | P | 498 | 598.47 | 2.60 | 1276 | 124.03 | 4.91 | 248 | 164.99 | 5.56 | 0.21 | S | 5.70 | 3.82 | 1.91 |
| 2012 | 10_22 | 4 | 10_28 | 7 | IE | P | 285 | 591.72 | 2.45 | 1595 | 114.07 | 5.50 | 171 | 162.91 | 3.88 | 0.19 | S | 5.30 | 3.03 | 1.52 |
| 2012 | 10_22 | 4 | 10_28 | 7 | IE | P | 315 | 600.74 | 2.73 | 1440 | 121.95 | 7.96 | 255 | 201.05 | 4.65 | 0.20 | AF | 5.58 |  |  |
| 2012 | 10_22 | 4 | 10_28 | 7 | IE | P | 319 | 601.86 | 2.22 | 1590 | 115.02 | 4.85 | 202 | 164.12 | 4.06 | 0.19 | S | 5.25 | 3.01 | 1.51 |
| 2012 | 10_22 | 4 | 10_28 | 7 | IE | P | 265 | 601.95 | 2.32 | 1346 | 116.30 | 5.26 | 206 | 163.73 | 4.11 | 0.19 | S | 5.31 | 3.15 | 1.57 |
| 2012 | 10_22 | 4 | 10_28 | 7 | IE | P | 413 | 598.62 | 2.59 | 1814 | 111.50 | 5.72 | 155 | 154.62 | 3.65 | 0.19 | S | 5.12 | 3.14 | 1.57 |
| 2012 | 10_22 | 4 | 10_28 | 7 | IE | P | 332 | 600.39 | 2.79 | 1653 | 114.69 | 4.88 | 166 | 162.56 | 4.63 | 0.19 | S | 5.25 | 3.06 | 1.53 |
| 2012 | 10_22 | 4 | 10_28 | 7 | IE | P | 294 | 597.91 | 2.50 | 1496 | 109.78 | 6.43 | 246 | 150.46 | 4.52 | 0.18 | S | 5.05 | 3.18 | 1.59 |
| 2012 | 10_22 | 4 | 10_28 | 7 | IE | P | 406 | 602.88 | 2.56 | 1954 | 110.28 | 4.79 | 316 | 156.81 | 5.31 | 0.18 | S | 5.03 | 2.91 | 1.45 |
| 2012 | 10_22 | 4 | 10_28 | 7 | IE | P | 214 | 607.82 | 2.24 | 1163 | 116.83 | 5.89 | 216 | 162.43 | 4.08 | 0.19 | S | 5.28 | 3.22 | 1.61 |
| 2012 | 10_22 | 4 | 10_32 | 7 | IE | P | 382 | 607.85 | 2.75 | 1745 | 119.18 | 5.86 | 251 | 161.55 | 4.78 | 0.20 | S | 5.39 | 3.47 | 1.74 |
| 2012 | 10_22 | 4 | 10_33 | 5 | IE | P | 174 | 597.64 | 2.89 | 1709 | 109.50 | 5.59 | 258 | 152.37 | 3.86 | 0.18 | S | 5.04 | 3.07 | 1.53 |
| 2013 | 10_22 | 4 | 37_16 | 6 | IE | P | 324 | 624.07 | 4.07 | 1489 | 152.81 | 4.51 | 113 | 248.37 | 5.81 | 0.245 | S | 6.73 |  |  |
| 2013 | 10_22 | 4 | 37_16 | 6 | IE | P | 516 | 614.64 | 4.42 | 1064 | 113.09 | 3.96 | 154 | 158.73 | 3.29 | 0.184 | S | 5.06 |  |  |
| 2013 | 10_22 | 4 | 37_16 | 6 | IE | P | 487 | 623.95 | 4.47 | 984 | 114.93 | 3.85 | 105 | 163.56 | 3.25 | 0.184 | S | 5.06 |  |  |
| 2013 | 10_22 | 4 | 37_16 | 6 | IE | P | 785 | 605.48 | 4.62 | 861 | 113.54 | 4.10 | 133 | 160.01 | 4.61 | 0.188 | S | 5.16 |  |  |
| 2012 | 10_26 | 7 | 10_01 | 4 | IE | P | 489 | 605.98 | 2.78 | 1809 | 149.81 | 5.23 | 91 | 373.09 | 4.31 | 0.25 | A | 7.00 | 3.43 | 0.98 |
| 2012 | 10_26 | 7 | 10_01 | 4 | IE | P | 498 | 608.52 | 2.67 | 1773 | 150.94 | 4.94 | 109 | 381.17 | 4.95 | 0.25 | A | 7.00 | 3.68 | 1.05 |
| 2012 | 10_26 | 7 | 10_01 | 4 | IE | P | 442 | 600.52 | 2.86 | 1795 | 148.17 | 4.94 | 96 | 378.50 | 4.25 | 0.25 | A | 7.00 | 3.88 | 1.11 |
| 2012 | 10_26 | 7 | 10_01 | 4 | IE | P | 316 | 600.89 | 2.02 | 1510 | 145.95 | 4.64 | 68 | 375.59 | 2.73 | 0.24 | A | 7.00 | 4.01 | 1.15 |
| 2012 | 10_26 | 7 | 10_01 | 4 | IE | P | 466 | 608.72 | 2.59 | 1841 | 151.19 | 4.94 | 85 | 387.53 | 3.38 | 0.25 | A | 7.00 | 3.94 | 1.13 |
| 2012 | 10_26 | 7 | 10_01 | 4 | IE | P | 464 | 603.18 | 2.69 | 1551 | 147.76 | 4.68 | 101 | 383.25 | 5.58 | 0.24 | A | 7.00 | 4.16 | 1.19 |
| 2012 | 10_26 | 7 | 10_01 | 4 | IE | P | 428 | 602.85 | 2.10 | 1497 | 148.44 | 4.38 | 73 | 379.92 | 3.58 | 0.25 | A | 7.00 | 3.92 | 1.12 |
| 2012 | 10_26 | 7 | 10_01 | 4 | IE | P | 327 | 597.22 | 2.25 | 1427 | 148.70 | 4.97 | 106 | 385.14 | 3.56 | 0.25 | A | 7.00 | 4.13 | 1.18 |
| 2012 | 10_26 | 7 | 10_01 | 4 | IE | P | 156 | 611.59 | 1.84 | 699 | 151.82 | 4.28 | 30 | 392.95 | 2.60 | 0.25 | A | 7.00 | 4.12 | 1.18 |
| 2012 | 10_26 | 7 | 10_03 | 5 | IE | P | 184 | 593.57 | 2.34 | 2206 | 146.80 | 6.09 | 91 | 443.27 | 3.63 | 0.25 | A | 7.00 | 7.14 | 2.04 |
| 2012 | 10_26 | 7 | 10_03 | 5 | IE | P | 233 | 599.21 | 2.63 | 2016 | 148.95 | 5.78 | 89 | 420.83 | 3.75 | 0.25 | A | 7.00 | 5.78 | 1.65 |
| 2012 | 10_26 | 7 | 10_06 | 5 | IE | P | 390 | 602.87 | 2.90 | 1882 | 150.01 | 5.40 | 128 | 424.71 | 4.81 | 0.25 | A | 7.00 | 5.82 | 1.66 |
| 2012 | 10_26 | 7 | 10_06 | 5 | IE | P | 379 | 601.79 | 2.05 | 1716 | 148.95 | 5.09 | 107 | 415.73 | 3.66 | 0.25 | A | 7.00 | 5.54 | 1.58 |
| 2012 | 10_26 | 7 | 10_06 | 5 | IE | P | 415 | 603.71 | 2.08 | 1724 | 148.13 | 4.56 | 106 | 433.28 | 4.26 | 0.25 | A | 7.00 | 6.47 | 1.85 |
| 2012 | 10_26 | 7 | 10_06 | 5 | IE | P | 425 | 598.38 | 2.31 | 2139 | 146.27 | 4.85 | 156 | 432.70 | 4.63 | 0.24 | A | 7.00 | 6.71 | 1.92 |
| 2012 | 10_26 | 7 | 10_06 | 5 | IE | P | 347 | 597.29 | 2.19 | 1830 | 146.69 | 5.11 | 109 | 433.17 | 4.15 | 0.25 | A | 7.00 | 6.67 | 1.91 |
| 2012 | 10_26 | 7 | 10_06 | 5 | IE | P | 292 | 596.86 | 2.53 | 1961 | 146.56 | 5.00 | 105 | 430.75 | 3.35 | 0.25 | A | 7.00 | 6.57 | 1.88 |
| 2012 | 10_26 | 7 | 10_06 | 5 | IE | P | 436 | 599.12 | 2.08 | 1548 | 220.63 | 4.33 | 101 | 377.46 | 4.66 | 0.37 | AF | 10.40 |  |  |
| 2012 | 10_26 | 7 | 10_06 | 5 | IE | P | 344 | 601.84 | 2.40 | 1269 | 149.61 | 4.34 | 88 | 437.15 | 3.65 | 0.25 | A | 7.00 | 6.45 | 1.84 |
| 2012 | 10_26 | 7 | 10_06 | 5 | IE | P | 367 | 606.54 | 2.29 | 1755 | 149.34 | 4.48 | 147 | 229.89 | 4.43 | 0.25 | S | 6.95 | 3.20 | 0.92 |
| 2012 | 10_26 | 7 | 10_06 | 5 | IE | P | 292 | 604.86 | 2.23 | 1614 | 148.65 | 3.93 | 74 | 443.15 | 4.08 | 0.25 | A | 7.00 | 6.87 | 1.96 |
| 2012 | 10_26 | 7 | 10_08 | 4 | IE | P | 438 | 617.53 | 3.98 | 1654 | 155.44 | 4.98 | 79 | 391.23 | 4.30 | 0.25 | A | 7.00 | 3.62 | 1.03 |
| 2012 | 10_26 | 7 | 10_08 | 4 | IE | P | 371 | 617.87 | 4.97 | 1476 | 157.64 | 4.34 | 97 | 402.41 | 4.46 | 0.26 | A | 7.00 | 3.87 | 1.11 |
| 2012 | 10_26 | 7 | 10_08 | 4 | IE | P | 415 | 603.94 | 2.89 | 1875 | 151.06 | 4.76 | 87 | 393.66 | 5.11 | 0.25 | A | 7.00 | 4.24 | 1.21 |
| 2012 | 10_26 | 7 | 10_08 | 4 | IE | P | 396 | 615.70 | 3.27 | 1197 | 156.30 | 5.42 | 83 | 406.73 | 4.28 | 0.25 | A | 7.00 | 4.22 | 1.20 |
| 2012 | 10_26 | 7 | 10_08 | 4 | IE | P | 416 | 608.32 | 3.31 | 1763 | 152.60 | 4.44 | 98 | 390.72 | 3.93 | 0.25 | A | 7.00 | 3.92 | 1.12 |
| 2012 | 10_26 | 7 | 10_08 | 4 | IE | P | 863 | 590.57 | 4.06 | 1334 | 149.10 | 5.95 | 63 | 379.36 | 3.74 | 0.25 | A | 7.00 | 3.81 | 1.09 |
| 2012 | 10_26 | 7 | 10_08 | 4 | IE | P | 505 | 613.38 | 3.49 | 1360 | 153.50 | 3.66 | 98 | 391.77 | 4.04 | 0.25 | A | 7.00 | 3.87 | 1.10 |
| 2012 | 10_26 | 7 | 10_08 | 4 | IE | P | 525 | 589.17 | 3.54 | 1670 | 147.60 | 5.03 | 68 | 379.70 | 3.50 | 0.25 | A | 7.00 | 4.01 | 1.14 |
| 2012 | 10_26 | 7 | 10_08 | 4 | IE | P | 604 | 586.39 | 2.60 | 1457 | 147.61 | 5.04 | 105 | 380.66 | 4.75 | 0.25 | A | 7.00 | 4.05 | 1.16 |
| 2012 | 10_26 | 7 | 10_08 | 4 | IE | P | 410 | 601.01 | 3.21 | 1840 | 150.67 | 4.90 | 86 | 383.77 | 3.70 | 0.25 | A | 7.00 | 3.83 | 1.09 |
| 2012 | 10_26 | 7 | 10_22 | 4 | IE | P | 265 | 601.48 | 2.98 | 1634 | 150.15 | 4.60 | 84 | 387.63 | 3.30 | 0.25 | A | 7.00 | 4.07 | 1.16 |
| 2012 | 10_26 | 7 | 10_22 | 4 | IE | P | 296 | 604.13 | 2.38 | 1816 | 149.24 | 4.98 | 116 | 384.25 | 4.65 | 0.25 | A | 7.00 | 4.02 | 1.15 |
| 2012 | 10_26 | 7 | 10_22 | 4 | IE | P | 282 | 601.53 | 2.52 | 1882 | 151.22 | 5.75 | 107 | 340.91 | 4.80 | 0.25 | A | 7.00 | 1.78 | 0.51 |
| 2012 | 10_26 | 7 | 10_22 | 4 | IE | P | 167 | 596.82 | 2.49 | 2093 | 146.06 | 5.67 | 91 | 443.00 | 3.89 | 0.24 | A | 7.00 | 7.23 | 2.07 |
| 2012 | 10_26 | 7 | 10_22 | 4 | IE | P | 206 | 597.52 | 2.51 | 2252 | 147.08 | 5.09 | 99 | 427.84 | 4.54 | 0.25 | A | 7.00 | 6.36 | 1.82 |
| 2012 | 10_26 | 7 | 10_28 | 7 | IA | P | 351 | 605.50 | 2.52 | 2031 | 152.46 | 5.17 | 98 | 442.56 | 3.50 | 0.25 | A | 7.00 | 6.32 | 1.81 |
| 2012 | 10_26 | 7 | 10_28 | 7 | IA | P | 274 | 603.50 | 2.06 | 1749 | 148.40 | 4.21 | 62 | 433.85 | 2.48 | 0.25 | A | 7.00 | 6.46 | 1.85 |
| 2012 | 10_26 | 7 | 10_28 | 7 | IA | P | 326 | 608.34 | 2.57 | 1817 | 149.40 | 4.58 | 79 | 439.19 | 3.31 | 0.25 | A | 7.00 | 6.58 | 1.88 |
| 2012 | 10_26 | 7 | 10_28 | 7 | IA | P | 352 | 600.55 | 2.45 | 1886 | 150.12 | 4.71 | 106 | 439.32 | 3.87 | 0.25 | A | 7.00 | 6.49 | 1.85 |
| 2012 | 10_26 | 7 | 10_28 | 7 | IA | P | 247 | 604.06 | 2.54 | 1092 | 149.16 | 4.77 | 80 | 440.87 | 3.96 | 0.25 | A | 7.00 | 6.69 | 1.91 |
| 2012 | 10_26 | 7 | 10_28 | 7 | IA | P | 371 | 598.95 | 2.54 | 1910 | 146.91 | 4.06 | 77 | 511.14 | 2.46 | 0.25 | A | 7.00 | 10.35 | 2.96 |
| 2012 | 10_26 | 7 | 10_28 | 7 | IA | P | 217 | 601.84 | 2.32 | 1791 | 147.55 | 4.71 | 100 | 432.02 | 3.44 | 0.25 | A | 7.00 | 6.50 | 1.86 |
| 2012 | 10_26 | 7 | 10_28 | 7 | IA | P | 273 | 596.98 | 2.39 | 1781 | 148.02 | 4.84 | 113 | 446.32 | 4.34 | 0.25 | A | 7.00 | 7.11 | 2.03 |
| 2012 | 10_26 | 7 | 10_28 | 7 | IA | P | 295 | 602.70 | 2.25 | 1695 | 148.03 | 4.15 | 112 | 443.01 | 4.12 | 0.25 | A | 7.00 | 6.95 | 1.99 |
| 2012 | 10_26 | 7 | 10_28 | 7 | IA | P | 378 | 596.40 | 2.18 | 1729 | 146.58 | 5.35 | 67 | 406.92 | 3.82 | 0.25 | A | 7.00 | 5.43 | 1.55 |
| 2012 | 10_26 | 7 | 10_32 | 7 | IA | P | 339 | 596.56 | 2.32 | 1592 | 146.75 | 4.31 | 52 | 382.38 | 2.64 | 0.25 | A | 7.00 | 4.24 | 1.21 |
| 2012 | 10_26 | 7 | 10_32 | 7 | IA | P | 454 | 602.94 | 2.61 | 1643 | 148.81 | 4.78 | 100 | 460.61 | 4.05 | 0.25 | A | 7.00 | 7.67 | 2.19 |
| 2012 | 10_26 | 7 | 10_32 | 7 | IA | P | 324 | 589.36 | 2.75 | 1562 | 147.58 | 4.70 | 83 | 372.74 | 4.56 | 0.25 | A | 7.00 | 3.68 | 1.05 |
| 2012 | 10_26 | 7 | 10_32 | 7 | IA | P | 360 | 597.95 | 2.18 | 1789 | 148.51 | 4.83 | 108 | 444.76 | 4.74 | 0.25 | A | 7.00 | 6.96 | 1.99 |
| 2012 | 10_26 | 7 | 10_32 | 7 | IA | P | 305 | 602.73 | 2.11 | 1528 | 149.50 | 5.45 | 88 | 441.46 | 3.78 | 0.25 | A | 7.00 | 6.67 | 1.91 |
| 2012 | 10_26 | 7 | 10_32 | 7 | IA | P | 364 | 597.90 | 3.62 | 1821 | 149.84 | 4.92 | 86 | 446.92 | 3.58 | 0.25 | A | 7.00 | 6.88 | 1.97 |
| 2012 | 10_26 | 7 | 10_32 | 7 | IA | P | 282 | 601.20 | 2.10 | 1383 | 148.65 | 4.79 | 79 | 390.47 | 5.15 | 0.25 | A | 7.00 | 4.39 | 1.25 |
| 2012 | 10_26 | 7 | 10_32 | 7 | IA | P | 228 | 588.92 | 2.31 | 1717 | 146.76 | 5.62 | 108 | 353.47 | 4.33 | 0.25 | A | 7.00 | 2.86 | 0.82 |
| 2012 | 10_26 | 7 | 10_32 | 7 | IA | P | 515 | 603.19 | 1.95 | 1709 | 148.29 | 5.07 | 102 | 444.88 | 3.25 | 0.25 | A | 7.00 | 7.00 | 2.00 |
| 2012 | 10_26 | 7 | 10_32 | 7 | IA | P | 304 | 600.81 | 2.51 | 1812 | 148.75 | 5.24 | 102 | 430.80 | 3.52 | 0.25 | A | 7.00 | 6.27 | 1.79 |
| 2012 | 10_26 | 7 | 10_33 | 5 | IE | P | 263 | 589.45 | 2.64 | 2192 | 145.56 | 5.50 | 93 | 426.77 | 4.76 | 0.25 | A | 7.00 | 6.52 | 1.86 |
| 2012 | 10_26 | 7 | 10_41 | 5 | IE | P | 297 | 602.34 | 2.21 | 2081 | 145.86 | 4.75 | 92 | 433.03 | 4.51 | 0.24 | A | 7.00 | 6.78 | 1.94 |
| 2012 | 10_26 | 7 | 10_41 | 5 | IE | P | 306 | 603.07 | 2.73 | 1972 | 149.32 | 4.91 | 87 | 434.51 | 3.50 | 0.25 | A | 7.00 | 6.37 | 1.82 |
| 2012 | 10_26 | 7 | 10_41 | 5 | IE | P | 364 | 593.95 | 2.51 | 1630 | 146.12 | 4.32 | 58 | 369.25 | 3.59 | 0.25 | A | 7.00 | 3.69 | 1.05 |
| 2012 | 10_26 | 7 | 10_41 | 5 | IE | P | 379 | 605.79 | 1.95 | 1596 | 149.35 | 5.09 | 79 | 440.71 | 4.13 | 0.25 | A | 7.00 | 6.66 | 1.90 |
| 2012 | 10_26 | 7 | 10_41 | 5 | IE | P | 474 | 601.09 | 2.84 | 1940 | 145.06 | 4.71 | 83 | 437.31 | 3.92 | 0.24 | A | 7.00 | 7.10 | 2.03 |
| 2012 | 10_26 | 7 | 10_41 | 5 | IE | P | 414 | 598.82 | 2.17 | 1839 | 146.60 | 4.97 | 67 | 382.65 | 3.52 | 0.24 | A | 7.00 | 4.27 | 1.22 |
| 2012 | 10_26 | 7 | 10_41 | 5 | IE | P | 304 | 606.31 | 2.26 | 2007 | 148.55 | 5.21 | 107 | 435.16 | 4.77 | 0.25 | A | 7.00 | 6.51 | 1.86 |
| 2012 | 10_26 | 7 | 10_41 | 5 | IE | P | 420 | 600.65 | 2.09 | 2162 | 148.14 | 4.97 | 128 | 431.27 | 3.87 | 0.25 | A | 7.00 | 6.38 | 1.82 |
| 2012 | 10_26 | 7 | 10_41 | 5 | IE | P | 385 | 595.09 | 2.18 | 1806 | 147.81 | 4.77 | 108 | 441.88 | 3.39 | 0.25 | A | 7.00 | 6.93 | 1.98 |
| 2012 | 10_26 | 7 | 10_41 | 5 | IE | P | 316 | 598.44 | 2.28 | 1335 | 150.09 | 5.08 | 57 | 414.08 | 2.66 | 0.25 | A | 7.00 | 5.31 | 1.52 |
| 2012 | 10_28 | 7 | 10_01 | 4 | IE | P | 247 | 608.00 | 3.92 | 1893 | 152.12 | 4.17 | 83 | 433.23 | 3.57 | 0.25 | A | 7.00 | 5.94 | 1.70 |
| 2012 | 10_28 | 7 | 10_01 | 4 | IE | P | 403 | 603.39 | 4.26 | 1989 | 151.73 | 5.49 | 89 | 374.44 | 5.07 | 0.25 | A | 7.00 | 3.27 | 0.94 |
| 2012 | 10_28 | 7 | 10_01 | 4 | IE | P | 368 | 592.58 | 3.73 | 1998 | 148.10 | 5.24 | 110 | 438.28 | 4.98 | 0.25 | A | 7.00 | 6.72 | 1.92 |
| 2012 | 10_28 | 7 | 10_01 | 4 | IE | P | 248 | 607.84 | 2.93 | 2105 | 150.37 | 4.58 | 74 | 444.42 | 4.11 | 0.25 | A | 7.00 | 6.69 | 1.91 |
| 2012 | 10_28 | 7 | 10_01 | 4 | IE | P | 276 | 609.03 | 3.81 | 2152 | 152.65 | 4.93 | 98 | 445.29 | 4.11 | 0.25 | A | 7.00 | 6.42 | 1.83 |
| 2012 | 10_28 | 7 | 10_01 | 4 | IE | P | 419 | 604.08 | 2.96 | 1893 | 149.89 | 4.29 | 93 | 374.19 | 3.46 | 0.25 | A | 7.00 | 3.48 | 0.99 |
| 2012 | 10_28 | 7 | 10_01 | 4 | IE | P | 730 | 600.06 | 3.71 | 1514 | 154.48 | 5.28 | 133 | 240.70 | 4.81 | 0.26 | S | 7.27 | 3.21 | 0.92 |
| 2012 | 10_28 | 7 | 10_01 | 4 | IE | P | 319 | 609.31 | 2.79 | 1977 | 147.78 | 3.78 | 65 | 433.41 | 4.19 | 0.24 | A | 7.00 | 6.53 | 1.87 |
| 2012 | 10_28 | 7 | 10_01 | 4 | IE | P | 331 | 611.72 | 2.31 | 1953 | 149.99 | 3.71 | 104 | 443.45 | 4.08 | 0.25 | A | 7.00 | 6.70 | 1.91 |
| 2012 | 10_28 | 7 | 10_03 | 5 | IE | P | 181 | 593.48 | 2.20 | 2020 | 144.26 | 4.57 | 82 | 430.07 | 3.28 | 0.24 | A | 7.00 | 6.87 | 1.96 |
| 2012 | 10_28 | 7 | 10_22 | 4 | IE | P | 263 | 605.84 | 3.51 | 1587 | 154.26 | 3.84 | 52 | 380.06 | 3.96 | 0.25 | A | 7.00 | 3.25 | 0.93 |
| 2012 | 10_28 | 7 | 10_22 | 4 | IE | P | 434 | 617.52 | 5.26 | 1601 | 158.74 | 5.30 | 94 | 388.27 | 5.79 | 0.26 | A | 7.00 | 3.12 | 0.89 |
| 2012 | 10_28 | 7 | 10_22 | 4 | IE | P | 399 | 596.02 | 6.69 | 1797 | 154.77 | 5.86 | 90 | 390.92 | 5.39 | 0.26 | A | 7.00 | 3.68 | 1.05 |
| 2012 | 10_28 | 7 | 10_22 | 4 | IE | P | 112 | 605.52 | 3.49 | 869 | 152.23 | 4.56 | 48 | 390.29 | 3.36 | 0.25 | A | 7.00 | 3.95 | 1.13 |
| 2012 | 10_28 | 7 | 10_26 | 7 | IA | P | 209 | 599.01 | 2.33 | 2125 | 143.08 | 4.82 | 107 | 212.61 | 4.10 | 0.24 | S | 6.75 | 3.47 | 0.99 |
| 2012 | 10_28 | 7 | 10_26 | 7 | IA | P | 113 | 594.37 | 2.00 | 1434 | 134.92 | 5.70 | 87 | 206.04 | 3.51 | 0.23 | S | 6.41 | 3.03 | 0.87 |
| 2012 | 10_28 | 7 | 10_26 | 7 | IA | P | 229 | 594.73 | 2.39 | 695 | 156.93 | 8.09 | 278 | 228.28 | 3.57 | 0.26 | S | 7.45 | 4.06 | 1.16 |
| 2012 | 10_28 | 7 | 10_28 | 7 | S | P | 132 | 607.90 | 2.81 | 1866 | 158.53 | 5.20 | 118 | 449.61 | 3.80 | 0.26 | A | 7.00 | 5.85 | 1.67 |
| 2012 | 10_28 | 7 | 10_28 | 7 | S | P | 193 | 610.60 | 2.06 | 1368 | 73.64 | 8.93 | 240 | 217.70 | 4.78 | 0.12 | HP | 3.41 |  |  |
| 2012 | 10_28 | 7 | 10_28 | 7 | S | P | 138 | 603.08 | 2.25 | 1995 | 149.66 | 5.05 | 101 | 431.14 | 4.20 | 0.25 | A | 7.00 | 6.17 | 1.76 |
| 2012 | 10_28 | 7 | 10_28 | 7 | S | P | 233 | 599.23 | 2.28 | 1901 | 145.62 | 5.41 | 130 | 223.47 | 4.56 | 0.24 | S | 6.86 | 3.19 | 0.91 |
| 2012 | 10_28 | 7 | 10_28 | 7 | S | P | 198 | 602.49 | 1.91 | 1935 | 161.21 | 5.29 | 123 | 465.36 | 4.77 | 0.27 | A | 7.00 | 6.21 | 1.77 |
| 2012 | 10_28 | 7 | 10_28 | 7 | S | P | 298 | 602.77 | 2.28 | 3350 | 148.23 | 4.64 | 127 | 425.76 | 2.94 | 0.25 | A | 7.00 | 6.11 | 1.74 |
| 2012 | 10_28 | 7 | 10_32 | 7 | IA | P | 264 | 603.81 | 3.05 | 1857 | 149.38 | 4.37 | 105 | 444.16 | 3.47 | 0.25 | A | 7.00 | 6.81 | 1.95 |
| 2012 | 10_28 | 7 | 10_32 | 7 | IA | P | 225 | 599.03 | 2.58 | 2023 | 147.41 | 4.75 | 107 | 430.89 | 4.77 | 0.25 | A | 7.00 | 6.46 | 1.85 |
| 2012 | 10_28 | 7 | 10_33 | 5 | IE | P | 406 | 592.27 | 3.51 | 1576 | 219.38 | 4.65 | 109 | 380.22 | 4.09 | 0.37 | AF | 10.46 |  |  |
| 2012 | 10_28 | 7 | 10_33 | 5 | IE | P | 373 | 595.01 | 2.16 | 1869 | 146.35 | 4.20 | 137 | 238.44 | 4.13 | 0.25 | S | 6.95 | 2.58 | 0.74 |
| 2012 | 10_28 | 7 | 10_33 | 5 | IE | P | 340 | 585.33 | 2.45 | 1970 | 146.30 | 4.70 | 73 | 399.00 | 2.68 | 0.25 | A | 7.00 | 5.09 | 1.45 |
| 2012 | 10_28 | 7 | 10_33 | 5 | IE | P | 374 | 586.01 | 2.82 | 1860 | 147.34 | 5.01 | 123 | 404.96 | 4.67 | 0.25 | A | 7.00 | 5.24 | 1.50 |
| 2012 | 10_28 | 7 | 10_33 | 5 | IE | P | 403 | 591.23 | 2.10 | 1846 | 146.14 | 4.38 | 103 | 433.31 | 3.20 | 0.25 | A | 7.00 | 6.76 | 1.93 |
| 2012 | 10_28 | 7 | 10_33 | 5 | IE | P | 316 | 593.82 | 2.17 | 2007 | 145.86 | 4.35 | 71 | 377.87 | 3.85 | 0.25 | A | 7.00 | 4.13 | 1.18 |
| 2012 | 10_28 | 7 | 10_33 | 5 | IE | P | 250 | 605.62 | 2.20 | 1383 | 152.17 | 4.70 | 48 | 390.24 | 4.35 | 0.25 | A | 7.00 | 3.95 | 1.13 |
| 2012 | 10_28 | 7 | 10_33 | 5 | IE | P | 377 | 594.89 | 2.46 | 1496 | 148.91 | 4.73 | 128 | 394.86 | 6.18 | 0.25 | A | 7.00 | 4.56 | 1.30 |
| 2012 | 10_28 | 7 | 10_33 | 5 | IE | P | 242 | 603.84 | 2.33 | 2139 | 148.05 | 4.75 | 104 | 428.27 | 4.89 | 0.25 | A | 7.00 | 6.25 | 1.79 |
| 2012 | 10_28 | 7 | 10_33 | 5 | IE | P | 312 | 603.95 | 2.55 | 1709 | 150.51 | 5.28 | 115 | 401.31 | 5.90 | 0.25 | A | 7.00 | 4.66 | 1.33 |
| 2012 | 10_28 | 7 | 10_41 | 5 | IE | P | 261 | 602.61 | 2.46 | 1915 | 147.01 | 4.19 | 131 | 420.05 | 3.98 | 0.24 | A | 7.00 | 6.00 | 1.71 |
| 2012 | 10_28 | 7 | 10_41 | 5 | IE | P | 281 | 594.85 | 2.90 | 1833 | 147.43 | 4.52 | 113 | 432.17 | 4.51 | 0.25 | A | 7.00 | 6.52 | 1.86 |
| 2012 | 10_28 | 7 | 10_41 | 5 | IE | P | 315 | 600.69 | 2.88 | 1787 | 150.22 | 5.42 | 101 | 466.17 | 4.80 | 0.25 | A | 7.00 | 7.72 | 2.21 |
| 2012 | 10_28 | 7 | 10_41 | 5 | IE | P | 238 | 595.07 | 2.76 | 2015 | 147.01 | 4.73 | 60 | 442.69 | 3.90 | 0.25 | A | 7.00 | 7.08 | 2.02 |
| 2012 | 10_28 | 7 | 10_41 | 5 | IE | P | 283 | 598.94 | 2.95 | 2060 | 145.32 | 3.69 | 72 | 413.26 | 4.04 | 0.24 | A | 7.00 | 5.91 | 1.69 |
| 2012 | 10_28 | 7 | 10_41 | 5 | IE | P | 261 | 599.05 | 2.46 | 1884 | 147.30 | 4.41 | 86 | 454.63 | 3.17 | 0.25 | A | 7.00 | 7.60 | 2.17 |
| 2012 | 10_28 | 7 | 10_41 | 5 | IE | P | 283 | 592.97 | 2.40 | 1924 | 145.40 | 4.30 | 102 | 438.53 | 4.31 | 0.25 | A | 7.00 | 7.11 | 2.03 |
| 2012 | 10_28 | 7 | 10_41 | 5 | IE | P | 345 | 595.98 | 2.16 | 1807 | 145.64 | 4.62 | 92 | 367.92 | 4.23 | 0.24 | A | 7.00 | 3.68 | 1.05 |
| 2012 | 10_28 | 7 | 10_41 | 5 | IE | P | 297 | 590.28 | 2.39 | 1887 | 143.58 | 4.59 | 95 | 424.00 | 5.27 | 0.24 | A | 7.00 | 6.67 | 1.91 |
| 2012 | 10_28 | 7 | 10_41 | 5 | IE | P | 249 | 592.26 | 1.95 | 1673 | 145.43 | 4.31 | 120 | 436.84 | 4.06 | 0.25 | A | 7.00 | 7.03 | 2.01 |
| 2012 | 10_28 | 7 | 10_42 | 5 | IE | P | 272 | 588.05 | 2.54 | 2050 | 144.56 | 4.72 | 98 | 423.89 | 4.37 | 0.25 | A | 7.00 | 6.53 | 1.86 |
| 2012 | 10_32 | 7 | 10_03 | 5 | IE | P | 144 | 636.33 | 2.27 | 1681 | 163.15 | 4.74 | 139 | 466.89 | 4.26 | 0.26 | A | 7.00 | 6.03 | 1.72 |
| 2012 | 10_32 | 7 | 10_06 | 5 | IE | P | 188 | 600.23 | 2.95 | 2127 | 146.34 | 5.29 | 83 | 476.16 | 4.20 | 0.24 | A | 7.00 | 8.78 | 2.51 |
| 2012 | 10_32 | 7 | 10_08 | 4 | IE | P | 61 | 602.34 | 2.45 | 1064 | 151.76 | 4.48 | 44 | 391.65 | 4.17 | 0.25 | A | 7.00 | 4.07 | 1.16 |
| 2012 | 10_32 | 7 | 10_08 | 4 | IE | P | 251 | 600.55 | 3.08 | 2139 | 147.82 | 4.48 | 75 | 383.22 | 3.98 | 0.25 | A | 7.00 | 4.15 | 1.18 |
| 2012 | 10_32 | 7 | 10_08 | 4 | IE | P | 126 | 609.80 | 2.20 | 2193 | 148.29 | 4.65 | 60 | 387.48 | 3.44 | 0.24 | A | 7.00 | 4.29 | 1.23 |
| 2012 | 10_32 | 7 | 10_08 | 4 | IE | P | 121 | 599.47 | 2.27 | 2017 | 146.29 | 4.62 | 92 | 381.20 | 3.94 | 0.24 | A | 7.00 | 4.24 | 1.21 |
| 2012 | 10_32 | 7 | 10_17 | 4 | IE | P | 355 | 608.72 | 3.08 | 1926 | 152.74 | 5.85 | 107 | 447.77 | 3.04 | 0.25 | A | 7.00 | 6.52 | 1.86 |
| 2012 | 10_32 | 7 | 10_22 | 4 | IE | P | 422 | 609.73 | 3.07 | 1889 | 150.71 | 4.79 | 87 | 383.09 | 3.77 | 0.25 | A | 7.00 | 3.79 | 1.08 |
| 2012 | 10_32 | 7 | 10_22 | 4 | IE | P | 367 | 604.05 | 3.10 | 2123 | 149.35 | 4.93 | 111 | 379.74 | 4.75 | 0.25 | A | 7.00 | 3.80 | 1.09 |
| 2012 | 10_32 | 7 | 10_32 | 7 | S | P | 107 | 600.13 | 2.02 | 1872 | 151.91 | 4.49 | 102 | 436.93 | 2.90 | 0.25 | A | 7.00 | 6.13 | 1.75 |
| 2012 | 10_32 | 7 | 10_32 | 7 | S | P | 154 | 606.28 | 1.57 | 2140 | 150.27 | 4.52 | 108 | 422.20 | 3.63 | 0.25 | A | 7.00 | 5.67 | 1.62 |
| 2012 | 10_32 | 7 | 10_32 | 7 | S | P | 187 | 583.54 | 1.92 | 1938 | 144.39 | 4.85 | 67 | 373.83 | 4.61 | 0.25 | A | 7.00 | 4.12 | 1.18 |
| 2012 | 10_32 | 7 | 10_32 | 7 | S | P | 122 | 594.67 | 1.98 | 2010 | 149.82 | 4.77 | 108 | 428.54 | 2.88 | 0.25 | A | 7.00 | 6.02 | 1.72 |
| 2012 | 10_33 | 5 | 10_01 | 4 | IE | P | 284 | 597.31 | 2.12 | 2181 | 107.17 | 4.96 | 108 | 300.73 | 2.95 | 0.18 | A | 5.00 | 4.03 | 1.61 |
| 2012 | 10_33 | 5 | 10_01 | 4 | IE | P | 314 | 589.86 | 2.06 | 1918 | 104.77 | 4.75 | 84 | 463.45 | 3.80 | 0.18 | A3 | 5.00 |  |  |
| 2012 | 10_33 | 5 | 10_01 | 4 | IE | P | 310 | 588.55 | 2.59 | 1949 | 105.98 | 4.65 | 116 | 294.19 | 3.89 | 0.18 | A | 5.00 | 3.88 | 1.55 |
| 2012 | 10_33 | 5 | 10_01 | 4 | IE | P | 579 | 590.48 | 3.52 | 1760 | 108.18 | 5.83 | 133 | 307.21 | 5.02 | 0.18 | A | 5.00 | 4.20 | 1.68 |
| 2012 | 10_33 | 5 | 10_01 | 4 | IE | P | 607 | 596.44 | 3.10 | 1602 | 147.36 | 4.97 | 93 | 342.31 | 4.17 | 0.25 | AF | 6.86 |  |  |
| 2012 | 10_33 | 5 | 10_01 | 4 | IE | P | 331 | 609.06 | 2.27 | 2049 | 108.79 | 5.40 | 109 | 302.80 | 3.59 | 0.18 | A | 5.00 | 3.92 | 1.57 |
| 2012 | 10_33 | 5 | 10_01 | 4 | IE | P | 332 | 596.63 | 2.27 | 2032 | 108.30 | 4.80 | 92 | 303.36 | 3.37 | 0.18 | A | 5.00 | 4.01 | 1.60 |
| 2012 | 10_33 | 5 | 10_01 | 4 | IE | P | 341 | 601.40 | 2.19 | 2077 | 108.60 | 5.18 | 94 | 285.81 | 5.16 | 0.18 | A | 5.00 | 3.16 | 1.26 |
| 2012 | 10_33 | 5 | 10_01 | 4 | IE | P | 296 | 608.21 | 2.25 | 2146 | 108.09 | 5.31 | 99 | 305.45 | 3.58 | 0.18 | A | 5.00 | 4.13 | 1.65 |
| 2012 | 10_33 | 5 | 10_01 | 4 | IE | P | 217 | 599.13 | 2.38 | 2080 | 109.02 | 5.76 | 128 | 303.60 | 3.78 | 0.18 | A | 5.00 | 3.92 | 1.57 |
| 2012 | 10_33 | 5 | 10_26 | 7 | IE | P | 135 | 596.82 | 2.36 | 1000 | 109.24 | 5.07 | 51 | 346.13 | 4.18 | 0.18 | A | 5.00 | 5.84 | 2.34 |
| 2012 | 10_33 | 5 | 10_26 | 7 | IE | P | 320 | 601.26 | 2.20 | 1702 | 110.33 | 5.75 | 127 | 373.57 | 5.08 | 0.18 | A | 5.00 | 6.93 | 2.77 |
| 2012 | 10_33 | 5 | 10_26 | 7 | IE | P | 324 | 602.86 | 2.14 | 1453 | 109.48 | 5.99 | 72 | 372.93 | 4.40 | 0.18 | A | 5.00 | 7.03 | 2.81 |
| 2012 | 10_33 | 5 | 10_26 | 7 | IE | P | 500 | 595.70 | 2.66 | 1904 | 107.71 | 5.84 | 119 | 365.34 | 5.96 | 0.18 | A | 5.00 | 6.96 | 2.78 |
| 2012 | 10_33 | 5 | 10_26 | 7 | IE | P | 273 | 596.17 | 2.45 | 1770 | 107.10 | 5.13 | 66 | 368.28 | 3.16 | 0.18 | A | 5.00 | 7.19 | 2.88 |
| 2012 | 10_33 | 5 | 10_26 | 7 | IE | P | 410 | 597.81 | 2.23 | 1961 | 107.35 | 5.64 | 115 | 368.66 | 3.92 | 0.18 | A | 5.00 | 7.17 | 2.87 |
| 2012 | 10_33 | 5 | 10_26 | 7 | IE | P | 407 | 599.87 | 2.86 | 2006 | 107.56 | 5.32 | 120 | 349.72 | 5.39 | 0.18 | A | 5.00 | 6.26 | 2.50 |
| 2012 | 10_33 | 5 | 10_26 | 7 | IE | P | 383 | 604.31 | 2.26 | 1600 | 108.59 | 5.54 | 91 | 368.35 | 3.87 | 0.18 | A | 5.00 | 6.96 | 2.78 |
| 2012 | 10_33 | 5 | 10_26 | 7 | IE | P | 374 | 599.47 | 2.30 | 1531 | 208.55 | 3.78 | 100 | 320.32 | 3.82 | 0.35 | AF | 9.66 |  |  |
| 2012 | 10_33 | 5 | 10_28 | 7 | IE | P | 238 | 609.48 | 2.84 | 1968 | 110.68 | 6.19 | 126 | 372.26 | 4.07 | 0.18 | A | 5.00 | 6.82 | 2.73 |
| 2012 | 10_33 | 5 | 10_28 | 7 | IE | P | 178 | 599.81 | 2.71 | 2203 | 106.17 | 5.65 | 106 | 334.79 | 3.90 | 0.18 | A | 5.00 | 5.77 | 2.31 |
| 2012 | 10_33 | 5 | 10_32 | 7 | IE | P | 394 | 603.71 | 2.62 | 1387 | 183.32 | 4.39 | 81 | 300.33 | 3.92 | 0.30 | AF | 8.43 |  |  |
| 2012 | 10_33 | 5 | 10_32 | 7 | IE | P | 430 | 598.65 | 2.73 | 1935 | 108.81 | 6.02 | 86 | 362.79 | 5.52 | 0.18 | A | 5.00 | 6.67 | 2.67 |
| 2012 | 10_33 | 5 | 10_32 | 7 | IE | P | 456 | 599.48 | 2.54 | 1612 | 183.69 | 5.43 | 99 | 291.60 | 3.79 | 0.31 | AF | 8.51 |  |  |
| 2012 | 10_33 | 5 | 10_32 | 7 | IE | P | 411 | 597.03 | 2.37 | 2133 | 106.44 | 5.21 | 88 | 293.33 | 3.51 | 0.18 | A | 5.00 | 3.78 | 1.51 |
| 2012 | 10_33 | 5 | 10_32 | 7 | IE | P | 315 | 597.91 | 2.34 | 1098 | 106.99 | 5.19 | 56 | 344.81 | 3.71 | 0.18 | A | 5.00 | 6.11 | 2.45 |
| 2012 | 10_33 | 5 | 10_32 | 7 | IE | P | 250 | 591.58 | 2.78 | 1342 | 105.75 | 4.96 | 55 | 336.58 | 4.81 | 0.18 | A | 5.00 | 5.91 | 2.37 |
| 2012 | 10_33 | 5 | 10_32 | 7 | IE | P | 434 | 596.39 | 2.52 | 2588 | 106.05 | 4.96 | 77 | 348.53 | 4.88 | 0.18 | A | 5.00 | 6.43 | 2.57 |
| 2012 | 10_33 | 5 | 10_32 | 7 | IE | P | 584 | 603.01 | 2.46 | 1731 | 179.50 | 4.56 | 104 | 295.65 | 3.80 | 0.30 | AF | 8.26 |  |  |
| 2012 | 10_33 | 5 | 10_32 | 7 | IE | P | 451 | 593.68 | 3.39 | 1866 | 167.89 | 4.63 | 81 | 282.32 | 4.30 | 0.28 | AF | 7.85 |  |  |
| 2012 | 10_33 | 5 | 10_32 | 7 | IE | P | 325 | 597.53 | 2.01 | 2141 | 105.97 | 4.86 | 88 | 294.22 | 5.24 | 0.18 | A | 5.00 | 3.88 | 1.55 |
| 2012 | 10_41 | 5 | 10_03 | 5 | IA | P | 170 | 602.97 | 2.07 | 2144 | 105.64 | 5.49 | 182 | 300.69 | 3.62 | 0.18 | A | 5.00 | 4.23 | 1.69 |
| 2012 | 10_41 | 5 | 10_08 | 4 | IE | P | 228 | 589.82 | 2.24 | 2136 | 103.28 | 5.18 | 126 | 289.41 | 4.11 | 0.18 | A | 5.00 | 4.01 | 1.60 |
| 2012 | 10_41 | 5 | 10_17 | 4 | IE | P | 243 | 593.87 | 2.35 | 1935 | 104.31 | 5.71 | 111 | 291.25 | 3.70 | 0.18 | A | 5.00 | 3.96 | 1.58 |
| 2012 | 10_41 | 5 | 10_28 | 7 | IE | P | 160 | 598.13 | 2.45 | 2264 | 104.73 | 5.72 | 146 | 281.16 | 3.39 | 0.18 | A | 5.00 | 3.42 | 1.37 |
| 2012 | 10_41 | 5 | 10_32 | 7 | IE | P | 140 | 594.93 | 2.44 | 2249 | 103.50 | 5.27 | 119 | 418.63 | 4.36 | 0.17 | A | 5.00 | 10.22 | 4.09 |
| 2012 | 10_41 | 5 | 10_32 | 7 | IE | P | 141 | 595.77 | 2.39 | 2117 | 201.57 | 4.03 | 172 | 282.21 | 3.87 | 0.34 | AF | 9.39 |  |  |
| 2012 | 10_41 | 5 | 10_33 | 5 | IA | P | 260 | 594.67 | 2.04 | 2045 | 111.64 | 4.65 | 154 | 168.43 | 4.08 | 0.19 | S | 5.21 | 2.56 | 1.02 |
| 2012 | 10_41 | 5 | 10_33 | 5 | IA | P | 144 | 598.42 | 2.06 | 1054 | 106.17 | 5.08 | 56 | 328.44 | 3.37 | 0.18 | A | 5.00 | 5.47 | 2.19 |
| 2012 | 10_42 | 5 | 10_01 | 4 | IE | P | 205 | 597.31 | 2.19 | 2228 | 106.04 | 5.20 | 131 | 290.93 | 3.26 | 0.18 | A | 5.00 | 3.72 | 1.49 |
| 2012 | 10_42 | 5 | 10_01 | 4 | IE | P | 233 | 599.79 | 1.92 | 2176 | 106.20 | 4.93 | 116 | 292.47 | 3.30 | 0.18 | A | 5.00 | 3.77 | 1.51 |
| 2012 | 10_42 | 5 | 10_03 | 5 | IA | P | 376 | 589.15 | 3.05 | 1557 | 107.94 | 4.86 | 94 | 309.18 | 3.87 | 0.18 | A | 5.00 | 4.32 | 1.73 |
| 2012 | 10_42 | 5 | 10_03 | 5 | IA | P | 444 | 597.75 | 2.06 | 1640 | 108.75 | 5.21 | 101 | 312.15 | 3.16 | 0.18 | A | 5.00 | 4.35 | 1.74 |
| 2012 | 10_42 | 5 | 10_03 | 5 | IA | P | 554 | 589.42 | 2.85 | 1337 | 108.06 | 5.11 | 96 | 279.62 | 3.56 | 0.18 | A | 5.00 | 2.94 | 1.18 |
| 2012 | 10_42 | 5 | 10_03 | 5 | IA | P | 430 | 590.42 | 2.96 | 1738 | 107.38 | 4.90 | 111 | 305.22 | 3.62 | 0.18 | A | 5.00 | 4.21 | 1.68 |
| 2012 | 10_42 | 5 | 10_03 | 5 | IA | P | 453 | 598.56 | 2.59 | 1425 | 109.23 | 5.05 | 117 | 347.40 | 4.03 | 0.18 | A | 5.00 | 5.90 | 2.36 |
| 2012 | 10_42 | 5 | 10_03 | 5 | IA | P | 804 | 613.70 | 3.73 | 1361 | 113.89 | 5.94 | 113 | 330.83 | 5.42 | 0.19 | A | 5.00 | 4.52 | 1.81 |
| 2012 | 10_42 | 5 | 10_03 | 5 | IA | P | 552 | 600.20 | 1.92 | 1845 | 109.47 | 6.43 | 106 | 435.13 | 4.83 | 0.18 | A | 5.00 | 9.87 | 3.95 |
| 2012 | 10_42 | 5 | 10_03 | 5 | IA | P | 595 | 592.08 | 2.50 | 1748 | 107.89 | 5.92 | 78 | 277.81 | 4.90 | 0.18 | A | 5.00 | 2.87 | 1.15 |
| 2012 | 10_42 | 5 | 10_03 | 5 | IA | P | 671 | 607.16 | 3.54 | 1585 | 113.42 | 5.53 | 114 | 341.65 | 5.31 | 0.19 | A | 5.00 | 5.06 | 2.02 |
| 2012 | 10_42 | 5 | 10_03 | 5 | IA | P | 732 | 586.96 | 3.92 | 1885 | 108.00 | 6.09 | 101 | 287.65 | 4.59 | 0.18 | A | 5.00 | 3.32 | 1.33 |
| 2012 | 10_42 | 5 | 10_08 | 4 | IE | P | 260 | 602.30 | 2.16 | 2334 | 106.00 | 5.44 | 140 | 295.53 | 3.01 | 0.18 | A | 5.00 | 3.94 | 1.58 |
| 2012 | 10_42 | 5 | 10_08 | 4 | IE | P | 203 | 610.27 | 1.93 | 2188 | 109.44 | 4.84 | 113 | 303.78 | 3.34 | 0.18 | A | 5.00 | 3.88 | 1.55 |
| 2012 | 10_42 | 5 | 10_08 | 4 | IE | P | 276 | 604.02 | 2.10 | 1981 | 106.47 | 5.10 | 121 | 294.08 | 3.29 | 0.18 | A | 5.00 | 3.81 | 1.52 |
| 2012 | 10_42 | 5 | 10_17 | 4 | IE | P | 298 | 601.14 | 3.13 | 2266 | 106.42 | 6.92 | 137 | 305.04 | 5.74 | 0.18 | A | 5.00 | 4.33 | 1.73 |
| 2012 | 10_42 | 5 | 10_22 | 4 | IE | P | 334 | 614.25 | 3.39 | 1883 | 110.49 | 5.48 | 141 | 300.37 | 4.24 | 0.18 | A | 5.00 | 3.59 | 1.44 |
| 2012 | 10_42 | 5 | 10_33 | 5 | IA | P | 652 | 610.69 | 2.36 | 1500 | 109.96 | 4.24 | 95 | 330.80 | 3.51 | 0.18 | A | 5.00 | 5.04 | 2.02 |
| 2012 | 10_42 | 5 | 10_33 | 5 | IA | P | 638 | 602.56 | 2.24 | 1463 | 108.17 | 4.98 | 139 | 288.76 | 6.36 | 0.18 | A | 5.00 | 3.35 | 1.34 |
| 2012 | 10_42 | 5 | 10_33 | 5 | IA | P | 539 | 588.07 | 2.25 | 1519 | 104.38 | 4.72 | 100 | 329.95 | 4.62 | 0.18 | A | 5.00 | 5.81 | 2.32 |
| 2012 | 10_42 | 5 | 10_33 | 5 | IA | P | 777 | 596.51 | 2.79 | 1360 | 114.21 | 6.96 | 98 | 285.48 | 4.71 | 0.19 | A | 5.00 | 2.50 | 1.00 |
| 2012 | 10_42 | 5 | 10_33 | 5 | IA | P | 767 | 606.25 | 2.07 | 1287 | 112.20 | 5.74 | 109 | 287.83 | 5.75 | 0.19 | A | 5.00 | 2.83 | 1.13 |
| 2012 | 10_42 | 5 | 10_33 | 5 | IA | P | 453 | 594.09 | 2.73 | 1301 | 106.11 | 4.43 | 86 | 272.39 | 3.04 | 0.18 | A | 5.00 | 2.84 | 1.13 |
| 2012 | 10_42 | 5 | 10_33 | 5 | IA | P | 658 | 604.36 | 1.77 | 1872 | 107.28 | 5.42 | 114 | 339.11 | 3.64 | 0.18 | A | 5.00 | 5.80 | 2.32 |
| 2012 | 10_42 | 5 | 10_33 | 5 | IA | P | 406 | 597.11 | 2.65 | 1291 | 105.20 | 4.59 | 97 | 274.01 | 4.34 | 0.18 | A | 5.00 | 3.02 | 1.21 |
| 2012 | 10_42 | 5 | 10_33 | 5 | IA | P | 575 | 598.09 | 2.25 | 1545 | 105.33 | 4.75 | 104 | 325.93 | 4.75 | 0.18 | A | 5.00 | 5.47 | 2.19 |
| 2012 | 10_42 | 5 | 10_33 | 5 | IA | P | 674 | 609.40 | 2.79 | 1482 | 109.70 | 4.93 | 93 | 288.46 | 4.23 | 0.18 | A | 5.00 | 3.15 | 1.26 |
| 2012 | 13_02 | 5 | 13_09 | 4 | IE | P | 442 | 607.04 | 3.13 | 1727 | 109.27 | 5.78 | 92 | 403.75 | 4.10 | 0.18 | A | 5.00 | 8.47 | 3.39 |
| 2012 | 13_02 | 5 | 13_12 | 4 | IE | P | 314 | 601.31 | 2.98 | 2120 | 105.48 | 5.49 | 115 | 297.59 | 4.29 | 0.18 | A | 5.00 | 4.11 | 1.64 |
| 2012 | 13_02 | 5 | 13_33 | 4 | IE | P | 355 | 592.90 | 3.12 | 1707 | 202.12 | 4.28 | 90 | 483.22 | 4.41 | 0.34 | AS | 9.46 |  |  |
| 2012 | 13_05 | 5 | 13_09 | 4 | IE | P | 263 | 596.52 | 2.53 | 2060 | 105.63 | 5.25 | 101 | 292.45 | 3.34 | 0.18 | A | 5.00 | 3.84 | 1.54 |
| 2012 | 13_06 | 5 | 13_33 | 4 | IE | P | 261 | 606.16 | 3.19 | 2034 | 110.24 | 5.50 | 126 | 302.05 | 4.74 | 0.18 | A | 5.00 | 3.70 | 1.48 |
| 2012 | 13_06 | 5 | 13_33 | 4 | IE | P | 228 | 603.38 | 2.94 | 2301 | 106.82 | 5.80 | 136 | 299.93 | 3.89 | 0.18 | A | 5.00 | 4.04 | 1.62 |
| 2012 | 13_09 | 4 | 13_22 | 4 | IA | P | 277 | 605.14 | 4.31 | 1855 | 91.52 | 6.30 | 171 | 136.98 | 5.86 | 0.15 | S | 4.16 | 2.09 | 1.05 |
| 2012 | 13_09 | 4 | 13_49 | 5 | IE | P | 170 | 595.82 | 2.13 | 1793 | 141.49 | 5.20 | 116 | 226.93 | 3.92 | 0.24 | AF | 6.53 |  |  |
| 2013 | 13_09 | 4 | 14_06 | 7 | IE | P | 328 | 622.13 | 2.73 | 1384 | 118.08 | 3.27 | 132 | 166.59 | 3.16 | 0.190 | S | 5.22 |  |  |
| 2013 | 13_09 | 4 | 14_06 | 7 | IE | P | 206 | 619.02 | 2.42 | 1518 | 116.30 | 3.94 | 165 | 161.68 | 3.82 | 0.188 | S | 5.17 |  |  |
| 2013 | 13_09 | 4 | 14_06 | 7 | IE | P | 319 | 613.62 | 2.45 | 1371 | 113.99 | 3.40 | 113 | 159.62 | 3.10 | 0.186 | S | 5.11 |  |  |
| 2013 | 13_09 | 4 | 14_06 | 7 | IE | P | 301 | 611.46 | 2.90 | 1442 | 117.28 | 3.49 | 143 | 161.79 | 3.36 | 0.192 | S | 5.27 |  |  |
| 2013 | 13_09 | 4 | 18_32 | 6 | IE | P | 403 | 614.49 | 2.41 | 1650 | 108.83 | 3.39 | 158 | 157.15 | 3.16 | 0.177 | S | 4.87 |  |  |
| 2013 | 13_09 | 4 | 18_32 | 6 | IE | P | 309 | 612.60 | 2.88 | 1417 | 112.55 | 3.40 | 111 | 160.83 | 3.65 | 0.184 | S | 5.05 |  |  |
| 2013 | 13_09 | 4 | 18_32 | 6 | IE | P | 299 | 609.16 | 2.72 | 1229 | 110.38 | 3.50 | 102 | 185.48 | 3.63 | 0.181 | S | 4.98 |  |  |
| 2013 | 13_09 | 4 | 18_32 | 6 | IE | P | 269 | 615.67 | 2.96 | 1837 | 113.20 | 3.53 | 156 | 162.88 | 3.10 | 0.184 | S | 5.06 |  |  |
| 2013 | 13_09 | 4 | 18_32 | 6 | IE | P | 323 | 608.35 | 3.06 | 1610 | 109.94 | 3.34 | 183 | 155.48 | 3.26 | 0.181 | S | 4.97 |  |  |
| 2013 | 13_09 | 4 | 41_02 | 8 | IE | P | 179 | 598.07 | 3.19 | 857 | 126.48 | 3.43 | 83 | 171.70 | 3.13 | 0.211 | S | 5.81 |  |  |
| 2013 | 13_09 | 4 | 41_02 | 8 | IE | P | 254 | 612.41 | 3.17 | 745 | 132.25 | 4.42 | 73 | 178.29 | 3.86 | 0.216 | S | 5.94 |  |  |
| 2013 | 13_09 | 4 | 41_02 | 8 | IE | P | 178 | 607.06 | 3.65 | 1050 | 116.45 | 3.79 | 102 | 167.53 | 3.53 | 0.192 | S | 5.27 |  |  |
| 2012 | 13_12 | 4 | 13_09 | 4 | IA | P | 163 | 594.16 | 3.47 | 1948 | 88.35 | 6.15 | 173 | 133.21 | 4.10 | 0.15 | S | 4.09 | 2.01 | 1.01 |
| 2012 | 13_12 | 4 | 13_22 | 4 | IA | P | 386 | 611.76 | 3.78 | 1844 | 89.98 | 5.93 | 189 | 136.66 | 4.19 | 0.15 | S | 4.04 | 1.95 | 0.97 |
| 2013 | 13_12 | 4 | 15_46 | 6 | IE | P | 311 | 605.25 | 3.30 | 1147 | 112.17 | 3.93 | 157 | 158.74 | 3.34 | 0.185 | S | 5.10 |  |  |
| 2013 | 13_12 | 4 | 15_46 | 6 | IE | P | 244 | 598.60 | 4.41 | 985 | 118.28 | 4.07 | 108 | 167.14 | 3.84 | 0.198 | S | 5.43 |  |  |
| 2013 | 13_12 | 4 | 15_46 | 6 | IE | P | 195 | 604.50 | 3.06 | 1299 | 109.53 | 5.29 | 103 | 159.00 | 4.08 | 0.181 | S | 4.98 |  |  |
| 2013 | 13_12 | 4 | 15_46 | 6 | IE | P | 204 | 601.29 | 3.45 | 1273 | 115.70 | 4.11 | 106 | 164.47 | 2.98 | 0.192 | S | 5.29 |  |  |
| 2013 | 13_12 | 4 | 27_17 | 7 | IE | P | 220 | 614.52 | 2.10 | 1157 | 106.50 | 3.16 | 150 | 153.93 | 3.35 | 0.173 | S | 4.77 |  |  |
| 2013 | 13_12 | 4 | 27_17 | 7 | IE | P | 248 | 620.45 | 2.97 | 831 | 123.75 | 3.56 | 96 | 168.65 | 3.15 | 0.199 | S | 5.48 |  |  |
| 2013 | 13_12 | 4 | 27_17 | 7 | IE | P | 293 | 605.79 | 3.39 | 967 | 115.07 | 3.35 | 121 | 162.84 | 3.07 | 0.190 | S | 5.22 |  |  |
| 2013 | 13_12 | 4 | 27_17 | 7 | IE | P | 260 | 601.31 | 2.73 | 676 | 122.70 | 3.62 | 168 | 167.77 | 4.24 | 0.204 | S | 5.61 |  |  |
| 2012 | 13_17 | 5 | 13_05 | 5 | IA | P | 441 | 612.00 | 4.51 | 1741 | 107.11 | 6.20 | 126 | 269.05 | 5.99 | 0.18 | A | 5.00 | 2.56 | 1.02 |
| 2012 | 13_17 | 5 | 13_09 | 4 | IE | P | 378 | 595.88 | 3.05 | 1708 | 109.38 | 5.99 | 76 | 299.35 | 4.79 | 0.18 | A | 5.00 | 3.68 | 1.47 |
| 2012 | 13_17 | 5 | 13_09 | 4 | IE | P | 375 | 598.24 | 2.83 | 1893 | 109.14 | 5.52 | 142 | 308.11 | 4.19 | 0.18 | A | 5.00 | 4.12 | 1.65 |
| 2012 | 13_17 | 5 | 13_09 | 4 | IE | P | 339 | 610.05 | 2.22 | 1995 | 109.86 | 5.47 | 72 | 263.08 | 3.51 | 0.18 | A | 5.00 | 1.97 | 0.79 |
| 2012 | 13_17 | 5 | 13_09 | 4 | IE | P | 407 | 604.98 | 2.38 | 1859 | 108.79 | 5.29 | 118 | 305.32 | 4.15 | 0.18 | A | 5.00 | 4.03 | 1.61 |
| 2012 | 13_17 | 5 | 13_09 | 4 | IE | P | 291 | 604.32 | 2.52 | 1734 | 108.33 | 5.67 | 129 | 295.80 | 3.60 | 0.18 | A | 5.00 | 3.65 | 1.46 |
| 2012 | 13_17 | 5 | 13_09 | 4 | IE | P | 291 | 609.27 | 3.04 | 2330 | 111.56 | 5.57 | 116 | 308.26 | 5.11 | 0.18 | A | 5.00 | 3.82 | 1.53 |
| 2012 | 13_17 | 5 | 13_09 | 4 | IE | P | 376 | 609.42 | 2.24 | 1874 | 109.93 | 5.34 | 55 | 268.06 | 3.80 | 0.18 | A | 5.00 | 2.19 | 0.88 |
| 2012 | 13_17 | 5 | 13_09 | 4 | IE | P | 385 | 600.41 | 2.85 | 1734 | 155.92 | 4.68 | 189 | 262.25 | 5.22 | 0.26 | AF | 7.21 |  |  |
| 2012 | 13_17 | 5 | 13_09 | 4 | IE | P | 459 | 596.39 | 2.26 | 1861 | 107.65 | 5.07 | 112 | 304.98 | 4.74 | 0.18 | A | 5.00 | 4.17 | 1.67 |
| 2012 | 13_17 | 5 | 13_09 | 4 | IE | P | 428 | 600.90 | 2.22 | 1913 | 106.72 | 5.12 | 125 | 299.31 | 4.15 | 0.18 | A | 5.00 | 4.02 | 1.61 |
| 2012 | 13_17 | 5 | 13_17 | 5 | S | P | 324 | 596.87 | 2.02 | 1712 | 104.79 | 5.13 | 110 | 321.98 | 3.85 | 0.18 | A | 5.00 | 5.36 | 2.15 |
| 2012 | 13_17 | 5 | 13_17 | 5 | S | P | 427 | 599.04 | 2.30 | 1627 | 105.35 | 5.13 | 119 | 318.33 | 3.70 | 0.18 | A | 5.00 | 5.11 | 2.04 |
| 2012 | 13_17 | 5 | 13_18 | 4 | IE | P | 377 | 598.92 | 3.18 | 1796 | 111.85 | 5.40 | 49 | 277.77 | 3.84 | 0.19 | A | 5.00 | 2.42 | 0.97 |
| 2012 | 13_17 | 5 | 13_22 | 4 | IE | P | 481 | 596.05 | 2.67 | 1992 | 108.62 | 5.70 | 107 | 301.98 | 4.38 | 0.18 | A | 5.00 | 3.90 | 1.56 |
| 2012 | 13_17 | 5 | 13_22 | 4 | IE | P | 286 | 596.95 | 2.63 | 2002 | 107.41 | 5.08 | 132 | 303.14 | 3.98 | 0.18 | A | 5.00 | 4.11 | 1.64 |
| 2012 | 13_17 | 5 | 13_22 | 4 | IE | P | 368 | 602.04 | 2.46 | 1928 | 109.90 | 5.53 | 102 | 304.46 | 4.05 | 0.18 | A | 5.00 | 3.85 | 1.54 |
| 2012 | 13_17 | 5 | 13_22 | 4 | IE | P | 447 | 603.78 | 2.24 | 2005 | 109.32 | 5.97 | 115 | 300.04 | 4.15 | 0.18 | A | 5.00 | 3.72 | 1.49 |
| 2012 | 13_17 | 5 | 13_22 | 4 | IE | P | 510 | 611.12 | 2.36 | 1964 | 109.90 | 4.73 | 87 | 306.90 | 3.98 | 0.18 | A | 5.00 | 3.96 | 1.59 |
| 2012 | 13_17 | 5 | 13_22 | 4 | IE | P | 302 | 608.97 | 3.06 | 1722 | 114.42 | 5.33 | 132 | 316.35 | 3.47 | 0.19 | A | 5.00 | 3.82 | 1.53 |
| 2012 | 13_17 | 5 | 13_22 | 4 | IE | P | 284 | 600.19 | 2.69 | 1968 | 109.00 | 5.02 | 124 | 307.95 | 3.56 | 0.18 | A | 5.00 | 4.13 | 1.65 |
| 2012 | 13_17 | 5 | 13_22 | 4 | IE | P | 356 | 603.83 | 2.41 | 1997 | 109.64 | 4.96 | 146 | 307.58 | 4.42 | 0.18 | A | 5.00 | 4.03 | 1.61 |
| 2012 | 13_17 | 5 | 13_22 | 4 | IE | P | 323 | 598.56 | 2.35 | 2076 | 106.55 | 4.85 | 88 | 304.59 | 3.79 | 0.18 | A | 5.00 | 4.29 | 1.72 |
| 2012 | 13_17 | 5 | 13_22 | 4 | IE | P | 440 | 605.90 | 2.05 | 2013 | 108.02 | 5.42 | 136 | 300.57 | 5.65 | 0.18 | A | 5.00 | 3.91 | 1.57 |
| 2012 | 13_22 | 4 | 13_05 | 5 | IE | P | 406 | 593.04 | 2.23 | 1656 | 103.01 | 5.42 | 192 | 146.71 | 4.73 | 0.17 | S | 4.78 | 2.75 | 1.37 |
| 2012 | 13_22 | 4 | 13_05 | 5 | IE | P | 233 | 599.17 | 2.31 | 1974 | 90.79 | 6.49 | 230 | 139.59 | 5.69 | 0.15 | S | 4.17 | 1.93 | 0.96 |
| 2012 | 13_22 | 4 | 13_05 | 5 | IE | P | 236 | 600.08 | 2.04 | 1767 | 103.73 | 5.28 | 196 | 151.70 | 4.43 | 0.17 | S | 4.75 | 2.55 | 1.28 |
| 2012 | 13_22 | 4 | 13_05 | 5 | IE | P | 176 | 596.36 | 2.01 | 2003 | 96.64 | 5.34 | 204 | 139.76 | 4.46 | 0.16 | S | 4.46 | 2.47 | 1.23 |
| 2012 | 13_22 | 4 | 13_05 | 5 | IE | P | 250 | 587.67 | 2.27 | 1750 | 85.74 | 6.03 | 144 | 132.38 | 4.09 | 0.15 | S | 4.01 | 1.83 | 0.91 |
| 2012 | 13_22 | 4 | 13_05 | 5 | IE | P | 127 | 588.11 | 2.23 | 2075 | 85.73 | 6.10 | 155 | 128.21 | 3.48 | 0.15 | S | 4.01 | 2.02 | 1.01 |
| 2012 | 13_22 | 4 | 13_05 | 5 | IE | P | 334 | 593.59 | 2.66 | 1623 | 93.44 | 6.07 | 254 | 138.12 | 4.14 | 0.16 | S | 4.33 | 2.26 | 1.13 |
| 2012 | 13_22 | 4 | 13_05 | 5 | IE | P | 381 | 602.13 | 2.53 | 997 | 108.94 | 7.47 | 375 | 145.27 | 4.12 | 0.18 | S | 4.97 | 3.32 | 1.66 |
| 2012 | 13_22 | 4 | 13_05 | 5 | IE | P | 346 | 601.95 | 2.19 | 2124 | 94.79 | 5.63 | 227 | 140.42 | 5.52 | 0.16 | S | 4.33 | 2.25 | 1.12 |
| 2012 | 13_22 | 4 | 13_05 | 5 | IE | P | 183 | 597.66 | 2.38 | 2098 | 90.28 | 6.11 | 142 | 134.45 | 4.46 | 0.15 | S | 4.15 | 2.12 | 1.06 |
| 2012 | 13_22 | 4 | 13_09 | 4 | IA | P | 370 | 596.06 | 3.82 | 1642 | 86.83 | 5.92 | 192 | 132.07 | 5.64 | 0.15 | S | 4.01 | 1.92 | 0.96 |
| 2012 | 13_22 | 4 | 13_12 | 4 | IA | P | 364 | 606.43 | 3.92 | 1499 | 89.09 | 5.62 | 154 | 134.86 | 4.98 | 0.15 | S | 4.04 | 1.96 | 0.98 |
| 2012 | 13_22 | 4 | 13_33 | 4 | IA | P | 361 | 620.48 | 3.32 | 1765 | 90.21 | 5.66 | 173 | 135.41 | 4.57 | 0.15 | S | 4.00 | 1.99 | 1.00 |
| 2012 | 13_22 | 4 | 13_49 | 5 | IE | P | 403 | 602.01 | 2.28 | 1563 | 108.50 | 5.45 | 199 | 152.89 | 4.49 | 0.18 | S | 4.96 | 2.93 | 1.46 |
| 2012 | 13_22 | 4 | 13_49 | 5 | IE | P | 320 | 592.63 | 2.10 | 1888 | 98.47 | 5.22 | 195 | 143.52 | 4.12 | 0.17 | S | 4.57 | 2.48 | 1.24 |
| 2012 | 13_22 | 4 | 13_49 | 5 | IE | P | 281 | 592.36 | 1.88 | 2205 | 91.29 | 5.56 | 222 | 137.56 | 3.97 | 0.15 | S | 4.24 | 2.09 | 1.04 |
| 2012 | 13_22 | 4 | 13_49 | 5 | IE | P | 313 | 595.25 | 2.09 | 1574 | 100.79 | 6.06 | 274 | 141.32 | 4.59 | 0.17 | S | 4.66 | 2.78 | 1.39 |
| 2012 | 13_22 | 4 | 13_49 | 5 | IE | P | 222 | 585.88 | 3.03 | 1983 | 88.05 | 5.83 | 162 | 131.54 | 3.93 | 0.15 | S | 4.13 | 2.09 | 1.05 |
| 2012 | 13_22 | 4 | 13_49 | 5 | IE | P | 345 | 590.08 | 2.11 | 1882 | 105.14 | 5.38 | 157 | 150.32 | 3.24 | 0.18 | S | 4.90 | 2.79 | 1.40 |
| 2012 | 13_22 | 4 | 13_49 | 5 | IE | P | 417 | 593.95 | 2.03 | 1556 | 100.82 | 6.51 | 183 | 142.84 | 3.69 | 0.17 | S | 4.67 | 2.72 | 1.36 |
| 2012 | 13_22 | 4 | 13_49 | 5 | IE | P | 383 | 594.93 | 2.29 | 1521 | 97.83 | 6.00 | 243 | 140.71 | 3.88 | 0.16 | S | 4.52 | 2.54 | 1.27 |
| 2012 | 13_22 | 4 | 13_49 | 5 | IE | P | 362 | 597.89 | 2.11 | 1805 | 105.86 | 5.47 | 190 | 149.51 | 4.01 | 0.18 | S | 4.87 | 2.86 | 1.43 |
| 2012 | 13_22 | 4 | 13_49 | 5 | IE | P | 451 | 598.29 | 1.94 | 1550 | 97.88 | 6.20 | 284 | 141.77 | 4.15 | 0.16 | S | 4.50 | 2.48 | 1.24 |
| 2013 | 13_22 | 4 | 14_19 | 8 | IE | P | 297 | 610.83 | 3.03 | 1448 | 91.28 | 3.97 | 182 | 136.10 | 3.37 | 0.149 | S | 4.11 |  |  |
| 2013 | 13_22 | 4 | 14_19 | 8 | IE | P | 341 | 604.45 | 3.50 | 1335 | 133.11 | 3.24 | 101 | 175.21 | 3.28 | 0.220 | S | 6.05 |  |  |
| 2013 | 13_22 | 4 | 14_19 | 8 | IE | P | 312 | 626.86 | 3.56 | 1011 | 118.24 | 3.70 | 114 | 167.78 | 3.25 | 0.189 | S | 5.19 |  |  |
| 2013 | 13_22 | 4 | 18_05 | 6 | IE | P | 515 | 613.71 | 3.53 | 990 | 112.23 | 3.57 | 90 | 155.92 | 3.11 | 0.183 | S | 5.03 |  |  |
| 2013 | 13_22 | 4 | 18_05 | 6 | IE | P | 212 | 613.32 | 3.04 | 696 | 111.95 | 3.39 | 91 | 156.71 | 3.17 | 0.183 | S | 5.02 |  |  |
| 2013 | 13_22 | 4 | 18_05 | 6 | IE | P | 585 | 610.89 | 2.98 | 1166 | 111.14 | 3.25 | 127 | 154.99 | 3.62 | 0.182 | S | 5.00 |  |  |
| 2013 | 13_22 | 4 | 18_05 | 6 | IE | P | 162 | 615.76 | 2.65 | 608 | 105.77 | 3.36 | 84 | 147.24 | 4.38 | 0.172 | S | 4.72 |  |  |
| 2013 | 13_22 | 4 | 18_05 | 6 | IE | P | 363 | 602.07 | 3.81 | 1707 | 105.69 | 4.12 | 140 | 148.47 | 3.42 | 0.176 | S | 4.83 |  |  |
| 2012 | 13_33 | 4 | 13_12 | 4 | IA | P | 335 | 600.15 | 2.47 | 1988 | 85.78 | 5.67 | 160 | 131.68 | 4.41 | 0.14 | S | 3.93 | 1.83 | 0.91 |
| 2012 | 13_33 | 4 | 13_12 | 4 | IA | P | 334 | 608.81 | 1.74 | 1961 | 86.04 | 5.49 | 140 | 132.99 | 4.14 | 0.14 | S | 3.89 | 1.77 | 0.88 |
| 2012 | 13_33 | 4 | 13_12 | 4 | IA | P | 327 | 595.91 | 1.98 | 1847 | 85.44 | 5.69 | 141 | 132.06 | 4.02 | 0.14 | S | 3.94 | 1.79 | 0.90 |
| 2012 | 13_33 | 4 | 13_12 | 4 | IA | P | 428 | 596.22 | 1.84 | 2766 | 86.46 | 6.03 | 260 | 136.21 | 5.03 | 0.15 | S | 3.99 | 1.69 | 0.85 |
| 2012 | 13_33 | 4 | 13_12 | 4 | IA | P | 339 | 591.60 | 1.97 | 1841 | 85.96 | 6.06 | 157 | 133.77 | 4.32 | 0.15 | S | 4.00 | 1.77 | 0.89 |
| 2012 | 13_33 | 4 | 13_12 | 4 | IA | P | 278 | 599.78 | 1.56 | 1934 | 86.41 | 5.57 | 148 | 133.03 | 5.22 | 0.14 | S | 3.96 | 1.82 | 0.91 |
| 2012 | 13_33 | 4 | 13_12 | 4 | IA | P | 379 | 599.85 | 2.02 | 1964 | 86.57 | 5.60 | 126 | 132.62 | 3.67 | 0.14 | S | 3.97 | 1.86 | 0.93 |
| 2012 | 13_33 | 4 | 13_12 | 4 | IA | P | 401 | 597.73 | 2.13 | 1825 | 86.72 | 6.09 | 140 | 139.87 | 4.90 | 0.15 | S | 3.99 | 1.54 | 0.77 |
| 2012 | 13_33 | 4 | 13_12 | 4 | IA | P | 374 | 600.96 | 1.87 | 1897 | 87.11 | 5.69 | 165 | 138.39 | 5.23 | 0.14 | S | 3.99 | 1.64 | 0.82 |
| 2012 | 13_33 | 4 | 13_12 | 4 | IA | P | 257 | 593.11 | 1.96 | 1941 | 85.76 | 6.29 | 186 | 135.26 | 5.49 | 0.14 | S | 3.98 | 1.68 | 0.84 |
| 2012 | 13_33 | 4 | 13_18 | 4 | IA | P | 343 | 609.29 | 3.35 | 2195 | 89.45 | 6.00 | 150 | 134.31 | 4.29 | 0.15 | S | 4.04 | 2.01 | 1.01 |
| 2012 | 13_33 | 4 | 13_22 | 4 | IA | P | 435 | 599.19 | 2.60 | 1417 | 88.10 | 5.84 | 149 | 138.14 | 5.11 | 0.15 | S | 4.04 | 1.75 | 0.87 |
| 2012 | 13_33 | 4 | 13_22 | 4 | IA | P | 361 | 599.97 | 2.29 | 1476 | 88.15 | 6.11 | 139 | 135.16 | 4.36 | 0.15 | S | 4.04 | 1.89 | 0.94 |
| 2012 | 13_33 | 4 | 13_22 | 4 | IA | P | 524 | 600.75 | 2.35 | 1416 | 87.84 | 6.06 | 218 | 135.96 | 4.47 | 0.15 | S | 4.02 | 1.82 | 0.91 |
| 2012 | 13_33 | 4 | 13_22 | 4 | IA | P | 456 | 610.29 | 2.55 | 1568 | 88.30 | 6.30 | 157 | 139.32 | 4.71 | 0.14 | S | 3.98 | 1.68 | 0.84 |
| 2012 | 13_33 | 4 | 13_22 | 4 | IA | P | 295 | 600.20 | 2.45 | 1868 | 87.40 | 6.23 | 184 | 137.22 | 4.61 | 0.15 | S | 4.00 | 1.72 | 0.86 |
| 2012 | 13_33 | 4 | 13_22 | 4 | IA | P | 523 | 602.71 | 2.11 | 1789 | 87.57 | 6.03 | 138 | 133.04 | 3.96 | 0.15 | S | 3.99 | 1.92 | 0.96 |
| 2012 | 13_33 | 4 | 13_22 | 4 | IA | P | 329 | 597.84 | 3.14 | 1713 | 85.44 | 5.89 | 197 | 133.11 | 4.20 | 0.14 | S | 3.93 | 1.74 | 0.87 |
| 2012 | 13_33 | 4 | 13_22 | 4 | IA | P | 567 | 593.72 | 3.16 | 1631 | 86.34 | 6.46 | 177 | 132.89 | 5.17 | 0.15 | S | 4.00 | 1.84 | 0.92 |
| 2012 | 13_33 | 4 | 13_22 | 4 | IA | P | 518 | 593.60 | 2.83 | 1628 | 85.48 | 6.21 | 179 | 133.35 | 4.80 | 0.14 | S | 3.96 | 1.74 | 0.87 |
| 2012 | 13_33 | 4 | 13_22 | 4 | IA | P | 539 | 590.59 | 3.24 | 1461 | 86.04 | 6.24 | 199 | 135.50 | 4.93 | 0.15 | S | 4.01 | 1.70 | 0.85 |
| 2012 | 13_33 | 4 | 13_33 | 4 | S | P | 127 | 603.68 | 1.95 | 1833 | 87.73 | 6.06 | 130 | 136.31 | 4.32 | 0.15 | S | 4.00 | 1.78 | 0.89 |
| 2012 | 13_33 | 4 | 13_49 | 5 | IE | P | 166 | 597.58 | 2.22 | 1887 | 118.42 | 5.11 | 211 | 158.92 | 4.14 | 0.20 | S | 5.45 | 3.59 | 1.79 |
| 2012 | 13_49 | 5 | 13_02 | 5 | IA | P | 128 | 570.05 | 3.30 | 688 | 98.06 | 4.84 | 44 | 291.47 | 4.16 | 0.17 | A | 5.00 | 4.86 | 1.94 |
| 2012 | 13_49 | 5 | 13_09 | 4 | IE | P | 342 | 601.92 | 3.15 | 1758 | 108.83 | 5.58 | 121 | 296.55 | 4.45 | 0.18 | A | 5.00 | 3.62 | 1.45 |
| 2012 | 13_49 | 5 | 13_17 | 5 | IA | P | 370 | 610.29 | 3.79 | 1951 | 108.49 | 6.48 | 128 | 314.02 | 3.62 | 0.18 | A | 5.00 | 4.47 | 1.79 |
| 2012 | 13_49 | 5 | 13_18 | 4 | IE | P | 359 | 600.02 | 3.09 | 1907 | 107.59 | 5.85 | 104 | 303.32 | 4.22 | 0.18 | A | 5.00 | 4.10 | 1.64 |
| 2012 | 13_49 | 5 | 13_33 | 4 | IE | P | 445 | 603.97 | 3.18 | 1614 | 107.04 | 5.52 | 96 | 298.35 | 4.33 | 0.18 | A | 5.00 | 3.94 | 1.57 |
| 2012 | 14_05 | 8 | 14_05 | 6 | IE | L | 116 | 698.96 | 2.91 | 2218 | 131.78 | 4.62 | 94 | 201.81 | 3.37 | 0.19 | S | 8.10 | 3.79 | 0.95 |
| 2012 | 14_05 | 8 | 14_23 | 7 | IE | L | 219 | 701.19 | 3.44 | 2008 | 126.43 | 4.85 | 87 | 354.31 | 5.80 | 0.18 | A | 8.00 | 6.42 | 1.60 |
| 2012 | 14_05 | 8 | 14_24 | 8 | IA | L | 169 | 702.33 | 3.20 | 2118 | 121.30 | 5.70 | 56 | 189.25 | 4.38 | 0.17 | S | 7.42 | 3.26 | 0.82 |
| 2012 | 14_06 | 7 | 14_06 | 7 | S | P | 309 | 605.90 | 2.18 | 2012 | 148.41 | 4.80 | 114 | 435.84 | 3.77 | 0.24 | A | 7.00 | 6.56 | 1.87 |
| 2012 | 14_06 | 7 | 14_06 | 7 | S | P | 225 | 605.19 | 2.15 | 2037 | 161.61 | 4.65 | 63 | 462.01 | 4.51 | 0.27 | A | 7.00 | 6.01 | 1.72 |
| 2012 | 14_06 | 7 | 14_14 | 7 | IA | P | 164 | 604.33 | 2.35 | 1909 | 152.62 | 4.69 | 116 | 433.74 | 4.19 | 0.25 | A | 7.00 | 5.89 | 1.68 |
| 2012 | 14_06 | 7 | 14_22 | 8 | IE | P | 500 | 635.54 | 5.78 | 1928 | 161.67 | 5.84 | 106 | 471.97 | 5.63 | 0.25 | A | 7.00 | 6.44 | 1.84 |
| 2012 | 14_06 | 7 | 14_22 | 8 | IE | P | 506 | 621.40 | 6.03 | 1801 | 156.62 | 5.78 | 130 | 444.40 | 6.49 | 0.25 | A | 7.00 | 5.86 | 1.67 |
| 2012 | 14_06 | 7 | 14_22 | 8 | IE | P | 528 | 616.75 | 5.44 | 2225 | 155.14 | 5.74 | 132 | 438.23 | 5.63 | 0.25 | A | 7.00 | 5.77 | 1.65 |
| 2012 | 14_06 | 7 | 14_22 | 8 | IE | P | 460 | 604.01 | 4.44 | 1899 | 153.18 | 5.32 | 74 | 457.14 | 4.89 | 0.25 | A | 7.00 | 6.89 | 1.97 |
| 2012 | 14_06 | 7 | 14_22 | 8 | IE | P | 351 | 609.91 | 2.15 | 1859 | 147.28 | 3.39 | 65 | 447.75 | 3.68 | 0.24 | A | 7.00 | 7.28 | 2.08 |
| 2012 | 14_06 | 7 | 14_22 | 8 | IE | P | 488 | 595.47 | 3.34 | 1808 | 148.41 | 4.50 | 110 | 441.45 | 4.37 | 0.25 | A | 7.00 | 6.82 | 1.95 |
| 2012 | 14_06 | 7 | 14_22 | 8 | IE | P | 488 | 612.40 | 2.76 | 1824 | 153.09 | 4.32 | 90 | 433.30 | 4.15 | 0.25 | A | 7.00 | 5.81 | 1.66 |
| 2012 | 14_06 | 7 | 14_22 | 8 | IE | P | 516 | 608.79 | 2.26 | 1787 | 148.20 | 3.39 | 107 | 437.01 | 3.50 | 0.24 | A | 7.00 | 6.64 | 1.90 |
| 2012 | 14_06 | 7 | 14_22 | 8 | IE | P | 280 | 622.02 | 1.62 | 1461 | 152.11 | 3.31 | 42 | 451.55 | 2.78 | 0.24 | A | 7.00 | 6.78 | 1.94 |
| 2012 | 14_06 | 7 | 14_26 | 8 | IE | P | 328 | 620.20 | 2.53 | 1426 | 155.56 | 3.96 | 67 | 452.04 | 3.01 | 0.25 | A | 7.00 | 6.34 | 1.81 |
| 2012 | 14_06 | 7 | 14_26 | 8 | IE | P | 66 | 614.71 | 2.07 | 460 | 150.56 | 3.26 | 24 | 466.91 | 1.94 | 0.24 | A | 7.00 | 7.71 | 2.20 |
| 2012 | 14_06 | 7 | 14_26 | 8 | IE | P | 370 | 589.42 | 5.11 | 1935 | 147.63 | 4.67 | 101 | 429.74 | 4.60 | 0.25 | A | 7.00 | 6.38 | 1.82 |
| 2012 | 14_06 | 7 | 14_26 | 8 | IE | P | 527 | 615.62 | 4.25 | 1623 | 155.27 | 4.56 | 120 | 446.91 | 5.02 | 0.25 | A | 7.00 | 6.15 | 1.76 |
| 2012 | 14_06 | 7 | 14_26 | 8 | IE | P | 398 | 615.22 | 3.62 | 1941 | 155.72 | 4.32 | 83 | 468.30 | 4.24 | 0.25 | A | 7.00 | 7.05 | 2.01 |
| 2012 | 14_06 | 7 | 14_26 | 8 | IE | P | 321 | 625.03 | 3.53 | 1422 | 160.50 | 4.30 | 83 | 453.01 | 4.10 | 0.26 | A | 7.00 | 5.76 | 1.64 |
| 2012 | 14_06 | 7 | 14_26 | 8 | IE | P | 352 | 609.13 | 2.50 | 1809 | 149.29 | 3.27 | 95 | 448.55 | 3.59 | 0.25 | A | 7.00 | 7.03 | 2.01 |
| 2012 | 14_06 | 7 | 14_26 | 8 | IE | P | 383 | 610.70 | 2.36 | 1823 | 151.41 | 3.64 | 81 | 461.00 | 3.59 | 0.25 | A | 7.00 | 7.31 | 2.09 |
| 2012 | 14_06 | 7 | 14_26 | 8 | IE | P | 576 | 603.32 | 3.57 | 1916 | 153.34 | 4.83 | 71 | 398.09 | 4.46 | 0.25 | A | 7.00 | 4.17 | 1.19 |
| 2012 | 14_06 | 7 | 14_26 | 8 | IE | P | 533 | 600.65 | 4.34 | 1493 | 154.21 | 5.42 | 65 | 399.62 | 4.41 | 0.26 | A | 7.00 | 4.14 | 1.18 |
| 2012 | 14_12 | 5 | 14_22 | 8 | IE | P | 151 | 604.27 | 2.32 | 2702 | 111.65 | 5.36 | 128 | 374.12 | 4.02 | 0.18 | A | 5.00 | 6.75 | 2.70 |
| 2012 | 14_12 | 5 | 14_22 | 8 | IE | P | 205 | 606.15 | 2.52 | 2048 | 109.16 | 5.82 | 84 | 382.04 | 3.79 | 0.18 | A | 5.00 | 7.50 | 3.00 |
| 2012 | 14_12 | 5 | 14_23 | 7 | IE | P | 171 | 604.76 | 2.68 | 2567 | 107.61 | 5.96 | 84 | 366.54 | 3.58 | 0.18 | A | 5.00 | 7.03 | 2.81 |
| 2012 | 14_12 | 5 | 14_28 | 5 | IA | P | 332 | 613.24 | 4.20 | 2218 | 115.19 | 6.59 | 155 | 322.03 | 4.62 | 0.19 | A | 5.00 | 3.98 | 1.59 |
| 2012 | 14_12 | 5 | 14_28 | 5 | IA | P | 431 | 615.19 | 4.06 | 1852 | 110.53 | 6.27 | 131 | 345.97 | 5.81 | 0.18 | A | 5.00 | 5.65 | 2.26 |
| 2012 | 14_13 | 7 | 14_19 | 8 | IE | P | 262 | 604.20 | 2.97 | 1858 | 149.57 | 4.39 | 92 | 443.05 | 2.81 | 0.25 | A | 7.00 | 6.74 | 1.92 |
| 2012 | 14_13 | 7 | 14_22 | 8 | IE | P | 341 | 599.41 | 3.04 | 1822 | 146.45 | 4.39 | 108 | 448.19 | 4.25 | 0.24 | A | 7.00 | 7.42 | 2.12 |
| 2012 | 14_13 | 7 | 14_22 | 8 | IE | P | 452 | 601.68 | 2.86 | 1890 | 148.68 | 4.64 | 103 | 405.07 | 2.93 | 0.25 | A | 7.00 | 5.07 | 1.45 |
| 2012 | 14_13 | 7 | 14_22 | 8 | IE | P | 372 | 597.17 | 3.06 | 1894 | 147.79 | 4.92 | 97 | 469.08 | 3.14 | 0.25 | A | 7.00 | 8.22 | 2.35 |
| 2012 | 14_13 | 7 | 14_22 | 8 | IE | P | 403 | 595.01 | 3.07 | 1608 | 149.02 | 5.02 | 87 | 411.59 | 3.37 | 0.25 | A | 7.00 | 5.33 | 1.52 |
| 2012 | 14_13 | 7 | 14_22 | 8 | IE | P | 344 | 604.75 | 2.50 | 1943 | 146.39 | 4.74 | 120 | 451.40 | 3.63 | 0.24 | A | 7.00 | 7.58 | 2.17 |
| 2012 | 14_13 | 7 | 14_22 | 8 | IE | P | 352 | 612.01 | 2.40 | 1815 | 148.47 | 4.36 | 81 | 439.14 | 2.39 | 0.24 | A | 7.00 | 6.70 | 1.92 |
| 2012 | 14_13 | 7 | 14_22 | 8 | IE | P | 393 | 595.75 | 3.16 | 1982 | 145.99 | 4.81 | 97 | 387.46 | 3.35 | 0.25 | A | 7.00 | 4.58 | 1.31 |
| 2012 | 14_13 | 7 | 14_22 | 8 | IE | P | 381 | 604.44 | 2.88 | 1943 | 148.82 | 4.73 | 84 | 450.96 | 3.75 | 0.25 | A | 7.00 | 7.21 | 2.06 |
| 2012 | 14_13 | 7 | 14_24 | 8 | IE | P | 499 | 605.01 | 3.02 | 1926 | 144.51 | 4.66 | 91 | 419.09 | 4.05 | 0.24 | A | 7.00 | 6.30 | 1.80 |
| 2012 | 14_13 | 7 | 14_24 | 8 | IE | P | 594 | 604.44 | 2.80 | 1523 | 147.37 | 4.89 | 93 | 444.45 | 3.91 | 0.24 | A | 7.00 | 7.11 | 2.03 |
| 2012 | 14_13 | 7 | 14_28 | 5 | IE | P | 142 | 604.31 | 1.81 | 2019 | 148.81 | 4.31 | 108 | 417.85 | 3.66 | 0.25 | A | 7.00 | 5.66 | 1.62 |
| 2012 | 14_13 | 7 | 14_32 | 7 | IA | P | 235 | 595.50 | 2.63 | 1633 | 226.09 | 3.85 | 131 | 347.48 | 3.99 | 0.38 | S | 10.72 | 4.97 | 1.42 |
| 2012 | 14_14 | 7 | 14_14 | 7 | S | L | 167 | 714.88 | 2.77 | 2157 | 115.42 | 5.79 | 129 | 357.10 | 3.52 | 0.16 | A | 7.00 | 7.66 | 2.19 |
| 2012 | 14_14 | 7 | 14_14 | 7 | S | P | 541 | 593.36 | 2.85 | 1635 | 146.69 | 6.37 | 64 | 445.75 | 3.66 | 0.25 | A | 7.00 | 7.27 | 2.08 |
| 2012 | 14_14 | 7 | 14_14 | 7 | S | P | 615 | 590.48 | 2.53 | 1907 | 145.44 | 5.66 | 77 | 450.77 | 4.04 | 0.25 | A | 7.00 | 7.70 | 2.20 |
| 2012 | 14_14 | 7 | 14_19 | 8 | IE | P | 433 | 609.13 | 2.80 | 1989 | 159.39 | 7.91 | 90 | 501.26 | 4.02 | 0.26 | A | 7.00 | 8.01 | 2.29 |
| 2012 | 14_14 | 7 | 14_19 | 8 | IE | P | 393 | 598.49 | 2.63 | 2067 | 155.09 | 6.48 | 92 | 462.64 | 5.07 | 0.26 | A | 7.00 | 6.88 | 1.97 |
| 2012 | 14_14 | 7 | 14_19 | 8 | IE | P | 442 | 600.69 | 2.52 | 1801 | 158.59 | 6.63 | 80 | 475.81 | 4.12 | 0.26 | A | 7.00 | 7.00 | 2.00 |
| 2012 | 14_14 | 7 | 14_19 | 8 | IE | P | 441 | 612.33 | 2.91 | 1901 | 159.23 | 6.88 | 55 | 485.39 | 3.55 | 0.26 | A | 7.00 | 7.34 | 2.10 |
| 2012 | 14_14 | 7 | 14_21 | 5 | IE | P | 268 | 610.86 | 2.00 | 1808 | 151.84 | 5.22 | 97 | 445.86 | 3.82 | 0.25 | A | 7.00 | 6.55 | 1.87 |
| 2012 | 14_14 | 7 | 14_22 | 8 | IE | P | 340 | 612.80 | 2.63 | 1622 | 152.02 | 4.60 | 82 | 455.01 | 4.17 | 0.25 | A | 7.00 | 6.95 | 1.99 |
| 2012 | 14_14 | 7 | 14_22 | 8 | IE | P | 361 | 611.81 | 2.14 | 1927 | 150.54 | 4.67 | 82 | 450.54 | 3.23 | 0.25 | A | 7.00 | 6.95 | 1.99 |
| 2012 | 14_14 | 7 | 14_22 | 8 | IE | P | 299 | 612.91 | 2.60 | 1592 | 151.65 | 4.60 | 68 | 450.00 | 4.51 | 0.25 | A | 7.00 | 6.77 | 1.93 |
| 2012 | 14_14 | 7 | 14_24 | 8 | IE | P | 415 | 609.26 | 3.58 | 1928 | 148.24 | 4.94 | 85 | 433.14 | 3.62 | 0.24 | A | 7.00 | 6.45 | 1.84 |
| 2012 | 14_14 | 7 | 14_32 | 7 | IA | P | 169 | 603.68 | 2.32 | 2019 | 151.55 | 5.59 | 99 | 428.99 | 3.77 | 0.25 | A | 7.00 | 5.81 | 1.66 |
| 2012 | 14_15 | 5 | 14_06 | 7 | IE | P | 805 | 606.35 | 3.44 | 1430 | 111.39 | 4.93 | 86 | 357.95 | 4.04 | 0.18 | A | 5.00 | 6.07 | 2.43 |
| 2012 | 14_15 | 5 | 14_06 | 7 | IE | P | 631 | 607.73 | 2.32 | 1663 | 111.48 | 4.65 | 101 | 330.86 | 3.96 | 0.18 | A | 5.00 | 4.84 | 1.94 |
| 2012 | 14_15 | 5 | 14_06 | 7 | IE | P | 978 | 608.64 | 4.64 | 1341 | 110.76 | 5.39 | 108 | 366.96 | 4.73 | 0.18 | A | 5.00 | 6.57 | 2.63 |
| 2012 | 14_15 | 5 | 14_06 | 7 | IE | P | 937 | 581.74 | 4.49 | 1419 | 106.81 | 4.56 | 105 | 358.87 | 3.82 | 0.18 | A | 5.00 | 6.80 | 2.72 |
| 2012 | 14_15 | 5 | 14_06 | 7 | IE | P | 985 | 587.53 | 3.61 | 1407 | 108.57 | 5.84 | 141 | 365.91 | 4.65 | 0.18 | A | 5.00 | 6.85 | 2.74 |
| 2012 | 14_15 | 5 | 14_06 | 7 | IE | P | 842 | 590.26 | 2.92 | 1435 | 106.62 | 6.85 | 73 | 328.82 | 3.16 | 0.18 | A | 5.00 | 5.42 | 2.17 |
| 2012 | 14_15 | 5 | 14_06 | 7 | IE | P | 987 | 603.19 | 2.07 | 1323 | 87.59 | 5.85 | 90 | 138.96 | 4.12 | 0.15 | S | 4.03 | 1.67 | 0.67 |
| 2012 | 14_15 | 5 | 14_06 | 7 | IE | P | 860 | 588.08 | 2.39 | 1492 | 105.76 | 5.52 | 84 | 324.10 | 3.39 | 0.18 | A | 5.00 | 5.32 | 2.13 |
| 2012 | 14_15 | 5 | 14_06 | 7 | IE | P | 544 | 602.00 | 2.47 | 1654 | 110.91 | 4.94 | 117 | 305.34 | 4.77 | 0.18 | A | 5.00 | 3.77 | 1.51 |
| 2012 | 14_15 | 5 | 14_06 | 7 | IE | P | 891 | 590.85 | 3.80 | 1421 | 110.46 | 4.83 | 121 | 305.01 | 4.49 | 0.19 | A | 5.00 | 3.81 | 1.52 |
| 2012 | 14_15 | 5 | 14_19 | 8 | IE | P | 98 | 596.72 | 4.02 | 1907 | 103.68 | 6.09 | 82 | 365.03 | 4.75 | 0.17 | A | 5.00 | 7.60 | 3.04 |
| 2012 | 14_15 | 5 | 14_22 | 8 | IE | P | 155 | 592.25 | 2.11 | 2359 | 105.41 | 5.65 | 133 | 364.19 | 3.28 | 0.18 | A | 5.00 | 7.27 | 2.91 |
| 2012 | 14_15 | 5 | 14_24 | 8 | IE | P | 243 | 594.72 | 3.04 | 1778 | 107.53 | 6.11 | 136 | 415.40 | 4.05 | 0.18 | A | 5.00 | 9.32 | 3.73 |
| 2012 | 14_15 | 5 | 14_24 | 8 | IE | P | 192 | 595.21 | 2.33 | 2251 | 103.55 | 5.21 | 99 | 322.14 | 2.97 | 0.17 | A | 5.00 | 5.55 | 2.22 |
| 2012 | 14_15 | 5 | 14_28 | 5 | IA | P | 440 | 602.78 | 3.59 | 1632 | 109.92 | 6.01 | 90 | 306.86 | 3.55 | 0.18 | A | 5.00 | 3.96 | 1.58 |
| 2012 | 14_15 | 5 | 14_32 | 7 | IE | P | 604 | 619.50 | 4.68 | 1680 | 109.53 | 5.67 | 83 | 373.35 | 2.79 | 0.18 | A | 5.00 | 7.04 | 2.82 |
| 2012 | 14_15 | 5 | 14_32 | 7 | IE | P | 906 | 602.10 | 2.65 | 1329 | 111.38 | 5.69 | 89 | 296.69 | 4.74 | 0.18 | A | 5.00 | 3.32 | 1.33 |
| 2012 | 14_15 | 5 | 14_32 | 7 | IE | P | 669 | 593.10 | 3.54 | 1525 | 109.69 | 6.12 | 131 | 357.62 | 4.29 | 0.18 | A | 5.00 | 6.30 | 2.52 |
| 2012 | 14_15 | 5 | 14_32 | 7 | IE | P | 624 | 608.76 | 3.42 | 1721 | 110.42 | 5.79 | 100 | 316.29 | 3.80 | 0.18 | A | 5.00 | 4.32 | 1.73 |
| 2012 | 14_15 | 5 | 14_32 | 7 | IE | P | 854 | 612.84 | 4.90 | 1380 | 111.56 | 5.98 | 122 | 359.90 | 4.09 | 0.18 | A | 5.00 | 6.13 | 2.45 |
| 2012 | 14_15 | 5 | 14_32 | 7 | IE | P | 821 | 604.75 | 3.79 | 1122 | 111.08 | 6.26 | 99 | 395.64 | 4.54 | 0.18 | A | 5.00 | 7.81 | 3.12 |
| 2012 | 14_15 | 5 | 14_32 | 7 | IE | P | 666 | 596.56 | 3.78 | 1823 | 107.14 | 5.50 | 106 | 306.30 | 4.54 | 0.18 | A | 5.00 | 4.29 | 1.72 |
| 2012 | 14_15 | 5 | 14_32 | 7 | IE | P | 436 | 598.12 | 3.12 | 1637 | 108.87 | 6.53 | 76 | 285.48 | 5.17 | 0.18 | A | 5.00 | 3.11 | 1.24 |
| 2012 | 14_15 | 5 | 14_32 | 7 | IE | P | 1088 | 597.72 | 1.71 | 1084 | 105.93 | 4.44 | 100 | 308.08 | 4.53 | 0.18 | A | 5.00 | 4.54 | 1.82 |
| 2012 | 14_15 | 5 | 14_32 | 7 | IE | P | 1282 | 600.27 | 2.14 | 891 | 106.76 | 4.63 | 112 | 277.63 | 5.06 | 0.18 | A | 5.00 | 3.00 | 1.20 |
| 2012 | 14_19 | 8 | 14_06 | 7 | IE | L | 205 | 694.65 | 2.32 | 1548 | 122.59 | 4.31 | 66 | 346.44 | 5.22 | 0.18 | A | 8.00 | 6.61 | 1.65 |
| 2012 | 14_19 | 8 | 14_15 | 5 | IE | L | 235 | 707.42 | 2.49 | 2018 | 124.08 | 5.11 | 63 | 324.65 | 3.26 | 0.18 | A | 8.00 | 4.93 | 1.23 |
| 2012 | 14_19 | 8 | 14_19 | 8 | S | L | 126 | 707.30 | 2.76 | 2552 | 125.19 | 4.81 | 46 | 359.03 | 3.46 | 0.18 | A | 8.00 | 6.94 | 1.74 |
| 2012 | 14_19 | 8 | 14_19 | 8 | S | L | 147 | 685.17 | 2.76 | 2378 | 122.98 | 5.03 | 53 | 355.65 | 4.54 | 0.18 | A | 8.00 | 7.14 | 1.78 |
| 2012 | 14_19 | 8 | 14_19 | 8 | S | L | 90 | 703.68 | 2.35 | 2193 | 124.88 | 4.92 | 37 | 355.52 | 3.57 | 0.18 | A | 8.00 | 6.78 | 1.69 |
| 2012 | 14_19 | 8 | 14_22 | 8 | IA | L | 117 | 702.65 | 2.99 | 2790 | 124.61 | 4.81 | 32 | 352.05 | 4.48 | 0.18 | A | 8.00 | 6.60 | 1.65 |
| 2012 | 14_19 | 8 | 14_23 | 7 | IE | L | 171 | 711.43 | 2.97 | 1800 | 128.36 | 4.94 | 81 | 353.02 | 6.42 | 0.18 | A | 8.00 | 6.00 | 1.50 |
| 2012 | 14_19 | 8 | 14_23 | 7 | IE | L | 181 | 707.27 | 3.38 | 2069 | 132.78 | 4.95 | 164 | 285.14 | 4.91 | 0.19 | A | 8.00 | 1.18 | 0.29 |
| 2012 | 14_19 | 8 | 14_24 | 8 | IA | L | 135 | 708.03 | 2.49 | 2249 | 127.36 | 5.03 | 48 | 365.18 | 3.14 | 0.18 | A | 8.00 | 6.94 | 1.73 |
| 2012 | 14_19 | 8 | 14_24 | 8 | IA | L | 177 | 724.18 | 2.67 | 2209 | 130.11 | 5.46 | 47 | 353.88 | 4.99 | 0.18 | A | 8.00 | 5.76 | 1.44 |
| 2012 | 14_19 | 8 | 14_28 | 5 | IE | L | 273 | 700.52 | 3.20 | 2443 | 123.27 | 4.26 | 70 | 315.59 | 5.79 | 0.18 | A | 8.00 | 4.48 | 1.12 |
| 2012 | 14_20 | 5 | 14_12 | 5 | IA | P | 240 | 610.25 | 4.15 | 2088 | 119.45 | 10.03 | 118 | 417.74 | 5.43 | 0.20 | A | 5.00 | 7.49 | 2.99 |
| 2012 | 14_20 | 5 | 14_19 | 8 | IE | P | 110 | 599.45 | 2.77 | 2233 | 110.59 | 4.95 | 167 | 369.73 | 3.22 | 0.18 | A | 5.00 | 6.72 | 2.69 |
| 2012 | 14_20 | 5 | 14_23 | 7 | IE | P | 123 | 611.15 | 2.28 | 1964 | 110.71 | 6.04 | 97 | 334.90 | 3.82 | 0.18 | A | 5.00 | 5.13 | 2.05 |
| 2012 | 14_20 | 5 | 14_24 | 8 | IE | P | 371 | 592.86 | 2.14 | 1615 | 181.81 | 3.40 | 100 | 291.55 | 3.23 | 0.31 | AF | 8.51 |  |  |
| 2012 | 14_20 | 5 | 14_24 | 8 | IE | P | 199 | 593.01 | 2.56 | 2409 | 105.60 | 5.20 | 114 | 306.19 | 3.35 | 0.18 | A | 5.00 | 4.50 | 1.80 |
| 2012 | 14_20 | 5 | 14_24 | 8 | IE | P | 222 | 600.46 | 1.90 | 2178 | 107.77 | 4.90 | 87 | 369.89 | 3.83 | 0.18 | A | 5.00 | 7.16 | 2.86 |
| 2012 | 14_20 | 5 | 14_24 | 8 | IE | P | 240 | 604.55 | 1.75 | 1737 | 186.45 | 3.82 | 104 | 302.98 | 3.84 | 0.31 | AF | 8.56 |  |  |
| 2012 | 14_20 | 5 | 14_24 | 8 | IE | P | 240 | 599.38 | 2.17 | 2351 | 108.02 | 5.42 | 94 | 369.51 | 4.56 | 0.18 | A | 5.00 | 7.10 | 2.84 |
| 2012 | 14_20 | 5 | 14_24 | 8 | IE | P | 258 | 601.59 | 2.08 | 1873 | 187.34 | 3.80 | 140 | 306.45 | 3.62 | 0.31 | AF | 8.65 |  |  |
| 2012 | 14_20 | 5 | 14_24 | 8 | IE | P | 205 | 594.45 | 2.44 | 1916 | 108.93 | 4.73 | 117 | 372.20 | 3.80 | 0.18 | A | 5.00 | 7.08 | 2.83 |
| 2012 | 14_20 | 5 | 14_24 | 8 | IE | P | 122 | 595.50 | 2.13 | 2199 | 109.23 | 4.97 | 114 | 398.51 | 3.10 | 0.18 | A | 5.00 | 8.24 | 3.30 |
| 2012 | 14_20 | 5 | 14_24 | 8 | IE | P | 243 | 600.31 | 1.99 | 1762 | 109.68 | 5.44 | 118 | 323.32 | 3.94 | 0.18 | A | 5.00 | 4.74 | 1.90 |
| 2012 | 14_20 | 5 | 14_24 | 8 | IE | P | 199 | 596.96 | 1.80 | 2165 | 107.13 | 4.82 | 78 | 368.63 | 3.45 | 0.18 | A | 5.00 | 7.20 | 2.88 |
| 2012 | 14_20 | 5 | 14_26 | 8 | IE | P | 221 | 596.92 | 3.56 | 1617 | 213.72 | 5.13 | 51 | 460.39 | 5.06 | 0.36 | AS | 9.94 |  |  |
| 2012 | 14_20 | 5 | 14_26 | 8 | IE | P | 265 | 604.08 | 2.14 | 2087 | 110.25 | 5.10 | 95 | 306.31 | 4.08 | 0.18 | A | 5.00 | 3.89 | 1.56 |
| 2012 | 14_20 | 5 | 14_26 | 8 | IE | P | 283 | 595.06 | 2.44 | 2028 | 107.73 | 5.89 | 100 | 367.75 | 4.28 | 0.18 | A | 5.00 | 7.07 | 2.83 |
| 2012 | 14_20 | 5 | 14_26 | 8 | IE | P | 191 | 598.63 | 2.12 | 2238 | 109.17 | 6.04 | 131 | 392.37 | 3.74 | 0.18 | A | 5.00 | 7.97 | 3.19 |
| 2012 | 14_20 | 5 | 14_26 | 8 | IE | P | 260 | 596.78 | 2.27 | 2223 | 108.69 | 5.37 | 123 | 352.72 | 4.46 | 0.18 | A | 5.00 | 6.23 | 2.49 |
| 2012 | 14_20 | 5 | 14_26 | 8 | IE | P | 149 | 604.82 | 3.46 | 2276 | 108.21 | 4.93 | 83 | 381.35 | 3.55 | 0.18 | A | 5.00 | 7.62 | 3.05 |
| 2012 | 14_20 | 5 | 14_26 | 8 | IE | P | 241 | 597.77 | 2.03 | 1857 | 107.37 | 4.85 | 118 | 360.26 | 3.14 | 0.18 | A | 5.00 | 6.78 | 2.71 |
| 2012 | 14_20 | 5 | 14_26 | 8 | IE | P | 263 | 594.05 | 2.13 | 2358 | 105.32 | 5.05 | 102 | 359.05 | 3.98 | 0.18 | A | 5.00 | 7.05 | 2.82 |
| 2012 | 14_20 | 5 | 14_26 | 8 | IE | P | 321 | 593.02 | 2.06 | 1787 | 111.26 | 6.51 | 144 | 363.74 | 3.48 | 0.19 | A | 5.00 | 6.35 | 2.54 |
| 2012 | 14_20 | 5 | 14_26 | 8 | IE | P | 211 | 591.81 | 2.35 | 1842 | 106.41 | 5.99 | 94 | 373.75 | 3.21 | 0.18 | A | 5.00 | 7.56 | 3.02 |
| 2012 | 14_21 | 5 | 14_06 | 7 | IE | P | 143 | 591.51 | 2.93 | 1436 | 112.61 | 7.57 | 126 | 363.33 | 4.09 | 0.19 | A | 5.00 | 6.13 | 2.45 |
| 2012 | 14_21 | 5 | 14_13 | 7 | IE | P | 114 | 605.78 | 1.93 | 1591 | 112.51 | 8.31 | 116 | 340.54 | 3.32 | 0.19 | A | 5.00 | 5.13 | 2.05 |
| 2012 | 14_21 | 5 | 14_13 | 7 | IE | P | 161 | 606.76 | 2.65 | 1839 | 109.59 | 6.06 | 91 | 375.55 | 3.92 | 0.18 | A | 5.00 | 7.13 | 2.85 |
| 2012 | 14_21 | 5 | 14_22 | 8 | IE | P | 160 | 607.04 | 1.83 | 2230 | 108.41 | 5.24 | 113 | 347.99 | 3.99 | 0.18 | A | 5.00 | 6.05 | 2.42 |
| 2012 | 14_21 | 5 | 14_22 | 8 | IE | P | 149 | 594.68 | 2.61 | 2020 | 108.86 | 5.05 | 50 | 388.49 | 3.51 | 0.18 | A | 5.00 | 7.84 | 3.14 |
| 2012 | 14_21 | 5 | 14_32 | 7 | IE | P | 356 | 604.60 | 3.17 | 2042 | 107.22 | 6.13 | 126 | 290.23 | 4.08 | 0.18 | A | 5.00 | 3.53 | 1.41 |
| 2012 | 14_22 | 8 | 14_06 | 7 | IE | L | 213 | 709.46 | 3.34 | 2250 | 128.21 | 4.63 | 91 | 372.33 | 3.38 | 0.18 | A | 8.00 | 7.23 | 1.81 |
| 2012 | 14_22 | 8 | 14_06 | 7 | IE | L | 141 | 696.45 | 2.80 | 1460 | 127.26 | 4.53 | 82 | 348.70 | 3.78 | 0.18 | A | 8.00 | 5.92 | 1.48 |
| 2012 | 14_22 | 8 | 14_06 | 7 | IE | L | 189 | 701.04 | 3.05 | 2421 | 127.45 | 4.95 | 102 | 371.78 | 5.09 | 0.18 | A | 8.00 | 7.34 | 1.83 |
| 2012 | 14_22 | 8 | 14_20 | 5 | IE | L | 98 | 702.70 | 2.95 | 2356 | 126.50 | 4.98 | 77 | 331.49 | 4.42 | 0.18 | A | 8.00 | 4.96 | 1.24 |
| 2012 | 14_22 | 8 | 14_24 | 8 | IA | L | 102 | 706.65 | 2.79 | 2388 | 127.44 | 4.71 | 49 | 368.27 | 3.23 | 0.18 | A | 8.00 | 7.12 | 1.78 |
| 2012 | 14_22 | 8 | 14_32 | 7 | IE | L | 212 | 708.18 | 2.67 | 1908 | 127.29 | 4.46 | 54 | 369.70 | 3.56 | 0.18 | A | 8.00 | 7.24 | 1.81 |
| 2012 | 14_23 | 7 | 14_13 | 7 | IA | P | 244 | 594.52 | 3.08 | 1798 | 107.57 | 6.36 | 135 | 415.11 | 3.98 | 0.18 | HP | 5.11 |  |  |
| 2012 | 14_23 | 7 | 14_13 | 7 | IA | P | 206 | 611.90 | 2.35 | 2068 | 149.78 | 4.07 | 125 | 441.29 | 3.31 | 0.24 | A | 7.00 | 6.62 | 1.89 |
| 2012 | 14_23 | 7 | 14_19 | 8 | IE | P | 437 | 597.95 | 2.70 | 1728 | 146.04 | 4.23 | 107 | 431.37 | 4.02 | 0.24 | A | 7.00 | 6.68 | 1.91 |
| 2012 | 14_23 | 7 | 14_19 | 8 | IE | P | 390 | 601.69 | 2.57 | 1816 | 146.89 | 4.26 | 91 | 449.16 | 3.79 | 0.24 | A | 7.00 | 7.40 | 2.12 |
| 2012 | 14_23 | 7 | 14_19 | 8 | IE | P | 277 | 604.37 | 2.90 | 2111 | 147.91 | 4.29 | 106 | 423.72 | 3.35 | 0.24 | A | 7.00 | 6.05 | 1.73 |
| 2012 | 14_23 | 7 | 14_23 | 7 | S | P | 418 | 615.64 | 2.03 | 1908 | 151.18 | 4.30 | 99 | 458.62 | 4.10 | 0.25 | A | 7.00 | 7.24 | 2.07 |
| 2012 | 14_23 | 7 | 14_23 | 7 | S | P | 416 | 596.87 | 2.48 | 2057 | 145.86 | 4.61 | 87 | 435.62 | 4.10 | 0.24 | A | 7.00 | 6.91 | 1.97 |
| 2012 | 14_23 | 7 | 14_23 | 7 | S | P | 266 | 611.26 | 1.95 | 2272 | 148.97 | 4.76 | 109 | 455.58 | 3.59 | 0.24 | A | 7.00 | 7.41 | 2.12 |
| 2012 | 14_23 | 7 | 14_23 | 7 | S | P | 337 | 603.33 | 1.93 | 1885 | 146.97 | 4.75 | 94 | 429.29 | 3.64 | 0.24 | A | 7.00 | 6.45 | 1.84 |
| 2012 | 14_24 | 8 | 14_06 | 7 | IE | L | 119 | 714.58 | 2.55 | 2425 | 128.89 | 4.51 | 124 | 358.88 | 4.59 | 0.18 | A | 8.00 | 6.28 | 1.57 |
| 2012 | 14_24 | 8 | 14_06 | 7 | IE | L | 195 | 705.43 | 2.62 | 2170 | 125.52 | 4.74 | 96 | 350.50 | 3.16 | 0.18 | A | 8.00 | 6.34 | 1.58 |
| 2012 | 14_24 | 8 | 14_06 | 7 | IE | L | 101 | 716.32 | 3.23 | 1976 | 135.29 | 5.75 | 98 | 374.40 | 4.14 | 0.19 | A | 8.00 | 6.14 | 1.53 |
| 2012 | 14_24 | 8 | 14_06 | 7 | IE | L | 130 | 707.88 | 2.83 | 2187 | 134.87 | 5.37 | 41 | 366.63 | 3.01 | 0.19 | A | 8.00 | 5.75 | 1.44 |
| 2012 | 14_24 | 8 | 14_06 | 7 | IE | L | 195 | 690.09 | 3.33 | 2068 | 129.66 | 5.31 | 41 | 349.07 | 2.69 | 0.19 | A | 8.00 | 5.54 | 1.38 |
| 2012 | 14_24 | 8 | 14_06 | 7 | IE | L | 95 | 697.21 | 3.35 | 1856 | 131.68 | 5.06 | 65 | 378.99 | 5.08 | 0.19 | A | 8.00 | 7.02 | 1.76 |
| 2012 | 14_24 | 8 | 14_06 | 7 | IE | L | 152 | 708.41 | 2.99 | 2026 | 133.27 | 5.43 | 81 | 367.36 | 4.95 | 0.19 | A | 8.00 | 6.05 | 1.51 |
| 2012 | 14_24 | 8 | 14_06 | 7 | IE | L | 125 | 702.77 | 3.21 | 2262 | 133.96 | 5.44 | 71 | 364.66 | 4.43 | 0.19 | A | 8.00 | 5.78 | 1.44 |
| 2012 | 14_24 | 8 | 14_06 | 7 | IE | L | 176 | 700.95 | 4.16 | 2364 | 132.36 | 5.40 | 85 | 374.31 | 4.52 | 0.19 | A | 8.00 | 6.62 | 1.66 |
| 2012 | 14_24 | 8 | 14_06 | 7 | IE | L | 115 | 701.55 | 3.09 | 2105 | 132.76 | 5.31 | 60 | 370.04 | 4.13 | 0.19 | A | 8.00 | 6.30 | 1.57 |
| 2012 | 14_24 | 8 | 14_13 | 7 | IE | L | 193 | 713.15 | 2.78 | 1768 | 127.87 | 5.27 | 143 | 301.95 | 4.00 | 0.18 | A | 8.00 | 2.89 | 0.72 |
| 2012 | 14_24 | 8 | 14_14 | 7 | IE | L | 139 | 706.71 | 3.07 | 2172 | 127.63 | 4.77 | 48 | 368.26 | 4.98 | 0.18 | A | 8.00 | 7.08 | 1.77 |
| 2012 | 14_24 | 8 | 14_22 | 8 | IA | L | 175 | 699.92 | 3.03 | 2891 | 126.54 | 5.19 | 53 | 353.27 | 4.80 | 0.18 | A | 8.00 | 6.33 | 1.58 |
| 2012 | 14_24 | 8 | 14_23 | 7 | IE | L | 138 | 713.97 | 2.62 | 2130 | 126.61 | 4.49 | 79 | 344.73 | 3.49 | 0.18 | A | 8.00 | 5.78 | 1.45 |
| 2012 | 14_24 | 8 | 14_23 | 7 | IE | L | 173 | 713.84 | 3.04 | 1954 | 125.46 | 4.62 | 94 | 353.14 | 3.71 | 0.18 | A | 8.00 | 6.52 | 1.63 |
| 2012 | 14_24 | 8 | 14_23 | 7 | IE | L | 163 | 703.13 | 2.76 | 2058 | 124.37 | 4.98 | 95 | 349.69 | 4.13 | 0.18 | A | 8.00 | 6.49 | 1.62 |
| 2012 | 14_24 | 8 | 14_23 | 7 | IE | L | 126 | 714.62 | 2.22 | 1997 | 127.99 | 4.67 | 106 | 281.52 | 3.23 | 0.18 | A | 8.00 | 1.60 | 0.40 |
| 2012 | 14_24 | 8 | 14_23 | 7 | IE | L | 171 | 712.45 | 2.58 | 1920 | 128.07 | 4.94 | 98 | 352.65 | 3.83 | 0.18 | A | 8.00 | 6.03 | 1.51 |
| 2012 | 14_24 | 8 | 14_23 | 7 | IE | L | 192 | 701.01 | 2.59 | 2463 | 125.70 | 4.97 | 75 | 348.72 | 4.98 | 0.18 | A | 8.00 | 6.19 | 1.55 |
| 2012 | 14_24 | 8 | 14_23 | 7 | IE | L | 179 | 707.97 | 2.28 | 1535 | 125.40 | 4.64 | 77 | 366.82 | 4.88 | 0.18 | A | 8.00 | 7.40 | 1.85 |
| 2012 | 14_24 | 8 | 14_23 | 7 | IE | L | 212 | 696.63 | 2.60 | 2770 | 124.03 | 4.96 | 70 | 390.42 | 5.90 | 0.18 | A | 8.00 | 9.18 | 2.30 |
| 2012 | 14_24 | 8 | 14_23 | 7 | IE | L | 150 | 703.23 | 2.94 | 2578 | 127.01 | 5.16 | 68 | 351.22 | 4.85 | 0.18 | A | 8.00 | 6.12 | 1.53 |
| 2012 | 14_24 | 8 | 14_23 | 7 | IE | L | 155 | 702.50 | 2.49 | 2187 | 125.72 | 4.88 | 99 | 345.32 | 3.52 | 0.18 | A | 8.00 | 5.97 | 1.49 |
| 2012 | 14_24 | 8 | 14_26 | 8 | IA | L | 111 | 718.81 | 3.05 | 1930 | 128.07 | 5.18 | 87 | 386.84 | 4.76 | 0.18 | A | 8.00 | 8.16 | 2.04 |
| 2012 | 14_24 | 8 | 14_26 | 8 | IA | L | 152 | 707.97 | 3.31 | 2291 | 126.23 | 4.99 | 42 | 369.21 | 3.78 | 0.18 | A | 8.00 | 7.40 | 1.85 |
| 2012 | 14_26 | 8 | 14_06 | 7 | IE | L | 123 | 707.74 | 2.92 | 2178 | 126.19 | 4.73 | 78 | 357.49 | 2.43 | 0.18 | A | 8.00 | 6.66 | 1.67 |
| 2012 | 14_26 | 8 | 14_13 | 7 | IE | L | 136 | 705.96 | 3.06 | 2101 | 125.49 | 4.83 | 92 | 339.29 | 3.98 | 0.18 | A | 8.00 | 5.63 | 1.41 |
| 2012 | 14_26 | 8 | 14_14 | 7 | IE | L | 108 | 698.40 | 2.93 | 3032 | 125.10 | 4.66 | 58 | 191.43 | 3.85 | 0.18 | S | 7.69 | 3.61 | 0.90 |
| 2012 | 14_26 | 8 | 14_24 | 8 | IA | L | 152 | 697.07 | 3.19 | 1809 | 125.12 | 4.41 | 35 | 379.79 | 3.07 | 0.18 | A | 8.00 | 8.28 | 2.07 |
| 2012 | 14_26 | 8 | 14_26 | 8 | S | L | 142 | 715.06 | 2.86 | 2656 | 128.06 | 5.18 | 101 | 366.91 | 4.21 | 0.18 | A | 8.00 | 6.92 | 1.73 |
| 2012 | 14_26 | 8 | 14_26 | 8 | S | L | 108 | 691.91 | 2.57 | 2718 | 122.24 | 4.43 | 81 | 356.32 | 5.98 | 0.18 | A | 8.00 | 7.32 | 1.83 |
| 2012 | 14_26 | 8 | 14_26 | 8 | S | L | 108 | 702.82 | 2.84 | 2407 | 123.83 | 4.82 | 63 | 355.69 | 3.71 | 0.18 | A | 8.00 | 6.98 | 1.74 |
| 2012 | 14_26 | 8 | 14_26 | 8 | S | L | 86 | 696.44 | 2.88 | 2185 | 124.72 | 4.80 | 93 | 363.18 | 3.71 | 0.18 | A | 8.00 | 7.30 | 1.82 |
| 2012 | 14_26 | 8 | 14_28 | 5 | IE | L | 205 | 701.51 | 2.89 | 2248 | 123.70 | 4.59 | 52 | 320.72 | 4.74 | 0.18 | A | 8.00 | 4.74 | 1.19 |
| 2012 | 14_26 | 8 | 14_28 | 5 | IE | L | 223 | 724.56 | 2.59 | 2008 | 127.83 | 5.41 | 55 | 334.39 | 2.73 | 0.18 | A | 8.00 | 4.93 | 1.23 |
| 2012 | 14_26 | 8 | 14_32 | 7 | IE | L | 124 | 708.90 | 2.75 | 2273 | 125.77 | 4.42 | 68 | 344.98 | 5.41 | 0.18 | A | 8.00 | 5.94 | 1.49 |
| 2012 | 14_26 | 8 | 14_32 | 7 | IE | L | 145 | 699.53 | 2.98 | 2064 | 123.27 | 4.52 | 91 | 334.83 | 4.18 | 0.18 | A | 8.00 | 5.73 | 1.43 |
| 2012 | 14_26 | 8 | 14_32 | 7 | IE | L | 122 | 702.00 | 2.88 | 2189 | 123.55 | 4.58 | 83 | 328.35 | 4.43 | 0.18 | A | 8.00 | 5.26 | 1.32 |
| 2012 | 14_26 | 8 | 14_32 | 7 | IE | L | 123 | 699.83 | 2.64 | 2168 | 123.54 | 4.53 | 55 | 344.84 | 3.76 | 0.18 | A | 8.00 | 6.33 | 1.58 |
| 2012 | 14_28 | 5 | 14_06 | 7 | IE | P | 629 | 596.55 | 3.35 | 1517 | 206.03 | 4.67 | 116 | 453.09 | 3.87 | 0.35 | AS | 9.59 |  |  |
| 2012 | 14_28 | 5 | 14_13 | 7 | IE | P | 389 | 599.38 | 2.98 | 1973 | 106.12 | 5.87 | 81 | 352.66 | 3.02 | 0.18 | A | 5.00 | 6.62 | 2.65 |
| 2012 | 14_28 | 5 | 14_28 | 5 | S | P | 456 | 601.31 | 2.25 | 2012 | 105.53 | 5.52 | 69 | 273.56 | 3.76 | 0.18 | A | 5.00 | 2.96 | 1.18 |
| 2012 | 14_32 | 7 | 14_21 | 5 | IE | P | 259 | 614.35 | 2.30 | 1598 | 160.03 | 7.59 | 43 | 443.97 | 2.92 | 0.26 | A | 7.00 | 5.42 | 1.55 |
| 2012 | 14_32 | 7 | 14_32 | 7 | S | P | 437 | 599.55 | 2.46 | 2364 | 222.48 | 4.75 | 139 | 376.83 | 4.34 | 0.37 | AF | 10.48 |  |  |
| 2012 | 14_32 | 7 | 14_32 | 7 | S | P | 347 | 600.14 | 2.46 | 1983 | 148.64 | 5.30 | 110 | 430.62 | 3.10 | 0.25 | A | 7.00 | 6.28 | 1.79 |
| 2012 | 15_04 | 5 | 15_04 | 5 | S | P | 166 | 613.13 | 2.05 | 2294 | 109.38 | 5.79 | 128 | 330.60 | 3.55 | 0.18 | A | 5.00 | 5.11 | 2.04 |
| 2012 | 15_06 | 5 | 15_41 | 6 | IE | P | 164 | 604.19 | 1.75 | 1463 | 109.03 | 5.33 | 92 | 340.91 | 3.41 | 0.18 | A | 5.00 | 5.63 | 2.25 |
| 2012 | 15_06 | 5 | 15_41 | 6 | IE | P | 225 | 610.93 | 2.10 | 1558 | 109.91 | 5.00 | 93 | 345.77 | 4.19 | 0.18 | A | 5.00 | 5.73 | 2.29 |
| 2012 | 15_06 | 5 | 15_41 | 6 | IE | P | 377 | 601.79 | 2.66 | 1802 | 108.81 | 5.24 | 107 | 343.69 | 3.01 | 0.18 | A | 5.00 | 5.79 | 2.32 |
| 2012 | 15_06 | 5 | 15_41 | 6 | IE | P | 301 | 595.31 | 2.00 | 1768 | 106.77 | 5.43 | 99 | 334.12 | 3.25 | 0.18 | A | 5.00 | 5.65 | 2.26 |
| 2012 | 15_20 | 5 | 15_20 | 5 | S | P | 395 | 604.02 | 2.50 | 1714 | 106.01 | 5.87 | 123 | 322.66 | 3.70 | 0.18 | A | 5.00 | 5.22 | 2.09 |
| 2012 | 15_20 | 5 | 15_20 | 5 | S | P | 323 | 606.15 | 1.69 | 2062 | 107.23 | 5.12 | 95 | 273.00 | 2.91 | 0.18 | A | 5.00 | 2.73 | 1.09 |
| 2012 | 15_20 | 5 | 15_20 | 5 | S | P | 284 | 607.03 | 2.78 | 1856 | 108.81 | 5.41 | 104 | 336.63 | 4.30 | 0.18 | A | 5.00 | 5.47 | 2.19 |
| 2012 | 15_20 | 5 | 15_20 | 5 | S | P | 458 | 596.92 | 2.25 | 1774 | 106.75 | 4.83 | 102 | 311.13 | 2.81 | 0.18 | A | 5.00 | 4.57 | 1.83 |
| 2012 | 15_20 | 5 | 15_20 | 5 | S | P | 409 | 604.46 | 1.74 | 1288 | 106.95 | 5.06 | 104 | 331.94 | 3.90 | 0.18 | A | 5.00 | 5.52 | 2.21 |
| 2012 | 15_20 | 5 | 15_20 | 5 | S | P | 244 | 591.64 | 2.05 | 1809 | 103.44 | 5.26 | 135 | 288.82 | 3.82 | 0.17 | A | 5.00 | 3.96 | 1.58 |
| 2012 | 15_20 | 5 | 15_20 | 5 | S | P | 328 | 595.65 | 2.09 | 1559 | 104.62 | 4.72 | 117 | 320.81 | 3.34 | 0.18 | A | 5.00 | 5.33 | 2.13 |
| 2012 | 15_20 | 5 | 15_20 | 5 | S | P | 432 | 606.07 | 1.84 | 1762 | 106.79 | 4.86 | 125 | 314.23 | 3.73 | 0.18 | A | 5.00 | 4.71 | 1.89 |
| 2012 | 15_23 | 6 | 15_06 | 5 | IE | P | 327 | 597.09 | 2.25 | 2102 | 128.94 | 4.66 | 74 | 387.12 | 3.05 | 0.22 | A | 6.00 | 6.01 | 2.00 |
| 2012 | 15_23 | 6 | 15_06 | 5 | IE | P | 318 | 595.08 | 2.19 | 2023 | 128.15 | 4.19 | 78 | 382.14 | 2.92 | 0.22 | A | 6.00 | 5.89 | 1.96 |
| 2012 | 15_23 | 6 | 15_06 | 5 | IE | P | 309 | 595.55 | 2.24 | 2085 | 124.47 | 4.39 | 94 | 378.69 | 3.17 | 0.21 | A | 6.00 | 6.25 | 2.08 |
| 2012 | 15_23 | 6 | 15_06 | 5 | IE | P | 387 | 594.36 | 2.86 | 2167 | 127.08 | 4.65 | 97 | 381.75 | 4.37 | 0.21 | A | 6.00 | 6.02 | 2.01 |
| 2012 | 15_23 | 6 | 15_06 | 5 | IE | P | 502 | 595.15 | 3.30 | 1328 | 129.95 | 5.08 | 50 | 333.70 | 3.68 | 0.22 | A | 6.00 | 3.41 | 1.14 |
| 2012 | 15_23 | 6 | 15_06 | 5 | IE | P | 348 | 586.63 | 2.15 | 2069 | 125.89 | 4.41 | 65 | 378.42 | 3.88 | 0.21 | A | 6.00 | 6.04 | 2.01 |
| 2012 | 15_23 | 6 | 15_06 | 5 | IE | P | 384 | 596.32 | 2.30 | 2100 | 129.93 | 4.55 | 92 | 385.18 | 2.70 | 0.22 | A | 6.00 | 5.79 | 1.93 |
| 2012 | 15_23 | 6 | 15_06 | 5 | IE | P | 294 | 600.64 | 2.50 | 2198 | 128.66 | 4.22 | 62 | 386.12 | 2.73 | 0.21 | A | 6.00 | 6.01 | 2.00 |
| 2012 | 15_23 | 6 | 15_06 | 5 | IE | P | 463 | 591.07 | 2.65 | 1914 | 126.71 | 5.14 | 63 | 329.78 | 4.17 | 0.21 | A | 6.00 | 3.62 | 1.21 |
| 2012 | 15_23 | 6 | 15_06 | 5 | IE | P | 255 | 613.15 | 2.25 | 2192 | 130.59 | 4.47 | 58 | 337.47 | 3.34 | 0.21 | A | 6.00 | 3.51 | 1.17 |
| 2012 | 15_23 | 6 | 15_23 | 6 | S | P | 472 | 621.04 | 4.53 | 1649 | 133.11 | 5.90 | 79 | 410.45 | 3.08 | 0.21 | A | 6.00 | 6.50 | 2.17 |
| 2012 | 15_23 | 6 | 15_23 | 6 | S | P | 384 | 609.07 | 3.05 | 1731 | 130.22 | 5.50 | 47 | 513.90 | 2.94 | 0.21 | A | 6.00 | 11.68 | 3.89 |
| 2012 | 15_23 | 6 | 15_23 | 6 | S | P | 259 | 606.49 | 2.56 | 1993 | 130.45 | 4.92 | 73 | 387.66 | 3.07 | 0.22 | A | 6.00 | 5.83 | 1.94 |
| 2012 | 15_23 | 6 | 15_23 | 6 | S | P | 340 | 607.60 | 3.02 | 1842 | 130.49 | 4.99 | 83 | 393.21 | 3.85 | 0.21 | A | 6.00 | 6.08 | 2.03 |
| 2012 | 15_23 | 6 | 15_37 | 5 | IE | P | 405 | 606.52 | 2.82 | 2500 | 129.87 | 4.60 | 57 | 348.16 | 3.42 | 0.21 | A | 6.00 | 4.09 | 1.36 |
| 2012 | 15_23 | 6 | 15_37 | 5 | IE | P | 422 | 598.30 | 3.31 | 2239 | 129.60 | 4.13 | 88 | 383.12 | 3.86 | 0.22 | A | 6.00 | 5.74 | 1.91 |
| 2012 | 15_23 | 6 | 15_37 | 5 | IE | P | 336 | 599.84 | 2.05 | 1951 | 128.08 | 4.69 | 80 | 387.02 | 2.77 | 0.21 | A | 6.00 | 6.13 | 2.04 |
| 2012 | 15_23 | 6 | 15_37 | 5 | IE | P | 370 | 587.78 | 2.62 | 2058 | 125.64 | 4.89 | 111 | 343.27 | 3.79 | 0.21 | A | 6.00 | 4.39 | 1.46 |
| 2012 | 15_23 | 6 | 15_37 | 5 | IE | P | 422 | 594.97 | 2.36 | 2103 | 129.10 | 4.49 | 62 | 339.59 | 3.16 | 0.22 | A | 6.00 | 3.78 | 1.26 |
| 2012 | 15_23 | 6 | 15_37 | 5 | IE | P | 312 | 602.88 | 2.53 | 2107 | 130.64 | 4.88 | 83 | 372.88 | 4.25 | 0.22 | A | 6.00 | 5.13 | 1.71 |
| 2012 | 15_23 | 6 | 15_37 | 5 | IE | P | 326 | 601.41 | 2.18 | 1760 | 132.22 | 4.35 | 86 | 392.45 | 2.52 | 0.22 | A | 6.00 | 5.81 | 1.94 |
| 2012 | 15_23 | 6 | 15_37 | 5 | IE | P | 326 | 601.35 | 2.28 | 2095 | 132.53 | 4.69 | 135 | 399.23 | 3.72 | 0.22 | A | 6.00 | 6.07 | 2.02 |
| 2012 | 15_23 | 6 | 15_37 | 5 | IE | P | 296 | 606.03 | 2.34 | 1772 | 135.01 | 5.03 | 55 | 409.06 | 2.98 | 0.22 | A | 6.00 | 6.18 | 2.06 |
| 2012 | 15_23 | 6 | 15_37 | 5 | IE | P | 275 | 590.96 | 4.04 | 2279 | 132.75 | 4.91 | 80 | 394.11 | 3.45 | 0.22 | A | 6.00 | 5.81 | 1.94 |
| 2012 | 15_29 | 5 | 15_41 | 6 | IE | P | 199 | 599.85 | 2.29 | 2303 | 107.93 | 4.82 | 120 | 338.13 | 3.02 | 0.18 | A | 5.00 | 5.66 | 2.27 |
| 2012 | 15_29 | 5 | 15_41 | 6 | IE | P | 258 | 597.57 | 1.83 | 2040 | 106.85 | 4.93 | 112 | 337.79 | 3.04 | 0.18 | A | 5.00 | 5.81 | 2.32 |
| 2012 | 15_29 | 5 | 15_41 | 6 | IE | P | 159 | 596.11 | 2.10 | 2102 | 107.60 | 5.03 | 101 | 281.85 | 3.40 | 0.18 | A | 5.00 | 3.10 | 1.24 |
| 2012 | 15_29 | 5 | 15_41 | 6 | IE | P | 174 | 595.13 | 2.20 | 2100 | 107.18 | 5.19 | 120 | 336.51 | 2.90 | 0.18 | A | 5.00 | 5.70 | 2.28 |
| 2012 | 15_29 | 5 | 15_41 | 6 | IE | P | 132 | 592.20 | 1.80 | 1584 | 109.68 | 5.38 | 84 | 287.66 | 3.54 | 0.19 | A | 5.00 | 3.11 | 1.25 |
| 2012 | 15_29 | 5 | 15_41 | 6 | IE | P | 197 | 590.60 | 1.93 | 2011 | 106.48 | 5.20 | 91 | 333.17 | 2.93 | 0.18 | A | 5.00 | 5.64 | 2.26 |
| 2012 | 15_29 | 5 | 15_41 | 6 | IE | P | 208 | 597.10 | 1.90 | 2136 | 106.78 | 5.20 | 99 | 333.64 | 3.83 | 0.18 | A | 5.00 | 5.62 | 2.25 |
| 2012 | 15_29 | 5 | 15_41 | 6 | IE | P | 140 | 599.33 | 2.05 | 2167 | 108.67 | 4.98 | 102 | 285.01 | 3.55 | 0.18 | A | 5.00 | 3.11 | 1.25 |
| 2012 | 15_29 | 5 | 15_41 | 6 | IE | P | 295 | 591.46 | 2.24 | 2107 | 105.94 | 5.38 | 106 | 340.57 | 3.62 | 0.18 | A | 5.00 | 6.07 | 2.43 |
| 2012 | 15_29 | 5 | 15_41 | 6 | IE | P | 370 | 599.33 | 1.75 | 2009 | 108.48 | 5.24 | 108 | 295.16 | 3.62 | 0.18 | A | 5.00 | 3.60 | 1.44 |
| 2012 | 15_29 | 5 | 15_46 | 6 | IE | P | 178 | 603.61 | 1.41 | 2221 | 107.52 | 4.84 | 85 | 342.44 | 3.81 | 0.18 | A | 5.00 | 5.92 | 2.37 |
| 2012 | 15_29 | 5 | 15_46 | 6 | IE | P | 238 | 598.92 | 1.79 | 2247 | 106.90 | 4.88 | 84 | 284.48 | 2.88 | 0.18 | A | 5.00 | 3.31 | 1.32 |
| 2012 | 15_29 | 5 | 15_46 | 6 | IE | P | 175 | 602.39 | 2.14 | 2253 | 107.63 | 4.87 | 104 | 343.56 | 3.38 | 0.18 | A | 5.00 | 5.96 | 2.38 |
| 2012 | 15_29 | 5 | 15_46 | 6 | IE | P | 180 | 597.51 | 2.15 | 2141 | 108.52 | 4.61 | 90 | 289.33 | 3.55 | 0.18 | A | 5.00 | 3.33 | 1.33 |
| 2012 | 15_29 | 5 | 15_46 | 6 | IE | P | 214 | 596.18 | 1.82 | 2298 | 106.59 | 4.66 | 97 | 282.79 | 3.40 | 0.18 | A | 5.00 | 3.27 | 1.31 |
| 2012 | 15_29 | 5 | 15_46 | 6 | IE | P | 149 | 596.84 | 2.13 | 2449 | 109.40 | 5.24 | 114 | 337.63 | 3.17 | 0.18 | A | 5.00 | 5.43 | 2.17 |
| 2012 | 15_29 | 5 | 15_46 | 6 | IE | P | 207 | 595.84 | 1.84 | 2049 | 166.23 | 3.70 | 101 | 280.96 | 3.51 | 0.28 | AF | 7.75 |  |  |
| 2012 | 15_29 | 5 | 15_46 | 6 | IE | P | 196 | 596.79 | 1.95 | 2125 | 107.85 | 4.77 | 98 | 284.05 | 3.66 | 0.18 | A | 5.00 | 3.17 | 1.27 |
| 2012 | 15_29 | 5 | 15_46 | 6 | IE | P | 232 | 593.45 | 1.59 | 2017 | 168.81 | 3.95 | 100 | 284.84 | 3.09 | 0.28 | AF | 7.90 |  |  |
| 2012 | 15_29 | 5 | 15_46 | 6 | IE | P | 205 | 600.68 | 2.12 | 2226 | 108.62 | 4.98 | 92 | 344.39 | 3.17 | 0.18 | A | 5.00 | 5.85 | 2.34 |
| 2012 | 15_33 | 6 | 15_04 | 5 | IE | P | 199 | 613.68 | 2.73 | 2063 | 135.61 | 4.70 | 129 | 386.19 | 3.34 | 0.22 | A | 6.00 | 5.09 | 1.70 |
| 2012 | 15_33 | 6 | 15_33 | 6 | S | P | 197 | 602.86 | 2.97 | 1854 | 127.76 | 5.44 | 165 | 383.25 | 3.63 | 0.21 | A | 6.00 | 6.00 | 2.00 |
| 2012 | 15_33 | 6 | 15_37 | 5 | IE | P | 358 | 607.96 | 2.43 | 1608 | 129.96 | 6.46 | 119 | 377.80 | 3.81 | 0.21 | A | 6.00 | 5.44 | 1.81 |
| 2012 | 15_33 | 6 | 15_41 | 6 | IA | P | 287 | 592.69 | 2.49 | 1592 | 188.92 | 4.21 | 136 | 319.70 | 4.38 | 0.32 | AF | 8.93 |  |  |
| 2012 | 15_33 | 6 | 15_41 | 6 | IA | P | 310 | 597.16 | 2.40 | 1870 | 127.27 | 5.10 | 77 | 375.00 | 3.27 | 0.21 | A | 6.00 | 5.68 | 1.89 |
| 2012 | 15_33 | 6 | 15_41 | 6 | IA | P | 217 | 600.98 | 1.90 | 2119 | 127.83 | 4.94 | 133 | 382.66 | 3.65 | 0.21 | A | 6.00 | 5.96 | 1.99 |
| 2012 | 15_33 | 6 | 15_41 | 6 | IA | P | 267 | 594.05 | 2.29 | 1809 | 126.38 | 4.87 | 136 | 322.85 | 3.79 | 0.21 | A | 6.00 | 3.33 | 1.11 |
| 2012 | 15_33 | 6 | 15_41 | 6 | IA | P | 304 | 602.44 | 2.07 | 1706 | 128.54 | 4.78 | 117 | 324.66 | 3.23 | 0.21 | A | 6.00 | 3.15 | 1.05 |
| 2012 | 15_33 | 6 | 15_41 | 6 | IA | P | 299 | 601.25 | 2.15 | 1819 | 128.40 | 4.69 | 107 | 326.06 | 3.43 | 0.21 | A | 6.00 | 3.24 | 1.08 |
| 2012 | 15_33 | 6 | 15_41 | 6 | IA | P | 301 | 592.13 | 2.15 | 1643 | 127.41 | 5.76 | 114 | 189.30 | 4.94 | 0.22 | S | 6.03 | 3.10 | 1.03 |
| 2012 | 15_33 | 6 | 15_41 | 6 | IA | P | 385 | 599.52 | 2.17 | 1814 | 127.95 | 5.05 | 138 | 385.92 | 3.81 | 0.21 | A | 6.00 | 6.10 | 2.03 |
| 2012 | 15_33 | 6 | 15_41 | 6 | IA | P | 334 | 594.11 | 1.91 | 1825 | 128.22 | 5.76 | 120 | 381.09 | 3.74 | 0.22 | A | 6.00 | 5.83 | 1.94 |
| 2012 | 15_33 | 6 | 15_41 | 6 | IA | P | 378 | 592.51 | 2.16 | 1823 | 126.31 | 4.95 | 119 | 322.13 | 4.52 | 0.21 | A | 6.00 | 3.30 | 1.10 |
| 2012 | 15_37 | 5 | 15_46 | 6 | IE | P | 312 | 598.59 | 1.85 | 2087 | 106.01 | 5.10 | 97 | 321.04 | 2.97 | 0.18 | A | 5.00 | 5.14 | 2.06 |
| 2012 | 15_41 | 6 | 15_06 | 5 | IE | P | 216 | 599.77 | 1.98 | 2167 | 127.85 | 4.73 | 88 | 371.54 | 3.78 | 0.21 | A | 6.00 | 5.44 | 1.81 |
| 2012 | 15_41 | 6 | 15_17 | 6 | IA | P | 324 | 608.71 | 3.20 | 1705 | 130.04 | 6.12 | 132 | 388.51 | 5.45 | 0.21 | A | 6.00 | 5.93 | 1.98 |
| 2012 | 15_41 | 6 | 15_20 | 5 | IE | P | 205 | 602.06 | 2.11 | 2152 | 127.92 | 5.27 | 87 | 372.09 | 3.09 | 0.21 | A | 6.00 | 5.45 | 1.82 |
| 2012 | 15_41 | 6 | 15_33 | 6 | IA | P | 274 | 604.74 | 1.62 | 1813 | 128.72 | 4.51 | 113 | 377.09 | 3.50 | 0.21 | A | 6.00 | 5.58 | 1.86 |
| 2012 | 15_41 | 6 | 15_33 | 6 | IA | P | 302 | 592.73 | 3.77 | 1882 | 95.77 | 7.00 | 169 | 345.98 | 4.57 | 0.16 | HP | 4.52 |  |  |
| 2012 | 15_41 | 6 | 15_41 | 6 | S | P | 174 | 603.45 | 2.13 | 2183 | 132.13 | 5.64 | 90 | 392.93 | 3.48 | 0.22 | A | 6.00 | 5.84 | 1.95 |
| 2012 | 15_41 | 6 | 15_41 | 6 | S | P | 169 | 600.67 | 2.82 | 2135 | 130.05 | 5.61 | 82 | 378.39 | 3.28 | 0.22 | A | 6.00 | 5.46 | 1.82 |
| 2012 | 15_41 | 6 | 15_41 | 6 | S | P | 127 | 605.96 | 2.43 | 2315 | 130.13 | 5.62 | 57 | 393.75 | 2.51 | 0.21 | A | 6.00 | 6.15 | 2.05 |
| 2012 | 15_41 | 6 | 15_41 | 6 | S | P | 182 | 607.04 | 2.28 | 1616 | 130.68 | 4.87 | 67 | 398.19 | 4.20 | 0.22 | A | 6.00 | 6.28 | 2.09 |
| 2012 | 15_41 | 6 | 15_46 | 6 | IA | P | 350 | 593.82 | 2.31 | 1938 | 126.96 | 5.05 | 145 | 375.14 | 3.80 | 0.21 | A | 6.00 | 5.73 | 1.91 |
| 2012 | 15_46 | 6 | 15_04 | 5 | IE | P | 301 | 608.77 | 1.68 | 2084 | 130.01 | 5.29 | 98 | 383.51 | 3.57 | 0.21 | A | 6.00 | 5.70 | 1.90 |
| 2012 | 15_46 | 6 | 15_04 | 5 | IE | P | 278 | 597.35 | 2.04 | 2145 | 127.22 | 4.88 | 105 | 370.87 | 3.46 | 0.21 | A | 6.00 | 5.49 | 1.83 |
| 2012 | 15_46 | 6 | 15_17 | 6 | IA | P | 403 | 603.42 | 2.24 | 1830 | 129.72 | 4.79 | 98 | 375.50 | 7.21 | 0.21 | A | 6.00 | 5.37 | 1.79 |
| 2012 | 15_46 | 6 | 15_17 | 6 | IA | P | 372 | 594.61 | 2.79 | 1722 | 125.71 | 5.61 | 111 | 374.35 | 4.19 | 0.21 | A | 6.00 | 5.87 | 1.96 |
| 2012 | 15_46 | 6 | 15_41 | 6 | IA | P | 509 | 608.83 | 2.27 | 1785 | 135.61 | 7.23 | 75 | 521.79 | 3.09 | 0.22 | A | 6.00 | 11.09 | 3.70 |
| 2012 | 17_04 | 5 | 17_04 | 5 | S | P | 553 | 595.97 | 2.43 | 2059 | 104.53 | 5.74 | 105 | 266.86 | 4.02 | 0.18 | A | 5.00 | 2.76 | 1.11 |
| 2012 | 17_04 | 5 | 17_15 | 8 | IE | P | 171 | 605.33 | 2.16 | 2468 | 107.07 | 4.99 | 48 | 472.55 | 4.17 | 0.18 | A | 5.00 | 12.07 | 4.83 |
| 2012 | 17_05 | 5 | 17_04 | 5 | IA | P | 309 | 599.29 | 3.49 | 2280 | 106.63 | 5.82 | 97 | 318.91 | 3.40 | 0.18 | A | 5.00 | 4.95 | 1.98 |
| 2012 | 17_05 | 5 | 17_14 | 8 | IE | P | 260 | 599.23 | 2.13 | 1849 | 171.77 | 4.32 | 160 | 272.75 | 3.76 | 0.29 | AF | 7.96 |  |  |
| 2012 | 17_05 | 5 | 17_14 | 8 | IE | P | 298 | 602.74 | 2.51 | 2097 | 173.42 | 3.90 | 202 | 273.63 | 3.79 | 0.29 | AF | 7.99 |  |  |
| 2012 | 17_05 | 5 | 17_15 | 8 | IE | P | 216 | 606.66 | 3.49 | 1984 | 113.24 | 5.25 | 110 | 363.28 | 3.10 | 0.19 | A | 5.00 | 6.04 | 2.42 |
| 2012 | 17_05 | 5 | 17_15 | 8 | IE | P | 212 | 606.37 | 3.01 | 1964 | 175.79 | 4.22 | 125 | 287.15 | 3.72 | 0.29 | AF | 8.05 |  |  |
| 2012 | 17_05 | 5 | 17_16 | 8 | IE | P | 290 | 605.64 | 3.21 | 1804 | 193.93 | 4.23 | 119 | 303.24 | 3.52 | 0.32 | AF | 8.89 |  |  |
| 2012 | 17_05 | 5 | 17_16 | 8 | IE | P | 262 | 619.10 | 2.52 | 1446 | 195.80 | 4.52 | 268 | 295.26 | 3.69 | 0.32 | AF | 8.78 |  |  |
| 2012 | 17_05 | 5 | 17_17 | 8 | IE | P | 344 | 602.54 | 2.80 | 1843 | 187.49 | 3.71 | 104 | 300.95 | 3.67 | 0.31 | AF | 8.64 |  |  |
| 2012 | 17_07 | 5 | 17_07 | 5 | S | P | 220 | 611.83 | 2.75 | 2113 | 115.17 | 5.31 | 101 | 358.20 | 4.05 | 0.19 | A | 5.00 | 5.55 | 2.22 |
| 2012 | 17_07 | 5 | 17_16 | 8 | IE | P | 240 | 610.87 | 3.14 | 2420 | 114.69 | 5.65 | 107 | 312.15 | 3.91 | 0.19 | A | 5.00 | 3.61 | 1.44 |
| 2012 | 17_07 | 5 | 17_17 | 8 | IE | P | 203 | 606.56 | 2.99 | 2059 | 110.94 | 5.64 | 68 | 376.99 | 4.13 | 0.18 | A | 5.00 | 6.99 | 2.80 |
| 2012 | 17_08 | 5 | 17_09 | 5 | IA | P | 470 | 613.80 | 3.89 | 1578 | 208.06 | 5.46 | 112 | 318.73 | 5.34 | 0.34 | AF | 9.41 |  |  |
| 2012 | 17_08 | 5 | 17_15 | 8 | IE | P | 154 | 602.84 | 2.35 | 2289 | 104.13 | 5.34 | 85 | 387.99 | 3.39 | 0.17 | A | 5.00 | 8.63 | 3.45 |
| 2012 | 17_08 | 5 | 17_16 | 8 | IE | P | 304 | 602.62 | 2.28 | 1849 | 109.94 | 5.11 | 95 | 389.25 | 3.14 | 0.18 | A | 5.00 | 7.70 | 3.08 |
| 2012 | 17_08 | 5 | 17_17 | 8 | IE | P | 268 | 604.35 | 2.60 | 2019 | 108.11 | 4.97 | 97 | 309.10 | 4.33 | 0.18 | A | 5.00 | 4.30 | 1.72 |
| 2012 | 17_08 | 5 | 17_19 | 8 | IE | P | 347 | 597.35 | 2.70 | 1739 | 105.63 | 6.06 | 92 | 349.03 | 3.55 | 0.18 | A | 5.00 | 6.52 | 2.61 |
| 2012 | 17_08 | 5 | 17_19 | 8 | IE | P | 273 | 601.83 | 2.79 | 2006 | 106.17 | 6.53 | 70 | 380.63 | 3.60 | 0.18 | A | 5.00 | 7.93 | 3.17 |
| 2012 | 17_09 | 5 | 17_14 | 8 | IE | P | 200 | 602.43 | 3.73 | 1452 | 107.44 | 6.57 | 124 | 289.89 | 5.47 | 0.18 | A | 5.00 | 3.49 | 1.40 |
| 2012 | 17_09 | 5 | 17_14 | 8 | IE | P | 277 | 601.11 | 4.30 | 1746 | 108.58 | 6.74 | 97 | 378.57 | 4.32 | 0.18 | A | 5.00 | 7.43 | 2.97 |
| 2012 | 17_09 | 5 | 17_16 | 8 | IE | P | 910 | 629.81 | 4.13 | 1457 | 118.89 | 4.95 | 100 | 379.25 | 4.21 | 0.19 | A | 5.00 | 5.95 | 2.38 |
| 2012 | 17_09 | 5 | 17_16 | 8 | IE | P | 904 | 595.26 | 2.58 | 1253 | 106.32 | 4.54 | 98 | 299.44 | 3.80 | 0.18 | A | 5.00 | 4.08 | 1.63 |
| 2012 | 17_09 | 5 | 17_16 | 8 | IE | P | 947 | 605.99 | 2.18 | 1562 | 108.32 | 4.59 | 124 | 362.07 | 5.10 | 0.18 | A | 5.00 | 6.71 | 2.69 |
| 2012 | 17_09 | 5 | 17_16 | 8 | IE | P | 988 | 611.10 | 2.45 | 1391 | 109.80 | 4.96 | 111 | 368.66 | 3.82 | 0.18 | A | 5.00 | 6.79 | 2.72 |
| 2012 | 17_09 | 5 | 17_16 | 8 | IE | P | 865 | 601.01 | 2.69 | 1408 | 107.51 | 4.54 | 96 | 346.57 | 5.96 | 0.18 | A | 5.00 | 6.12 | 2.45 |
| 2012 | 17_09 | 5 | 17_16 | 8 | IE | P | 894 | 603.02 | 2.41 | 1201 | 105.83 | 4.27 | 99 | 298.92 | 4.61 | 0.18 | A | 5.00 | 4.12 | 1.65 |
| 2012 | 17_09 | 5 | 17_16 | 8 | IE | P | 816 | 601.37 | 2.19 | 1460 | 107.59 | 4.40 | 75 | 359.99 | 3.08 | 0.18 | A | 5.00 | 6.73 | 2.69 |
| 2012 | 17_09 | 5 | 17_16 | 8 | IE | P | 764 | 605.60 | 2.14 | 1620 | 109.26 | 4.97 | 81 | 289.66 | 4.16 | 0.18 | A | 5.00 | 3.26 | 1.30 |
| 2012 | 17_09 | 5 | 17_16 | 8 | IE | P | 901 | 616.96 | 2.00 | 1384 | 112.43 | 5.44 | 83 | 372.70 | 3.29 | 0.18 | A | 5.00 | 6.57 | 2.63 |
| 2012 | 17_09 | 5 | 17_16 | 8 | IE | P | 819 | 616.05 | 2.66 | 1375 | 112.57 | 5.17 | 75 | 305.04 | 3.55 | 0.18 | A | 5.00 | 3.55 | 1.42 |
| 2012 | 17_09 | 5 | 17_17 | 8 | IE | P | 1134 | 615.86 | 3.95 | 1341 | 115.16 | 4.15 | 107 | 388.75 | 4.04 | 0.19 | A | 5.00 | 6.88 | 2.75 |
| 2012 | 17_09 | 5 | 17_17 | 8 | IE | P | 681 | 606.03 | 2.08 | 1491 | 108.32 | 4.13 | 96 | 301.07 | 4.33 | 0.18 | A | 5.00 | 3.90 | 1.56 |
| 2012 | 17_09 | 5 | 17_17 | 8 | IE | P | 823 | 602.74 | 2.95 | 1325 | 110.00 | 4.44 | 99 | 361.40 | 4.18 | 0.18 | A | 5.00 | 6.43 | 2.57 |
| 2012 | 17_09 | 5 | 17_17 | 8 | IE | P | 895 | 618.42 | 2.87 | 1358 | 111.84 | 4.46 | 109 | 364.97 | 4.56 | 0.18 | A | 5.00 | 6.32 | 2.53 |
| 2012 | 17_09 | 5 | 17_17 | 8 | IE | P | 751 | 598.11 | 2.90 | 1415 | 109.23 | 5.34 | 151 | 368.68 | 6.51 | 0.18 | A | 5.00 | 6.88 | 2.75 |
| 2012 | 17_09 | 5 | 17_17 | 8 | IE | P | 866 | 612.40 | 2.33 | 1302 | 110.36 | 4.60 | 115 | 390.72 | 3.81 | 0.18 | A | 5.00 | 7.70 | 3.08 |
| 2012 | 17_09 | 5 | 17_17 | 8 | IE | P | 820 | 590.63 | 3.14 | 1538 | 107.23 | 4.28 | 132 | 367.10 | 5.19 | 0.18 | A | 5.00 | 7.12 | 2.85 |
| 2012 | 17_09 | 5 | 17_17 | 8 | IE | P | 933 | 591.65 | 4.91 | 1041 | 108.80 | 5.62 | 109 | 337.45 | 6.44 | 0.18 | A | 5.00 | 5.51 | 2.20 |
| 2012 | 17_09 | 5 | 17_17 | 8 | IE | P | 1060 | 594.13 | 3.40 | 1409 | 108.58 | 4.70 | 148 | 372.31 | 5.66 | 0.18 | A | 5.00 | 7.14 | 2.86 |
| 2012 | 17_09 | 5 | 17_17 | 8 | IE | P | 927 | 609.23 | 2.68 | 1293 | 109.46 | 4.24 | 76 | 353.28 | 4.57 | 0.18 | A | 5.00 | 6.14 | 2.45 |
| 2012 | 17_14 | 8 | 17_04 | 5 | IE | L | 96 | 707.27 | 3.09 | 1911 | 135.10 | 5.20 | 71 | 392.57 | 3.32 | 0.19 | A | 8.00 | 7.25 | 1.81 |
| 2012 | 17_14 | 8 | 17_08 | 5 | IE | L | 131 | 689.77 | 2.94 | 1712 | 123.57 | 4.58 | 87 | 330.58 | 3.55 | 0.18 | A | 8.00 | 5.40 | 1.35 |
| 2012 | 17_14 | 8 | 17_14 | 8 | S | L | 96 | 714.96 | 2.30 | 2003 | 127.83 | 4.58 | 38 | 379.64 | 3.56 | 0.18 | A | 8.00 | 7.76 | 1.94 |
| 2012 | 17_14 | 8 | 17_14 | 8 | S | L | 97 | 715.31 | 2.67 | 2289 | 125.58 | 5.04 | 50 | 367.28 | 2.95 | 0.18 | A | 8.00 | 7.40 | 1.85 |
| 2012 | 17_14 | 8 | 17_15 | 8 | IA | L | 147 | 700.74 | 3.07 | 1882 | 123.56 | 5.33 | 40 | 366.92 | 3.86 | 0.18 | A | 8.00 | 7.76 | 1.94 |
| 2012 | 17_14 | 8 | 17_15 | 8 | IA | L | 221 | 717.85 | 2.92 | 2027 | 125.88 | 4.87 | 58 | 370.75 | 3.82 | 0.18 | A | 8.00 | 7.56 | 1.89 |
| 2012 | 17_14 | 8 | 17_19 | 8 | IA | L | 255 | 700.60 | 3.61 | 1925 | 123.51 | 5.33 | 41 | 361.12 | 3.82 | 0.18 | A | 8.00 | 7.39 | 1.85 |
| 2012 | 17_14 | 8 | 17_19 | 8 | IA | L | 175 | 696.67 | 4.15 | 1682 | 123.77 | 5.84 | 52 | 370.08 | 4.96 | 0.18 | A | 8.00 | 7.92 | 1.98 |
| 2012 | 17_15 | 8 | 17_09 | 5 | IE | L | 92 | 712.79 | 2.82 | 1833 | 125.55 | 4.96 | 68 | 368.57 | 3.06 | 0.18 | A | 8.00 | 7.49 | 1.87 |
| 2012 | 17_15 | 8 | 17_09 | 5 | IE | L | 112 | 714.84 | 2.42 | 1948 | 123.50 | 5.08 | 82 | 371.46 | 4.84 | 0.17 | A | 8.00 | 8.06 | 2.02 |
| 2012 | 17_15 | 8 | 17_15 | 8 | S | L | 131 | 711.77 | 2.82 | 1893 | 127.60 | 4.80 | 64 | 373.16 | 3.91 | 0.18 | A | 8.00 | 7.40 | 1.85 |
| 2012 | 17_15 | 8 | 17_15 | 8 | S | L | 213 | 707.84 | 2.98 | 1984 | 124.49 | 4.54 | 80 | 363.21 | 3.97 | 0.18 | A | 8.00 | 7.34 | 1.84 |
| 2012 | 17_15 | 8 | 17_15 | 8 | S | L | 162 | 707.46 | 3.22 | 2038 | 125.60 | 4.52 | 51 | 367.88 | 4.16 | 0.18 | A | 8.00 | 7.43 | 1.86 |
| 2012 | 17_15 | 8 | 17_15 | 8 | S | L | 190 | 701.39 | 3.13 | 2003 | 123.72 | 4.85 | 59 | 356.89 | 4.30 | 0.18 | A | 8.00 | 7.08 | 1.77 |
| 2012 | 17_15 | 8 | 17_15 | 8 | S | L | 113 | 699.88 | 3.54 | 1881 | 124.38 | 4.80 | 53 | 377.15 | 3.45 | 0.18 | A | 8.00 | 8.26 | 2.06 |
| 2012 | 17_15 | 8 | 17_15 | 8 | S | L | 183 | 705.39 | 3.11 | 2121 | 124.32 | 5.18 | 60 | 369.63 | 6.10 | 0.18 | A | 8.00 | 7.79 | 1.95 |
| 2012 | 17_15 | 8 | 17_19 | 8 | IA | L | 137 | 710.43 | 2.90 | 1727 | 125.95 | 4.97 | 83 | 356.57 | 5.03 | 0.18 | A | 8.00 | 6.65 | 1.66 |
| 2012 | 17_16 | 8 | 17_04 | 5 | IE | L | 155 | 722.37 | 2.83 | 1938 | 127.22 | 4.73 | 87 | 387.14 | 3.73 | 0.18 | A | 8.00 | 8.34 | 2.09 |
| 2012 | 17_16 | 8 | 17_04 | 5 | IE | L | 154 | 702.56 | 2.09 | 1910 | 128.40 | 5.01 | 95 | 372.26 | 3.72 | 0.18 | A | 8.00 | 7.19 | 1.80 |
| 2012 | 17_16 | 8 | 17_04 | 5 | IE | L | 145 | 706.02 | 3.14 | 2092 | 132.25 | 5.31 | 73 | 380.90 | 3.30 | 0.19 | A | 8.00 | 7.04 | 1.76 |
| 2012 | 17_16 | 8 | 17_04 | 5 | IE | L | 159 | 693.16 | 2.84 | 2392 | 128.06 | 5.68 | 61 | 502.92 | 4.81 | 0.18 | A | 8.00 | 15.42 | 3.85 |
| 2012 | 17_16 | 8 | 17_04 | 5 | IE | L | 133 | 681.91 | 2.89 | 2465 | 128.50 | 5.47 | 80 | 379.94 | 4.07 | 0.19 | A | 8.00 | 7.65 | 1.91 |
| 2012 | 17_16 | 8 | 17_04 | 5 | IE | L | 154 | 712.32 | 2.71 | 1971 | 127.99 | 5.20 | 70 | 378.28 | 3.14 | 0.18 | A | 8.00 | 7.64 | 1.91 |
| 2012 | 17_16 | 8 | 17_04 | 5 | IE | L | 190 | 706.36 | 4.48 | 2081 | 124.25 | 6.41 | 69 | 378.50 | 4.67 | 0.18 | A | 8.00 | 8.37 | 2.09 |
| 2012 | 17_16 | 8 | 17_04 | 5 | IE | L | 127 | 695.55 | 3.22 | 1967 | 123.50 | 5.67 | 108 | 379.27 | 4.86 | 0.18 | A | 8.00 | 8.57 | 2.14 |
| 2012 | 17_16 | 8 | 17_04 | 5 | IE | L | 155 | 678.82 | 4.58 | 1939 | 120.33 | 6.40 | 42 | 360.78 | 3.33 | 0.18 | A | 8.00 | 7.99 | 2.00 |
| 2012 | 17_16 | 8 | 17_04 | 5 | IE | L | 166 | 707.30 | 3.07 | 2376 | 131.29 | 5.12 | 86 | 379.06 | 3.12 | 0.19 | A | 8.00 | 7.10 | 1.77 |
| 2012 | 17_16 | 8 | 17_08 | 5 | IE | L | 183 | 703.88 | 2.82 | 2081 | 126.78 | 5.01 | 94 | 311.00 | 4.99 | 0.18 | A | 8.00 | 3.62 | 0.91 |
| 2012 | 17_16 | 8 | 17_08 | 5 | IE | L | 236 | 694.07 | 2.96 | 2174 | 125.30 | 5.03 | 62 | 312.38 | 3.07 | 0.18 | A | 8.00 | 3.94 | 0.99 |
| 2012 | 17_16 | 8 | 17_08 | 5 | IE | L | 96 | 706.95 | 2.56 | 1999 | 127.14 | 4.65 | 73 | 340.56 | 4.38 | 0.18 | A | 8.00 | 5.43 | 1.36 |
| 2012 | 17_16 | 8 | 17_08 | 5 | IE | L | 225 | 704.82 | 3.11 | 1525 | 128.05 | 5.26 | 86 | 377.93 | 3.12 | 0.18 | A | 8.00 | 7.61 | 1.90 |
| 2012 | 17_16 | 8 | 17_08 | 5 | IE | L | 212 | 702.92 | 2.65 | 1945 | 128.26 | 5.56 | 103 | 366.12 | 3.78 | 0.18 | A | 8.00 | 6.84 | 1.71 |
| 2012 | 17_16 | 8 | 17_14 | 8 | IA | L | 203 | 702.67 | 3.01 | 1543 | 125.39 | 5.06 | 88 | 359.06 | 3.64 | 0.18 | A | 8.00 | 6.91 | 1.73 |
| 2012 | 17_16 | 8 | 17_16 | 8 | S | L | 181 | 707.15 | 2.60 | 1855 | 127.02 | 4.74 | 58 | 364.30 | 3.58 | 0.18 | A | 8.00 | 6.94 | 1.74 |
| 2012 | 17_16 | 8 | 17_16 | 8 | S | L | 177 | 711.09 | 2.35 | 2118 | 124.99 | 4.71 | 98 | 361.05 | 3.90 | 0.18 | A | 8.00 | 7.11 | 1.78 |
| 2012 | 17_16 | 8 | 17_19 | 8 | IA | L | 135 | 698.86 | 2.38 | 1913 | 122.77 | 6.72 | 86 | 357.45 | 3.88 | 0.18 | A | 8.00 | 7.29 | 1.82 |
| 2012 | 17_17 | 8 | 17_08 | 5 | IE | L | 180 | 710.19 | 3.01 | 1563 | 127.68 | 4.89 | 62 | 330.81 | 3.70 | 0.18 | A | 8.00 | 4.73 | 1.18 |
| 2012 | 17_17 | 8 | 17_08 | 5 | IE | L | 184 | 709.30 | 2.66 | 1857 | 125.60 | 5.02 | 86 | 338.11 | 3.38 | 0.18 | A | 8.00 | 5.54 | 1.38 |
| 2012 | 17_17 | 8 | 17_08 | 5 | IE | L | 191 | 706.64 | 2.73 | 2105 | 125.63 | 5.04 | 80 | 342.21 | 4.30 | 0.18 | A | 8.00 | 5.79 | 1.45 |
| 2012 | 17_17 | 8 | 17_09 | 5 | IE | L | 111 | 708.14 | 2.41 | 2036 | 126.41 | 4.96 | 43 | 506.42 | 4.56 | 0.18 | A | 8.00 | 16.05 | 4.01 |
| 2012 | 17_17 | 8 | 17_14 | 8 | IA | L | 179 | 701.85 | 3.04 | 2318 | 124.01 | 5.33 | 97 | 344.03 | 5.88 | 0.18 | A | 8.00 | 6.19 | 1.55 |
| 2012 | 17_17 | 8 | 17_14 | 8 | IA | L | 165 | 705.41 | 3.13 | 1641 | 126.09 | 5.37 | 57 | 358.89 | 4.31 | 0.18 | A | 8.00 | 6.77 | 1.69 |
| 2012 | 17_17 | 8 | 17_17 | 8 | S | L | 135 | 701.77 | 2.53 | 2225 | 124.68 | 4.67 | 68 | 369.44 | 3.98 | 0.18 | A | 8.00 | 7.70 | 1.93 |
| 2012 | 17_17 | 8 | 17_17 | 8 | S | L | 150 | 701.99 | 2.81 | 2041 | 124.55 | 4.79 | 91 | 364.90 | 4.02 | 0.18 | A | 8.00 | 7.44 | 1.86 |
| 2012 | 17_17 | 8 | 17_17 | 8 | S | L | 174 | 699.92 | 2.09 | 1970 | 124.13 | 4.15 | 41 | 352.83 | 3.25 | 0.18 | A | 8.00 | 6.74 | 1.68 |
| 2012 | 17_17 | 8 | 17_17 | 8 | S | L | 165 | 694.83 | 2.71 | 2016 | 121.58 | 4.73 | 49 | 362.21 | 3.18 | 0.17 | A | 8.00 | 7.83 | 1.96 |
| 2012 | 17_17 | 8 | 17_17 | 8 | S | L | 190 | 698.35 | 2.48 | 1475 | 125.44 | 4.52 | 101 | 360.92 | 4.61 | 0.18 | A | 8.00 | 7.02 | 1.75 |
| 2012 | 17_17 | 8 | 17_19 | 8 | IA | L | 150 | 714.89 | 3.16 | 1938 | 127.54 | 4.95 | 135 | 384.12 | 4.45 | 0.18 | A | 8.00 | 8.09 | 2.02 |
| 2012 | 17_19 | 8 | 17_09 | 5 | IE | L | 134 | 714.86 | 2.78 | 1977 | 128.77 | 4.58 | 70 | 363.73 | 3.78 | 0.18 | A | 8.00 | 6.60 | 1.65 |
| 2012 | 17_19 | 8 | 17_14 | 8 | IA | L | 163 | 696.53 | 2.84 | 2491 | 122.58 | 4.60 | 58 | 343.19 | 4.73 | 0.18 | A | 8.00 | 6.40 | 1.60 |
| 2012 | 17_19 | 8 | 17_15 | 8 | IA | L | 136 | 714.44 | 2.88 | 2089 | 125.54 | 4.69 | 62 | 356.77 | 4.33 | 0.18 | A | 8.00 | 6.74 | 1.68 |
| 2012 | 17_19 | 8 | 17_16 | 8 | IA | L | 136 | 702.29 | 2.64 | 1632 | 122.93 | 4.64 | 61 | 299.81 | 5.14 | 0.18 | A | 8.00 | 3.51 | 0.88 |
| 2012 | 17_19 | 8 | 17_19 | 8 | S | L | 244 | 700.84 | 3.29 | 1953 | 123.21 | 4.61 | 69 | 346.62 | 4.45 | 0.18 | A | 8.00 | 6.51 | 1.63 |
| 2012 | 17_19 | 8 | 17_19 | 8 | S | L | 164 | 710.76 | 3.41 | 2425 | 123.74 | 5.54 | 54 | 383.82 | 3.90 | 0.17 | A | 8.00 | 8.81 | 2.20 |
| 2012 | 17_19 | 8 | 17_19 | 8 | S | L | 151 | 702.92 | 3.15 | 2328 | 122.32 | 5.33 | 39 | 373.88 | 2.44 | 0.17 | A | 8.00 | 8.45 | 2.11 |
| 2012 | 17_19 | 8 | 17_19 | 8 | S | L | 161 | 700.31 | 3.51 | 2257 | 123.31 | 5.65 | 52 | 375.42 | 4.44 | 0.18 | A | 8.00 | 8.36 | 2.09 |
| 2012 | 17_27 | 5 | 17_27 | 5 | S | P | 220 | 601.00 | 2.24 | 2383 | 108.65 | 5.31 | 102 | 338.27 | 3.15 | 0.18 | A | 5.00 | 5.57 | 2.23 |
| 2012 | 17_27 | 5 | 17_27 | 5 | S | P | 246 | 599.46 | 2.53 | 2005 | 108.00 | 5.03 | 111 | 331.04 | 3.43 | 0.18 | A | 5.00 | 5.33 | 2.13 |
| 2012 | 17_27 | 5 | 17_45 | 5 | IA | P | 388 | 618.33 | 3.61 | 1617 | 111.87 | 6.63 | 128 | 332.11 | 5.28 | 0.18 | A | 5.00 | 4.84 | 1.94 |
| 2012 | 17_28 | 7 | 17_29 | 7 | IA | P | 102 | 595.24 | 2.89 | 681 | 160.56 | 5.97 | 136 | 453.93 | 3.59 | 0.27 | A | 7.00 | 5.79 | 1.65 |
| 2012 | 17_28 | 7 | 17_30 | 5 | IE | P | 142 | 615.56 | 2.31 | 2207 | 153.94 | 4.62 | 102 | 458.66 | 2.77 | 0.25 | A | 7.00 | 6.86 | 1.96 |
| 2012 | 17_28 | 7 | 17_45 | 5 | IE | P | 245 | 598.21 | 2.45 | 2049 | 145.42 | 4.61 | 89 | 428.90 | 3.55 | 0.24 | A | 7.00 | 6.65 | 1.90 |
| 2012 | 17_29 | 7 | 17_38 | 5 | IE | P | 178 | 603.03 | 2.20 | 2073 | 136.61 | 4.73 | 112 | 406.04 | 3.01 | 0.23 | A | 7.00 | 6.81 | 1.94 |
| 2012 | 17_29 | 7 | 17_49 | 7 | IA | P | 171 | 596.13 | 2.27 | 2062 | 138.71 | 5.37 | 192 | 214.86 | 3.42 | 0.23 | S | 6.57 | 2.96 | 0.85 |
| 2012 | 17_30 | 5 | 17_28 | 7 | IE | P | 490 | 600.06 | 3.04 | 1454 | 108.78 | 6.29 | 88 | 286.14 | 5.15 | 0.18 | A | 5.00 | 3.15 | 1.26 |
| 2012 | 17_30 | 5 | 17_28 | 7 | IE | P | 290 | 604.59 | 3.11 | 1544 | 108.13 | 5.93 | 111 | 359.33 | 4.45 | 0.18 | A | 5.00 | 6.62 | 2.65 |
| 2012 | 17_30 | 5 | 17_29 | 7 | IE | P | 221 | 600.72 | 2.59 | 1844 | 108.35 | 5.59 | 104 | 334.04 | 3.38 | 0.18 | A | 5.00 | 5.41 | 2.17 |
| 2012 | 17_30 | 5 | 17_29 | 7 | IE | P | 221 | 600.72 | 2.59 | 1844 | 108.35 | 5.59 | 40 | 412.06 | 2.50 | 0.18 | A | 5.00 | 9.02 | 3.61 |
| 2012 | 17_30 | 5 | 17_45 | 5 | IA | P | 244 | 600.66 | 3.48 | 1716 | 105.68 | 5.48 | 115 | 317.59 | 4.80 | 0.18 | A | 5.00 | 5.03 | 2.01 |
| 2012 | 17_38 | 5 | 17_27 | 5 | IA | P | 421 | 600.96 | 3.90 | 1315 | 112.11 | 7.87 | 58 | 323.57 | 4.29 | 0.19 | A | 5.00 | 4.43 | 1.77 |
| 2012 | 17_38 | 5 | 17_29 | 7 | IE | P | 326 | 601.58 | 2.54 | 1991 | 107.02 | 5.34 | 103 | 346.74 | 3.85 | 0.18 | A | 5.00 | 6.20 | 2.48 |
| 2012 | 17_38 | 5 | 17_29 | 7 | IE | P | 330 | 598.48 | 2.77 | 2253 | 106.13 | 5.46 | 109 | 331.94 | 4.30 | 0.18 | A | 5.00 | 5.64 | 2.26 |
| 2012 | 17_49 | 7 | 17_38 | 5 | IE | P | 185 | 604.54 | 1.90 | 1805 | 146.42 | 4.32 | 60 | 440.26 | 3.80 | 0.24 | A | 7.00 | 7.05 | 2.01 |
| 2012 | 17_49 | 7 | 17_45 | 5 | IE | P | 201 | 599.40 | 2.63 | 2096 | 146.94 | 5.41 | 89 | 424.82 | 3.68 | 0.25 | A | 7.00 | 6.24 | 1.78 |
| 2012 | 17_49 | 7 | 17_45 | 5 | IE | P | 185 | 603.85 | 2.24 | 2270 | 146.27 | 4.51 | 84 | 429.97 | 3.43 | 0.24 | A | 7.00 | 6.58 | 1.88 |
| 2012 | 18_01 | 6 | 18_01 | 6 | S | P | 186 | 600.31 | 1.88 | 1781 | 127.77 | 5.20 | 83 | 384.62 | 3.27 | 0.21 | A | 6.00 | 6.06 | 2.02 |
| 2012 | 18_01 | 6 | 18_02 | 6 | IA | P | 285 | 611.16 | 2.69 | 1994 | 137.46 | 4.89 | 128 | 406.94 | 4.41 | 0.22 | A | 6.00 | 5.76 | 1.92 |
| 2012 | 18_01 | 6 | 18_02 | 6 | IA | P | 280 | 604.88 | 2.17 | 1820 | 131.37 | 4.80 | 111 | 392.91 | 3.37 | 0.22 | A | 6.00 | 5.95 | 1.98 |
| 2012 | 18_01 | 6 | 18_05 | 6 | IA | P | 301 | 597.06 | 2.31 | 1729 | 130.36 | 5.50 | 81 | 329.47 | 4.50 | 0.22 | A | 6.00 | 3.16 | 1.05 |
| 2012 | 18_01 | 6 | 18_05 | 6 | IA | P | 218 | 597.59 | 2.00 | 2052 | 130.02 | 5.21 | 76 | 384.57 | 3.11 | 0.22 | A | 6.00 | 5.75 | 1.92 |
| 2012 | 18_01 | 6 | 18_07 | 6 | IA | P | 359 | 607.19 | 2.39 | 1909 | 132.24 | 5.01 | 131 | 385.53 | 3.87 | 0.22 | A | 6.00 | 5.49 | 1.83 |
| 2012 | 18_01 | 6 | 18_07 | 6 | IA | P | 219 | 595.48 | 1.95 | 2112 | 126.42 | 4.61 | 124 | 377.46 | 3.45 | 0.21 | A | 6.00 | 5.91 | 1.97 |
| 2012 | 18_01 | 6 | 18_14 | 4 | IE | P | 202 | 618.74 | 2.63 | 2094 | 135.74 | 5.64 | 101 | 394.21 | 3.89 | 0.22 | A | 6.00 | 5.42 | 1.81 |
| 2012 | 18_01 | 6 | 18_14 | 4 | IE | P | 219 | 603.50 | 2.02 | 1869 | 129.02 | 4.92 | 90 | 376.58 | 3.49 | 0.21 | A | 6.00 | 5.51 | 1.84 |
| 2012 | 18_01 | 6 | 18_15 | 4 | IE | P | 264 | 615.01 | 2.16 | 2193 | 134.28 | 4.84 | 159 | 392.60 | 3.16 | 0.22 | A | 6.00 | 5.54 | 1.85 |
| 2012 | 18_01 | 6 | 18_15 | 4 | IE | P | 198 | 599.32 | 2.35 | 1849 | 128.93 | 4.65 | 117 | 309.29 | 3.12 | 0.22 | A | 6.00 | 2.39 | 0.80 |
| 2012 | 18_01 | 6 | 18_15 | 4 | IE | P | 202 | 597.59 | 1.97 | 1622 | 128.46 | 4.94 | 110 | 375.10 | 3.67 | 0.21 | A | 6.00 | 5.52 | 1.84 |
| 2012 | 18_01 | 6 | 18_23 | 7 | IE | P | 295 | 599.97 | 2.22 | 1875 | 129.31 | 5.43 | 111 | 383.14 | 3.51 | 0.22 | A | 6.00 | 5.78 | 1.93 |
| 2012 | 18_01 | 6 | 18_23 | 7 | IE | P | 299 | 602.50 | 2.18 | 1741 | 131.43 | 5.23 | 105 | 509.13 | 3.92 | 0.22 | A | 6.00 | 11.24 | 3.75 |
| 2012 | 18_01 | 6 | 18_23 | 7 | IE | P | 277 | 597.82 | 1.79 | 2192 | 126.91 | 4.92 | 105 | 380.52 | 3.05 | 0.21 | A | 6.00 | 5.99 | 2.00 |
| 2012 | 18_01 | 6 | 18_23 | 7 | IE | P | 330 | 599.38 | 2.01 | 1895 | 128.74 | 4.98 | 84 | 385.63 | 3.21 | 0.21 | A | 6.00 | 5.97 | 1.99 |
| 2012 | 18_01 | 6 | 18_25 | 5 | IE | P | 183 | 597.92 | 1.69 | 2098 | 127.68 | 4.89 | 123 | 372.92 | 2.94 | 0.21 | A | 6.00 | 5.52 | 1.84 |
| 2012 | 18_01 | 6 | 18_27 | 8 | IE | P | 167 | 592.56 | 1.86 | 2082 | 126.15 | 4.65 | 99 | 395.17 | 3.21 | 0.21 | A | 6.00 | 6.80 | 2.27 |
| 2012 | 18_01 | 6 | 18_36 | 5 | IE | P | 253 | 599.34 | 1.98 | 2307 | 127.53 | 4.76 | 110 | 381.67 | 3.41 | 0.21 | A | 6.00 | 5.96 | 1.99 |
| 2012 | 18_01 | 6 | 18_36 | 5 | IE | P | 161 | 601.88 | 2.20 | 2158 | 128.26 | 5.06 | 112 | 375.72 | 2.91 | 0.21 | A | 6.00 | 5.58 | 1.86 |
| 2012 | 18_01 | 6 | 18_37 | 7 | IE | P | 318 | 597.25 | 2.05 | 1870 | 128.03 | 4.47 | 123 | 336.48 | 2.80 | 0.21 | A | 6.00 | 3.77 | 1.26 |
| 2012 | 18_01 | 6 | 18_38 | 7 | IE | P | 228 | 590.42 | 1.96 | 2058 | 128.46 | 6.01 | 94 | 379.54 | 3.75 | 0.22 | A | 6.00 | 5.73 | 1.91 |
| 2012 | 18_01 | 6 | 18_38 | 7 | IE | P | 200 | 600.74 | 1.80 | 2345 | 129.25 | 4.74 | 121 | 383.18 | 3.29 | 0.22 | A | 6.00 | 5.79 | 1.93 |
| 2012 | 18_01 | 6 | 18_38 | 7 | IE | P | 298 | 599.13 | 1.92 | 1937 | 128.21 | 4.95 | 113 | 375.07 | 3.53 | 0.21 | A | 6.00 | 5.55 | 1.85 |
| 2012 | 18_01 | 6 | 18_38 | 7 | IE | P | 301 | 592.28 | 1.94 | 1492 | 126.28 | 4.85 | 111 | 393.24 | 3.83 | 0.21 | A | 6.00 | 6.68 | 2.23 |
| 2012 | 18_01 | 6 | 18_38 | 7 | IE | P | 443 | 590.39 | 2.19 | 1858 | 126.11 | 4.70 | 88 | 328.37 | 3.38 | 0.21 | A | 6.00 | 3.62 | 1.21 |
| 2012 | 18_01 | 6 | 18_38 | 7 | IE | P | 318 | 595.36 | 2.11 | 296 | 128.78 | 5.37 | 89 | 206.59 | 3.36 | 0.22 | S | 6.06 | 2.40 | 0.80 |
| 2012 | 18_01 | 6 | 18_38 | 7 | IE | P | 228 | 607.94 | 1.76 | 2037 | 130.48 | 4.63 | 110 | 385.42 | 3.80 | 0.21 | A | 6.00 | 5.72 | 1.91 |
| 2012 | 18_01 | 6 | 18_38 | 7 | IE | P | 195 | 600.24 | 1.96 | 1976 | 127.44 | 4.61 | 146 | 393.80 | 3.74 | 0.21 | A | 6.00 | 6.54 | 2.18 |
| 2012 | 18_01 | 6 | 18_43 | 4 | IE | P | 307 | 608.24 | 2.13 | 1942 | 132.57 | 4.57 | 111 | 394.70 | 3.37 | 0.22 | A | 6.00 | 5.86 | 1.95 |
| 2012 | 18_01 | 6 | 18_43 | 4 | IE | P | 209 | 599.26 | 1.80 | 2216 | 129.87 | 4.90 | 106 | 421.38 | 3.11 | 0.22 | A | 6.00 | 7.47 | 2.49 |
| 2012 | 18_01 | 6 | 18_43 | 4 | IE | P | 232 | 599.49 | 1.69 | 2016 | 127.39 | 4.51 | 104 | 372.79 | 3.39 | 0.21 | A | 6.00 | 5.56 | 1.85 |
| 2012 | 18_01 | 6 | 18_43 | 4 | IE | P | 237 | 593.48 | 1.82 | 1618 | 129.16 | 4.56 | 94 | 384.39 | 3.35 | 0.22 | A | 6.00 | 5.86 | 1.95 |
| 2012 | 18_01 | 6 | 18_43 | 4 | IE | P | 217 | 597.27 | 2.06 | 1221 | 206.88 | 3.68 | 111 | 333.45 | 3.39 | 0.35 | AF | 9.70 |  |  |
| 2012 | 18_01 | 6 | 18_43 | 4 | IE | P | 237 | 602.23 | 2.06 | 2039 | 128.94 | 4.39 | 102 | 379.09 | 2.99 | 0.21 | A | 6.00 | 5.64 | 1.88 |
| 2012 | 18_01 | 6 | 18_44 | 8 | IE | P | 334 | 595.88 | 2.16 | 1966 | 127.10 | 4.60 | 116 | 400.04 | 3.07 | 0.21 | A | 6.00 | 6.88 | 2.29 |
| 2012 | 18_01 | 6 | 18_44 | 8 | IE | P | 189 | 606.16 | 2.01 | 2048 | 127.88 | 4.57 | 102 | 509.37 | 3.06 | 0.21 | A | 6.00 | 11.90 | 3.97 |
| 2012 | 18_01 | 6 | 18_44 | 8 | IE | P | 239 | 610.61 | 1.50 | 1993 | 130.07 | 4.91 | 105 | 437.67 | 3.46 | 0.21 | A | 6.00 | 8.19 | 2.73 |
| 2012 | 18_01 | 6 | 18_44 | 8 | IE | P | 228 | 605.70 | 1.94 | 1887 | 127.92 | 4.69 | 99 | 343.97 | 2.82 | 0.21 | A | 6.00 | 4.13 | 1.38 |
| 2012 | 18_01 | 6 | 18_44 | 8 | IE | P | 298 | 598.99 | 1.81 | 2022 | 126.39 | 4.16 | 119 | 392.27 | 3.26 | 0.21 | A | 6.00 | 6.62 | 2.21 |
| 2012 | 18_01 | 6 | 18_47 | 4 | IE | P | 306 | 603.30 | 1.72 | 2020 | 131.19 | 5.12 | 123 | 378.60 | 3.34 | 0.22 | A | 6.00 | 5.32 | 1.77 |
| 2012 | 18_01 | 6 | 18_47 | 4 | IE | P | 289 | 599.42 | 2.11 | 1868 | 127.50 | 5.02 | 110 | 373.10 | 3.17 | 0.21 | A | 6.00 | 5.56 | 1.85 |
| 2012 | 18_01 | 6 | 18_47 | 4 | IE | P | 440 | 594.87 | 2.39 | 1602 | 128.06 | 5.13 | 77 | 327.24 | 3.07 | 0.22 | A | 6.00 | 3.33 | 1.11 |
| 2012 | 18_02 | 6 | 18_01 | 6 | IA | P | 170 | 614.98 | 2.54 | 1921 | 136.78 | 4.29 | 101 | 404.04 | 3.32 | 0.22 | A | 6.00 | 5.72 | 1.91 |
| 2012 | 18_02 | 6 | 18_01 | 6 | IA | P | 233 | 602.97 | 2.38 | 1796 | 130.90 | 4.94 | 125 | 383.10 | 3.80 | 0.22 | A | 6.00 | 5.56 | 1.85 |
| 2012 | 18_02 | 6 | 18_02 | 6 | S | P | 178 | 606.16 | 2.50 | 1888 | 133.18 | 4.99 | 155 | 388.09 | 3.44 | 0.22 | A | 6.00 | 5.48 | 1.83 |
| 2012 | 18_02 | 6 | 18_05 | 6 | IA | P | 204 | 599.61 | 2.69 | 2843 | 126.16 | 5.08 | 135 | 368.28 | 2.92 | 0.21 | A | 6.00 | 5.51 | 1.84 |
| 2012 | 18_02 | 6 | 18_14 | 4 | IE | P | 249 | 588.53 | 2.54 | 1980 | 124.35 | 4.43 | 130 | 360.93 | 3.10 | 0.21 | A | 6.00 | 5.42 | 1.81 |
| 2012 | 18_02 | 6 | 18_14 | 4 | IE | P | 296 | 598.36 | 2.78 | 1923 | 128.89 | 4.58 | 71 | 343.06 | 3.38 | 0.22 | A | 6.00 | 3.97 | 1.32 |
| 2012 | 18_02 | 6 | 18_14 | 4 | IE | P | 501 | 603.12 | 2.61 | 1385 | 189.57 | 4.36 | 94 | 324.98 | 3.73 | 0.31 | AF | 8.80 |  |  |
| 2012 | 18_02 | 6 | 18_14 | 4 | IE | P | 225 | 602.86 | 2.87 | 1798 | 127.66 | 5.45 | 96 | 371.60 | 3.28 | 0.21 | A | 6.00 | 5.47 | 1.82 |
| 2012 | 18_02 | 6 | 18_20 | 8 | IE | P | 80 | 600.17 | 1.69 | 984 | 127.28 | 3.92 | 65 | 356.22 | 3.45 | 0.21 | A | 6.00 | 4.79 | 1.60 |
| 2012 | 18_02 | 6 | 18_20 | 8 | IE | P | 410 | 609.12 | 2.70 | 1594 | 128.40 | 5.43 | 137 | 377.29 | 3.97 | 0.21 | A | 6.00 | 5.63 | 1.88 |
| 2012 | 18_02 | 6 | 18_20 | 8 | IE | P | 165 | 599.53 | 1.82 | 974 | 128.73 | 4.29 | 47 | 394.92 | 3.50 | 0.21 | A | 6.00 | 6.41 | 2.14 |
| 2012 | 18_02 | 6 | 18_20 | 8 | IE | P | 91 | 593.94 | 1.94 | 1036 | 128.20 | 4.36 | 51 | 382.88 | 2.03 | 0.22 | A | 6.00 | 5.92 | 1.97 |
| 2012 | 18_02 | 6 | 18_20 | 8 | IE | P | 185 | 598.25 | 1.80 | 1329 | 127.96 | 4.20 | 56 | 387.05 | 2.78 | 0.21 | A | 6.00 | 6.15 | 2.05 |
| 2012 | 18_02 | 6 | 18_20 | 8 | IE | P | 85 | 605.22 | 2.06 | 1006 | 128.99 | 3.62 | 59 | 387.56 | 2.79 | 0.21 | A | 6.00 | 6.03 | 2.01 |
| 2012 | 18_02 | 6 | 18_20 | 8 | IE | P | 165 | 599.53 | 1.82 | 971 | 128.77 | 4.26 | 47 | 394.92 | 3.50 | 0.21 | A | 6.00 | 6.40 | 2.13 |
| 2012 | 18_02 | 6 | 18_20 | 8 | IE | P | 170 | 602.99 | 2.39 | 1148 | 129.81 | 3.85 | 76 | 405.01 | 3.61 | 0.22 | A | 6.00 | 6.72 | 2.24 |
| 2012 | 18_02 | 6 | 18_20 | 8 | IE | P | 132 | 585.05 | 1.94 | 959 | 125.22 | 4.40 | 57 | 386.99 | 2.54 | 0.21 | A | 6.00 | 6.54 | 2.18 |
| 2012 | 18_02 | 6 | 18_20 | 8 | IE | P | 51 | 595.06 | 2.17 | 561 | 129.33 | 3.77 | 41 | 400.63 | 2.68 | 0.22 | A | 6.00 | 6.59 | 2.20 |
| 2012 | 18_02 | 6 | 18_23 | 7 | IE | P | 437 | 607.65 | 2.28 | 1603 | 131.29 | 4.44 | 100 | 386.70 | 3.16 | 0.22 | A | 6.00 | 5.67 | 1.89 |
| 2012 | 18_02 | 6 | 18_23 | 7 | IE | P | 473 | 600.44 | 2.12 | 1676 | 129.31 | 4.49 | 107 | 392.45 | 3.61 | 0.22 | A | 6.00 | 6.21 | 2.07 |
| 2012 | 18_02 | 6 | 18_23 | 7 | IE | P | 449 | 594.86 | 2.67 | 1933 | 129.01 | 4.58 | 160 | 386.03 | 6.33 | 0.22 | A | 6.00 | 5.95 | 1.98 |
| 2012 | 18_02 | 6 | 18_23 | 7 | IE | P | 443 | 588.60 | 2.28 | 1665 | 126.58 | 4.62 | 102 | 371.22 | 3.15 | 0.22 | A | 6.00 | 5.60 | 1.87 |
| 2012 | 18_02 | 6 | 18_23 | 7 | IE | P | 357 | 603.26 | 1.77 | 1988 | 126.02 | 4.67 | 98 | 385.47 | 4.02 | 0.21 | A | 6.00 | 6.35 | 2.12 |
| 2012 | 18_02 | 6 | 18_23 | 7 | IE | P | 322 | 596.75 | 2.11 | 1755 | 129.99 | 4.86 | 95 | 392.81 | 3.35 | 0.22 | A | 6.00 | 6.13 | 2.04 |
| 2012 | 18_02 | 6 | 18_23 | 7 | IE | P | 353 | 596.59 | 1.89 | 1732 | 127.92 | 4.26 | 121 | 375.51 | 2.88 | 0.21 | A | 6.00 | 5.61 | 1.87 |
| 2012 | 18_02 | 6 | 18_23 | 7 | IE | P | 411 | 597.12 | 2.28 | 1588 | 128.11 | 4.35 | 81 | 386.69 | 2.89 | 0.21 | A | 6.00 | 6.11 | 2.04 |
| 2012 | 18_02 | 6 | 18_23 | 7 | IE | P | 398 | 592.23 | 2.15 | 1695 | 127.47 | 4.27 | 94 | 376.69 | 2.57 | 0.22 | A | 6.00 | 5.73 | 1.91 |
| 2012 | 18_02 | 6 | 18_23 | 7 | IE | P | 378 | 611.80 | 1.87 | 2191 | 127.65 | 4.49 | 106 | 388.46 | 3.58 | 0.21 | A | 6.00 | 6.26 | 2.09 |
| 2012 | 18_02 | 6 | 18_27 | 8 | IE | P | 298 | 601.25 | 3.69 | 2084 | 125.89 | 5.16 | 90 | 332.42 | 4.28 | 0.21 | A | 6.00 | 3.84 | 1.28 |
| 2012 | 18_02 | 6 | 18_27 | 8 | IE | P | 148 | 597.38 | 3.20 | 2160 | 126.02 | 4.92 | 94 | 382.50 | 3.64 | 0.21 | A | 6.00 | 6.21 | 2.07 |
| 2012 | 18_02 | 6 | 18_27 | 8 | IE | P | 157 | 598.06 | 3.03 | 2373 | 125.57 | 4.90 | 105 | 333.26 | 3.83 | 0.21 | A | 6.00 | 3.92 | 1.31 |
| 2012 | 18_02 | 6 | 18_27 | 8 | IE | P | 220 | 595.81 | 2.79 | 2213 | 126.61 | 4.80 | 117 | 336.27 | 3.92 | 0.21 | A | 6.00 | 3.94 | 1.31 |
| 2012 | 18_02 | 6 | 18_27 | 8 | IE | P | 189 | 599.32 | 3.14 | 2224 | 126.35 | 4.89 | 94 | 338.12 | 4.21 | 0.21 | A | 6.00 | 4.06 | 1.35 |
| 2012 | 18_02 | 6 | 18_27 | 8 | IE | P | 125 | 596.06 | 1.54 | 1854 | 124.85 | 3.77 | 79 | 536.32 | 2.98 | 0.21 | A | 6.00 | 13.77 | 4.59 |
| 2012 | 18_02 | 6 | 18_27 | 8 | IE | P | 196 | 595.51 | 2.10 | 1824 | 125.30 | 4.09 | 94 | 342.69 | 3.57 | 0.21 | A | 6.00 | 4.41 | 1.47 |
| 2012 | 18_02 | 6 | 18_27 | 8 | IE | P | 141 | 597.43 | 2.39 | 2218 | 125.77 | 4.40 | 103 | 376.19 | 3.47 | 0.21 | A | 6.00 | 5.95 | 1.98 |
| 2012 | 18_02 | 6 | 18_27 | 8 | IE | P | 202 | 597.55 | 2.93 | 2055 | 126.29 | 4.86 | 87 | 375.93 | 3.61 | 0.21 | A | 6.00 | 5.86 | 1.95 |
| 2012 | 18_02 | 6 | 18_27 | 8 | IE | P | 193 | 596.27 | 2.75 | 1877 | 124.29 | 4.67 | 59 | 519.27 | 2.49 | 0.21 | A | 6.00 | 13.07 | 4.36 |
| 2012 | 18_02 | 6 | 18_29 | 5 | IE | P | 153 | 607.37 | 2.25 | 2238 | 131.94 | 4.30 | 107 | 387.78 | 2.86 | 0.22 | A | 6.00 | 5.63 | 1.88 |
| 2012 | 18_02 | 6 | 18_29 | 5 | IE | P | 250 | 608.79 | 1.70 | 2730 | 129.34 | 4.56 | 135 | 406.13 | 4.05 | 0.21 | A | 6.00 | 6.84 | 2.28 |
| 2012 | 18_02 | 6 | 18_32 | 6 | IA | P | 199 | 596.36 | 1.87 | 2071 | 125.79 | 4.60 | 116 | 373.20 | 3.45 | 0.21 | A | 6.00 | 5.80 | 1.93 |
| 2012 | 18_02 | 6 | 18_37 | 7 | IE | P | 528 | 602.19 | 2.36 | 1472 | 130.80 | 4.61 | 94 | 412.81 | 3.45 | 0.22 | A | 6.00 | 6.94 | 2.31 |
| 2012 | 18_02 | 6 | 18_37 | 7 | IE | P | 559 | 602.59 | 2.32 | 1542 | 130.50 | 4.90 | 102 | 334.50 | 3.11 | 0.22 | A | 6.00 | 3.38 | 1.13 |
| 2012 | 18_02 | 6 | 18_37 | 7 | IE | P | 458 | 613.29 | 2.15 | 1760 | 132.45 | 4.86 | 92 | 409.82 | 2.96 | 0.22 | A | 6.00 | 6.56 | 2.19 |
| 2012 | 18_02 | 6 | 18_37 | 7 | IE | P | 436 | 602.18 | 2.19 | 1821 | 127.70 | 4.60 | 111 | 379.32 | 2.90 | 0.21 | A | 6.00 | 5.82 | 1.94 |
| 2012 | 18_02 | 6 | 18_37 | 7 | IE | P | 401 | 601.29 | 2.61 | 1810 | 128.18 | 5.16 | 73 | 387.07 | 4.92 | 0.21 | A | 6.00 | 6.12 | 2.04 |
| 2012 | 18_02 | 6 | 18_37 | 7 | IE | P | 435 | 591.48 | 2.27 | 1680 | 129.25 | 4.62 | 103 | 399.46 | 3.50 | 0.22 | A | 6.00 | 6.54 | 2.18 |
| 2012 | 18_02 | 6 | 18_37 | 7 | IE | P | 400 | 598.67 | 2.18 | 1361 | 129.09 | 4.54 | 99 | 388.30 | 3.72 | 0.22 | A | 6.00 | 6.05 | 2.02 |
| 2012 | 18_02 | 6 | 18_37 | 7 | IE | P | 550 | 602.31 | 2.23 | 1769 | 131.08 | 4.91 | 106 | 339.42 | 3.68 | 0.22 | A | 6.00 | 3.54 | 1.18 |
| 2012 | 18_02 | 6 | 18_37 | 7 | IE | P | 523 | 596.84 | 2.05 | 1693 | 129.58 | 4.59 | 134 | 393.48 | 3.83 | 0.22 | A | 6.00 | 6.22 | 2.07 |
| 2012 | 18_02 | 6 | 18_37 | 7 | IE | P | 613 | 604.04 | 2.44 | 1721 | 126.62 | 5.25 | 127 | 347.46 | 3.52 | 0.21 | A | 6.00 | 4.46 | 1.49 |
| 2012 | 18_02 | 6 | 18_38 | 7 | IE | P | 197 | 603.40 | 2.38 | 2024 | 133.28 | 4.57 | 79 | 522.13 | 2.11 | 0.22 | A | 6.00 | 11.51 | 3.84 |
| 2012 | 18_02 | 6 | 18_38 | 7 | IE | P | 168 | 605.64 | 2.53 | 2062 | 129.97 | 4.38 | 130 | 400.20 | 3.56 | 0.21 | A | 6.00 | 6.48 | 2.16 |
| 2012 | 18_02 | 6 | 18_38 | 7 | IE | P | 200 | 602.80 | 2.27 | 2189 | 127.35 | 4.85 | 101 | 504.55 | 3.16 | 0.21 | A | 6.00 | 11.77 | 3.92 |
| 2012 | 18_02 | 6 | 18_41 | 8 | IE | P | 327 | 601.47 | 2.32 | 1938 | 129.86 | 4.73 | 116 | 421.25 | 2.48 | 0.22 | A | 6.00 | 7.46 | 2.49 |
| 2012 | 18_02 | 6 | 18_41 | 8 | IE | P | 290 | 607.47 | 2.70 | 2188 | 126.21 | 5.08 | 114 | 385.89 | 4.34 | 0.21 | A | 6.00 | 6.35 | 2.12 |
| 2012 | 18_02 | 6 | 18_41 | 8 | IE | P | 185 | 593.70 | 1.97 | 2069 | 125.17 | 5.00 | 98 | 415.90 | 3.17 | 0.21 | A | 6.00 | 7.94 | 2.65 |
| 2012 | 18_02 | 6 | 18_44 | 8 | IE | P | 166 | 594.85 | 3.66 | 2045 | 123.45 | 6.82 | 86 | 412.12 | 4.13 | 0.21 | A | 6.00 | 8.03 | 2.68 |
| 2012 | 18_02 | 6 | 18_47 | 4 | IE | P | 178 | 601.46 | 2.18 | 2215 | 127.16 | 4.85 | 97 | 374.86 | 2.94 | 0.21 | A | 6.00 | 5.69 | 1.90 |
| 2012 | 18_02 | 6 | 18_48 | 4 | IE | P | 346 | 599.47 | 2.48 | 1693 | 124.31 | 4.64 | 130 | 187.03 | 2.51 | 0.21 | S | 5.81 | 2.88 | 0.96 |
| 2012 | 18_02 | 6 | 18_48 | 4 | IE | P | 197 | 609.27 | 2.55 | 2051 | 128.32 | 4.32 | 92 | 382.90 | 3.21 | 0.21 | A | 6.00 | 5.90 | 1.97 |
| 2012 | 18_05 | 6 | 18_01 | 6 | IA | P | 253 | 599.44 | 1.94 | 1975 | 127.46 | 4.28 | 100 | 367.87 | 3.34 | 0.21 | A | 6.00 | 5.32 | 1.77 |
| 2012 | 18_05 | 6 | 18_05 | 6 | S | P | 136 | 603.10 | 2.57 | 2296 | 127.04 | 4.67 | 131 | 377.77 | 3.73 | 0.21 | A | 6.00 | 5.84 | 1.95 |
| 2012 | 18_05 | 6 | 18_05 | 6 | S | P | 200 | 603.38 | 2.74 | 2427 | 127.48 | 4.97 | 128 | 377.25 | 3.43 | 0.21 | A | 6.00 | 5.76 | 1.92 |
| 2012 | 18_05 | 6 | 18_07 | 6 | IA | P | 343 | 607.26 | 2.33 | 1677 | 128.96 | 4.60 | 168 | 383.81 | 3.36 | 0.21 | A | 6.00 | 5.86 | 1.95 |
| 2012 | 18_05 | 6 | 18_11 | 5 | IE | P | 244 | 601.93 | 1.90 | 2062 | 126.65 | 4.18 | 112 | 372.39 | 3.29 | 0.21 | A | 6.00 | 5.64 | 1.88 |
| 2012 | 18_05 | 6 | 18_14 | 4 | IE | P | 334 | 606.41 | 2.76 | 1923 | 129.64 | 4.66 | 125 | 381.06 | 3.91 | 0.21 | A | 6.00 | 5.64 | 1.88 |
| 2012 | 18_05 | 6 | 18_14 | 4 | IE | P | 256 | 595.22 | 2.77 | 2030 | 124.19 | 4.36 | 125 | 373.27 | 3.11 | 0.21 | A | 6.00 | 6.03 | 2.01 |
| 2012 | 18_05 | 6 | 18_15 | 4 | IE | P | 376 | 605.31 | 3.35 | 1575 | 252.65 | 3.93 | 100 | 378.63 | 4.51 | 0.42 | AF | 11.69 |  |  |
| 2012 | 18_05 | 6 | 18_15 | 4 | IE | P | 439 | 609.72 | 2.84 | 1665 | 127.14 | 4.62 | 125 | 373.52 | 4.17 | 0.21 | A | 6.00 | 5.63 | 1.88 |
| 2012 | 18_05 | 6 | 18_15 | 4 | IE | P | 264 | 610.49 | 3.12 | 2227 | 129.32 | 4.53 | 158 | 383.48 | 3.65 | 0.21 | A | 6.00 | 5.79 | 1.93 |
| 2012 | 18_05 | 6 | 18_15 | 4 | IE | P | 252 | 610.24 | 2.44 | 1907 | 129.87 | 4.44 | 100 | 384.97 | 3.54 | 0.21 | A | 6.00 | 5.79 | 1.93 |
| 2012 | 18_05 | 6 | 18_15 | 4 | IE | P | 238 | 607.99 | 2.40 | 2016 | 126.97 | 4.30 | 126 | 375.46 | 3.75 | 0.21 | A | 6.00 | 5.74 | 1.91 |
| 2012 | 18_05 | 6 | 18_15 | 4 | IE | P | 293 | 603.00 | 2.83 | 2114 | 125.18 | 4.45 | 129 | 378.74 | 3.62 | 0.21 | A | 6.00 | 6.15 | 2.05 |
| 2012 | 18_05 | 6 | 18_15 | 4 | IE | P | 238 | 603.42 | 2.60 | 1984 | 124.99 | 4.46 | 118 | 374.09 | 3.61 | 0.21 | A | 6.00 | 5.96 | 1.99 |
| 2012 | 18_05 | 6 | 18_23 | 7 | IE | P | 213 | 606.96 | 2.55 | 2108 | 127.56 | 4.79 | 101 | 404.17 | 3.31 | 0.21 | A | 6.00 | 7.01 | 2.34 |
| 2012 | 18_05 | 6 | 18_25 | 5 | IE | P | 253 | 588.62 | 4.60 | 2027 | 119.20 | 7.45 | 119 | 300.78 | 4.83 | 0.20 | A | 6.00 | 3.14 | 1.05 |
| 2012 | 18_05 | 6 | 18_27 | 8 | IE | P | 174 | 607.48 | 2.89 | 1877 | 124.64 | 5.22 | 114 | 368.01 | 4.04 | 0.21 | A | 6.00 | 5.72 | 1.91 |
| 2012 | 18_05 | 6 | 18_29 | 5 | IE | P | 208 | 592.91 | 2.51 | 2108 | 125.36 | 4.47 | 128 | 368.25 | 3.50 | 0.21 | A | 6.00 | 5.63 | 1.88 |
| 2012 | 18_05 | 6 | 18_29 | 5 | IE | P | 205 | 601.74 | 2.51 | 2049 | 126.92 | 4.26 | 124 | 365.91 | 3.62 | 0.21 | A | 6.00 | 5.30 | 1.77 |
| 2012 | 18_05 | 6 | 18_36 | 5 | IE | P | 261 | 605.32 | 2.55 | 2131 | 126.14 | 4.27 | 124 | 340.93 | 2.67 | 0.21 | A | 6.00 | 4.22 | 1.41 |
| 2012 | 18_05 | 6 | 18_37 | 7 | IE | P | 267 | 606.40 | 2.52 | 1903 | 125.65 | 4.66 | 89 | 318.92 | 2.94 | 0.21 | A | 6.00 | 3.23 | 1.08 |
| 2012 | 18_05 | 6 | 18_41 | 8 | IE | P | 148 | 600.14 | 2.39 | 1802 | 126.22 | 5.14 | 79 | 403.88 | 2.72 | 0.21 | A | 6.00 | 7.20 | 2.40 |
| 2012 | 18_05 | 6 | 18_41 | 8 | IE | P | 231 | 602.53 | 3.52 | 2027 | 123.09 | 4.96 | 37 | 327.28 | 3.36 | 0.20 | A | 6.00 | 3.95 | 1.32 |
| 2012 | 18_05 | 6 | 18_43 | 4 | IE | P | 365 | 610.69 | 2.82 | 1667 | 129.94 | 5.01 | 87 | 385.51 | 3.35 | 0.21 | A | 6.00 | 5.80 | 1.93 |
| 2012 | 18_05 | 6 | 18_43 | 4 | IE | P | 425 | 605.96 | 2.95 | 1541 | 128.89 | 4.62 | 113 | 395.63 | 3.90 | 0.21 | A | 6.00 | 6.42 | 2.14 |
| 2012 | 18_05 | 6 | 18_47 | 4 | IE | P | 417 | 599.05 | 2.91 | 1632 | 125.89 | 4.67 | 90 | 475.37 | 4.11 | 0.21 | A | 6.00 | 10.66 | 3.55 |
| 2012 | 18_05 | 6 | 18_47 | 4 | IE | P | 329 | 610.40 | 2.48 | 1267 | 129.43 | 4.69 | 54 | 382.50 | 2.84 | 0.21 | A | 6.00 | 5.73 | 1.91 |
| 2012 | 18_05 | 6 | 18_48 | 4 | IE | P | 361 | 613.49 | 2.66 | 1741 | 129.06 | 4.70 | 132 | 380.64 | 4.64 | 0.21 | A | 6.00 | 5.70 | 1.90 |
| 2012 | 18_05 | 6 | 18_48 | 4 | IE | P | 246 | 602.19 | 2.98 | 1727 | 127.26 | 4.73 | 100 | 368.49 | 3.42 | 0.21 | A | 6.00 | 5.37 | 1.79 |
| 2012 | 18_11 | 5 | 18_05 | 6 | IE | P | 362 | 595.99 | 2.14 | 1730 | 165.35 | 3.85 | 125 | 274.33 | 2.74 | 0.28 | AF | 7.70 |  |  |
| 2012 | 18_11 | 5 | 18_05 | 6 | IE | P | 387 | 601.33 | 1.84 | 1702 | 107.20 | 4.83 | 107 | 277.93 | 3.36 | 0.18 | A | 5.00 | 2.96 | 1.19 |
| 2012 | 18_11 | 5 | 18_23 | 7 | IE | P | 345 | 597.32 | 3.40 | 1782 | 106.11 | 4.94 | 105 | 347.75 | 5.13 | 0.18 | A | 5.00 | 6.39 | 2.55 |
| 2012 | 18_11 | 5 | 18_27 | 8 | IE | P | 205 | 603.41 | 3.59 | 2001 | 107.41 | 6.41 | 101 | 304.75 | 3.99 | 0.18 | A | 5.00 | 4.19 | 1.67 |
| 2012 | 18_11 | 5 | 18_32 | 6 | IE | P | 368 | 596.64 | 1.87 | 1435 | 107.98 | 4.79 | 116 | 347.12 | 3.87 | 0.18 | A | 5.00 | 6.07 | 2.43 |
| 2012 | 18_11 | 5 | 18_37 | 7 | IE | P | 412 | 599.94 | 2.92 | 1516 | 163.65 | 4.20 | 126 | 271.19 | 3.58 | 0.27 | AF | 7.57 |  |  |
| 2012 | 18_11 | 5 | 18_41 | 8 | IE | P | 233 | 613.76 | 3.13 | 1616 | 109.87 | 6.64 | 82 | 366.66 | 3.48 | 0.18 | A | 5.00 | 6.69 | 2.67 |
| 2012 | 18_14 | 4 | 18_01 | 6 | IE | P | 224 | 605.74 | 1.94 | 1852 | 155.38 | 3.70 | 147 | 249.71 | 3.87 | 0.26 | AF | 6.79 |  |  |
| 2012 | 18_14 | 4 | 18_01 | 6 | IE | P | 116 | 595.99 | 1.62 | 1357 | 89.90 | 5.14 | 86 | 240.52 | 3.29 | 0.15 | A | 4.00 | 2.70 | 1.35 |
| 2012 | 18_14 | 4 | 18_01 | 6 | IE | P | 288 | 599.23 | 2.09 | 2140 | 91.43 | 5.13 | 96 | 302.89 | 2.72 | 0.15 | A | 4.00 | 5.25 | 2.63 |
| 2012 | 18_14 | 4 | 18_01 | 6 | IE | P | 299 | 598.76 | 1.73 | 2030 | 90.41 | 5.31 | 117 | 252.29 | 4.22 | 0.15 | A | 4.00 | 3.16 | 1.58 |
| 2012 | 18_14 | 4 | 18_01 | 6 | IE | P | 267 | 602.91 | 1.97 | 1602 | 90.98 | 5.72 | 107 | 313.47 | 3.25 | 0.15 | A | 4.00 | 5.78 | 2.89 |
| 2012 | 18_14 | 4 | 18_01 | 6 | IE | P | 216 | 607.09 | 1.64 | 2060 | 93.67 | 5.02 | 130 | 253.92 | 3.22 | 0.15 | A | 4.00 | 2.84 | 1.42 |
| 2012 | 18_14 | 4 | 18_01 | 6 | IE | P | 263 | 597.87 | 2.19 | 1880 | 91.55 | 5.26 | 101 | 253.11 | 4.06 | 0.15 | A | 4.00 | 3.06 | 1.53 |
| 2012 | 18_14 | 4 | 18_01 | 6 | IE | P | 306 | 599.52 | 1.75 | 2190 | 92.03 | 5.36 | 138 | 253.80 | 4.51 | 0.15 | A | 4.00 | 3.03 | 1.52 |
| 2012 | 18_14 | 4 | 18_01 | 6 | IE | P | 297 | 598.85 | 1.70 | 2064 | 91.10 | 5.10 | 146 | 253.38 | 4.05 | 0.15 | A | 4.00 | 3.13 | 1.56 |
| 2012 | 18_14 | 4 | 18_01 | 6 | IE | P | 267 | 600.18 | 1.92 | 2084 | 92.19 | 5.60 | 128 | 305.57 | 4.14 | 0.15 | A | 4.00 | 5.26 | 2.63 |
| 2012 | 18_14 | 4 | 18_05 | 6 | IE | P | 234 | 625.91 | 3.01 | 2512 | 101.17 | 5.54 | 131 | 323.02 | 3.07 | 0.16 | A | 4.00 | 4.77 | 2.39 |
| 2012 | 18_14 | 4 | 18_05 | 6 | IE | P | 317 | 600.75 | 1.99 | 1962 | 89.42 | 5.00 | 121 | 244.25 | 4.35 | 0.15 | A | 4.00 | 2.93 | 1.46 |
| 2012 | 18_14 | 4 | 18_07 | 6 | IE | P | 256 | 601.58 | 2.01 | 1877 | 90.34 | 5.26 | 106 | 312.33 | 4.67 | 0.15 | A | 4.00 | 5.83 | 2.91 |
| 2012 | 18_14 | 4 | 18_11 | 5 | IE | P | 310 | 603.60 | 3.48 | 1820 | 97.85 | 5.87 | 143 | 325.41 | 3.05 | 0.16 | A | 4.00 | 5.30 | 2.65 |
| 2012 | 18_14 | 4 | 18_11 | 5 | IE | P | 199 | 605.82 | 2.24 | 1814 | 92.51 | 5.35 | 88 | 245.07 | 3.38 | 0.15 | A | 4.00 | 2.60 | 1.30 |
| 2012 | 18_14 | 4 | 18_11 | 5 | IE | P | 342 | 605.81 | 2.60 | 1923 | 92.89 | 5.97 | 135 | 291.88 | 3.83 | 0.15 | A | 4.00 | 4.57 | 2.28 |
| 2012 | 18_14 | 4 | 18_11 | 5 | IE | P | 176 | 600.67 | 2.22 | 2250 | 90.19 | 5.66 | 108 | 305.61 | 3.61 | 0.15 | A | 4.00 | 5.55 | 2.78 |
| 2012 | 18_14 | 4 | 18_11 | 5 | IE | P | 341 | 593.82 | 2.43 | 1989 | 89.91 | 5.33 | 120 | 276.44 | 5.87 | 0.15 | A | 4.00 | 4.30 | 2.15 |
| 2012 | 18_14 | 4 | 18_14 | 4 | S | P | 162 | 594.09 | 1.69 | 2172 | 90.58 | 6.04 | 148 | 275.24 | 3.46 | 0.15 | A | 4.00 | 4.15 | 2.08 |
| 2012 | 18_14 | 4 | 18_14 | 4 | S | P | 182 | 597.50 | 1.78 | 2052 | 90.65 | 5.64 | 64 | 352.98 | 3.37 | 0.15 | A | 4.00 | 7.58 | 3.79 |
| 2012 | 18_14 | 4 | 18_14 | 4 | S | P | 165 | 593.10 | 2.03 | 2040 | 88.20 | 5.64 | 114 | 266.01 | 3.67 | 0.15 | A | 4.00 | 4.06 | 2.03 |
| 2012 | 18_14 | 4 | 18_14 | 4 | S | P | 193 | 605.34 | 1.64 | 2272 | 92.07 | 5.63 | 112 | 279.81 | 3.67 | 0.15 | A | 4.00 | 4.16 | 2.08 |
| 2012 | 18_14 | 4 | 18_27 | 8 | IE | P | 297 | 605.98 | 2.44 | 1074 | 180.92 | 3.94 | 60 | 527.68 | 2.38 | 0.30 | AS | 7.91 |  |  |
| 2012 | 18_14 | 4 | 18_27 | 8 | IE | P | 177 | 601.12 | 2.19 | 2020 | 89.67 | 4.92 | 100 | 267.84 | 3.15 | 0.15 | A | 4.00 | 3.95 | 1.97 |
| 2012 | 18_14 | 4 | 18_27 | 8 | IE | P | 203 | 592.36 | 2.14 | 2115 | 85.94 | 5.48 | 132 | 264.62 | 3.29 | 0.15 | A | 4.00 | 4.32 | 2.16 |
| 2012 | 18_14 | 4 | 18_27 | 8 | IE | P | 207 | 603.79 | 2.00 | 2223 | 90.52 | 4.98 | 127 | 262.32 | 3.35 | 0.15 | A | 4.00 | 3.59 | 1.80 |
| 2012 | 18_14 | 4 | 18_27 | 8 | IE | P | 221 | 603.56 | 1.81 | 2323 | 90.02 | 4.76 | 106 | 256.06 | 3.29 | 0.15 | A | 4.00 | 3.38 | 1.69 |
| 2012 | 18_14 | 4 | 18_27 | 8 | IE | P | 297 | 600.51 | 1.82 | 2104 | 89.48 | 4.91 | 151 | 253.03 | 3.60 | 0.15 | A | 4.00 | 3.31 | 1.66 |
| 2012 | 18_14 | 4 | 18_27 | 8 | IE | P | 187 | 606.41 | 1.96 | 2281 | 91.04 | 5.10 | 87 | 281.20 | 2.69 | 0.15 | A | 4.00 | 4.36 | 2.18 |
| 2012 | 18_14 | 4 | 18_27 | 8 | IE | P | 263 | 597.69 | 1.75 | 2102 | 89.14 | 5.10 | 105 | 269.31 | 3.88 | 0.15 | A | 4.00 | 4.08 | 2.04 |
| 2012 | 18_14 | 4 | 18_32 | 6 | IE | P | 396 | 596.44 | 2.31 | 2803 | 88.26 | 5.07 | 154 | 306.17 | 4.21 | 0.15 | A | 4.00 | 5.88 | 2.94 |
| 2012 | 18_14 | 4 | 18_32 | 6 | IE | P | 310 | 598.90 | 1.92 | 1761 | 89.15 | 5.07 | 117 | 304.25 | 3.73 | 0.15 | A | 4.00 | 5.65 | 2.83 |
| 2012 | 18_14 | 4 | 18_32 | 6 | IE | P | 121 | 595.30 | 1.98 | 2225 | 89.06 | 4.82 | 126 | 298.44 | 4.73 | 0.15 | A | 4.00 | 5.40 | 2.70 |
| 2012 | 18_14 | 4 | 18_32 | 6 | IE | P | 191 | 592.18 | 2.23 | 2243 | 88.01 | 5.02 | 119 | 302.87 | 4.13 | 0.15 | A | 4.00 | 5.77 | 2.88 |
| 2012 | 18_14 | 4 | 18_32 | 6 | IE | P | 230 | 594.48 | 2.15 | 2269 | 87.81 | 5.19 | 74 | 303.32 | 3.49 | 0.15 | A | 4.00 | 5.82 | 2.91 |
| 2012 | 18_14 | 4 | 18_32 | 6 | IE | P | 247 | 592.06 | 2.04 | 1873 | 88.22 | 5.43 | 131 | 307.99 | 3.32 | 0.15 | A | 4.00 | 5.96 | 2.98 |
| 2012 | 18_14 | 4 | 18_32 | 6 | IE | P | 270 | 590.79 | 2.04 | 2232 | 87.82 | 4.95 | 123 | 304.42 | 3.39 | 0.15 | A | 4.00 | 5.87 | 2.93 |
| 2012 | 18_14 | 4 | 18_32 | 6 | IE | P | 214 | 593.58 | 1.92 | 1970 | 153.43 | 3.48 | 102 | 248.95 | 3.49 | 0.26 | AF | 6.85 |  |  |
| 2012 | 18_14 | 4 | 18_32 | 6 | IE | P | 236 | 588.49 | 1.92 | 2162 | 88.41 | 4.90 | 105 | 247.80 | 3.30 | 0.15 | A | 4.00 | 3.21 | 1.61 |
| 2012 | 18_14 | 4 | 18_36 | 5 | IE | P | 171 | 611.76 | 3.40 | 1049 | 94.88 | 5.58 | 94 | 316.97 | 5.73 | 0.16 | A | 4.00 | 5.36 | 2.68 |
| 2012 | 18_14 | 4 | 18_36 | 5 | IE | P | 311 | 598.68 | 2.28 | 1921 | 91.36 | 5.82 | 108 | 244.42 | 4.28 | 0.15 | A | 4.00 | 2.70 | 1.35 |
| 2012 | 18_14 | 4 | 18_36 | 5 | IE | P | 193 | 603.70 | 2.42 | 2111 | 91.77 | 6.51 | 90 | 458.44 | 4.04 | 0.15 | A3 | 4.00 |  |  |
| 2012 | 18_14 | 4 | 18_36 | 5 | IE | P | 218 | 605.99 | 2.35 | 2570 | 91.46 | 5.68 | 174 | 245.09 | 4.42 | 0.15 | A | 4.00 | 2.72 | 1.36 |
| 2012 | 18_14 | 4 | 18_36 | 5 | IE | P | 146 | 606.63 | 2.03 | 1933 | 91.83 | 6.05 | 162 | 250.80 | 4.27 | 0.15 | A | 4.00 | 2.92 | 1.46 |
| 2012 | 18_14 | 4 | 18_38 | 7 | IE | P | 215 | 597.73 | 1.93 | 2145 | 91.30 | 5.21 | 145 | 316.10 | 3.50 | 0.15 | A | 4.00 | 5.85 | 2.92 |
| 2012 | 18_14 | 4 | 18_38 | 7 | IE | P | 164 | 593.61 | 2.09 | 2239 | 90.22 | 6.33 | 105 | 318.68 | 3.37 | 0.15 | A | 4.00 | 6.13 | 3.06 |
| 2012 | 18_14 | 4 | 18_41 | 8 | IE | P | 194 | 598.77 | 1.82 | 2070 | 90.01 | 4.86 | 122 | 256.58 | 3.76 | 0.15 | A | 4.00 | 3.40 | 1.70 |
| 2012 | 18_14 | 4 | 18_41 | 8 | IE | P | 330 | 595.18 | 1.57 | 2318 | 89.47 | 4.95 | 142 | 261.51 | 2.89 | 0.15 | A | 4.00 | 3.69 | 1.85 |
| 2012 | 18_14 | 4 | 18_41 | 8 | IE | P | 240 | 595.16 | 2.01 | 1941 | 92.62 | 5.78 | 129 | 342.13 | 3.45 | 0.16 | A | 4.00 | 6.78 | 3.39 |
| 2012 | 18_14 | 4 | 18_41 | 8 | IE | P | 291 | 589.76 | 1.57 | 2267 | 89.98 | 5.37 | 65 | 438.78 | 3.70 | 0.15 | A | 4.00 | 11.51 | 5.75 |
| 2012 | 18_14 | 4 | 18_41 | 8 | IE | P | 277 | 600.95 | 1.72 | 2247 | 90.79 | 5.64 | 176 | 270.02 | 3.92 | 0.15 | A | 4.00 | 3.90 | 1.95 |
| 2012 | 18_14 | 4 | 18_41 | 8 | IE | P | 180 | 591.85 | 2.01 | 2058 | 88.16 | 5.22 | 94 | 308.44 | 2.93 | 0.15 | A | 4.00 | 5.99 | 3.00 |
| 2012 | 18_14 | 4 | 18_41 | 8 | IE | P | 363 | 591.06 | 2.01 | 1508 | 91.44 | 6.00 | 181 | 343.73 | 3.78 | 0.15 | A | 4.00 | 7.04 | 3.52 |
| 2012 | 18_14 | 4 | 18_41 | 8 | IE | P | 246 | 589.88 | 1.56 | 2179 | 88.21 | 5.37 | 110 | 254.04 | 4.28 | 0.15 | A | 4.00 | 3.52 | 1.76 |
| 2012 | 18_14 | 4 | 18_41 | 8 | IE | P | 171 | 595.62 | 1.79 | 2275 | 88.13 | 5.05 | 124 | 256.30 | 2.60 | 0.15 | A | 4.00 | 3.63 | 1.82 |
| 2012 | 18_14 | 4 | 18_41 | 8 | IE | P | 300 | 593.39 | 2.43 | 2057 | 89.67 | 5.30 | 67 | 427.46 | 3.15 | 0.15 | A | 4.00 | 11.07 | 5.53 |
| 2012 | 18_14 | 4 | 18_43 | 4 | IA | P | 342 | 610.30 | 3.74 | 1858 | 94.81 | 5.89 | 124 | 269.19 | 4.02 | 0.16 | A | 4.00 | 3.36 | 1.68 |
| 2012 | 18_14 | 4 | 18_43 | 4 | IA | P | 294 | 623.82 | 4.11 | 1727 | 94.58 | 6.19 | 100 | 269.28 | 4.26 | 0.15 | A | 4.00 | 3.39 | 1.69 |
| 2012 | 18_14 | 4 | 18_44 | 8 | IE | P | 193 | 603.72 | 2.00 | 2159 | 93.35 | 5.74 | 182 | 431.89 | 3.34 | 0.15 | A | 4.00 | 10.51 | 5.25 |
| 2012 | 18_15 | 4 | 18_01 | 6 | IE | P | 264 | 593.07 | 2.08 | 2132 | 88.37 | 5.19 | 125 | 292.74 | 3.75 | 0.15 | A | 4.00 | 5.25 | 2.63 |
| 2012 | 18_15 | 4 | 18_01 | 6 | IE | P | 218 | 596.79 | 2.42 | 1966 | 87.83 | 5.74 | 93 | 301.51 | 3.45 | 0.15 | A | 4.00 | 5.73 | 2.87 |
| 2012 | 18_15 | 4 | 18_01 | 6 | IE | P | 336 | 596.74 | 2.02 | 1757 | 89.92 | 5.47 | 95 | 302.84 | 3.71 | 0.15 | A | 4.00 | 5.47 | 2.74 |
| 2012 | 18_15 | 4 | 18_02 | 6 | IE | P | 433 | 596.28 | 1.80 | 1827 | 87.66 | 5.32 | 118 | 237.89 | 3.79 | 0.15 | A | 4.00 | 2.86 | 1.43 |
| 2012 | 18_15 | 4 | 18_02 | 6 | IE | P | 362 | 604.26 | 2.30 | 1301 | 91.97 | 5.86 | 84 | 246.93 | 5.19 | 0.15 | A | 4.00 | 2.74 | 1.37 |
| 2012 | 18_15 | 4 | 18_02 | 6 | IE | P | 282 | 600.96 | 1.93 | 2051 | 88.57 | 5.29 | 118 | 297.60 | 4.71 | 0.15 | A | 4.00 | 5.44 | 2.72 |
| 2012 | 18_15 | 4 | 18_05 | 6 | IE | P | 399 | 594.92 | 2.10 | 1934 | 88.73 | 5.40 | 125 | 239.57 | 3.87 | 0.15 | A | 4.00 | 2.80 | 1.40 |
| 2012 | 18_15 | 4 | 18_05 | 6 | IE | P | 316 | 598.31 | 2.04 | 2002 | 89.60 | 5.66 | 133 | 244.32 | 3.67 | 0.15 | A | 4.00 | 2.91 | 1.45 |
| 2012 | 18_15 | 4 | 18_05 | 6 | IE | P | 268 | 598.71 | 1.86 | 2150 | 89.52 | 5.32 | 108 | 244.53 | 4.23 | 0.15 | A | 4.00 | 2.93 | 1.46 |
| 2012 | 18_15 | 4 | 18_05 | 6 | IE | P | 317 | 597.13 | 2.11 | 1907 | 89.22 | 5.84 | 93 | 342.21 | 4.21 | 0.15 | A | 4.00 | 7.34 | 3.67 |
| 2012 | 18_15 | 4 | 18_07 | 6 | IE | P | 193 | 597.43 | 2.24 | 2060 | 89.73 | 5.77 | 75 | 306.14 | 3.53 | 0.15 | A | 4.00 | 5.65 | 2.82 |
| 2012 | 18_15 | 4 | 18_07 | 6 | IE | P | 291 | 596.94 | 2.60 | 2207 | 88.62 | 5.87 | 146 | 243.23 | 3.73 | 0.15 | A | 4.00 | 2.98 | 1.49 |
| 2012 | 18_15 | 4 | 18_07 | 6 | IE | P | 194 | 600.66 | 2.31 | 2303 | 90.47 | 5.78 | 68 | 405.80 | 3.89 | 0.15 | A | 4.00 | 9.94 | 4.97 |
| 2012 | 18_15 | 4 | 18_11 | 5 | IE | P | 158 | 592.08 | 2.01 | 2171 | 89.74 | 5.24 | 125 | 284.16 | 3.72 | 0.15 | A | 4.00 | 4.67 | 2.33 |
| 2012 | 18_15 | 4 | 18_15 | 4 | S | P | 282 | 587.72 | 3.45 | 1987 | 90.98 | 6.65 | 127 | 269.32 | 3.66 | 0.15 | A | 4.00 | 3.84 | 1.92 |
| 2012 | 18_15 | 4 | 18_15 | 4 | S | P | 276 | 602.09 | 3.60 | 1874 | 93.75 | 6.81 | 94 | 247.15 | 4.78 | 0.16 | A | 4.00 | 2.55 | 1.27 |
| 2012 | 18_15 | 4 | 18_15 | 4 | S | P | 281 | 602.52 | 3.64 | 2240 | 92.86 | 6.67 | 122 | 282.79 | 5.82 | 0.15 | A | 4.00 | 4.18 | 2.09 |
| 2012 | 18_15 | 4 | 18_15 | 4 | S | P | 238 | 592.80 | 3.00 | 1950 | 86.23 | 6.25 | 139 | 252.62 | 3.94 | 0.15 | A | 4.00 | 3.72 | 1.86 |
| 2012 | 18_15 | 4 | 18_15 | 4 | S | P | 325 | 591.77 | 3.73 | 1848 | 86.80 | 6.25 | 163 | 257.43 | 4.19 | 0.15 | A | 4.00 | 3.86 | 1.93 |
| 2012 | 18_15 | 4 | 18_23 | 7 | IE | P | 185 | 600.16 | 2.34 | 2070 | 90.72 | 5.49 | 115 | 266.99 | 3.33 | 0.15 | A | 4.00 | 3.77 | 1.89 |
| 2012 | 18_15 | 4 | 18_23 | 7 | IE | P | 306 | 595.51 | 2.16 | 2131 | 90.01 | 5.45 | 132 | 311.53 | 3.47 | 0.15 | A | 4.00 | 5.84 | 2.92 |
| 2012 | 18_15 | 4 | 18_25 | 5 | IE | P | 245 | 595.51 | 2.18 | 2011 | 89.96 | 5.21 | 140 | 279.11 | 3.69 | 0.15 | A | 4.00 | 4.41 | 2.21 |
| 2012 | 18_15 | 4 | 18_29 | 5 | IE | P | 346 | 610.83 | 3.08 | 1671 | 93.31 | 5.67 | 99 | 279.48 | 3.27 | 0.15 | A | 4.00 | 3.98 | 1.99 |
| 2012 | 18_15 | 4 | 18_32 | 6 | IE | P | 249 | 608.16 | 2.32 | 1854 | 93.04 | 5.47 | 206 | 318.45 | 3.95 | 0.15 | A | 4.00 | 5.69 | 2.85 |
| 2012 | 18_15 | 4 | 18_32 | 6 | IE | P | 174 | 592.86 | 2.41 | 1969 | 89.09 | 5.62 | 77 | 385.20 | 4.71 | 0.15 | A | 4.00 | 9.29 | 4.65 |
| 2012 | 18_15 | 4 | 18_36 | 5 | IE | P | 482 | 625.45 | 3.63 | 1806 | 95.98 | 6.24 | 93 | 307.73 | 4.35 | 0.15 | A | 4.00 | 4.82 | 2.41 |
| 2012 | 18_15 | 4 | 18_37 | 7 | IE | P | 203 | 598.95 | 3.15 | 1983 | 90.92 | 6.17 | 103 | 327.46 | 3.72 | 0.15 | A | 4.00 | 6.41 | 3.20 |
| 2012 | 18_15 | 4 | 18_37 | 7 | IE | P | 190 | 598.40 | 2.84 | 1926 | 92.96 | 6.23 | 142 | 320.81 | 3.87 | 0.16 | A | 4.00 | 5.80 | 2.90 |
| 2012 | 18_15 | 4 | 18_38 | 7 | IE | P | 253 | 601.34 | 2.40 | 2135 | 90.79 | 6.36 | 145 | 252.25 | 3.38 | 0.15 | A | 4.00 | 3.11 | 1.56 |
| 2012 | 18_15 | 4 | 18_38 | 7 | IE | P | 253 | 601.34 | 2.40 | 2135 | 90.79 | 6.36 | 63 | 433.90 | 3.24 | 0.15 | A | 4.00 | 11.12 | 5.56 |
| 2012 | 18_15 | 4 | 18_41 | 8 | IE | P | 496 | 603.14 | 2.03 | 1571 | 178.68 | 4.31 | 145 | 269.33 | 4.18 | 0.30 | AF | 7.85 |  |  |
| 2012 | 18_15 | 4 | 18_41 | 8 | IE | P | 172 | 603.18 | 1.80 | 2184 | 91.22 | 5.82 | 127 | 263.62 | 4.29 | 0.15 | A | 4.00 | 3.56 | 1.78 |
| 2012 | 18_15 | 4 | 18_41 | 8 | IE | P | 361 | 603.32 | 2.24 | 1958 | 90.45 | 5.86 | 136 | 260.08 | 4.35 | 0.15 | A | 4.00 | 3.50 | 1.75 |
| 2012 | 18_15 | 4 | 18_44 | 8 | IE | P | 124 | 597.00 | 2.04 | 2134 | 90.52 | 5.96 | 149 | 333.76 | 3.01 | 0.15 | A | 4.00 | 6.75 | 3.37 |
| 2012 | 18_15 | 4 | 18_44 | 8 | IE | P | 495 | 602.65 | 2.91 | 1865 | 92.51 | 6.58 | 117 | 331.07 | 4.99 | 0.15 | A | 4.00 | 6.31 | 3.16 |
| 2012 | 18_15 | 4 | 18_44 | 8 | IE | P | 252 | 592.01 | 2.37 | 1832 | 87.27 | 6.19 | 185 | 257.50 | 3.77 | 0.15 | A | 4.00 | 3.80 | 1.90 |
| 2012 | 18_20 | 8 | 18_01 | 6 | IE | L | 204 | 713.49 | 2.71 | 1935 | 126.17 | 4.49 | 131 | 347.22 | 4.06 | 0.18 | A | 8.00 | 6.02 | 1.50 |
| 2012 | 18_23 | 7 | 18_01 | 6 | IE | P | 344 | 594.68 | 2.58 | 1600 | 148.04 | 4.38 | 94 | 422.53 | 3.55 | 0.25 | A | 7.00 | 5.98 | 1.71 |
| 2012 | 18_23 | 7 | 18_01 | 6 | IE | P | 282 | 600.45 | 2.58 | 1936 | 149.61 | 5.00 | 68 | 385.80 | 4.17 | 0.25 | A | 7.00 | 4.05 | 1.16 |
| 2012 | 18_23 | 7 | 18_01 | 6 | IE | P | 268 | 600.11 | 2.33 | 1874 | 79.13 | 6.59 | 108 | 227.24 | 3.87 | 0.13 | HP | 3.72 |  |  |
| 2012 | 18_23 | 7 | 18_01 | 6 | IE | P | 162 | 589.91 | 2.30 | 1474 | 146.09 | 4.87 | 52 | 433.51 | 4.32 | 0.25 | A | 7.00 | 6.77 | 1.93 |
| 2012 | 18_23 | 7 | 18_07 | 6 | IE | P | 380 | 601.77 | 3.29 | 1690 | 151.94 | 5.82 | 89 | 447.93 | 4.15 | 0.25 | A | 7.00 | 6.64 | 1.90 |
| 2012 | 18_23 | 7 | 18_11 | 5 | IE | P | 214 | 599.41 | 2.13 | 1839 | 146.89 | 4.89 | 82 | 393.99 | 3.50 | 0.25 | A | 7.00 | 4.78 | 1.36 |
| 2012 | 18_23 | 7 | 18_23 | 7 | S | P | 666 | 597.03 | 3.62 | 1076 | 151.89 | 5.52 | 87 | 433.11 | 4.88 | 0.25 | A | 7.00 | 5.96 | 1.70 |
| 2012 | 18_23 | 7 | 18_27 | 8 | IE | P | 438 | 604.52 | 2.08 | 1651 | 149.76 | 4.38 | 113 | 443.07 | 3.90 | 0.25 | A | 7.00 | 6.71 | 1.92 |
| 2012 | 18_23 | 7 | 18_27 | 8 | IE | P | 654 | 598.65 | 3.00 | 1210 | 151.07 | 5.13 | 90 | 442.45 | 3.76 | 0.25 | A | 7.00 | 6.50 | 1.86 |
| 2012 | 18_23 | 7 | 18_27 | 8 | IE | P | 666 | 601.56 | 2.27 | 1429 | 148.50 | 4.36 | 87 | 387.94 | 3.22 | 0.25 | A | 7.00 | 4.29 | 1.22 |
| 2012 | 18_23 | 7 | 18_27 | 8 | IE | P | 740 | 604.24 | 2.35 | 1173 | 149.81 | 5.08 | 101 | 367.63 | 4.86 | 0.25 | A | 7.00 | 3.18 | 0.91 |
| 2012 | 18_23 | 7 | 18_27 | 8 | IE | P | 548 | 603.79 | 2.27 | 1292 | 152.63 | 4.93 | 91 | 452.44 | 3.92 | 0.25 | A | 7.00 | 6.75 | 1.93 |
| 2012 | 18_23 | 7 | 18_27 | 8 | IE | P | 566 | 598.24 | 2.40 | 1302 | 146.38 | 4.23 | 117 | 444.82 | 3.63 | 0.24 | A | 7.00 | 7.27 | 2.08 |
| 2012 | 18_23 | 7 | 18_27 | 8 | IE | P | 492 | 603.74 | 2.17 | 1321 | 148.86 | 4.11 | 100 | 437.18 | 3.69 | 0.25 | A | 7.00 | 6.56 | 1.87 |
| 2012 | 18_23 | 7 | 18_27 | 8 | IE | P | 696 | 603.18 | 2.14 | 1334 | 153.59 | 4.11 | 93 | 448.07 | 4.29 | 0.25 | A | 7.00 | 6.42 | 1.83 |
| 2012 | 18_23 | 7 | 18_27 | 8 | IE | P | 390 | 604.98 | 2.06 | 1241 | 148.45 | 3.68 | 84 | 443.35 | 3.72 | 0.25 | A | 7.00 | 6.91 | 1.97 |
| 2012 | 18_23 | 7 | 18_27 | 8 | IE | P | 583 | 595.63 | 2.30 | 1237 | 145.09 | 4.00 | 87 | 456.34 | 4.34 | 0.24 | A | 7.00 | 8.02 | 2.29 |
| 2012 | 18_23 | 7 | 18_38 | 7 | IA | P | 372 | 611.03 | 3.38 | 1763 | 152.39 | 5.48 | 81 | 444.06 | 3.59 | 0.25 | A | 7.00 | 6.40 | 1.83 |
| 2012 | 18_23 | 7 | 18_44 | 8 | IE | P | 222 | 596.79 | 1.94 | 1755 | 150.65 | 4.14 | 91 | 449.01 | 3.06 | 0.25 | A | 7.00 | 6.86 | 1.96 |
| 2012 | 18_23 | 7 | 18_44 | 8 | IE | P | 414 | 592.42 | 2.29 | 1072 | 223.02 | 4.51 | 152 | 366.76 | 3.57 | 0.38 | AF | 10.63 |  |  |
| 2012 | 18_23 | 7 | 18_44 | 8 | IE | P | 388 | 587.37 | 2.32 | 1689 | 146.66 | 4.96 | 76 | 380.14 | 3.02 | 0.25 | A | 7.00 | 4.14 | 1.18 |
| 2012 | 18_23 | 7 | 18_44 | 8 | IE | P | 293 | 593.24 | 2.34 | 1705 | 145.21 | 4.30 | 84 | 420.25 | 2.89 | 0.24 | A | 7.00 | 6.26 | 1.79 |
| 2012 | 18_23 | 7 | 18_44 | 8 | IE | P | 442 | 592.47 | 2.24 | 892 | 223.12 | 4.08 | 98 | 368.52 | 3.31 | 0.38 | AF | 10.64 |  |  |
| 2012 | 18_23 | 7 | 18_45 | 5 | IE | P | 323 | 606.63 | 2.80 | 1100 | 147.60 | 4.72 | 59 | 420.03 | 3.13 | 0.24 | A | 7.00 | 5.92 | 1.69 |
| 2012 | 18_23 | 7 | 18_45 | 5 | IE | P | 300 | 605.77 | 2.49 | 1794 | 146.68 | 4.36 | 105 | 435.30 | 3.41 | 0.24 | A | 7.00 | 6.77 | 1.94 |
| 2012 | 18_23 | 7 | 18_47 | 4 | IE | P | 339 | 596.05 | 3.04 | 1744 | 146.44 | 4.68 | 119 | 458.64 | 3.54 | 0.25 | A | 7.00 | 7.92 | 2.26 |
| 2012 | 18_25 | 5 | 18_05 | 6 | IE | P | 212 | 588.03 | 1.99 | 1839 | 104.37 | 4.60 | 84 | 277.21 | 3.18 | 0.18 | A | 5.00 | 3.28 | 1.31 |
| 2012 | 18_25 | 5 | 18_36 | 5 | IA | P | 323 | 607.64 | 3.65 | 1508 | 111.84 | 7.01 | 118 | 274.65 | 4.99 | 0.18 | A | 5.00 | 2.28 | 0.91 |
| 2012 | 18_25 | 5 | 18_36 | 5 | IA | P | 312 | 608.85 | 3.73 | 1839 | 109.28 | 6.26 | 95 | 274.52 | 4.35 | 0.18 | A | 5.00 | 2.56 | 1.02 |
| 2012 | 18_25 | 5 | 18_37 | 7 | IE | P | 306 | 604.70 | 2.94 | 1959 | 106.88 | 5.06 | 107 | 358.44 | 3.55 | 0.18 | A | 5.00 | 6.77 | 2.71 |
| 2012 | 18_25 | 5 | 18_44 | 8 | IE | P | 282 | 591.20 | 3.15 | 1411 | 104.72 | 6.00 | 121 | 340.27 | 4.25 | 0.18 | A | 5.00 | 6.25 | 2.50 |
| 2012 | 18_27 | 8 | 18_01 | 6 | IE | L | 253 | 720.86 | 2.87 | 1735 | 128.74 | 4.61 | 75 | 350.67 | 3.72 | 0.18 | A | 8.00 | 5.79 | 1.45 |
| 2012 | 18_27 | 8 | 18_01 | 6 | IE | L | 167 | 709.87 | 3.20 | 1813 | 125.21 | 5.00 | 87 | 346.03 | 3.30 | 0.18 | A | 8.00 | 6.11 | 1.53 |
| 2012 | 18_27 | 8 | 18_01 | 6 | IE | L | 223 | 708.52 | 3.53 | 1601 | 126.49 | 5.05 | 64 | 352.54 | 4.29 | 0.18 | A | 8.00 | 6.30 | 1.57 |
| 2012 | 18_27 | 8 | 18_01 | 6 | IE | L | 263 | 707.30 | 2.96 | 1718 | 126.48 | 4.82 | 128 | 352.62 | 3.85 | 0.18 | A | 8.00 | 6.30 | 1.58 |
| 2012 | 18_27 | 8 | 18_01 | 6 | IE | L | 133 | 705.31 | 3.13 | 1900 | 126.04 | 5.02 | 89 | 325.47 | 5.19 | 0.18 | A | 8.00 | 4.66 | 1.16 |
| 2012 | 18_27 | 8 | 18_01 | 6 | IE | L | 165 | 689.15 | 3.18 | 1636 | 123.41 | 4.68 | 75 | 339.01 | 4.82 | 0.18 | A | 8.00 | 5.98 | 1.49 |
| 2012 | 18_27 | 8 | 18_01 | 6 | IE | L | 158 | 700.79 | 3.28 | 1617 | 123.73 | 4.69 | 73 | 334.47 | 3.72 | 0.18 | A | 8.00 | 5.63 | 1.41 |
| 2012 | 18_27 | 8 | 18_02 | 6 | IE | L | 285 | 705.31 | 3.06 | 1708 | 125.12 | 5.36 | 89 | 352.74 | 4.48 | 0.18 | A | 8.00 | 6.55 | 1.64 |
| 2012 | 18_27 | 8 | 18_02 | 6 | IE | L | 135 | 711.91 | 3.73 | 2003 | 126.89 | 5.03 | 89 | 350.62 | 4.52 | 0.18 | A | 8.00 | 6.11 | 1.53 |
| 2012 | 18_27 | 8 | 18_02 | 6 | IE | L | 175 | 695.66 | 3.42 | 2072 | 122.02 | 5.02 | 80 | 341.02 | 3.22 | 0.18 | A | 8.00 | 6.36 | 1.59 |
| 2012 | 18_27 | 8 | 18_02 | 6 | IE | L | 106 | 703.56 | 2.68 | 1727 | 120.51 | 5.04 | 80 | 336.83 | 4.19 | 0.17 | A | 8.00 | 6.36 | 1.59 |
| 2012 | 18_27 | 8 | 18_02 | 6 | IE | L | 216 | 717.65 | 3.56 | 2120 | 124.97 | 5.34 | 92 | 345.95 | 4.67 | 0.17 | A | 8.00 | 6.15 | 1.54 |
| 2012 | 18_27 | 8 | 18_02 | 6 | IE | L | 254 | 716.47 | 3.00 | 1759 | 126.80 | 5.19 | 74 | 351.69 | 3.06 | 0.18 | A | 8.00 | 6.19 | 1.55 |
| 2012 | 18_27 | 8 | 18_02 | 6 | IE | L | 177 | 698.45 | 3.49 | 1939 | 121.19 | 4.75 | 99 | 331.47 | 3.21 | 0.17 | A | 8.00 | 5.88 | 1.47 |
| 2012 | 18_27 | 8 | 18_05 | 6 | IE | L | 223 | 699.61 | 3.46 | 1748 | 124.06 | 4.64 | 101 | 340.97 | 3.20 | 0.18 | A | 8.00 | 5.99 | 1.50 |
| 2012 | 18_27 | 8 | 18_05 | 6 | IE | L | 285 | 703.60 | 3.09 | 1677 | 125.07 | 4.99 | 72 | 350.30 | 3.49 | 0.18 | A | 8.00 | 6.41 | 1.60 |
| 2012 | 18_27 | 8 | 18_07 | 6 | IE | L | 224 | 695.64 | 3.04 | 2023 | 126.84 | 4.60 | 142 | 366.81 | 3.61 | 0.18 | A | 8.00 | 7.14 | 1.78 |
| 2012 | 18_27 | 8 | 18_07 | 6 | IE | L | 203 | 706.66 | 2.60 | 1463 | 128.51 | 4.62 | 85 | 350.18 | 4.06 | 0.18 | A | 8.00 | 5.80 | 1.45 |
| 2012 | 18_27 | 8 | 18_07 | 6 | IE | L | 220 | 706.77 | 2.81 | 1620 | 122.96 | 4.88 | 88 | 345.60 | 4.42 | 0.17 | A | 8.00 | 6.49 | 1.62 |
| 2012 | 18_27 | 8 | 18_23 | 7 | IE | L | 270 | 711.75 | 3.12 | 2040 | 128.13 | 4.97 | 86 | 355.55 | 3.69 | 0.18 | A | 8.00 | 6.20 | 1.55 |
| 2012 | 18_27 | 8 | 18_23 | 7 | IE | L | 115 | 698.23 | 3.45 | 2105 | 123.16 | 5.10 | 62 | 346.00 | 3.88 | 0.18 | A | 8.00 | 6.47 | 1.62 |
| 2012 | 18_27 | 8 | 18_23 | 7 | IE | L | 111 | 701.64 | 2.79 | 1540 | 122.07 | 4.55 | 86 | 344.14 | 3.44 | 0.17 | A | 8.00 | 6.55 | 1.64 |
| 2012 | 18_27 | 8 | 18_23 | 7 | IE | L | 170 | 724.08 | 2.49 | 1689 | 128.60 | 5.45 | 77 | 355.38 | 3.17 | 0.18 | A | 8.00 | 6.11 | 1.53 |
| 2012 | 18_27 | 8 | 18_23 | 7 | IE | L | 169 | 716.86 | 2.93 | 1544 | 127.79 | 4.39 | 76 | 359.40 | 3.76 | 0.18 | A | 8.00 | 6.50 | 1.62 |
| 2012 | 18_27 | 8 | 18_23 | 7 | IE | L | 157 | 686.08 | 2.85 | 1650 | 122.03 | 4.61 | 55 | 336.23 | 3.79 | 0.18 | A | 8.00 | 6.04 | 1.51 |
| 2012 | 18_27 | 8 | 18_27 | 8 | S | L | 154 | 718.25 | 3.48 | 2220 | 125.56 | 4.54 | 60 | 346.91 | 3.84 | 0.17 | A | 8.00 | 6.10 | 1.53 |
| 2012 | 18_27 | 8 | 18_27 | 8 | S | L | 231 | 702.28 | 3.19 | 1864 | 124.60 | 5.12 | 65 | 366.78 | 4.51 | 0.18 | A | 8.00 | 7.55 | 1.89 |
| 2012 | 18_27 | 8 | 18_27 | 8 | S | L | 203 | 695.69 | 3.02 | 2044 | 123.11 | 4.95 | 68 | 354.48 | 4.96 | 0.18 | A | 8.00 | 7.04 | 1.76 |
| 2012 | 18_27 | 8 | 18_27 | 8 | S | L | 199 | 706.01 | 3.25 | 1630 | 125.91 | 5.09 | 56 | 375.59 | 4.47 | 0.18 | A | 8.00 | 7.86 | 1.97 |
| 2012 | 18_27 | 8 | 18_29 | 5 | IE | L | 137 | 713.92 | 2.39 | 1776 | 126.72 | 4.84 | 92 | 370.90 | 3.77 | 0.18 | A | 8.00 | 7.42 | 1.85 |
| 2012 | 18_27 | 8 | 18_29 | 5 | IE | L | 164 | 699.24 | 2.44 | 2030 | 125.59 | 4.87 | 101 | 316.43 | 4.41 | 0.18 | A | 8.00 | 4.16 | 1.04 |
| 2012 | 18_27 | 8 | 18_32 | 6 | IE | L | 187 | 693.76 | 2.72 | 1358 | 128.52 | 4.76 | 60 | 315.08 | 3.03 | 0.19 | A | 8.00 | 3.61 | 0.90 |
| 2012 | 18_27 | 8 | 18_32 | 6 | IE | L | 237 | 717.63 | 2.81 | 1479 | 129.58 | 4.57 | 68 | 358.85 | 3.82 | 0.18 | A | 8.00 | 6.15 | 1.54 |
| 2012 | 18_27 | 8 | 18_32 | 6 | IE | L | 438 | 696.82 | 3.88 | 2006 | 125.55 | 5.30 | 68 | 346.83 | 4.39 | 0.18 | A | 8.00 | 6.10 | 1.52 |
| 2012 | 18_27 | 8 | 18_37 | 7 | IE | L | 201 | 707.70 | 3.42 | 1998 | 125.11 | 5.09 | 118 | 349.16 | 4.49 | 0.18 | A | 8.00 | 6.33 | 1.58 |
| 2012 | 18_27 | 8 | 18_37 | 7 | IE | L | 192 | 700.94 | 3.18 | 1660 | 125.31 | 5.00 | 59 | 357.88 | 3.11 | 0.18 | A | 8.00 | 6.85 | 1.71 |
| 2012 | 18_27 | 8 | 18_37 | 7 | IE | L | 96 | 700.10 | 2.77 | 1907 | 124.28 | 5.49 | 98 | 352.35 | 3.62 | 0.18 | A | 8.00 | 6.68 | 1.67 |
| 2012 | 18_27 | 8 | 18_37 | 7 | IE | L | 151 | 699.20 | 2.87 | 1695 | 124.30 | 4.94 | 109 | 346.53 | 4.57 | 0.18 | A | 8.00 | 6.30 | 1.58 |
| 2012 | 18_27 | 8 | 18_37 | 7 | IE | L | 197 | 707.75 | 3.50 | 1655 | 125.27 | 4.85 | 86 | 352.14 | 3.37 | 0.18 | A | 8.00 | 6.49 | 1.62 |
| 2012 | 18_27 | 8 | 18_37 | 7 | IE | L | 158 | 712.80 | 4.03 | 1585 | 128.71 | 4.98 | 111 | 353.20 | 3.98 | 0.18 | A | 8.00 | 5.95 | 1.49 |
| 2012 | 18_27 | 8 | 18_38 | 7 | IE | L | 247 | 720.99 | 2.75 | 1741 | 127.90 | 4.84 | 118 | 366.57 | 4.36 | 0.18 | A | 8.00 | 6.93 | 1.73 |
| 2012 | 18_27 | 8 | 18_38 | 7 | IE | L | 270 | 703.95 | 3.40 | 1698 | 125.24 | 5.42 | 108 | 360.16 | 4.00 | 0.18 | A | 8.00 | 7.01 | 1.75 |
| 2012 | 18_27 | 8 | 18_38 | 7 | IE | L | 206 | 710.21 | 3.21 | 2062 | 128.40 | 4.56 | 92 | 357.77 | 4.51 | 0.18 | A | 8.00 | 6.29 | 1.57 |
| 2012 | 18_27 | 8 | 18_38 | 7 | IE | L | 183 | 700.46 | 3.01 | 1709 | 126.04 | 5.32 | 100 | 313.28 | 4.42 | 0.18 | A | 8.00 | 3.88 | 0.97 |
| 2012 | 18_27 | 8 | 18_44 | 8 | IA | L | 208 | 709.33 | 3.21 | 1766 | 126.91 | 4.67 | 100 | 358.64 | 3.46 | 0.18 | A | 8.00 | 6.61 | 1.65 |
| 2012 | 18_27 | 8 | 18_48 | 4 | IE | L | 327 | 700.08 | 3.78 | 2229 | 127.40 | 5.20 | 64 | 307.52 | 4.46 | 0.18 | A | 8.00 | 3.31 | 0.83 |
| 2012 | 18_27 | 8 | 18_48 | 4 | IE | L | 188 | 721.09 | 3.29 | 1695 | 128.82 | 5.17 | 109 | 362.48 | 4.16 | 0.18 | A | 8.00 | 6.51 | 1.63 |
| 2012 | 18_27 | 8 | 18_48 | 4 | IE | L | 255 | 697.03 | 4.10 | 1833 | 125.66 | 4.98 | 55 | 316.94 | 3.68 | 0.18 | A | 8.00 | 4.18 | 1.04 |
| 2012 | 18_29 | 5 | 18_23 | 7 | IE | P | 328 | 600.41 | 3.22 | 1835 | 106.11 | 5.05 | 93 | 287.79 | 5.70 | 0.18 | A | 5.00 | 3.56 | 1.42 |
| 2012 | 18_29 | 5 | 18_29 | 5 | S | P | 222 | 600.92 | 2.39 | 1809 | 109.22 | 5.65 | 104 | 346.33 | 3.29 | 0.18 | A | 5.00 | 5.85 | 2.34 |
| 2012 | 18_29 | 5 | 18_36 | 5 | IA | P | 215 | 607.13 | 4.20 | 1523 | 111.90 | 7.04 | 117 | 335.54 | 4.49 | 0.18 | A | 5.00 | 4.99 | 2.00 |
| 2012 | 18_32 | 6 | 18_01 | 6 | IA | P | 164 | 600.70 | 3.06 | 2038 | 127.97 | 5.00 | 125 | 380.98 | 3.63 | 0.21 | A | 6.00 | 5.86 | 1.95 |
| 2012 | 18_32 | 6 | 18_01 | 6 | IA | P | 160 | 600.41 | 2.20 | 1901 | 127.23 | 4.58 | 81 | 382.49 | 3.82 | 0.21 | A | 6.00 | 6.04 | 2.01 |
| 2012 | 18_32 | 6 | 18_01 | 6 | IA | P | 205 | 596.78 | 2.22 | 2268 | 125.97 | 4.35 | 106 | 380.41 | 3.16 | 0.21 | A | 6.00 | 6.12 | 2.04 |
| 2012 | 18_32 | 6 | 18_01 | 6 | IA | P | 191 | 599.83 | 2.14 | 2072 | 126.72 | 4.44 | 109 | 382.48 | 3.24 | 0.21 | A | 6.00 | 6.11 | 2.04 |
| 2012 | 18_32 | 6 | 18_01 | 6 | IA | P | 164 | 598.59 | 2.21 | 2037 | 126.27 | 4.10 | 111 | 375.69 | 3.89 | 0.21 | A | 6.00 | 5.85 | 1.95 |
| 2012 | 18_32 | 6 | 18_01 | 6 | IA | P | 128 | 596.67 | 2.10 | 2253 | 125.78 | 4.18 | 109 | 368.41 | 3.51 | 0.21 | A | 6.00 | 5.57 | 1.86 |
| 2012 | 18_32 | 6 | 18_01 | 6 | IA | P | 202 | 594.13 | 2.18 | 2131 | 126.01 | 4.45 | 84 | 374.21 | 2.92 | 0.21 | A | 6.00 | 5.82 | 1.94 |
| 2012 | 18_32 | 6 | 18_01 | 6 | IA | P | 154 | 597.88 | 2.51 | 1838 | 126.24 | 4.56 | 79 | 386.70 | 3.05 | 0.21 | A | 6.00 | 6.38 | 2.13 |
| 2012 | 18_32 | 6 | 18_01 | 6 | IA | P | 196 | 605.73 | 2.35 | 2066 | 128.59 | 3.83 | 97 | 387.04 | 3.40 | 0.21 | A | 6.00 | 6.06 | 2.02 |
| 2012 | 18_32 | 6 | 18_05 | 6 | IA | P | 224 | 602.55 | 2.32 | 1911 | 131.65 | 4.27 | 140 | 384.11 | 3.55 | 0.22 | A | 6.00 | 5.51 | 1.84 |
| 2012 | 18_32 | 6 | 18_07 | 6 | IA | P | 188 | 609.79 | 2.17 | 2164 | 130.75 | 4.34 | 101 | 394.65 | 3.09 | 0.21 | A | 6.00 | 6.11 | 2.04 |
| 2012 | 18_32 | 6 | 18_07 | 6 | IA | P | 238 | 602.61 | 2.17 | 2134 | 129.19 | 4.56 | 115 | 407.99 | 3.82 | 0.21 | A | 6.00 | 6.95 | 2.32 |
| 2012 | 18_32 | 6 | 18_07 | 6 | IA | P | 249 | 593.98 | 2.07 | 1671 | 129.15 | 4.60 | 86 | 389.27 | 2.93 | 0.22 | A | 6.00 | 6.08 | 2.03 |
| 2012 | 18_32 | 6 | 18_07 | 6 | IA | P | 221 | 607.16 | 2.35 | 2580 | 132.36 | 4.01 | 155 | 400.33 | 3.09 | 0.22 | A | 6.00 | 6.15 | 2.05 |
| 2012 | 18_32 | 6 | 18_07 | 6 | IA | P | 327 | 594.66 | 2.74 | 1902 | 127.15 | 4.41 | 83 | 389.10 | 3.44 | 0.21 | A | 6.00 | 6.36 | 2.12 |
| 2012 | 18_32 | 6 | 18_07 | 6 | IA | P | 237 | 604.57 | 2.12 | 2219 | 130.13 | 4.55 | 81 | 400.42 | 4.29 | 0.22 | A | 6.00 | 6.46 | 2.15 |
| 2012 | 18_32 | 6 | 18_07 | 6 | IA | P | 146 | 601.46 | 2.17 | 2240 | 128.30 | 4.55 | 71 | 389.14 | 3.79 | 0.21 | A | 6.00 | 6.20 | 2.07 |
| 2012 | 18_32 | 6 | 18_07 | 6 | IA | P | 332 | 597.67 | 2.28 | 1996 | 127.56 | 4.19 | 103 | 379.51 | 2.70 | 0.21 | A | 6.00 | 5.85 | 1.95 |
| 2012 | 18_32 | 6 | 18_07 | 6 | IA | P | 202 | 597.71 | 2.59 | 2159 | 129.18 | 3.96 | 83 | 393.26 | 3.48 | 0.22 | A | 6.00 | 6.27 | 2.09 |
| 2012 | 18_32 | 6 | 18_07 | 6 | IA | P | 315 | 602.69 | 2.62 | 2173 | 130.37 | 4.49 | 120 | 395.25 | 3.93 | 0.22 | A | 6.00 | 6.19 | 2.06 |
| 2012 | 18_32 | 6 | 18_11 | 5 | IE | P | 336 | 600.41 | 2.61 | 1711 | 129.67 | 4.83 | 171 | 192.36 | 3.42 | 0.22 | S | 6.05 | 3.12 | 1.04 |
| 2012 | 18_32 | 6 | 18_11 | 5 | IE | P | 333 | 603.06 | 3.07 | 1740 | 130.03 | 5.59 | 89 | 374.23 | 3.32 | 0.22 | A | 6.00 | 5.27 | 1.76 |
| 2012 | 18_32 | 6 | 18_11 | 5 | IE | P | 355 | 609.69 | 2.93 | 1848 | 130.87 | 4.60 | 97 | 389.10 | 4.23 | 0.21 | A | 6.00 | 5.84 | 1.95 |
| 2012 | 18_32 | 6 | 18_15 | 4 | IE | P | 330 | 613.74 | 2.78 | 1388 | 133.21 | 4.47 | 87 | 388.03 | 3.23 | 0.22 | A | 6.00 | 5.48 | 1.83 |
| 2012 | 18_32 | 6 | 18_15 | 4 | IE | P | 411 | 592.93 | 2.44 | 1516 | 127.80 | 5.22 | 97 | 367.23 | 4.18 | 0.22 | A | 6.00 | 5.24 | 1.75 |
| 2012 | 18_32 | 6 | 18_20 | 8 | IE | P | 184 | 606.53 | 2.11 | 1831 | 130.56 | 5.09 | 86 | 387.58 | 3.06 | 0.22 | A | 6.00 | 5.81 | 1.94 |
| 2012 | 18_32 | 6 | 18_20 | 8 | IE | P | 157 | 595.09 | 2.73 | 1592 | 128.57 | 5.12 | 167 | 373.20 | 3.56 | 0.22 | A | 6.00 | 5.42 | 1.81 |
| 2012 | 18_32 | 6 | 18_23 | 7 | IE | P | 294 | 598.37 | 2.58 | 1814 | 128.60 | 4.60 | 97 | 396.20 | 3.51 | 0.21 | A | 6.00 | 6.49 | 2.16 |
| 2012 | 18_32 | 6 | 18_23 | 7 | IE | P | 212 | 594.78 | 2.60 | 1983 | 126.78 | 5.11 | 130 | 391.70 | 3.98 | 0.21 | A | 6.00 | 6.54 | 2.18 |
| 2012 | 18_32 | 6 | 18_23 | 7 | IE | P | 254 | 590.38 | 2.17 | 1765 | 126.00 | 4.43 | 82 | 389.18 | 3.97 | 0.21 | A | 6.00 | 6.53 | 2.18 |
| 2012 | 18_32 | 6 | 18_23 | 7 | IE | P | 120 | 596.78 | 2.22 | 1670 | 127.69 | 4.70 | 101 | 390.55 | 3.48 | 0.21 | A | 6.00 | 6.35 | 2.12 |
| 2012 | 18_32 | 6 | 18_23 | 7 | IE | P | 268 | 603.35 | 3.06 | 1847 | 129.09 | 4.90 | 104 | 398.20 | 4.19 | 0.21 | A | 6.00 | 6.51 | 2.17 |
| 2012 | 18_32 | 6 | 18_23 | 7 | IE | P | 220 | 587.98 | 4.12 | 1775 | 122.84 | 6.86 | 100 | 385.55 | 4.10 | 0.21 | A | 6.00 | 6.83 | 2.28 |
| 2012 | 18_32 | 6 | 18_23 | 7 | IE | P | 231 | 599.46 | 4.11 | 1775 | 127.85 | 5.66 | 87 | 411.50 | 3.67 | 0.21 | A | 6.00 | 7.31 | 2.44 |
| 2012 | 18_32 | 6 | 18_23 | 7 | IE | P | 190 | 589.36 | 3.45 | 1858 | 125.83 | 5.85 | 101 | 401.28 | 4.29 | 0.21 | A | 6.00 | 7.13 | 2.38 |
| 2012 | 18_32 | 6 | 18_23 | 7 | IE | P | 227 | 589.11 | 3.83 | 1707 | 125.31 | 6.25 | 101 | 388.12 | 4.20 | 0.21 | A | 6.00 | 6.58 | 2.19 |
| 2012 | 18_32 | 6 | 18_23 | 7 | IE | P | 128 | 586.74 | 2.80 | 1727 | 122.47 | 4.95 | 56 | 378.18 | 3.07 | 0.21 | A | 6.00 | 6.53 | 2.18 |
| 2012 | 18_32 | 6 | 18_25 | 5 | IE | P | 319 | 615.03 | 2.62 | 1589 | 134.09 | 4.55 | 126 | 361.95 | 4.21 | 0.22 | A | 6.00 | 4.20 | 1.40 |
| 2012 | 18_32 | 6 | 18_32 | 6 | S | P | 164 | 590.30 | 2.55 | 1855 | 128.26 | 4.84 | 108 | 386.49 | 4.54 | 0.22 | A | 6.00 | 6.08 | 2.03 |
| 2012 | 18_32 | 6 | 18_37 | 7 | IE | P | 145 | 601.84 | 2.11 | 2007 | 132.25 | 4.71 | 141 | 397.66 | 3.55 | 0.22 | A | 6.00 | 6.04 | 2.01 |
| 2012 | 18_32 | 6 | 18_37 | 7 | IE | P | 265 | 592.39 | 2.66 | 1828 | 127.09 | 4.20 | 144 | 379.84 | 3.20 | 0.21 | A | 6.00 | 5.93 | 1.98 |
| 2012 | 18_32 | 6 | 18_37 | 7 | IE | P | 205 | 590.78 | 2.60 | 2068 | 129.52 | 4.72 | 122 | 392.48 | 2.75 | 0.22 | A | 6.00 | 6.18 | 2.06 |
| 2012 | 18_32 | 6 | 18_37 | 7 | IE | P | 299 | 602.29 | 2.44 | 1858 | 128.82 | 4.81 | 115 | 340.33 | 3.90 | 0.21 | A | 6.00 | 3.85 | 1.28 |
| 2012 | 18_32 | 6 | 18_37 | 7 | IE | P | 282 | 588.60 | 3.36 | 1656 | 128.79 | 5.17 | 80 | 393.99 | 3.62 | 0.22 | A | 6.00 | 6.35 | 2.12 |
| 2012 | 18_32 | 6 | 18_37 | 7 | IE | P | 268 | 597.12 | 2.55 | 2132 | 129.31 | 5.00 | 162 | 383.44 | 3.19 | 0.22 | A | 6.00 | 5.79 | 1.93 |
| 2012 | 18_32 | 6 | 18_37 | 7 | IE | P | 177 | 601.95 | 2.42 | 2274 | 132.50 | 4.39 | 115 | 401.03 | 3.21 | 0.22 | A | 6.00 | 6.16 | 2.05 |
| 2012 | 18_32 | 6 | 18_37 | 7 | IE | P | 170 | 600.76 | 2.28 | 1933 | 131.11 | 4.63 | 145 | 397.19 | 3.11 | 0.22 | A | 6.00 | 6.18 | 2.06 |
| 2012 | 18_32 | 6 | 18_37 | 7 | IE | P | 213 | 597.03 | 2.17 | 2085 | 129.42 | 4.61 | 114 | 399.59 | 3.15 | 0.22 | A | 6.00 | 6.53 | 2.18 |
| 2012 | 18_32 | 6 | 18_37 | 7 | IE | P | 109 | 593.63 | 2.40 | 1076 | 130.87 | 6.27 | 95 | 412.12 | 4.12 | 0.22 | A | 6.00 | 6.89 | 2.30 |
| 2012 | 18_32 | 6 | 18_41 | 8 | IE | P | 234 | 601.07 | 2.54 | 1829 | 132.61 | 4.98 | 157 | 392.56 | 3.50 | 0.22 | A | 6.00 | 5.76 | 1.92 |
| 2012 | 18_32 | 6 | 18_41 | 8 | IE | P | 348 | 605.77 | 2.83 | 1754 | 130.17 | 5.37 | 101 | 393.44 | 3.30 | 0.21 | A | 6.00 | 6.14 | 2.05 |
| 2012 | 18_32 | 6 | 18_41 | 8 | IE | P | 336 | 605.89 | 2.27 | 1695 | 127.51 | 4.94 | 128 | 377.55 | 3.61 | 0.21 | A | 6.00 | 5.77 | 1.92 |
| 2012 | 18_32 | 6 | 18_41 | 8 | IE | P | 235 | 603.77 | 2.47 | 2012 | 128.07 | 4.84 | 124 | 387.10 | 2.89 | 0.21 | A | 6.00 | 6.14 | 2.05 |
| 2012 | 18_32 | 6 | 18_44 | 8 | IE | P | 171 | 590.06 | 2.28 | 2149 | 125.42 | 5.27 | 125 | 411.62 | 2.78 | 0.21 | A | 6.00 | 7.69 | 2.56 |
| 2012 | 18_32 | 6 | 18_44 | 8 | IE | P | 119 | 596.51 | 1.91 | 1314 | 126.99 | 4.58 | 82 | 398.16 | 2.96 | 0.21 | A | 6.00 | 6.81 | 2.27 |
| 2012 | 18_32 | 6 | 18_45 | 5 | IE | P | 283 | 605.08 | 2.51 | 1930 | 129.91 | 4.82 | 85 | 389.54 | 4.01 | 0.21 | A | 6.00 | 5.99 | 2.00 |
| 2012 | 18_32 | 6 | 18_48 | 4 | IE | P | 412 | 602.28 | 2.86 | 1464 | 129.63 | 5.26 | 116 | 384.88 | 3.45 | 0.22 | A | 6.00 | 5.81 | 1.94 |
| 2012 | 18_32 | 6 | 18_48 | 4 | IE | P | 296 | 608.93 | 2.95 | 1599 | 129.89 | 5.58 | 92 | 387.20 | 3.04 | 0.21 | A | 6.00 | 5.89 | 1.96 |
| 2012 | 18_32 | 6 | 18_48 | 4 | IE | P | 471 | 613.38 | 3.45 | 1644 | 131.93 | 5.66 | 87 | 393.30 | 3.71 | 0.22 | A | 6.00 | 5.89 | 1.96 |
| 2012 | 18_36 | 5 | 18_05 | 6 | IE | P | 438 | 592.78 | 2.06 | 1241 | 63.05 | 6.96 | 215 | 247.28 | 4.86 | 0.11 | HP | 2.95 |  |  |
| 2012 | 18_36 | 5 | 18_07 | 6 | IE | P | 314 | 607.10 | 3.80 | 1845 | 110.67 | 5.66 | 98 | 285.48 | 4.35 | 0.18 | A | 5.00 | 2.90 | 1.16 |
| 2012 | 18_36 | 5 | 18_14 | 4 | IE | P | 479 | 602.48 | 3.29 | 1466 | 103.11 | 5.53 | 148 | 162.64 | 4.55 | 0.17 | S | 4.75 | 2.01 | 0.80 |
| 2012 | 18_36 | 5 | 18_44 | 8 | IE | P | 440 | 591.77 | 3.48 | 1768 | 100.92 | 6.05 | 87 | 343.23 | 5.73 | 0.17 | A | 5.00 | 7.01 | 2.80 |
| 2012 | 18_36 | 5 | 18_45 | 5 | IA | P | 408 | 606.84 | 2.34 | 1869 | 107.79 | 4.99 | 76 | 430.93 | 3.14 | 0.18 | A | 5.00 | 9.99 | 4.00 |
| 2012 | 18_37 | 7 | 18_01 | 6 | IE | P | 299 | 618.92 | 2.93 | 2043 | 153.09 | 4.93 | 101 | 421.89 | 3.48 | 0.25 | A | 7.00 | 5.29 | 1.51 |
| 2012 | 18_37 | 7 | 18_05 | 6 | IE | P | 481 | 608.13 | 2.76 | 2096 | 150.60 | 4.75 | 95 | 427.28 | 3.95 | 0.25 | A | 7.00 | 5.86 | 1.67 |
| 2012 | 18_37 | 7 | 18_05 | 6 | IE | P | 635 | 596.65 | 3.18 | 1579 | 147.80 | 4.58 | 71 | 420.66 | 3.83 | 0.25 | A | 7.00 | 5.92 | 1.69 |
| 2012 | 18_37 | 7 | 18_05 | 6 | IE | P | 343 | 607.93 | 3.81 | 1893 | 137.72 | 5.65 | 118 | 217.21 | 4.30 | 0.23 | S | 6.40 | 2.71 | 0.77 |
| 2012 | 18_37 | 7 | 18_05 | 6 | IE | P | 487 | 609.47 | 3.65 | 1678 | 153.27 | 4.98 | 117 | 433.74 | 5.35 | 0.25 | A | 7.00 | 5.81 | 1.66 |
| 2012 | 18_37 | 7 | 18_05 | 6 | IE | P | 588 | 603.37 | 3.63 | 1664 | 149.28 | 5.13 | 83 | 432.35 | 4.75 | 0.25 | A | 7.00 | 6.27 | 1.79 |
| 2012 | 18_37 | 7 | 18_05 | 6 | IE | P | 401 | 611.83 | 4.32 | 2023 | 153.38 | 5.29 | 97 | 432.37 | 4.58 | 0.25 | A | 7.00 | 5.73 | 1.64 |
| 2012 | 18_37 | 7 | 18_32 | 6 | IE | P | 557 | 595.23 | 3.47 | 1375 | 75.78 | 5.97 | 99 | 217.89 | 4.89 | 0.13 | HP | 3.60 |  |  |
| 2012 | 18_37 | 7 | 18_32 | 6 | IE | P | 386 | 601.91 | 3.22 | 1679 | 148.29 | 4.54 | 110 | 272.31 | 3.51 | 0.25 | A1 | 7.00 |  |  |
| 2012 | 18_37 | 7 | 18_32 | 6 | IE | P | 370 | 608.04 | 2.84 | 2056 | 148.81 | 4.89 | 59 | 416.80 | 3.91 | 0.24 | A | 7.00 | 5.61 | 1.60 |
| 2012 | 18_37 | 7 | 18_32 | 6 | IE | P | 488 | 609.08 | 3.58 | 1686 | 152.02 | 4.90 | 103 | 434.51 | 4.09 | 0.25 | A | 7.00 | 6.01 | 1.72 |
| 2012 | 18_37 | 7 | 18_38 | 7 | IA | P | 365 | 609.33 | 3.27 | 1859 | 151.39 | 5.65 | 91 | 384.78 | 3.81 | 0.25 | A | 7.00 | 3.79 | 1.08 |
| 2012 | 18_37 | 7 | 18_45 | 5 | IE | P | 257 | 592.92 | 2.64 | 1617 | 145.25 | 4.93 | 66 | 336.05 | 4.09 | 0.24 | A | 7.00 | 2.20 | 0.63 |
| 2012 | 18_37 | 7 | 18_48 | 4 | IE | P | 422 | 603.19 | 2.77 | 1660 | 148.16 | 4.51 | 127 | 424.16 | 3.21 | 0.25 | A | 7.00 | 6.04 | 1.73 |
| 2012 | 18_37 | 7 | 18_48 | 4 | IE | P | 449 | 592.06 | 3.28 | 1370 | 212.07 | 4.38 | 106 | 361.16 | 4.19 | 0.36 | AF | 10.12 |  |  |
| 2012 | 18_37 | 7 | 18_48 | 4 | IE | P | 718 | 601.72 | 3.03 | 1277 | 152.78 | 5.10 | 81 | 426.29 | 4.56 | 0.25 | A | 7.00 | 5.53 | 1.58 |
| 2012 | 18_41 | 8 | 18_01 | 6 | IE | L | 170 | 709.99 | 3.03 | 1130 | 131.39 | 5.62 | 83 | 367.58 | 4.94 | 0.19 | A | 8.00 | 6.38 | 1.60 |
| 2012 | 18_41 | 8 | 18_01 | 6 | IE | L | 182 | 718.05 | 2.78 | 1258 | 130.80 | 6.04 | 111 | 429.28 | 4.81 | 0.18 | A | 8.00 | 10.26 | 2.56 |
| 2012 | 18_41 | 8 | 18_05 | 6 | IE | L | 204 | 706.46 | 3.18 | 1415 | 130.31 | 5.49 | 90 | 365.36 | 4.36 | 0.18 | A | 8.00 | 6.43 | 1.61 |
| 2012 | 18_41 | 8 | 18_07 | 6 | IE | L | 146 | 722.00 | 3.38 | 2045 | 130.11 | 5.71 | 125 | 353.65 | 4.63 | 0.18 | A | 8.00 | 5.74 | 1.44 |
| 2012 | 18_41 | 8 | 18_14 | 4 | IE | L | 283 | 710.07 | 3.33 | 1856 | 128.36 | 4.97 | 140 | 358.35 | 4.37 | 0.18 | A | 8.00 | 6.33 | 1.58 |
| 2012 | 18_41 | 8 | 18_14 | 4 | IE | L | 222 | 702.75 | 3.21 | 1988 | 123.14 | 5.66 | 112 | 357.75 | 4.14 | 0.18 | A | 8.00 | 7.24 | 1.81 |
| 2012 | 18_41 | 8 | 18_15 | 4 | IE | L | 168 | 700.70 | 3.33 | 1706 | 122.99 | 5.19 | 78 | 490.98 | 3.36 | 0.18 | A | 8.00 | 15.94 | 3.98 |
| 2012 | 18_41 | 8 | 18_25 | 5 | IE | L | 169 | 711.48 | 2.83 | 1614 | 126.76 | 5.09 | 131 | 345.76 | 4.12 | 0.18 | A | 8.00 | 5.82 | 1.46 |
| 2012 | 18_41 | 8 | 18_32 | 6 | IE | L | 116 | 705.73 | 2.91 | 1400 | 124.84 | 5.07 | 66 | 335.19 | 4.26 | 0.18 | A | 8.00 | 5.48 | 1.37 |
| 2012 | 18_41 | 8 | 18_41 | 8 | S | L | 216 | 713.82 | 2.72 | 1714 | 125.61 | 4.70 | 123 | 373.62 | 3.56 | 0.18 | A | 8.00 | 7.80 | 1.95 |
| 2012 | 18_41 | 8 | 18_44 | 8 | IA | L | 141 | 723.79 | 3.08 | 2072 | 128.80 | 4.56 | 70 | 383.91 | 3.30 | 0.18 | A | 8.00 | 7.85 | 1.96 |
| 2012 | 18_41 | 8 | 18_45 | 5 | IE | L | 205 | 702.08 | 3.29 | 2067 | 125.74 | 6.03 | 125 | 328.04 | 4.62 | 0.18 | A | 8.00 | 4.87 | 1.22 |
| 2012 | 18_41 | 8 | 18_47 | 4 | IE | L | 134 | 718.06 | 2.81 | 1565 | 127.19 | 4.94 | 74 | 316.82 | 4.22 | 0.18 | A | 8.00 | 3.93 | 0.98 |
| 2012 | 18_41 | 8 | 18_47 | 4 | IE | L | 265 | 704.05 | 3.20 | 1555 | 127.74 | 6.05 | 51 | 311.17 | 4.19 | 0.18 | A | 8.00 | 3.49 | 0.87 |
| 2012 | 18_41 | 8 | 18_47 | 4 | IE | L | 204 | 699.74 | 3.07 | 1609 | 124.28 | 5.42 | 131 | 364.19 | 3.32 | 0.18 | A | 8.00 | 7.44 | 1.86 |
| 2012 | 18_41 | 8 | 18_47 | 4 | IE | L | 135 | 701.73 | 2.21 | 1607 | 124.77 | 4.73 | 49 | 308.64 | 3.58 | 0.18 | A | 8.00 | 3.79 | 0.95 |
| 2012 | 18_41 | 8 | 18_48 | 4 | IE | L | 170 | 704.85 | 4.03 | 1950 | 124.90 | 7.37 | 135 | 355.88 | 5.04 | 0.18 | A | 8.00 | 6.79 | 1.70 |
| 2012 | 18_43 | 4 | 18_01 | 6 | IE | P | 248 | 602.84 | 2.78 | 1892 | 92.22 | 5.78 | 122 | 307.20 | 4.16 | 0.15 | A | 4.00 | 5.32 | 2.66 |
| 2012 | 18_43 | 4 | 18_01 | 6 | IE | P | 324 | 606.97 | 2.07 | 2379 | 92.22 | 6.25 | 76 | 300.37 | 4.03 | 0.15 | A | 4.00 | 5.03 | 2.51 |
| 2012 | 18_43 | 4 | 18_02 | 6 | IE | P | 242 | 593.41 | 4.21 | 1775 | 88.87 | 6.29 | 131 | 297.23 | 5.35 | 0.15 | A | 4.00 | 5.38 | 2.69 |
| 2012 | 18_43 | 4 | 18_05 | 6 | IE | P | 149 | 595.80 | 2.75 | 1767 | 91.61 | 6.12 | 107 | 280.75 | 4.66 | 0.15 | A | 4.00 | 4.26 | 2.13 |
| 2012 | 18_43 | 4 | 18_07 | 6 | IE | P | 282 | 606.94 | 2.60 | 1917 | 90.53 | 5.40 | 125 | 247.40 | 5.23 | 0.15 | A | 4.00 | 2.93 | 1.47 |
| 2012 | 18_43 | 4 | 18_07 | 6 | IE | P | 280 | 598.85 | 2.65 | 2149 | 90.06 | 5.61 | 103 | 319.35 | 3.89 | 0.15 | A | 4.00 | 6.18 | 3.09 |
| 2012 | 18_43 | 4 | 18_07 | 6 | IE | P | 253 | 598.97 | 2.33 | 2006 | 91.15 | 5.63 | 107 | 305.88 | 4.17 | 0.15 | A | 4.00 | 5.42 | 2.71 |
| 2012 | 18_43 | 4 | 18_07 | 6 | IE | P | 282 | 584.78 | 2.43 | 1676 | 145.31 | 4.44 | 127 | 235.59 | 3.78 | 0.25 | AF | 6.58 |  |  |
| 2012 | 18_43 | 4 | 18_07 | 6 | IE | P | 155 | 596.31 | 2.53 | 1783 | 89.01 | 5.56 | 117 | 311.25 | 3.59 | 0.15 | A | 4.00 | 5.99 | 2.99 |
| 2012 | 18_43 | 4 | 18_07 | 6 | IE | P | 350 | 597.02 | 2.16 | 1783 | 90.68 | 5.40 | 123 | 309.43 | 3.01 | 0.15 | A | 4.00 | 5.65 | 2.82 |
| 2012 | 18_43 | 4 | 18_07 | 6 | IE | P | 255 | 602.77 | 2.17 | 1946 | 91.91 | 5.26 | 94 | 304.31 | 3.71 | 0.15 | A | 4.00 | 5.24 | 2.62 |
| 2012 | 18_43 | 4 | 18_07 | 6 | IE | P | 252 | 598.20 | 2.08 | 2037 | 90.36 | 5.53 | 92 | 304.43 | 3.48 | 0.15 | A | 4.00 | 5.48 | 2.74 |
| 2012 | 18_43 | 4 | 18_07 | 6 | IE | P | 298 | 598.36 | 2.50 | 2186 | 90.92 | 5.63 | 130 | 303.50 | 4.59 | 0.15 | A | 4.00 | 5.35 | 2.68 |
| 2012 | 18_43 | 4 | 18_07 | 6 | IE | P | 215 | 592.75 | 2.77 | 1870 | 89.55 | 5.52 | 89 | 313.48 | 3.29 | 0.15 | A | 4.00 | 6.00 | 3.00 |
| 2012 | 18_43 | 4 | 18_11 | 5 | IE | P | 411 | 607.86 | 3.51 | 1569 | 93.48 | 5.94 | 80 | 292.29 | 4.25 | 0.15 | A | 4.00 | 4.51 | 2.25 |
| 2012 | 18_43 | 4 | 18_11 | 5 | IE | P | 394 | 607.68 | 3.88 | 1768 | 92.68 | 6.63 | 119 | 230.79 | 4.49 | 0.15 | A | 4.00 | 1.96 | 0.98 |
| 2012 | 18_43 | 4 | 18_11 | 5 | IE | P | 448 | 611.02 | 3.39 | 1580 | 93.61 | 6.27 | 96 | 301.20 | 4.76 | 0.15 | A | 4.00 | 4.87 | 2.44 |
| 2012 | 18_43 | 4 | 18_23 | 7 | IE | P | 296 | 597.56 | 2.03 | 1945 | 90.99 | 4.85 | 136 | 254.68 | 2.76 | 0.15 | A | 4.00 | 3.20 | 1.60 |
| 2012 | 18_43 | 4 | 18_23 | 7 | IE | P | 302 | 603.51 | 1.56 | 1857 | 91.21 | 5.02 | 108 | 312.02 | 3.27 | 0.15 | A | 4.00 | 5.68 | 2.84 |
| 2012 | 18_43 | 4 | 18_23 | 7 | IE | P | 462 | 594.15 | 2.19 | 1515 | 89.67 | 4.86 | 139 | 161.00 | 3.94 | 0.15 | A1 | 4.00 |  |  |
| 2012 | 18_43 | 4 | 18_23 | 7 | IE | P | 457 | 595.71 | 2.19 | 2032 | 90.88 | 5.30 | 74 | 315.31 | 3.57 | 0.15 | A | 4.00 | 5.88 | 2.94 |
| 2012 | 18_43 | 4 | 18_23 | 7 | IE | P | 502 | 601.52 | 2.33 | 1788 | 91.53 | 5.14 | 118 | 256.93 | 3.91 | 0.15 | A | 4.00 | 3.23 | 1.61 |
| 2012 | 18_43 | 4 | 18_23 | 7 | IE | P | 302 | 607.25 | 2.16 | 1741 | 91.15 | 5.04 | 112 | 260.13 | 3.65 | 0.15 | A | 4.00 | 3.42 | 1.71 |
| 2012 | 18_43 | 4 | 18_23 | 7 | IE | P | 401 | 594.22 | 2.10 | 1867 | 89.63 | 5.19 | 125 | 255.38 | 4.15 | 0.15 | A | 4.00 | 3.40 | 1.70 |
| 2012 | 18_43 | 4 | 18_23 | 7 | IE | P | 379 | 603.30 | 1.99 | 2050 | 91.54 | 5.34 | 86 | 318.44 | 2.57 | 0.15 | A | 4.00 | 5.91 | 2.96 |
| 2012 | 18_43 | 4 | 18_23 | 7 | IE | P | 363 | 595.69 | 1.80 | 1878 | 89.20 | 5.10 | 97 | 250.13 | 3.63 | 0.15 | A | 4.00 | 3.22 | 1.61 |
| 2012 | 18_43 | 4 | 18_23 | 7 | IE | P | 359 | 596.35 | 2.15 | 1996 | 89.84 | 5.68 | 128 | 251.85 | 4.16 | 0.15 | A | 4.00 | 3.21 | 1.61 |
| 2012 | 18_43 | 4 | 18_27 | 8 | IE | P | 249 | 600.85 | 2.20 | 1796 | 92.05 | 5.43 | 98 | 325.07 | 4.28 | 0.15 | A | 4.00 | 6.13 | 3.06 |
| 2012 | 18_43 | 4 | 18_27 | 8 | IE | P | 178 | 597.63 | 1.87 | 1639 | 90.30 | 5.40 | 92 | 272.14 | 3.30 | 0.15 | A | 4.00 | 4.05 | 2.03 |
| 2012 | 18_43 | 4 | 18_27 | 8 | IE | P | 285 | 588.04 | 2.30 | 1756 | 90.39 | 5.70 | 110 | 310.88 | 3.58 | 0.15 | A | 4.00 | 5.76 | 2.88 |
| 2012 | 18_43 | 4 | 18_27 | 8 | IE | P | 196 | 599.01 | 1.64 | 2116 | 92.02 | 5.37 | 113 | 289.38 | 4.05 | 0.15 | A | 4.00 | 4.58 | 2.29 |
| 2012 | 18_43 | 4 | 18_27 | 8 | IE | P | 322 | 598.12 | 1.99 | 1750 | 91.65 | 5.30 | 88 | 311.69 | 3.51 | 0.15 | A | 4.00 | 5.60 | 2.80 |
| 2012 | 18_43 | 4 | 18_27 | 8 | IE | P | 208 | 595.71 | 2.10 | 1476 | 174.88 | 3.74 | 106 | 270.34 | 3.87 | 0.29 | AF | 7.78 |  |  |
| 2012 | 18_43 | 4 | 18_27 | 8 | IE | P | 261 | 596.42 | 2.19 | 2155 | 90.05 | 5.54 | 148 | 262.82 | 3.31 | 0.15 | A | 4.00 | 3.67 | 1.84 |
| 2012 | 18_43 | 4 | 18_27 | 8 | IE | P | 397 | 596.09 | 1.83 | 1013 | 98.38 | 6.15 | 204 | 359.99 | 4.23 | 0.17 | A | 4.00 | 6.64 | 3.32 |
| 2012 | 18_43 | 4 | 18_27 | 8 | IE | P | 252 | 593.76 | 1.95 | 1873 | 90.30 | 5.65 | 151 | 265.59 | 4.24 | 0.15 | A | 4.00 | 3.76 | 1.88 |
| 2012 | 18_43 | 4 | 18_32 | 6 | IE | P | 301 | 593.35 | 2.08 | 2099 | 90.50 | 5.15 | 78 | 306.49 | 3.04 | 0.15 | A | 4.00 | 5.55 | 2.77 |
| 2012 | 18_43 | 4 | 18_32 | 6 | IE | P | 583 | 586.95 | 2.47 | 1355 | 174.59 | 3.48 | 77 | 482.12 | 3.14 | 0.30 | AS | 7.88 |  |  |
| 2012 | 18_43 | 4 | 18_32 | 6 | IE | P | 594 | 598.98 | 2.19 | 1636 | 92.29 | 5.33 | 93 | 318.43 | 3.22 | 0.15 | A | 4.00 | 5.80 | 2.90 |
| 2012 | 18_43 | 4 | 18_32 | 6 | IE | P | 457 | 595.88 | 2.73 | 1675 | 91.21 | 5.22 | 94 | 314.34 | 3.13 | 0.15 | A | 4.00 | 5.79 | 2.89 |
| 2012 | 18_43 | 4 | 18_32 | 6 | IE | P | 441 | 602.04 | 2.00 | 1857 | 92.46 | 5.04 | 102 | 312.40 | 3.72 | 0.15 | A | 4.00 | 5.52 | 2.76 |
| 2012 | 18_43 | 4 | 18_32 | 6 | IE | P | 440 | 597.62 | 2.52 | 1952 | 157.42 | 3.87 | 117 | 256.84 | 3.11 | 0.26 | AF | 6.98 |  |  |
| 2012 | 18_43 | 4 | 18_32 | 6 | IE | P | 343 | 598.61 | 2.02 | 1751 | 154.96 | 3.94 | 145 | 254.10 | 4.52 | 0.26 | AF | 6.86 |  |  |
| 2012 | 18_43 | 4 | 18_32 | 6 | IE | P | 387 | 596.09 | 2.30 | 2099 | 91.86 | 5.87 | 110 | 320.16 | 3.72 | 0.15 | A | 4.00 | 5.94 | 2.97 |
| 2012 | 18_43 | 4 | 18_32 | 6 | IE | P | 366 | 614.90 | 2.31 | 2099 | 93.76 | 5.23 | 82 | 332.53 | 4.22 | 0.15 | A | 4.00 | 6.19 | 3.09 |
| 2012 | 18_43 | 4 | 18_32 | 6 | IE | P | 332 | 606.27 | 2.46 | 2011 | 93.33 | 5.12 | 83 | 266.77 | 3.37 | 0.15 | A | 4.00 | 3.43 | 1.72 |
| 2012 | 18_43 | 4 | 18_36 | 5 | IE | P | 367 | 611.94 | 3.36 | 1802 | 94.08 | 6.08 | 94 | 291.19 | 3.85 | 0.15 | A | 4.00 | 4.38 | 2.19 |
| 2012 | 18_43 | 4 | 18_36 | 5 | IE | P | 355 | 599.52 | 3.53 | 1709 | 91.72 | 6.13 | 142 | 293.19 | 4.76 | 0.15 | A | 4.00 | 4.79 | 2.39 |
| 2012 | 18_43 | 4 | 18_36 | 5 | IE | P | 364 | 599.18 | 3.35 | 1968 | 91.12 | 6.45 | 130 | 281.62 | 4.64 | 0.15 | A | 4.00 | 4.36 | 2.18 |
| 2012 | 18_43 | 4 | 18_37 | 7 | IE | P | 434 | 580.64 | 4.51 | 1818 | 84.53 | 5.66 | 121 | 236.43 | 3.89 | 0.15 | A | 4.00 | 3.19 | 1.59 |
| 2012 | 18_43 | 4 | 18_37 | 7 | IE | P | 351 | 589.33 | 2.90 | 1774 | 87.34 | 5.23 | 123 | 309.12 | 3.83 | 0.15 | A | 4.00 | 6.16 | 3.08 |
| 2012 | 18_43 | 4 | 18_37 | 7 | IE | P | 343 | 590.39 | 2.17 | 2003 | 86.99 | 5.54 | 130 | 256.43 | 4.20 | 0.15 | A | 4.00 | 3.79 | 1.90 |
| 2012 | 18_43 | 4 | 18_37 | 7 | IE | P | 469 | 593.52 | 1.99 | 1342 | 88.69 | 5.68 | 199 | 251.63 | 4.03 | 0.15 | A | 4.00 | 3.35 | 1.67 |
| 2012 | 18_43 | 4 | 18_37 | 7 | IE | P | 539 | 582.59 | 2.23 | 1491 | 161.12 | 3.82 | 163 | 249.45 | 4.25 | 0.28 | AF | 7.33 |  |  |
| 2012 | 18_43 | 4 | 18_37 | 7 | IE | P | 419 | 592.19 | 2.06 | 1948 | 88.84 | 5.26 | 127 | 244.57 | 3.59 | 0.15 | A | 4.00 | 3.01 | 1.51 |
| 2012 | 18_43 | 4 | 18_37 | 7 | IE | P | 488 | 588.78 | 2.09 | 1633 | 87.86 | 5.02 | 136 | 244.82 | 2.94 | 0.15 | A | 4.00 | 3.15 | 1.57 |
| 2012 | 18_43 | 4 | 18_37 | 7 | IE | P | 445 | 587.58 | 2.08 | 1851 | 88.26 | 4.91 | 123 | 243.64 | 4.37 | 0.15 | A | 4.00 | 3.04 | 1.52 |
| 2012 | 18_43 | 4 | 18_37 | 7 | IE | P | 391 | 597.93 | 2.16 | 1972 | 89.37 | 5.02 | 96 | 315.84 | 3.15 | 0.15 | A | 4.00 | 6.14 | 3.07 |
| 2012 | 18_43 | 4 | 18_37 | 7 | IE | P | 534 | 596.45 | 2.40 | 1940 | 89.55 | 5.08 | 73 | 266.76 | 2.88 | 0.15 | A | 4.00 | 3.92 | 1.96 |
| 2012 | 18_43 | 4 | 18_38 | 7 | IE | P | 205 | 598.51 | 2.70 | 1859 | 90.71 | 6.88 | 162 | 321.53 | 4.22 | 0.15 | A | 4.00 | 6.18 | 3.09 |
| 2012 | 18_43 | 4 | 18_38 | 7 | IE | P | 192 | 608.71 | 2.39 | 2184 | 91.45 | 5.76 | 130 | 259.25 | 5.50 | 0.15 | A | 4.00 | 3.34 | 1.67 |
| 2012 | 18_43 | 4 | 18_38 | 7 | IE | P | 223 | 606.18 | 2.37 | 2116 | 93.66 | 5.77 | 121 | 287.64 | 4.66 | 0.15 | A | 4.00 | 4.28 | 2.14 |
| 2012 | 18_43 | 4 | 18_41 | 8 | IE | P | 391 | 596.80 | 2.07 | 1650 | 93.01 | 5.66 | 159 | 348.55 | 4.50 | 0.16 | A | 4.00 | 6.99 | 3.49 |
| 2012 | 18_43 | 4 | 18_41 | 8 | IE | P | 348 | 596.61 | 2.53 | 1587 | 92.73 | 5.97 | 132 | 347.36 | 3.90 | 0.16 | A | 4.00 | 6.98 | 3.49 |
| 2012 | 18_43 | 4 | 18_41 | 8 | IE | P | 324 | 599.02 | 2.00 | 1955 | 92.97 | 5.53 | 104 | 333.03 | 3.62 | 0.16 | A | 4.00 | 6.33 | 3.16 |
| 2012 | 18_43 | 4 | 18_41 | 8 | IE | P | 357 | 596.80 | 2.35 | 1872 | 93.18 | 5.77 | 127 | 339.50 | 4.07 | 0.16 | A | 4.00 | 6.57 | 3.29 |
| 2012 | 18_43 | 4 | 18_41 | 8 | IE | P | 374 | 603.23 | 2.59 | 1866 | 93.31 | 5.26 | 145 | 358.89 | 3.09 | 0.15 | A | 4.00 | 7.38 | 3.69 |
| 2012 | 18_43 | 4 | 18_41 | 8 | IE | P | 304 | 594.78 | 2.22 | 2006 | 91.35 | 5.80 | 110 | 271.81 | 4.64 | 0.15 | A | 4.00 | 3.90 | 1.95 |
| 2012 | 18_43 | 4 | 18_41 | 8 | IE | P | 360 | 601.18 | 2.42 | 1809 | 92.38 | 5.27 | 151 | 267.48 | 4.04 | 0.15 | A | 4.00 | 3.58 | 1.79 |
| 2012 | 18_43 | 4 | 18_41 | 8 | IE | P | 472 | 598.15 | 2.34 | 1713 | 92.59 | 5.62 | 144 | 345.88 | 3.64 | 0.15 | A | 4.00 | 6.94 | 3.47 |
| 2012 | 18_43 | 4 | 18_41 | 8 | IE | P | 525 | 597.87 | 2.23 | 1927 | 92.09 | 5.21 | 109 | 348.45 | 5.37 | 0.15 | A | 4.00 | 7.14 | 3.57 |
| 2012 | 18_43 | 4 | 18_41 | 8 | IE | P | 472 | 602.99 | 2.33 | 1557 | 92.61 | 6.43 | 129 | 367.98 | 4.20 | 0.15 | A | 4.00 | 7.89 | 3.95 |
| 2012 | 18_43 | 4 | 18_44 | 8 | IE | P | 209 | 596.74 | 1.73 | 2306 | 88.36 | 5.43 | 140 | 249.35 | 3.29 | 0.15 | A | 4.00 | 3.29 | 1.64 |
| 2012 | 18_43 | 4 | 18_44 | 8 | IE | P | 138 | 592.50 | 1.97 | 2005 | 87.99 | 5.82 | 126 | 253.27 | 3.35 | 0.15 | A | 4.00 | 3.51 | 1.76 |
| 2012 | 18_43 | 4 | 18_44 | 8 | IE | P | 209 | 600.46 | 3.06 | 2232 | 88.28 | 5.91 | 87 | 319.93 | 3.19 | 0.15 | A | 4.00 | 6.50 | 3.25 |
| 2012 | 18_43 | 4 | 18_47 | 4 | IA | P | 368 | 590.83 | 3.71 | 1840 | 90.26 | 6.14 | 116 | 265.86 | 4.71 | 0.15 | A | 4.00 | 3.78 | 1.89 |
| 2012 | 18_44 | 8 | 18_01 | 6 | IE | L | 158 | 705.06 | 3.49 | 1740 | 130.27 | 5.36 | 124 | 348.89 | 4.99 | 0.18 | A | 8.00 | 5.43 | 1.36 |
| 2012 | 18_44 | 8 | 18_02 | 6 | IE | L | 159 | 706.48 | 3.45 | 1737 | 129.88 | 5.41 | 90 | 353.92 | 3.89 | 0.18 | A | 8.00 | 5.80 | 1.45 |
| 2012 | 18_44 | 8 | 18_02 | 6 | IE | L | 143 | 689.40 | 3.32 | 1818 | 124.11 | 5.23 | 110 | 338.91 | 3.38 | 0.18 | A | 8.00 | 5.85 | 1.46 |
| 2012 | 18_44 | 8 | 18_02 | 6 | IE | L | 205 | 716.55 | 3.12 | 1663 | 129.42 | 5.85 | 72 | 349.83 | 4.42 | 0.18 | A | 8.00 | 5.62 | 1.41 |
| 2012 | 18_44 | 8 | 18_05 | 6 | IE | L | 163 | 704.16 | 3.07 | 2162 | 124.65 | 4.89 | 117 | 340.57 | 4.08 | 0.18 | A | 8.00 | 5.86 | 1.46 |
| 2012 | 18_44 | 8 | 18_05 | 6 | IE | L | 179 | 708.11 | 2.85 | 1725 | 126.27 | 4.69 | 84 | 342.37 | 3.98 | 0.18 | A | 8.00 | 5.69 | 1.42 |
| 2012 | 18_44 | 8 | 18_07 | 6 | IE | L | 177 | 715.99 | 2.90 | 1771 | 128.44 | 4.58 | 84 | 352.94 | 3.67 | 0.18 | A | 8.00 | 5.98 | 1.50 |
| 2012 | 18_44 | 8 | 18_23 | 7 | IE | L | 193 | 698.29 | 3.09 | 1766 | 123.99 | 5.24 | 89 | 357.48 | 4.12 | 0.18 | A | 8.00 | 7.07 | 1.77 |
| 2012 | 18_44 | 8 | 18_23 | 7 | IE | L | 251 | 722.48 | 3.04 | 2041 | 127.59 | 5.13 | 103 | 367.89 | 3.89 | 0.18 | A | 8.00 | 7.07 | 1.77 |
| 2012 | 18_44 | 8 | 18_29 | 5 | IE | L | 218 | 700.77 | 3.75 | 2419 | 126.09 | 5.62 | 59 | 501.38 | 6.00 | 0.18 | A | 8.00 | 15.81 | 3.95 |
| 2012 | 18_44 | 8 | 18_32 | 6 | IE | L | 185 | 713.93 | 3.10 | 1968 | 127.49 | 4.93 | 123 | 308.60 | 3.96 | 0.18 | A | 8.00 | 3.36 | 0.84 |
| 2012 | 18_44 | 8 | 18_32 | 6 | IE | L | 160 | 708.59 | 3.21 | 2122 | 128.06 | 4.65 | 73 | 348.01 | 3.18 | 0.18 | A | 8.00 | 5.74 | 1.44 |
| 2012 | 18_44 | 8 | 18_32 | 6 | IE | L | 228 | 721.22 | 3.50 | 2062 | 129.10 | 4.73 | 74 | 346.36 | 5.11 | 0.18 | A | 8.00 | 5.46 | 1.37 |
| 2012 | 18_44 | 8 | 18_36 | 5 | IE | L | 164 | 703.66 | 2.97 | 2018 | 127.58 | 4.81 | 66 | 353.36 | 3.96 | 0.18 | A | 8.00 | 6.16 | 1.54 |
| 2012 | 18_44 | 8 | 18_37 | 7 | IE | L | 232 | 708.91 | 3.23 | 2183 | 126.40 | 5.02 | 98 | 362.50 | 3.43 | 0.18 | A | 8.00 | 6.94 | 1.74 |
| 2012 | 18_44 | 8 | 18_37 | 7 | IE | L | 220 | 718.13 | 3.02 | 1926 | 128.18 | 4.75 | 101 | 365.38 | 4.00 | 0.18 | A | 8.00 | 6.80 | 1.70 |
| 2012 | 18_44 | 8 | 18_44 | 8 | S | L | 249 | 707.79 | 3.11 | 2133 | 125.71 | 4.91 | 89 | 363.10 | 3.71 | 0.18 | A | 8.00 | 7.11 | 1.78 |
| 2012 | 18_44 | 8 | 18_44 | 8 | S | L | 260 | 706.22 | 3.22 | 2030 | 124.81 | 4.62 | 69 | 380.10 | 4.11 | 0.18 | A | 8.00 | 8.36 | 2.09 |
| 2012 | 18_44 | 8 | 18_44 | 8 | S | L | 207 | 701.33 | 3.01 | 2302 | 123.56 | 4.98 | 78 | 357.84 | 3.84 | 0.18 | A | 8.00 | 7.17 | 1.79 |
| 2012 | 18_45 | 5 | 18_07 | 6 | IE | P | 361 | 606.55 | 3.42 | 1776 | 109.55 | 6.00 | 115 | 347.52 | 4.41 | 0.18 | A | 5.00 | 5.86 | 2.34 |
| 2012 | 18_45 | 5 | 18_29 | 5 | IA | P | 368 | 598.50 | 2.62 | 2041 | 105.78 | 5.65 | 124 | 302.32 | 3.96 | 0.18 | A | 5.00 | 4.29 | 1.72 |
| 2012 | 18_47 | 4 | 18_01 | 6 | IE | P | 234 | 593.69 | 3.08 | 2496 | 92.34 | 6.09 | 53 | 243.79 | 2.75 | 0.16 | A | 4.00 | 2.56 | 1.28 |
| 2012 | 18_47 | 4 | 18_07 | 6 | IE | P | 274 | 594.32 | 2.80 | 1951 | 153.25 | 4.62 | 165 | 240.51 | 3.44 | 0.26 | AF | 6.83 |  |  |
| 2012 | 18_47 | 4 | 18_07 | 6 | IE | P | 291 | 598.42 | 2.60 | 1646 | 89.04 | 5.54 | 155 | 238.03 | 4.23 | 0.15 | A | 4.00 | 2.69 | 1.35 |
| 2012 | 18_47 | 4 | 18_07 | 6 | IE | P | 295 | 609.69 | 2.08 | 1694 | 90.08 | 5.91 | 140 | 238.97 | 4.13 | 0.15 | A | 4.00 | 2.61 | 1.31 |
| 2012 | 18_47 | 4 | 18_11 | 5 | IE | P | 357 | 603.44 | 3.39 | 1673 | 92.38 | 6.06 | 99 | 295.58 | 4.01 | 0.15 | A | 4.00 | 4.80 | 2.40 |
| 2012 | 18_47 | 4 | 18_11 | 5 | IE | P | 201 | 610.15 | 2.89 | 1156 | 94.91 | 5.77 | 84 | 305.26 | 5.15 | 0.16 | A | 4.00 | 4.87 | 2.43 |
| 2012 | 18_47 | 4 | 18_23 | 7 | IE | P | 228 | 602.37 | 2.41 | 2047 | 89.14 | 5.83 | 142 | 253.58 | 3.98 | 0.15 | A | 4.00 | 3.38 | 1.69 |
| 2012 | 18_47 | 4 | 18_23 | 7 | IE | P | 314 | 601.67 | 2.97 | 1967 | 89.46 | 6.05 | 124 | 301.23 | 4.26 | 0.15 | A | 4.00 | 5.47 | 2.73 |
| 2012 | 18_47 | 4 | 18_23 | 7 | IE | P | 215 | 602.53 | 2.76 | 2057 | 89.32 | 5.59 | 85 | 316.44 | 4.50 | 0.15 | A | 4.00 | 6.17 | 3.09 |
| 2012 | 18_47 | 4 | 18_23 | 7 | IE | P | 228 | 600.67 | 2.72 | 1825 | 89.36 | 6.05 | 88 | 246.76 | 4.27 | 0.15 | A | 4.00 | 3.05 | 1.52 |
| 2012 | 18_47 | 4 | 18_25 | 5 | IE | P | 308 | 613.95 | 3.01 | 1686 | 92.81 | 6.17 | 121 | 299.60 | 3.91 | 0.15 | A | 4.00 | 4.91 | 2.46 |
| 2012 | 18_47 | 4 | 18_32 | 6 | IE | P | 318 | 597.57 | 2.52 | 1878 | 89.89 | 5.94 | 93 | 297.74 | 3.85 | 0.15 | A | 4.00 | 5.25 | 2.62 |
| 2012 | 18_47 | 4 | 18_38 | 7 | IE | P | 408 | 600.56 | 2.98 | 1520 | 87.85 | 5.29 | 126 | 239.31 | 3.91 | 0.15 | A | 4.00 | 2.90 | 1.45 |
| 2012 | 18_47 | 4 | 18_38 | 7 | IE | P | 271 | 599.51 | 2.96 | 2332 | 90.10 | 5.63 | 82 | 321.59 | 4.35 | 0.15 | A | 4.00 | 6.28 | 3.14 |
| 2012 | 18_47 | 4 | 18_38 | 7 | IE | P | 386 | 595.23 | 2.69 | 1696 | 160.01 | 4.22 | 115 | 246.32 | 3.94 | 0.27 | AF | 7.12 |  |  |
| 2012 | 18_47 | 4 | 18_41 | 8 | IE | P | 226 | 611.50 | 2.88 | 1902 | 91.37 | 6.10 | 123 | 339.61 | 4.51 | 0.15 | A | 4.00 | 6.87 | 3.43 |
| 2012 | 18_47 | 4 | 18_41 | 8 | IE | P | 286 | 599.07 | 2.91 | 1411 | 92.52 | 6.58 | 100 | 328.98 | 4.33 | 0.15 | A | 4.00 | 6.22 | 3.11 |
| 2012 | 18_47 | 4 | 18_41 | 8 | IE | P | 297 | 608.22 | 2.88 | 2058 | 90.69 | 5.60 | 131 | 261.28 | 3.50 | 0.15 | A | 4.00 | 3.52 | 1.76 |
| 2012 | 18_47 | 4 | 18_44 | 8 | IE | P | 184 | 606.09 | 2.20 | 1637 | 89.46 | 5.88 | 127 | 327.48 | 5.79 | 0.15 | A | 4.00 | 6.64 | 3.32 |
| 2012 | 18_47 | 4 | 18_47 | 4 | S | P | 309 | 593.97 | 3.28 | 1802 | 89.37 | 6.60 | 112 | 265.02 | 3.92 | 0.15 | A | 4.00 | 3.86 | 1.93 |
| 2012 | 18_47 | 4 | 18_47 | 4 | S | P | 346 | 597.05 | 3.70 | 1676 | 89.74 | 7.34 | 116 | 273.59 | 4.48 | 0.15 | A | 4.00 | 4.19 | 2.10 |
| 2012 | 18_47 | 4 | 18_47 | 4 | S | P | 357 | 596.51 | 3.57 | 1791 | 90.20 | 6.57 | 110 | 273.73 | 4.66 | 0.15 | A | 4.00 | 4.14 | 2.07 |
| 2012 | 18_48 | 4 | 18_01 | 6 | IE | P | 203 | 600.18 | 2.56 | 2183 | 106.09 | 5.48 | 83 | 433.59 | 3.33 | 0.18 | A | 4.00 | 8.35 | 4.17 |
| 2012 | 18_48 | 4 | 18_05 | 6 | IE | P | 382 | 607.08 | 2.95 | 2296 | 91.80 | 5.69 | 81 | 388.34 | 4.68 | 0.15 | A | 4.00 | 8.92 | 4.46 |
| 2012 | 18_48 | 4 | 18_07 | 6 | IE | P | 193 | 604.12 | 2.55 | 1973 | 88.45 | 5.96 | 123 | 241.11 | 3.55 | 0.15 | A | 4.00 | 2.90 | 1.45 |
| 2012 | 18_48 | 4 | 18_07 | 6 | IE | P | 339 | 598.41 | 3.22 | 1989 | 89.96 | 5.97 | 113 | 235.91 | 4.37 | 0.15 | A | 4.00 | 2.49 | 1.24 |
| 2012 | 18_48 | 4 | 18_11 | 5 | IE | P | 317 | 599.44 | 2.72 | 1517 | 90.59 | 5.71 | 92 | 288.62 | 4.17 | 0.15 | A | 4.00 | 4.74 | 2.37 |
| 2012 | 18_48 | 4 | 18_11 | 5 | IE | P | 245 | 601.84 | 3.33 | 1749 | 90.80 | 5.70 | 123 | 236.38 | 4.64 | 0.15 | A | 4.00 | 2.41 | 1.21 |
| 2012 | 18_48 | 4 | 18_23 | 7 | IE | P | 398 | 593.14 | 2.85 | 1944 | 88.12 | 6.18 | 99 | 322.03 | 5.62 | 0.15 | A | 4.00 | 6.62 | 3.31 |
| 2012 | 18_48 | 4 | 18_23 | 7 | IE | P | 317 | 599.59 | 2.47 | 2061 | 88.75 | 5.36 | 93 | 312.58 | 3.06 | 0.15 | A | 4.00 | 6.09 | 3.04 |
| 2012 | 18_48 | 4 | 18_23 | 7 | IE | P | 265 | 608.93 | 2.92 | 2084 | 91.11 | 5.90 | 94 | 269.68 | 4.41 | 0.15 | A | 4.00 | 3.84 | 1.92 |
| 2012 | 18_48 | 4 | 18_27 | 8 | IE | P | 430 | 599.72 | 2.91 | 1431 | 171.08 | 4.35 | 125 | 259.28 | 4.07 | 0.29 | AF | 7.56 |  |  |
| 2012 | 18_48 | 4 | 18_27 | 8 | IE | P | 397 | 610.74 | 2.70 | 1467 | 174.94 | 4.77 | 119 | 260.96 | 4.02 | 0.29 | AF | 7.59 |  |  |
| 2012 | 18_48 | 4 | 18_32 | 6 | IE | P | 264 | 602.65 | 2.86 | 1904 | 90.97 | 6.08 | 86 | 299.68 | 4.78 | 0.15 | A | 4.00 | 5.18 | 2.59 |
| 2012 | 18_48 | 4 | 18_32 | 6 | IE | P | 188 | 611.91 | 2.64 | 2090 | 92.10 | 6.01 | 100 | 310.15 | 3.50 | 0.15 | A | 4.00 | 5.47 | 2.74 |
| 2012 | 18_48 | 4 | 18_32 | 6 | IE | P | 281 | 596.01 | 2.77 | 2006 | 87.49 | 5.95 | 105 | 291.50 | 3.71 | 0.15 | A | 4.00 | 5.33 | 2.66 |
| 2012 | 18_48 | 4 | 18_32 | 6 | IE | P | 270 | 609.40 | 2.69 | 2033 | 88.86 | 5.64 | 110 | 296.91 | 3.23 | 0.15 | A | 4.00 | 5.37 | 2.68 |
| 2012 | 18_48 | 4 | 18_32 | 6 | IE | P | 257 | 608.32 | 2.50 | 1774 | 92.30 | 5.77 | 83 | 308.51 | 3.61 | 0.15 | A | 4.00 | 5.37 | 2.68 |
| 2012 | 18_48 | 4 | 18_37 | 7 | IE | P | 263 | 615.49 | 2.50 | 2079 | 93.13 | 5.60 | 126 | 328.10 | 3.74 | 0.15 | A | 4.00 | 6.09 | 3.05 |
| 2012 | 18_48 | 4 | 18_37 | 7 | IE | P | 355 | 611.31 | 2.44 | 1775 | 90.48 | 6.26 | 122 | 248.96 | 3.53 | 0.15 | A | 4.00 | 3.01 | 1.50 |
| 2012 | 18_48 | 4 | 18_38 | 7 | IE | P | 322 | 597.37 | 2.92 | 1913 | 90.03 | 5.64 | 87 | 305.47 | 4.05 | 0.15 | A | 4.00 | 5.57 | 2.79 |
| 2012 | 18_48 | 4 | 18_38 | 7 | IE | P | 338 | 604.94 | 2.73 | 2006 | 90.22 | 5.89 | 144 | 260.27 | 3.64 | 0.15 | A | 4.00 | 3.54 | 1.77 |
| 2012 | 18_48 | 4 | 18_41 | 8 | IE | P | 289 | 609.97 | 2.62 | 2015 | 90.55 | 5.92 | 112 | 243.19 | 3.70 | 0.15 | A | 4.00 | 2.74 | 1.37 |
| 2012 | 18_48 | 4 | 18_41 | 8 | IE | P | 257 | 609.53 | 2.28 | 1989 | 89.95 | 5.75 | 133 | 254.34 | 3.78 | 0.15 | A | 4.00 | 3.31 | 1.66 |
| 2012 | 18_48 | 4 | 18_41 | 8 | IE | P | 285 | 611.06 | 2.40 | 2089 | 91.79 | 6.10 | 141 | 268.51 | 4.24 | 0.15 | A | 4.00 | 3.70 | 1.85 |
| 2012 | 18_48 | 4 | 18_41 | 8 | IE | P | 268 | 613.61 | 2.34 | 1694 | 91.23 | 5.68 | 155 | 342.80 | 4.71 | 0.15 | A | 4.00 | 7.03 | 3.52 |
| 2012 | 18_48 | 4 | 18_41 | 8 | IE | P | 386 | 589.52 | 2.94 | 1572 | 178.05 | 4.37 | 130 | 259.85 | 4.18 | 0.30 | AF | 8.00 |  |  |
| 2012 | 18_48 | 4 | 18_41 | 8 | IE | P | 412 | 595.42 | 2.50 | 1728 | 88.70 | 5.05 | 118 | 259.69 | 4.34 | 0.15 | A | 4.00 | 3.71 | 1.86 |
| 2012 | 18_48 | 4 | 18_44 | 8 | IE | P | 396 | 595.06 | 3.09 | 1550 | 170.90 | 4.80 | 89 | 472.56 | 4.10 | 0.29 | AS | 7.61 |  |  |
| 2012 | 18_48 | 4 | 18_48 | 4 | S | P | 293 | 595.11 | 3.24 | 1999 | 87.73 | 6.37 | 104 | 262.00 | 4.83 | 0.15 | A | 4.00 | 3.95 | 1.97 |
| 2012 | 18_48 | 4 | 18_48 | 4 | S | P | 325 | 600.84 | 3.61 | 1493 | 88.38 | 6.52 | 137 | 276.51 | 4.72 | 0.15 | A | 4.00 | 4.51 | 2.26 |
| 2012 | 18_48 | 4 | 18_48 | 4 | S | P | 330 | 601.90 | 3.14 | 1725 | 88.92 | 6.10 | 102 | 263.22 | 3.81 | 0.15 | A | 4.00 | 3.84 | 1.92 |
| 2012 | 18_48 | 4 | 18_48 | 4 | S | P | 366 | 598.31 | 3.21 | 1849 | 88.73 | 6.34 | 102 | 268.23 | 4.36 | 0.15 | A | 4.00 | 4.09 | 2.05 |
| 2012 | 27_02 | 7 | 27_02 | 7 | S | P | 341 | 599.34 | 3.24 | 1747 | 148.68 | 6.52 | 93 | 445.73 | 4.21 | 0.25 | A | 7.00 | 6.99 | 2.00 |
| 2012 | 27_02 | 7 | 27_02 | 7 | S | P | 349 | 591.85 | 2.89 | 1586 | 145.54 | 5.31 | 95 | 434.75 | 4.16 | 0.25 | A | 7.00 | 6.91 | 1.97 |
| 2012 | 27_02 | 7 | 27_02 | 7 | S | P | 502 | 589.68 | 2.46 | 1464 | 143.13 | 4.71 | 94 | 417.23 | 3.33 | 0.24 | A | 7.00 | 6.41 | 1.83 |
| 2012 | 27_02 | 7 | 27_02 | 7 | S | P | 535 | 596.26 | 2.70 | 1684 | 146.04 | 5.25 | 104 | 435.12 | 4.39 | 0.24 | A | 7.00 | 6.86 | 1.96 |
| 2012 | 27_02 | 7 | 27_02 | 7 | S | P | 454 | 603.98 | 3.00 | 1813 | 145.47 | 4.69 | 81 | 423.52 | 2.96 | 0.24 | A | 7.00 | 6.38 | 1.82 |
| 2012 | 27_02 | 7 | 27_04 | 7 | IA | P | 495 | 602.28 | 3.31 | 1751 | 147.26 | 5.32 | 130 | 420.42 | 3.78 | 0.24 | A | 7.00 | 5.98 | 1.71 |
| 2012 | 27_02 | 7 | 27_04 | 7 | IA | P | 356 | 606.29 | 2.88 | 1787 | 129.88 | 5.18 | 136 | 204.55 | 4.37 | 0.21 | S | 6.05 | 2.57 | 0.73 |
| 2012 | 27_02 | 7 | 27_26 | 7 | IA | P | 263 | 603.70 | 3.36 | 1421 | 146.30 | 5.21 | 80 | 420.89 | 4.18 | 0.24 | A | 7.00 | 6.14 | 1.75 |
| 2012 | 27_04 | 7 | 27_04 | 7 | S | P | 596 | 605.86 | 2.77 | 1106 | 216.31 | 4.01 | 104 | 365.29 | 5.25 | 0.36 | AF | 10.08 |  |  |
| 2012 | 27_04 | 7 | 27_04 | 7 | S | P | 607 | 605.23 | 2.81 | 1610 | 147.55 | 4.88 | 112 | 422.89 | 4.81 | 0.24 | A | 7.00 | 6.06 | 1.73 |
| 2012 | 27_04 | 7 | 27_04 | 7 | S | P | 530 | 603.56 | 2.79 | 1567 | 147.43 | 4.83 | 66 | 443.61 | 4.27 | 0.24 | A | 7.00 | 7.06 | 2.02 |
| 2012 | 27_04 | 7 | 27_13 | 7 | IA | P | 206 | 610.75 | 2.90 | 1961 | 152.25 | 5.22 | 119 | 426.19 | 4.02 | 0.25 | A | 7.00 | 5.59 | 1.60 |
| 2012 | 27_04 | 7 | 27_13 | 7 | IA | P | 355 | 612.36 | 3.47 | 1732 | 150.33 | 5.19 | 81 | 396.52 | 3.57 | 0.25 | A | 7.00 | 4.46 | 1.28 |
| 2012 | 27_04 | 7 | 27_13 | 7 | IA | P | 263 | 605.66 | 2.84 | 2127 | 148.03 | 4.61 | 58 | 445.28 | 3.56 | 0.24 | A | 7.00 | 7.06 | 2.02 |
| 2012 | 27_04 | 7 | 27_13 | 7 | IA | P | 378 | 604.63 | 3.44 | 1614 | 213.32 | 4.45 | 91 | 369.38 | 3.97 | 0.35 | AF | 9.97 |  |  |
| 2012 | 27_04 | 7 | 27_13 | 7 | IA | P | 238 | 606.49 | 3.26 | 1939 | 146.65 | 5.03 | 97 | 423.63 | 3.77 | 0.24 | A | 7.00 | 6.22 | 1.78 |
| 2012 | 27_04 | 7 | 27_13 | 7 | IA | P | 376 | 604.01 | 2.87 | 1833 | 146.83 | 4.87 | 95 | 380.21 | 3.57 | 0.24 | A | 7.00 | 4.13 | 1.18 |
| 2012 | 27_04 | 7 | 27_17 | 7 | IA | P | 378 | 600.48 | 3.16 | 1831 | 146.75 | 5.36 | 86 | 421.70 | 4.25 | 0.24 | A | 7.00 | 6.12 | 1.75 |
| 2012 | 27_04 | 7 | 27_17 | 7 | IA | P | 341 | 602.24 | 3.35 | 1519 | 146.61 | 5.57 | 59 | 416.04 | 5.92 | 0.24 | A | 7.00 | 5.86 | 1.68 |
| 2012 | 27_04 | 7 | 27_22 | 5 | IE | P | 243 | 605.07 | 2.34 | 2128 | 144.87 | 4.42 | 114 | 412.01 | 3.84 | 0.24 | A | 7.00 | 5.91 | 1.69 |
| 2012 | 27_04 | 7 | 27_22 | 5 | IE | P | 346 | 591.77 | 2.74 | 1817 | 142.86 | 4.86 | 92 | 429.95 | 4.61 | 0.24 | A | 7.00 | 7.07 | 2.02 |
| 2012 | 27_04 | 7 | 27_24 | 5 | IE | P | 374 | 608.13 | 3.34 | 1598 | 152.19 | 4.56 | 86 | 424.73 | 3.41 | 0.25 | A | 7.00 | 5.54 | 1.58 |
| 2012 | 27_04 | 7 | 27_26 | 7 | IA | P | 258 | 600.42 | 2.73 | 2185 | 71.90 | 7.02 | 161 | 275.45 | 4.22 | 0.12 | HP | 3.38 |  |  |
| 2012 | 27_04 | 7 | 27_26 | 7 | IA | P | 294 | 604.83 | 2.67 | 2073 | 142.89 | 4.46 | 97 | 412.74 | 3.53 | 0.24 | A | 7.00 | 6.22 | 1.78 |
| 2012 | 27_04 | 7 | 27_28 | 5 | IE | P | 477 | 603.75 | 2.64 | 2174 | 147.98 | 4.52 | 71 | 418.98 | 5.10 | 0.25 | A | 7.00 | 5.82 | 1.66 |
| 2012 | 27_06 | 5 | 27_04 | 7 | IE | P | 333 | 601.43 | 2.98 | 2094 | 105.75 | 4.84 | 141 | 342.45 | 3.73 | 0.18 | A | 5.00 | 6.19 | 2.48 |
| 2012 | 27_06 | 5 | 27_04 | 7 | IE | P | 257 | 597.88 | 2.80 | 1881 | 104.73 | 4.82 | 102 | 329.08 | 3.49 | 0.18 | A | 5.00 | 5.71 | 2.28 |
| 2012 | 27_06 | 5 | 27_04 | 7 | IE | P | 389 | 604.63 | 2.73 | 1946 | 105.37 | 5.25 | 90 | 343.42 | 3.20 | 0.17 | A | 5.00 | 6.30 | 2.52 |
| 2012 | 27_06 | 5 | 27_06 | 5 | S | P | 303 | 623.20 | 2.70 | 1721 | 116.97 | 5.32 | 102 | 339.45 | 3.77 | 0.19 | A | 5.00 | 4.51 | 1.80 |
| 2012 | 27_06 | 5 | 27_06 | 5 | S | P | 152 | 601.37 | 1.98 | 2229 | 107.19 | 4.77 | 77 | 418.49 | 2.84 | 0.18 | A | 5.00 | 9.52 | 3.81 |
| 2012 | 27_06 | 5 | 27_17 | 7 | IE | P | 219 | 606.56 | 3.05 | 1989 | 108.19 | 5.30 | 130 | 363.48 | 3.95 | 0.18 | A | 5.00 | 6.80 | 2.72 |
| 2012 | 27_06 | 5 | 27_22 | 5 | IA | P | 287 | 602.16 | 2.66 | 1997 | 110.05 | 5.62 | 145 | 318.82 | 3.33 | 0.18 | A | 5.00 | 4.49 | 1.79 |
| 2012 | 27_06 | 5 | 27_24 | 5 | IA | P | 423 | 610.14 | 2.85 | 1839 | 107.66 | 5.34 | 113 | 305.38 | 3.34 | 0.18 | A | 5.00 | 4.18 | 1.67 |
| 2012 | 27_13 | 7 | 27_02 | 7 | IA | P | 241 | 593.17 | 3.01 | 1961 | 72.15 | 7.09 | 104 | 273.26 | 3.51 | 0.12 | HP | 3.44 |  |  |
| 2012 | 27_13 | 7 | 27_04 | 7 | IA | P | 293 | 615.34 | 2.39 | 1982 | 146.19 | 4.17 | 96 | 416.99 | 3.82 | 0.24 | A | 7.00 | 5.97 | 1.70 |
| 2012 | 27_13 | 7 | 27_13 | 7 | S | P | 342 | 602.18 | 2.79 | 2052 | 142.59 | 4.66 | 107 | 415.53 | 4.30 | 0.24 | A | 7.00 | 6.40 | 1.83 |
| 2012 | 27_13 | 7 | 27_26 | 7 | IA | L | 199 | 706.35 | 2.65 | 2212 | 109.72 | 5.65 | 86 | 321.46 | 2.88 | 0.16 | A | 7.00 | 6.51 | 1.86 |
| 2012 | 27_17 | 7 | 27_02 | 7 | IA | P | 294 | 593.38 | 3.06 | 2015 | 140.69 | 4.35 | 70 | 404.67 | 3.64 | 0.24 | A | 7.00 | 6.13 | 1.75 |
| 2012 | 27_17 | 7 | 27_17 | 7 | S | L | 221 | 696.19 | 3.26 | 1838 | 110.46 | 5.36 | 75 | 340.54 | 4.10 | 0.16 | A | 7.00 | 7.58 | 2.17 |
| 2012 | 27_17 | 7 | 27_17 | 7 | S | P | 363 | 603.48 | 2.16 | 1711 | 145.34 | 4.43 | 93 | 421.62 | 4.43 | 0.24 | A | 7.00 | 6.31 | 1.80 |
| 2012 | 27_17 | 7 | 27_17 | 7 | S | P | 341 | 600.60 | 2.64 | 1841 | 144.75 | 4.59 | 81 | 429.30 | 4.25 | 0.24 | A | 7.00 | 6.76 | 1.93 |
| 2012 | 27_17 | 7 | 27_28 | 5 | IE | P | 388 | 606.46 | 2.45 | 1668 | 140.74 | 4.69 | 86 | 405.86 | 4.51 | 0.23 | A | 7.00 | 6.19 | 1.77 |
| 2012 | 27_22 | 5 | 27_02 | 7 | IE | P | 225 | 605.90 | 2.74 | 1573 | 106.43 | 5.49 | 76 | 356.33 | 3.48 | 0.18 | A | 5.00 | 6.74 | 2.70 |
| 2012 | 27_22 | 5 | 27_13 | 7 | IE | P | 288 | 602.13 | 3.41 | 1457 | 167.74 | 4.46 | 120 | 279.37 | 4.37 | 0.28 | AF | 7.73 |  |  |
| 2012 | 27_22 | 5 | 27_22 | 5 | S | P | 238 | 596.63 | 2.12 | 1687 | 114.16 | 5.07 | 174 | 166.57 | 4.05 | 0.19 | S | 5.31 | 2.87 | 1.15 |
| 2012 | 27_24 | 5 | 27_02 | 7 | IE | P | 286 | 601.42 | 3.12 | 2074 | 106.30 | 5.66 | 114 | 344.19 | 3.93 | 0.18 | A | 5.00 | 6.19 | 2.48 |
| 2012 | 27_24 | 5 | 27_02 | 7 | IE | P | 297 | 598.18 | 3.16 | 1934 | 105.08 | 5.39 | 81 | 272.97 | 3.28 | 0.18 | A | 5.00 | 2.99 | 1.20 |
| 2012 | 27_24 | 5 | 27_02 | 7 | IE | P | 259 | 603.12 | 3.22 | 2111 | 106.94 | 5.62 | 76 | 351.51 | 4.31 | 0.18 | A | 5.00 | 6.43 | 2.57 |
| 2012 | 27_24 | 5 | 27_13 | 7 | IE | P | 229 | 607.44 | 2.65 | 1760 | 106.25 | 5.42 | 79 | 366.63 | 2.84 | 0.17 | A | 5.00 | 7.25 | 2.90 |
| 2012 | 27_24 | 5 | 27_13 | 7 | IE | P | 292 | 590.13 | 3.07 | 1865 | 104.32 | 5.76 | 81 | 297.87 | 2.81 | 0.18 | A | 5.00 | 4.28 | 1.71 |
| 2012 | 27_24 | 5 | 27_13 | 7 | IE | P | 237 | 598.96 | 2.24 | 1735 | 104.88 | 5.17 | 102 | 328.53 | 3.45 | 0.18 | A | 5.00 | 5.66 | 2.26 |
| 2012 | 27_24 | 5 | 27_24 | 5 | S | P | 256 | 596.47 | 2.89 | 1885 | 110.55 | 6.38 | 152 | 341.32 | 4.52 | 0.19 | A | 5.00 | 5.44 | 2.17 |
| 2012 | 27_24 | 5 | 27_28 | 5 | IA | P | 289 | 602.58 | 2.53 | 1765 | 107.56 | 5.04 | 88 | 344.91 | 3.96 | 0.18 | A | 5.00 | 6.03 | 2.41 |
| 2012 | 27_26 | 7 | 27_02 | 7 | IA | P | 279 | 594.47 | 3.30 | 2085 | 148.36 | 4.61 | 107 | 422.02 | 3.94 | 0.25 | A | 7.00 | 5.91 | 1.69 |
| 2012 | 27_26 | 7 | 27_02 | 7 | IA | P | 157 | 601.90 | 2.55 | 2076 | 144.34 | 4.56 | 150 | 218.13 | 3.82 | 0.24 | S | 6.77 | 3.31 | 0.95 |
| 2012 | 27_26 | 7 | 27_02 | 7 | IA | P | 341 | 613.04 | 2.64 | 1961 | 158.91 | 4.65 | 140 | 241.00 | 4.22 | 0.26 | S | 7.32 | 3.54 | 1.01 |
| 2012 | 27_26 | 7 | 27_02 | 7 | IA | P | 280 | 592.63 | 2.76 | 1979 | 149.16 | 4.65 | 104 | 446.57 | 4.01 | 0.25 | A | 7.00 | 6.96 | 1.99 |
| 2012 | 27_26 | 7 | 27_02 | 7 | IA | P | 317 | 589.73 | 2.55 | 2030 | 75.54 | 6.06 | 231 | 220.15 | 4.25 | 0.13 | HP | 3.62 |  |  |
| 2012 | 27_26 | 7 | 27_02 | 7 | IA | P | 236 | 601.64 | 2.12 | 2021 | 134.13 | 4.26 | 111 | 202.85 | 3.28 | 0.22 | S | 6.30 | 3.07 | 0.88 |
| 2012 | 27_26 | 7 | 27_02 | 7 | IA | P | 138 | 604.62 | 2.12 | 2319 | 86.89 | 5.79 | 155 | 314.36 | 3.14 | 0.14 | HP | 4.06 |  |  |
| 2012 | 27_26 | 7 | 27_02 | 7 | IA | P | 240 | 596.28 | 2.96 | 2039 | 137.25 | 4.82 | 168 | 212.02 | 3.57 | 0.23 | S | 6.50 | 2.96 | 0.85 |
| 2012 | 27_26 | 7 | 27_02 | 7 | IA | P | 293 | 597.78 | 1.90 | 2263 | 139.89 | 4.11 | 137 | 214.51 | 3.57 | 0.23 | S | 6.61 | 3.08 | 0.88 |
| 2012 | 27_26 | 7 | 27_02 | 7 | IA | P | 333 | 606.44 | 2.82 | 1958 | 154.12 | 5.16 | 75 | 456.05 | 3.49 | 0.25 | A | 7.00 | 6.71 | 1.92 |
| 2012 | 27_26 | 7 | 27_04 | 7 | IA | P | 330 | 614.36 | 2.41 | 1736 | 152.02 | 4.47 | 95 | 438.23 | 3.39 | 0.25 | A | 7.00 | 6.18 | 1.77 |
| 2012 | 27_26 | 7 | 27_04 | 7 | IA | P | 304 | 608.85 | 2.51 | 1620 | 73.96 | 7.51 | 252 | 215.98 | 4.01 | 0.12 | HP | 3.43 |  |  |
| 2012 | 27_26 | 7 | 27_04 | 7 | IA | P | 255 | 604.38 | 2.42 | 1707 | 141.94 | 4.23 | 195 | 212.12 | 4.10 | 0.23 | S | 6.63 | 3.35 | 0.96 |
| 2012 | 27_26 | 7 | 27_17 | 7 | IA | P | 439 | 588.67 | 3.01 | 1183 | 148.78 | 5.94 | 150 | 417.79 | 4.97 | 0.25 | A | 7.00 | 5.66 | 1.62 |
| 2012 | 27_26 | 7 | 27_17 | 7 | IA | P | 198 | 611.06 | 2.23 | 2038 | 159.68 | 4.56 | 172 | 249.82 | 4.33 | 0.26 | S | 7.38 | 3.21 | 0.92 |
| 2012 | 27_26 | 7 | 27_17 | 7 | IA | L | 126 | 714.75 | 3.58 | 1758 | 114.35 | 5.24 | 148 | 173.89 | 3.96 | 0.16 | S | 4.52 | 2.17 | 0.62 |
| 2012 | 27_26 | 7 | 27_17 | 7 | IA | P | 237 | 601.48 | 2.22 | 1645 | 146.34 | 5.00 | 181 | 236.37 | 3.98 | 0.24 | S | 6.87 | 2.64 | 0.76 |
| 2012 | 27_26 | 7 | 27_17 | 7 | IA | P | 232 | 597.13 | 2.07 | 1854 | 142.45 | 4.13 | 128 | 222.43 | 3.64 | 0.24 | S | 6.74 | 2.95 | 0.84 |
| 2012 | 27_26 | 7 | 27_17 | 7 | IA | P | 254 | 596.76 | 2.25 | 1978 | 133.96 | 4.58 | 110 | 205.79 | 4.02 | 0.22 | S | 6.34 | 2.94 | 0.84 |
| 2012 | 27_26 | 7 | 27_17 | 7 | IA | P | 282 | 606.78 | 1.77 | 1840 | 150.82 | 4.06 | 95 | 438.16 | 3.71 | 0.25 | A | 7.00 | 6.34 | 1.81 |
| 2012 | 27_26 | 7 | 27_17 | 7 | IA | P | 422 | 595.58 | 2.16 | 1624 | 142.50 | 4.41 | 108 | 220.10 | 3.51 | 0.24 | S | 6.76 | 3.08 | 0.88 |
| 2012 | 27_26 | 7 | 27_17 | 7 | IA | P | 385 | 597.34 | 1.83 | 1608 | 132.07 | 4.60 | 137 | 199.32 | 3.52 | 0.22 | S | 6.24 | 3.07 | 0.88 |
| 2012 | 27_26 | 7 | 27_22 | 5 | IE | P | 357 | 601.54 | 2.12 | 1464 | 145.11 | 4.34 | 45 | 423.97 | 3.21 | 0.24 | A | 7.00 | 6.45 | 1.84 |
| 2012 | 27_26 | 7 | 27_22 | 5 | IE | P | 290 | 601.34 | 1.94 | 1555 | 144.58 | 4.13 | 137 | 218.28 | 3.59 | 0.24 | S | 6.79 | 3.33 | 0.95 |
| 2012 | 27_26 | 7 | 27_26 | 7 | S | P | 497 | 607.14 | 2.61 | 1542 | 147.22 | 4.18 | 76 | 445.07 | 3.64 | 0.24 | A | 7.00 | 7.16 | 2.05 |
| 2012 | 27_26 | 7 | 27_26 | 7 | S | P | 312 | 607.82 | 2.62 | 1845 | 146.86 | 4.27 | 61 | 441.33 | 2.73 | 0.24 | A | 7.00 | 7.04 | 2.01 |
| 2012 | 27_26 | 7 | 27_26 | 7 | S | P | 524 | 600.92 | 2.82 | 1640 | 146.68 | 4.88 | 88 | 442.41 | 4.00 | 0.24 | A | 7.00 | 7.11 | 2.03 |
| 2012 | 27_26 | 7 | 27_26 | 7 | S | P | 351 | 605.94 | 3.05 | 1647 | 137.23 | 4.65 | 109 | 206.97 | 3.86 | 0.23 | S | 6.40 | 3.15 | 0.90 |
| 2012 | 27_26 | 7 | 27_26 | 7 | S | P | 349 | 600.61 | 2.60 | 1360 | 129.43 | 5.15 | 55 | 196.32 | 3.07 | 0.22 | S | 6.09 | 2.94 | 0.84 |
| 2012 | 27_26 | 7 | 27_28 | 5 | IE | P | 248 | 611.19 | 2.25 | 1733 | 70.22 | 6.96 | 156 | 209.73 | 3.85 | 0.11 | HP | 3.25 |  |  |
| 2012 | 27_28 | 5 | 27_04 | 7 | IE | P | 204 | 596.98 | 2.92 | 1966 | 105.19 | 5.72 | 94 | 330.56 | 3.60 | 0.18 | A | 5.00 | 5.71 | 2.29 |
| 2012 | 27_28 | 5 | 27_17 | 7 | IE | P | 139 | 603.05 | 2.88 | 1567 | 104.89 | 5.12 | 39 | 516.77 | 3.49 | 0.17 | A | 5.00 | 14.63 | 5.85 |
| 2012 | 27_28 | 5 | 27_17 | 7 | IE | P | 322 | 601.14 | 2.58 | 1670 | 104.15 | 5.31 | 82 | 352.85 | 3.84 | 0.17 | A | 5.00 | 6.94 | 2.78 |
| 2012 | 27_28 | 5 | 27_22 | 5 | IA | P | 292 | 603.19 | 2.28 | 1681 | 106.06 | 4.89 | 81 | 310.66 | 3.30 | 0.18 | A | 5.00 | 4.65 | 1.86 |
| 2012 | 27_28 | 5 | 27_28 | 5 | S | P | 202 | 602.27 | 2.30 | 2306 | 105.97 | 5.14 | 128 | 325.06 | 4.21 | 0.18 | A | 5.00 | 5.34 | 2.13 |
| 2012 | 27_28 | 5 | 27_28 | 5 | S | P | 216 | 601.59 | 2.35 | 2091 | 106.44 | 5.21 | 109 | 317.66 | 3.96 | 0.18 | A | 5.00 | 4.92 | 1.97 |
| 2012 | 37_05 | 6 | 37_14 | 6 | IA | P | 227 | 598.00 | 2.01 | 1927 | 130.75 | 4.93 | 105 | 384.19 | 3.80 | 0.22 | A | 6.00 | 5.63 | 1.88 |
| 2012 | 37_06 | 5 | 37_05 | 6 | IE | P | 351 | 590.55 | 3.47 | 2237 | 104.66 | 5.94 | 105 | 335.86 | 3.88 | 0.18 | A | 5.00 | 6.05 | 2.42 |
| 2012 | 37_06 | 5 | 37_05 | 6 | IE | P | 333 | 608.07 | 2.84 | 1969 | 107.13 | 5.48 | 95 | 324.62 | 3.95 | 0.18 | A | 5.00 | 5.15 | 2.06 |
| 2012 | 37_06 | 5 | 37_12 | 5 | IA | P | 258 | 605.32 | 2.32 | 1931 | 106.04 | 4.99 | 108 | 328.25 | 4.59 | 0.18 | A | 5.00 | 5.48 | 2.19 |
| 2012 | 37_06 | 5 | 37_14 | 6 | IE | P | 262 | 605.61 | 3.37 | 2242 | 107.28 | 6.22 | 108 | 438.24 | 4.35 | 0.18 | A | 5.00 | 10.43 | 4.17 |
| 2012 | 37_06 | 5 | 37_14 | 6 | IE | P | 287 | 601.62 | 2.74 | 2017 | 104.42 | 4.93 | 90 | 328.28 | 2.96 | 0.17 | A | 5.00 | 5.72 | 2.29 |
| 2012 | 37_09 | 5 | 37_05 | 6 | IE | P | 514 | 599.53 | 2.96 | 1706 | 107.51 | 5.45 | 109 | 334.11 | 3.04 | 0.18 | A | 5.00 | 5.54 | 2.22 |
| 2012 | 37_09 | 5 | 37_09 | 5 | S | P | 271 | 591.10 | 2.65 | 2081 | 106.10 | 4.91 | 97 | 327.54 | 2.73 | 0.18 | A | 5.00 | 5.44 | 2.17 |
| 2012 | 37_09 | 5 | 37_14 | 6 | IE | P | 372 | 608.80 | 2.25 | 1040 | 207.47 | 3.53 | 53 | 533.09 | 2.39 | 0.34 | AS | 9.46 |  |  |
| 2012 | 37_09 | 5 | 37_14 | 6 | IE | P | 346 | 610.79 | 2.36 | 1551 | 108.21 | 5.29 | 93 | 274.57 | 3.10 | 0.18 | A | 5.00 | 2.69 | 1.07 |
| 2012 | 37_09 | 5 | 37_14 | 6 | IE | P | 277 | 612.11 | 2.55 | 1718 | 109.94 | 6.23 | 83 | 340.45 | 3.23 | 0.18 | A | 5.00 | 5.48 | 2.19 |
| 2012 | 37_09 | 5 | 37_14 | 6 | IE | P | 1290 | 595.08 | 1.65 | 723 | 204.59 | 3.71 | 74 | 343.06 | 3.09 | 0.34 | S | 9.54 | 3.08 | 1.23 |
| 2012 | 37_09 | 5 | 37_14 | 6 | IE | P | 1314 | 589.00 | 1.78 | 764 | 104.78 | 4.98 | 85 | 279.22 | 3.43 | 0.18 | A | 5.00 | 3.32 | 1.33 |
| 2012 | 37_09 | 5 | 37_14 | 6 | IE | P | 1097 | 591.64 | 1.89 | 1111 | 103.88 | 3.99 | 50 | 270.43 | 3.36 | 0.18 | A | 5.00 | 3.02 | 1.21 |
| 2012 | 37_09 | 5 | 37_14 | 6 | IE | P | 1103 | 601.84 | 2.10 | 1187 | 107.54 | 4.48 | 84 | 284.42 | 3.50 | 0.18 | A | 5.00 | 3.22 | 1.29 |
| 2012 | 37_09 | 5 | 37_14 | 6 | IE | P | 1125 | 603.49 | 1.98 | 1031 | 107.63 | 4.17 | 83 | 284.90 | 5.05 | 0.18 | A | 5.00 | 3.24 | 1.29 |
| 2012 | 37_09 | 5 | 37_14 | 6 | IE | P | 1268 | 590.70 | 2.24 | 924 | 165.15 | 3.23 | 87 | 277.91 | 4.53 | 0.28 | AF | 7.76 |  |  |
| 2012 | 37_09 | 5 | 37_14 | 6 | IE | P | 1104 | 622.97 | 2.01 | 1008 | 110.40 | 4.11 | 74 | 298.12 | 4.53 | 0.18 | A | 5.00 | 3.50 | 1.40 |
| 2012 | 37_09 | 5 | 37_16 | 6 | IE | P | 1051 | 595.07 | 2.14 | 1194 | 107.57 | 4.43 | 81 | 287.91 | 3.84 | 0.18 | A | 5.00 | 3.38 | 1.35 |
| 2012 | 37_09 | 5 | 37_16 | 6 | IE | P | 854 | 591.12 | 1.91 | 1369 | 102.25 | 4.89 | 94 | 269.92 | 4.19 | 0.17 | A | 5.00 | 3.20 | 1.28 |
| 2012 | 37_09 | 5 | 37_16 | 6 | IE | P | 689 | 604.86 | 2.02 | 1217 | 108.80 | 4.63 | 80 | 353.68 | 2.81 | 0.18 | A | 5.00 | 6.25 | 2.50 |
| 2012 | 37_09 | 5 | 37_16 | 6 | IE | P | 811 | 598.78 | 2.21 | 1088 | 107.68 | 4.87 | 112 | 353.08 | 5.50 | 0.18 | A | 5.00 | 6.39 | 2.56 |
| 2012 | 37_09 | 5 | 37_16 | 6 | IE | P | 964 | 598.56 | 1.87 | 1073 | 106.70 | 4.87 | 95 | 285.28 | 4.29 | 0.18 | A | 5.00 | 3.37 | 1.35 |
| 2012 | 37_09 | 5 | 37_16 | 6 | IE | P | 564 | 595.86 | 1.95 | 1448 | 106.89 | 4.89 | 94 | 336.41 | 3.47 | 0.18 | A | 5.00 | 5.74 | 2.29 |
| 2012 | 37_09 | 5 | 37_16 | 6 | IE | P | 695 | 587.92 | 2.28 | 1204 | 106.65 | 5.13 | 78 | 281.73 | 4.67 | 0.18 | A | 5.00 | 3.21 | 1.28 |
| 2012 | 37_09 | 5 | 37_16 | 6 | IE | P | 241 | 606.34 | 2.42 | 822 | 108.36 | 5.04 | 48 | 334.32 | 2.37 | 0.18 | A | 5.00 | 5.43 | 2.17 |
| 2012 | 37_09 | 5 | 37_16 | 6 | IE | P | 563 | 590.17 | 2.39 | 1597 | 106.59 | 5.77 | 102 | 283.74 | 5.00 | 0.18 | A | 5.00 | 3.31 | 1.32 |
| 2012 | 37_10 | 5 | 37_05 | 6 | IE | P | 454 | 597.31 | 2.82 | 1551 | 106.71 | 5.26 | 127 | 340.26 | 4.40 | 0.18 | A | 5.00 | 5.94 | 2.38 |
| 2012 | 37_10 | 5 | 37_05 | 6 | IE | P | 426 | 604.36 | 2.99 | 1719 | 107.85 | 5.64 | 119 | 445.46 | 5.10 | 0.18 | A | 5.00 | 10.65 | 4.26 |
| 2012 | 37_12 | 5 | 37_16 | 6 | IE | P | 200 | 600.63 | 2.48 | 1793 | 105.68 | 4.78 | 101 | 323.17 | 3.06 | 0.18 | A | 5.00 | 5.29 | 2.12 |
| 2012 | 37_14 | 6 | 37_10 | 5 | IE | P | 288 | 605.23 | 2.84 | 2019 | 125.77 | 5.11 | 100 | 376.33 | 3.61 | 0.21 | A | 6.00 | 5.95 | 1.98 |
| 2012 | 37_14 | 6 | 37_14 | 6 | S | P | 240 | 608.89 | 3.33 | 1927 | 129.94 | 6.47 | 96 | 381.00 | 4.63 | 0.21 | A | 6.00 | 5.59 | 1.86 |
| 2012 | 37_14 | 6 | 37_14 | 6 | S | P | 227 | 608.69 | 2.63 | 1998 | 128.62 | 5.80 | 109 | 377.20 | 3.83 | 0.21 | A | 6.00 | 5.60 | 1.87 |
| 2012 | 37_16 | 6 | 37_05 | 6 | IA | P | 215 | 609.09 | 2.02 | 2018 | 131.02 | 4.87 | 96 | 386.02 | 3.11 | 0.22 | A | 6.00 | 5.68 | 1.89 |
| 2012 | 37_16 | 6 | 37_06 | 5 | IE | P | 322 | 605.06 | 2.29 | 1906 | 131.98 | 4.44 | 146 | 384.44 | 3.71 | 0.22 | A | 6.00 | 5.48 | 1.83 |
| 2012 | 37_16 | 6 | 37_06 | 5 | IE | P | 308 | 601.35 | 1.87 | 1836 | 128.43 | 4.00 | 84 | 499.85 | 2.60 | 0.21 | A | 6.00 | 11.35 | 3.78 |
| 2012 | 37_16 | 6 | 37_06 | 5 | IE | P | 219 | 601.62 | 1.75 | 1314 | 128.96 | 3.54 | 88 | 380.17 | 3.32 | 0.21 | A | 6.00 | 5.69 | 1.90 |
| 2012 | 37_16 | 6 | 37_06 | 5 | IE | P | 364 | 602.70 | 1.80 | 1325 | 130.61 | 4.41 | 91 | 388.91 | 3.43 | 0.22 | A | 6.00 | 5.87 | 1.96 |
| 2012 | 37_16 | 6 | 37_06 | 5 | IE | P | 298 | 604.97 | 2.05 | 1929 | 130.67 | 5.09 | 104 | 387.88 | 2.59 | 0.22 | A | 6.00 | 5.81 | 1.94 |
| 2012 | 37_16 | 6 | 37_06 | 5 | IE | P | 299 | 609.61 | 1.85 | 2128 | 130.71 | 4.28 | 113 | 384.04 | 3.58 | 0.21 | A | 6.00 | 5.63 | 1.88 |
| 2012 | 37_16 | 6 | 37_06 | 5 | IE | P | 251 | 609.46 | 1.95 | 1686 | 131.63 | 4.69 | 83 | 381.02 | 3.68 | 0.22 | A | 6.00 | 5.37 | 1.79 |
| 2012 | 37_16 | 6 | 37_06 | 5 | IE | P | 329 | 605.94 | 1.94 | 1988 | 128.91 | 4.00 | 90 | 377.66 | 3.25 | 0.21 | A | 6.00 | 5.58 | 1.86 |
| 2012 | 37_16 | 6 | 37_06 | 5 | IE | P | 248 | 600.33 | 1.99 | 1844 | 130.09 | 4.24 | 143 | 392.64 | 3.54 | 0.22 | A | 6.00 | 6.11 | 2.04 |
| 2012 | 37_16 | 6 | 37_09 | 5 | IE | P | 462 | 602.24 | 1.77 | 1635 | 129.39 | 4.41 | 83 | 376.13 | 2.86 | 0.21 | A | 6.00 | 5.44 | 1.81 |
| 2012 | 37_16 | 6 | 37_09 | 5 | IE | P | 508 | 598.20 | 1.83 | 1318 | 129.37 | 4.21 | 100 | 387.31 | 3.77 | 0.22 | A | 6.00 | 5.96 | 1.99 |
| 2012 | 37_16 | 6 | 37_09 | 5 | IE | P | 366 | 597.83 | 1.89 | 1872 | 130.36 | 5.09 | 127 | 382.10 | 4.01 | 0.22 | A | 6.00 | 5.59 | 1.86 |
| 2012 | 37_16 | 6 | 37_09 | 5 | IE | P | 463 | 599.59 | 1.97 | 1698 | 131.01 | 4.72 | 104 | 389.00 | 3.99 | 0.22 | A | 6.00 | 5.82 | 1.94 |
| 2012 | 37_16 | 6 | 37_09 | 5 | IE | P | 248 | 595.67 | 1.95 | 1650 | 128.54 | 4.63 | 105 | 381.34 | 3.10 | 0.22 | A | 6.00 | 5.80 | 1.93 |
| 2012 | 37_16 | 6 | 37_09 | 5 | IE | P | 403 | 594.91 | 2.22 | 1676 | 130.37 | 5.36 | 110 | 388.33 | 3.78 | 0.22 | A | 6.00 | 5.87 | 1.96 |
| 2012 | 37_16 | 6 | 37_09 | 5 | IE | P | 496 | 599.56 | 2.56 | 1649 | 130.13 | 4.88 | 82 | 329.27 | 3.16 | 0.22 | A | 6.00 | 3.18 | 1.06 |
| 2012 | 37_16 | 6 | 37_09 | 5 | IE | P | 527 | 595.12 | 2.08 | 1799 | 129.26 | 4.74 | 117 | 333.19 | 3.01 | 0.22 | A | 6.00 | 3.47 | 1.16 |
| 2012 | 37_16 | 6 | 37_09 | 5 | IE | P | 342 | 596.72 | 2.04 | 1969 | 129.34 | 4.43 | 99 | 388.38 | 3.34 | 0.22 | A | 6.00 | 6.02 | 2.01 |
| 2012 | 37_16 | 6 | 37_09 | 5 | IE | P | 449 | 596.14 | 2.40 | 1717 | 130.46 | 6.09 | 117 | 381.47 | 3.36 | 0.22 | A | 6.00 | 5.54 | 1.85 |
| 2012 | 37_16 | 6 | 37_10 | 5 | IE | P | 378 | 603.16 | 3.15 | 1912 | 126.92 | 4.77 | 126 | 375.66 | 3.09 | 0.21 | A | 6.00 | 5.76 | 1.92 |
| 2012 | 37_16 | 6 | 37_14 | 6 | IA | P | 436 | 610.52 | 2.83 | 1724 | 130.89 | 4.83 | 108 | 384.16 | 4.23 | 0.21 | A | 6.00 | 5.61 | 1.87 |
| 2012 | 37_16 | 6 | 37_14 | 6 | IA | P | 389 | 607.63 | 3.10 | 1915 | 130.34 | 5.02 | 110 | 373.58 | 3.51 | 0.21 | A | 6.00 | 5.20 | 1.73 |
| 2012 | 37_16 | 6 | 37_16 | 6 | S | P | 298 | 596.08 | 2.61 | 1818 | 127.32 | 5.28 | 115 | 375.20 | 3.27 | 0.21 | A | 6.00 | 5.68 | 1.89 |
| 2012 | 37_16 | 6 | 37_16 | 6 | S | P | 193 | 608.11 | 2.34 | 2023 | 128.79 | 5.24 | 120 | 385.67 | 3.19 | 0.21 | A | 6.00 | 5.97 | 1.99 |
| 2012 | 37_16 | 6 | 37_16 | 6 | S | P | 214 | 608.77 | 2.30 | 1891 | 131.45 | 5.92 | 117 | 383.47 | 3.29 | 0.22 | A | 6.00 | 5.50 | 1.83 |
| 2012 | 37_16 | 6 | 37_16 | 6 | S | P | 319 | 589.40 | 2.89 | 1933 | 126.05 | 5.56 | 112 | 375.98 | 4.89 | 0.21 | A | 6.00 | 5.90 | 1.97 |
| 2012 | 37_16 | 6 | 37_16 | 6 | S | P | 321 | 595.14 | 2.12 | 1822 | 126.69 | 5.26 | 118 | 496.46 | 3.30 | 0.21 | A | 6.00 | 11.51 | 3.84 |
| 2012 | 37_16 | 6 | 37_16 | 6 | S | P | 326 | 588.68 | 2.48 | 1872 | 129.13 | 5.30 | 117 | 386.61 | 4.11 | 0.22 | A | 6.00 | 5.96 | 1.99 |
| 2012 | 37_16 | 6 | 37_16 | 6 | S | P | 295 | 597.62 | 2.57 | 2068 | 127.48 | 4.77 | 107 | 375.30 | 3.61 | 0.21 | A | 6.00 | 5.66 | 1.89 |
| 2012 | 41_02 | 8 | 41_02 | 8 | S | L | 159 | 712.25 | 2.63 | 2185 | 125.32 | 4.80 | 89 | 369.57 | 4.07 | 0.18 | A | 8.00 | 7.59 | 1.90 |
| 2012 | 41_02 | 8 | 41_02 | 8 | S | L | 277 | 702.25 | 3.64 | 2091 | 125.12 | 5.13 | 65 | 372.38 | 3.29 | 0.18 | A | 8.00 | 7.81 | 1.95 |
| 2012 | 41_02 | 8 | 41_02 | 8 | S | L | 148 | 722.46 | 2.95 | 2031 | 127.77 | 4.33 | 65 | 378.35 | 2.28 | 0.18 | A | 8.00 | 7.69 | 1.92 |
| 2012 | 41_02 | 8 | 41_04 | 5 | IE | L | 193 | 692.20 | 3.31 | 1849 | 121.65 | 4.99 | 82 | 288.73 | 4.54 | 0.18 | A | 8.00 | 2.99 | 0.75 |
| 2012 | 41_02 | 8 | 41_10 | 6 | IE | P | 356 | 597.66 | 2.03 | 2178 | 168.40 | 3.97 | 103 | 454.54 | 3.22 | 0.28 | A | 8.00 | 5.59 | 1.40 |
| 2012 | 41_02 | 8 | 41_10 | 6 | IE | P | 408 | 593.20 | 2.39 | 1463 | 169.37 | 3.59 | 104 | 454.38 | 7.21 | 0.29 | A | 8.00 | 5.46 | 1.37 |
| 2012 | 41_02 | 8 | 41_10 | 6 | IE | P | 566 | 589.74 | 2.45 | 1658 | 173.06 | 4.26 | 77 | 468.45 | 2.52 | 0.29 | A | 8.00 | 5.65 | 1.41 |
| 2012 | 41_02 | 8 | 41_10 | 6 | IE | P | 653 | 588.71 | 2.48 | 1212 | 154.45 | 4.80 | 175 | 451.08 | 3.44 | 0.26 | A | 8.00 | 7.36 | 1.84 |
| 2012 | 41_02 | 8 | 41_10 | 6 | IE | P | 506 | 597.45 | 2.53 | 1493 | 171.99 | 5.04 | 62 | 466.03 | 3.22 | 0.29 | A | 8.00 | 5.68 | 1.42 |
| 2012 | 41_02 | 8 | 41_10 | 6 | IE | P | 440 | 603.65 | 2.68 | 1665 | 174.36 | 4.81 | 89 | 472.49 | 3.73 | 0.29 | A | 8.00 | 5.68 | 1.42 |
| 2012 | 41_02 | 8 | 41_10 | 6 | IE | P | 449 | 596.11 | 2.60 | 1444 | 171.49 | 4.36 | 96 | 467.64 | 5.47 | 0.29 | A | 8.00 | 5.82 | 1.45 |
| 2012 | 41_02 | 8 | 41_10 | 6 | IE | P | 332 | 597.84 | 2.79 | 1919 | 168.76 | 3.93 | 90 | 459.03 | 4.11 | 0.28 | A | 8.00 | 5.76 | 1.44 |
| 2012 | 41_02 | 8 | 41_11 | 5 | IE | L | 218 | 707.20 | 2.42 | 1674 | 123.23 | 4.38 | 80 | 349.67 | 3.66 | 0.17 | A | 8.00 | 6.70 | 1.68 |
| 2012 | 41_02 | 8 | 41_11 | 5 | IE | L | 158 | 700.79 | 3.09 | 1992 | 122.71 | 4.92 | 92 | 361.55 | 4.00 | 0.18 | A | 8.00 | 7.57 | 1.89 |
| 2012 | 41_02 | 8 | 41_13 | 5 | IE | L | 247 | 681.04 | 2.77 | 2046 | 119.76 | 5.13 | 85 | 291.24 | 4.49 | 0.18 | A | 8.00 | 3.45 | 0.86 |
| 2012 | 41_02 | 8 | 41_15 | 8 | IA | L | 236 | 707.24 | 3.17 | 1679 | 126.34 | 4.67 | 87 | 372.06 | 3.89 | 0.18 | A | 8.00 | 7.56 | 1.89 |
| 2012 | 41_02 | 8 | 41_30 | 6 | IE | P | 421 | 596.72 | 2.47 | 1719 | 173.38 | 4.73 | 80 | 466.85 | 4.44 | 0.29 | A | 8.00 | 5.54 | 1.39 |
| 2012 | 41_02 | 8 | 41_30 | 6 | IE | P | 465 | 599.84 | 2.29 | 1637 | 173.98 | 4.96 | 107 | 475.91 | 3.32 | 0.29 | A | 8.00 | 5.88 | 1.47 |
| 2012 | 41_02 | 8 | 41_30 | 6 | IE | P | 673 | 604.36 | 3.29 | 1077 | 240.93 | 4.49 | 94 | 414.30 | 4.73 | 0.40 | AF | 17.12 |  |  |
| 2012 | 41_02 | 8 | 41_30 | 6 | IE | P | 351 | 610.81 | 2.52 | 1768 | 176.09 | 4.55 | 75 | 479.09 | 2.55 | 0.29 | A | 8.00 | 5.77 | 1.44 |
| 2012 | 41_02 | 8 | 41_30 | 6 | IE | P | 460 | 608.89 | 2.38 | 1771 | 174.56 | 4.69 | 100 | 473.89 | 3.04 | 0.29 | A | 8.00 | 5.72 | 1.43 |
| 2012 | 41_02 | 8 | 41_30 | 6 | IE | P | 413 | 591.76 | 2.47 | 1582 | 171.70 | 4.52 | 76 | 462.17 | 4.10 | 0.29 | A | 8.00 | 5.53 | 1.38 |
| 2012 | 41_02 | 8 | 41_30 | 6 | IE | P | 423 | 605.45 | 2.38 | 1631 | 173.21 | 4.74 | 80 | 479.12 | 4.22 | 0.29 | A | 8.00 | 6.13 | 1.53 |
| 2012 | 41_04 | 5 | 41_10 | 6 | IE | P | 228 | 585.07 | 2.67 | 1892 | 103.96 | 5.56 | 100 | 317.95 | 3.24 | 0.18 | A | 5.00 | 5.29 | 2.12 |
| 2012 | 41_04 | 5 | 41_11 | 5 | IA | P | 572 | 593.31 | 3.19 | 1624 | 103.95 | 5.35 | 74 | 329.10 | 3.13 | 0.18 | A | 5.00 | 5.83 | 2.33 |
| 2012 | 41_05 | 6 | 41_02 | 8 | IE | P | 518 | 592.48 | 3.46 | 1871 | 129.34 | 5.46 | 69 | 408.90 | 2.89 | 0.22 | A | 6.00 | 6.97 | 2.32 |
| 2012 | 41_05 | 6 | 41_02 | 8 | IE | P | 440 | 594.70 | 2.72 | 1953 | 127.83 | 4.92 | 120 | 394.81 | 5.06 | 0.21 | A | 6.00 | 6.53 | 2.18 |
| 2012 | 41_05 | 6 | 41_02 | 8 | IE | P | 443 | 596.41 | 2.44 | 1858 | 128.33 | 4.73 | 88 | 424.24 | 3.37 | 0.22 | A | 6.00 | 7.84 | 2.61 |
| 2012 | 41_05 | 6 | 41_02 | 8 | IE | P | 556 | 606.36 | 3.34 | 1873 | 129.13 | 5.04 | 79 | 383.76 | 5.49 | 0.21 | A | 6.00 | 5.83 | 1.94 |
| 2012 | 41_05 | 6 | 41_02 | 8 | IE | P | 463 | 604.15 | 3.86 | 1989 | 130.18 | 5.52 | 81 | 408.31 | 3.80 | 0.22 | A | 6.00 | 6.82 | 2.27 |
| 2012 | 41_05 | 6 | 41_05 | 6 | S | P | 272 | 609.89 | 2.03 | 1736 | 133.24 | 5.86 | 115 | 411.42 | 4.05 | 0.22 | A | 6.00 | 6.53 | 2.18 |
| 2012 | 41_05 | 6 | 41_05 | 6 | S | P | 305 | 602.31 | 1.99 | 1697 | 131.33 | 4.64 | 116 | 387.21 | 3.58 | 0.22 | A | 6.00 | 5.69 | 1.90 |
| 2012 | 41_05 | 6 | 41_05 | 6 | S | P | 235 | 611.44 | 2.08 | 1888 | 131.74 | 4.76 | 91 | 394.89 | 3.09 | 0.22 | A | 6.00 | 5.98 | 1.99 |
| 2012 | 41_05 | 6 | 41_05 | 6 | S | P | 307 | 610.97 | 1.98 | 1900 | 131.54 | 4.43 | 98 | 394.91 | 3.28 | 0.22 | A | 6.00 | 6.01 | 2.00 |
| 2012 | 41_05 | 6 | 41_05 | 6 | S | P | 182 | 605.79 | 1.75 | 1628 | 130.37 | 4.20 | 78 | 389.08 | 3.15 | 0.22 | A | 6.00 | 5.91 | 1.97 |
| 2012 | 41_05 | 6 | 41_05 | 6 | S | P | 353 | 605.88 | 2.20 | 1845 | 130.04 | 4.56 | 109 | 383.74 | 3.22 | 0.21 | A | 6.00 | 5.71 | 1.90 |
| 2012 | 41_05 | 6 | 41_11 | 5 | IE | P | 262 | 611.54 | 2.46 | 1850 | 130.90 | 4.87 | 114 | 423.39 | 3.66 | 0.21 | A | 6.00 | 7.41 | 2.47 |
| 2012 | 41_10 | 6 | 41_05 | 6 | IA | P | 335 | 613.69 | 2.40 | 2014 | 129.76 | 4.25 | 100 | 382.89 | 2.48 | 0.21 | A | 6.00 | 5.70 | 1.90 |
| 2012 | 41_10 | 6 | 41_10 | 6 | S | P | 486 | 604.81 | 3.31 | 1532 | 130.81 | 5.16 | 116 | 376.33 | 3.67 | 0.22 | A | 6.00 | 5.26 | 1.75 |
| 2012 | 41_10 | 6 | 41_10 | 6 | S | P | 462 | 607.69 | 2.85 | 1625 | 129.59 | 5.55 | 79 | 389.64 | 2.77 | 0.21 | A | 6.00 | 6.04 | 2.01 |
| 2012 | 41_10 | 6 | 41_10 | 6 | S | P | 537 | 610.52 | 2.75 | 1382 | 131.17 | 5.08 | 107 | 395.34 | 3.47 | 0.21 | A | 6.00 | 6.08 | 2.03 |
| 2012 | 41_10 | 6 | 41_10 | 6 | S | P | 490 | 605.98 | 2.91 | 1564 | 130.67 | 5.85 | 114 | 325.50 | 4.54 | 0.22 | A | 6.00 | 2.95 | 0.98 |
| 2012 | 41_10 | 6 | 41_15 | 8 | IE | P | 483 | 598.69 | 2.76 | 1705 | 129.28 | 4.97 | 87 | 386.33 | 4.13 | 0.22 | A | 6.00 | 5.93 | 1.98 |
| 2012 | 41_10 | 6 | 41_21 | 5 | IE | P | 421 | 605.71 | 2.75 | 1626 | 130.53 | 4.72 | 98 | 381.21 | 3.32 | 0.22 | A | 6.00 | 5.52 | 1.84 |
| 2012 | 41_10 | 6 | 41_21 | 5 | IE | P | 470 | 601.47 | 2.55 | 1301 | 128.18 | 4.51 | 90 | 371.15 | 3.59 | 0.21 | A | 6.00 | 5.37 | 1.79 |
| 2012 | 41_11 | 5 | 41_10 | 6 | IE | P | 235 | 611.11 | 2.85 | 1986 | 108.15 | 5.78 | 90 | 338.04 | 3.52 | 0.18 | A | 5.00 | 5.63 | 2.25 |
| 2012 | 41_13 | 5 | 41_11 | 5 | IA | P | 293 | 607.13 | 2.53 | 2012 | 109.29 | 5.28 | 72 | 274.19 | 2.95 | 0.18 | A | 5.00 | 2.54 | 1.02 |
| 2012 | 41_13 | 5 | 41_11 | 5 | IA | P | 326 | 607.80 | 2.56 | 2247 | 110.18 | 4.99 | 109 | 316.01 | 3.46 | 0.18 | A | 5.00 | 4.34 | 1.74 |
| 2012 | 41_13 | 5 | 41_13 | 5 | S | P | 313 | 604.82 | 2.59 | 1915 | 107.24 | 5.27 | 109 | 325.01 | 3.28 | 0.18 | A | 5.00 | 5.15 | 2.06 |
| 2012 | 41_13 | 5 | 41_13 | 5 | S | P | 332 | 608.37 | 2.39 | 2076 | 106.67 | 5.11 | 101 | 330.77 | 3.30 | 0.18 | A | 5.00 | 5.50 | 2.20 |
| 2012 | 41_13 | 5 | 41_29 | 5 | IA | P | 322 | 609.18 | 2.09 | 1983 | 108.72 | 5.10 | 82 | 338.56 | 3.77 | 0.18 | A | 5.00 | 5.57 | 2.23 |
| 2012 | 41_15 | 8 | 41_02 | 8 | IA | L | 244 | 705.24 | 3.01 | 1641 | 124.76 | 4.82 | 61 | 370.23 | 3.29 | 0.18 | A | 8.00 | 7.74 | 1.94 |
| 2012 | 41_15 | 8 | 41_02 | 8 | IA | L | 143 | 690.86 | 2.70 | 2507 | 121.10 | 4.63 | 43 | 336.80 | 3.21 | 0.18 | A | 8.00 | 6.25 | 1.56 |
| 2012 | 41_15 | 8 | 41_02 | 8 | IA | L | 155 | 704.17 | 2.85 | 1830 | 124.52 | 4.65 | 107 | 351.70 | 3.63 | 0.18 | A | 8.00 | 6.60 | 1.65 |
| 2012 | 41_15 | 8 | 41_04 | 5 | IE | L | 237 | 705.46 | 2.97 | 1668 | 126.75 | 5.51 | 84 | 319.89 | 5.30 | 0.18 | A | 8.00 | 4.19 | 1.05 |
| 2012 | 41_15 | 8 | 41_04 | 5 | IE | L | 236 | 708.06 | 2.61 | 1989 | 127.84 | 4.72 | 87 | 376.59 | 3.54 | 0.18 | A | 8.00 | 7.57 | 1.89 |
| 2012 | 41_15 | 8 | 41_04 | 5 | IE | L | 204 | 705.17 | 2.41 | 2091 | 126.02 | 5.29 | 81 | 382.84 | 3.94 | 0.18 | A | 8.00 | 8.30 | 2.08 |
| 2012 | 41_15 | 8 | 41_04 | 5 | IE | L | 202 | 700.75 | 2.96 | 1998 | 126.72 | 4.87 | 87 | 402.55 | 4.20 | 0.18 | A | 8.00 | 9.41 | 2.35 |
| 2012 | 41_15 | 8 | 41_04 | 5 | IE | L | 244 | 705.84 | 2.80 | 1748 | 126.53 | 4.92 | 66 | 368.91 | 3.26 | 0.18 | A | 8.00 | 7.32 | 1.83 |
| 2012 | 41_15 | 8 | 41_04 | 5 | IE | L | 186 | 713.68 | 2.84 | 2128 | 128.58 | 5.15 | 76 | 373.78 | 4.57 | 0.18 | A | 8.00 | 7.26 | 1.81 |
| 2012 | 41_15 | 8 | 41_04 | 5 | IE | L | 147 | 710.00 | 2.89 | 1966 | 127.79 | 4.77 | 34 | 383.89 | 2.48 | 0.18 | A | 8.00 | 8.03 | 2.01 |
| 2012 | 41_15 | 8 | 41_05 | 6 | IE | L | 193 | 715.30 | 2.91 | 1866 | 125.66 | 5.09 | 73 | 354.80 | 4.73 | 0.18 | A | 8.00 | 6.59 | 1.65 |
| 2012 | 41_15 | 8 | 41_21 | 5 | IE | L | 315 | 723.42 | 2.90 | 2244 | 126.14 | 4.90 | 84 | 350.05 | 4.12 | 0.17 | A | 8.00 | 6.20 | 1.55 |
| 2012 | 41_15 | 8 | 41_29 | 5 | IE | L | 215 | 700.00 | 3.09 | 1881 | 121.90 | 5.11 | 58 | 349.25 | 3.53 | 0.17 | A | 8.00 | 6.92 | 1.73 |
| 2012 | 41_15 | 8 | 41_29 | 5 | IE | L | 228 | 691.93 | 2.71 | 1871 | 122.68 | 5.05 | 57 | 381.12 | 4.28 | 0.18 | A | 8.00 | 8.85 | 2.21 |
| 2012 | 41_15 | 8 | 41_29 | 5 | IE | L | 161 | 699.58 | 2.77 | 2008 | 122.36 | 5.31 | 55 | 359.65 | 3.77 | 0.17 | A | 8.00 | 7.51 | 1.88 |
| 2012 | 41_15 | 8 | 41_29 | 5 | IE | L | 150 | 699.95 | 2.80 | 1065 | 193.49 | 4.40 | 67 | 313.40 | 4.00 | 0.28 | AF | 11.87 |  |  |
| 2012 | 41_15 | 8 | 41_29 | 5 | IE | L | 197 | 686.85 | 2.95 | 1759 | 121.43 | 4.68 | 61 | 296.73 | 3.13 | 0.18 | A | 8.00 | 3.55 | 0.89 |
| 2012 | 41_15 | 8 | 41_29 | 5 | IE | L | 246 | 697.89 | 3.33 | 1879 | 124.66 | 4.93 | 44 | 316.86 | 2.76 | 0.18 | A | 8.00 | 4.33 | 1.08 |
| 2012 | 41_15 | 8 | 41_29 | 5 | IE | L | 198 | 709.51 | 3.10 | 1564 | 126.35 | 4.89 | 62 | 315.33 | 4.44 | 0.18 | A | 8.00 | 3.97 | 0.99 |
| 2012 | 41_15 | 8 | 41_29 | 5 | IE | L | 128 | 702.63 | 3.00 | 1839 | 123.91 | 5.16 | 48 | 359.41 | 3.64 | 0.18 | A | 8.00 | 7.20 | 1.80 |
| 2012 | 41_15 | 8 | 41_30 | 6 | IE | L | 244 | 704.80 | 2.84 | 2084 | 123.32 | 5.29 | 93 | 347.02 | 3.96 | 0.17 | A | 8.00 | 6.51 | 1.63 |
| 2012 | 41_21 | 5 | 41_02 | 8 | IE | P | 191 | 606.31 | 2.95 | 1929 | 104.10 | 6.05 | 78 | 284.13 | 4.88 | 0.17 | A | 5.00 | 3.65 | 1.46 |
| 2012 | 41_21 | 5 | 41_10 | 6 | IE | P | 228 | 604.37 | 2.48 | 2018 | 105.63 | 5.40 | 72 | 330.63 | 3.04 | 0.17 | A | 5.00 | 5.65 | 2.26 |
| 2012 | 41_21 | 5 | 41_30 | 6 | IE | P | 209 | 602.36 | 2.45 | 2769 | 105.97 | 5.57 | 41 | 328.32 | 3.59 | 0.18 | A | 5.00 | 5.49 | 2.20 |
| 2012 | 41_30 | 6 | 41_02 | 8 | IE | P | 403 | 589.77 | 2.14 | 1952 | 128.14 | 4.50 | 86 | 349.27 | 4.49 | 0.22 | A | 6.00 | 4.35 | 1.45 |
| 2012 | 41_30 | 6 | 41_02 | 8 | IE | P | 233 | 590.14 | 2.54 | 2007 | 123.35 | 4.84 | 86 | 339.95 | 3.10 | 0.21 | A | 6.00 | 4.54 | 1.51 |
| 2012 | 41_30 | 6 | 41_02 | 8 | IE | P | 392 | 595.27 | 1.96 | 1822 | 130.34 | 4.80 | 89 | 361.80 | 4.36 | 0.22 | A | 6.00 | 4.65 | 1.55 |
| 2012 | 41_30 | 6 | 41_02 | 8 | IE | P | 271 | 591.24 | 1.99 | 1620 | 126.97 | 4.52 | 73 | 394.48 | 3.05 | 0.21 | A | 6.00 | 6.64 | 2.21 |
| 2012 | 41_30 | 6 | 41_15 | 8 | IE | P | 376 | 594.99 | 1.76 | 1736 | 127.16 | 4.68 | 87 | 321.64 | 4.14 | 0.21 | A | 6.00 | 3.18 | 1.06 |
| 2012 | 41_30 | 6 | 41_15 | 8 | IE | P | 354 | 594.37 | 1.68 | 1952 | 127.52 | 4.99 | 79 | 383.23 | 3.44 | 0.21 | A | 6.00 | 6.03 | 2.01 |
| 2012 | 41_30 | 6 | 41_15 | 8 | IE | P | 499 | 598.92 | 1.75 | 2520 | 128.43 | 4.83 | 80 | 509.55 | 2.75 | 0.21 | A | 6.00 | 11.81 | 3.94 |
| 2012 | 41_30 | 6 | 41_15 | 8 | IE | P | 384 | 596.04 | 1.75 | 1811 | 129.92 | 4.95 | 79 | 326.97 | 3.92 | 0.22 | A | 6.00 | 3.10 | 1.03 |
| 2012 | 41_30 | 6 | 41_15 | 8 | IE | P | 410 | 602.01 | 1.80 | 1796 | 127.89 | 4.30 | 67 | 321.19 | 3.63 | 0.21 | A | 6.00 | 3.07 | 1.02 |
| 2012 | 41_30 | 6 | 41_15 | 8 | IE | P | 303 | 592.92 | 2.28 | 1836 | 128.62 | 4.59 | 97 | 387.58 | 3.16 | 0.22 | A | 6.00 | 6.08 | 2.03 |
| 2012 | 41_30 | 6 | 41_15 | 8 | IE | P | 340 | 594.34 | 1.71 | 1741 | 127.10 | 4.45 | 101 | 324.94 | 2.97 | 0.21 | A | 6.00 | 3.34 | 1.11 |
| 2012 | 41_30 | 6 | 41_15 | 8 | IE | P | 356 | 599.47 | 2.03 | 1997 | 128.08 | 4.25 | 25 | 532.07 | 1.70 | 0.21 | A | 6.00 | 12.93 | 4.31 |
| 2012 | 41_30 | 6 | 41_15 | 8 | IE | P | 317 | 587.94 | 1.79 | 1941 | 126.66 | 4.84 | 90 | 393.58 | 3.48 | 0.22 | A | 6.00 | 6.64 | 2.21 |
| 2012 | 41_30 | 6 | 41_21 | 5 | IE | P | 185 | 610.47 | 2.29 | 878 | 129.34 | 4.32 | 51 | 358.34 | 4.02 | 0.21 | A | 6.00 | 4.62 | 1.54 |
| 2012 | 41_30 | 6 | 41_30 | 6 | S | P | 568 | 611.04 | 2.99 | 1669 | 127.70 | 5.04 | 92 | 380.07 | 3.59 | 0.21 | A | 6.00 | 5.86 | 1.95 |
| 2012 | 49_06 | 5 | 49_06 | 5 | S | P | 198 | 610.99 | 2.39 | 1967 | 109.74 | 5.31 | 74 | 335.00 | 3.27 | 0.18 | A | 5.00 | 5.26 | 2.11 |
| 2012 | 49_06 | 5 | 49_06 | 5 | S | P | 247 | 603.63 | 2.39 | 2079 | 109.49 | 5.22 | 81 | 341.65 | 3.26 | 0.18 | A | 5.00 | 5.60 | 2.24 |
| 2012 | 49_06 | 5 | 49_06 | 5 | S | P | 273 | 601.21 | 2.36 | 1946 | 107.38 | 5.24 | 101 | 319.39 | 2.59 | 0.18 | A | 5.00 | 4.87 | 1.95 |
| 2012 | 49_06 | 5 | 49_06 | 5 | S | P | 238 | 597.78 | 2.48 | 1727 | 108.37 | 5.20 | 86 | 337.03 | 3.64 | 0.18 | A | 5.00 | 5.55 | 2.22 |
| 2012 | 49_06 | 5 | 49_13 | 6 | IE | P | 249 | 595.23 | 2.54 | 1892 | 105.47 | 5.28 | 81 | 316.66 | 2.13 | 0.18 | A | 5.00 | 5.01 | 2.00 |
| 2012 | 49_06 | 5 | 49_23 | 6 | IE | P | 169 | 611.08 | 2.65 | 1992 | 110.17 | 5.50 | 96 | 349.20 | 3.62 | 0.18 | A | 5.00 | 5.85 | 2.34 |
| 2012 | 49_08 | 5 | 49_13 | 6 | IE | P | 138 | 605.59 | 2.50 | 2121 | 109.36 | 5.73 | 218 | 338.39 | 3.52 | 0.18 | A | 5.00 | 5.47 | 2.19 |
| 2012 | 49_08 | 5 | 49_24 | 6 | IE | P | 217 | 607.70 | 2.42 | 2243 | 108.24 | 5.16 | 98 | 324.79 | 3.38 | 0.18 | A | 5.00 | 5.00 | 2.00 |
| 2012 | 49_13 | 6 | 49_08 | 5 | IE | P | 579 | 598.94 | 3.24 | 1622 | 127.42 | 5.03 | 105 | 357.25 | 4.08 | 0.21 | A | 6.00 | 4.82 | 1.61 |
| 2012 | 49_13 | 6 | 49_13 | 6 | S | P | 422 | 602.97 | 2.74 | 2103 | 125.01 | 5.36 | 92 | 365.08 | 3.70 | 0.21 | A | 6.00 | 5.52 | 1.84 |
| 2012 | 49_13 | 6 | 49_13 | 6 | S | P | 367 | 604.15 | 2.49 | 1742 | 125.78 | 4.86 | 77 | 373.47 | 3.23 | 0.21 | A | 6.00 | 5.82 | 1.94 |
| 2012 | 49_13 | 6 | 49_24 | 6 | IA | P | 380 | 604.84 | 2.66 | 1765 | 129.81 | 4.70 | 136 | 385.08 | 3.35 | 0.21 | A | 6.00 | 5.80 | 1.93 |
| 2012 | 49_13 | 6 | 49_28 | 6 | IA | P | 316 | 602.15 | 2.53 | 2008 | 128.00 | 5.00 | 90 | 379.31 | 3.38 | 0.21 | A | 6.00 | 5.78 | 1.93 |
| 2012 | 49_13 | 6 | 49_28 | 6 | IA | P | 345 | 600.31 | 2.54 | 2089 | 126.43 | 5.24 | 76 | 376.55 | 2.87 | 0.21 | A | 6.00 | 5.87 | 1.96 |
| 2012 | 49_14 | 5 | 49_27 | 6 | IE | P | 116 | 599.19 | 2.64 | 2440 | 107.13 | 5.21 | 90 | 433.95 | 3.21 | 0.18 | A | 5.00 | 10.25 | 4.10 |
| 2012 | 49_17 | 5 | 49_23 | 6 | IE | P | 181 | 596.89 | 2.80 | 2184 | 104.49 | 5.54 | 109 | 333.25 | 3.84 | 0.18 | A | 5.00 | 5.95 | 2.38 |
| 2012 | 49_19 | 5 | 49_19 | 5 | S | P | 233 | 603.63 | 2.36 | 1874 | 109.73 | 5.06 | 117 | 323.04 | 3.24 | 0.18 | A | 5.00 | 4.72 | 1.89 |
| 2012 | 49_19 | 5 | 49_27 | 6 | IE | P | 128 | 592.58 | 2.84 | 1781 | 103.29 | 5.29 | 72 | 276.11 | 3.09 | 0.17 | A | 5.00 | 3.37 | 1.35 |
| 2012 | 49_23 | 6 | 49_06 | 5 | IE | P | 588 | 600.02 | 2.74 | 1297 | 129.21 | 5.11 | 86 | 382.01 | 3.52 | 0.22 | A | 6.00 | 5.74 | 1.91 |
| 2012 | 49_23 | 6 | 49_13 | 6 | IA | P | 483 | 601.48 | 3.01 | 1789 | 128.02 | 4.77 | 90 | 373.77 | 4.12 | 0.21 | A | 6.00 | 5.52 | 1.84 |
| 2012 | 49_23 | 6 | 49_17 | 5 | IE | P | 494 | 605.74 | 2.68 | 1462 | 129.05 | 5.13 | 87 | 368.71 | 3.60 | 0.21 | A | 6.00 | 5.14 | 1.71 |
| 2012 | 49_23 | 6 | 49_23 | 6 | S | P | 329 | 605.23 | 3.34 | 963 | 132.19 | 4.82 | 107 | 385.60 | 4.04 | 0.22 | A | 6.00 | 5.50 | 1.83 |
| 2012 | 49_23 | 6 | 49_23 | 6 | S | P | 567 | 614.96 | 3.37 | 1128 | 201.99 | 4.61 | 47 | 345.37 | 2.67 | 0.33 | AF | 9.20 |  |  |
| 2012 | 49_23 | 6 | 49_23 | 6 | S | P | 468 | 603.92 | 3.25 | 926 | 135.12 | 5.12 | 76 | 335.20 | 3.23 | 0.22 | A | 6.00 | 2.88 | 0.96 |
| 2012 | 49_23 | 6 | 49_24 | 6 | IA | P | 408 | 597.55 | 3.15 | 1610 | 133.27 | 5.97 | 71 | 339.07 | 3.95 | 0.22 | A | 6.00 | 3.27 | 1.09 |
| 2012 | 49_24 | 6 | 49_14 | 5 | IE | P | 400 | 602.38 | 2.83 | 1161 | 128.27 | 4.87 | 70 | 377.51 | 2.85 | 0.21 | A | 6.00 | 5.66 | 1.89 |
| 2012 | 49_24 | 6 | 49_28 | 6 | IA | P | 685 | 599.48 | 3.54 | 1507 | 127.25 | 5.27 | 74 | 374.30 | 3.67 | 0.21 | A | 6.00 | 5.65 | 1.88 |
| 2012 | 49_24 | 6 | 49_28 | 6 | IA | P | 587 | 593.25 | 4.10 | 1642 | 128.16 | 5.55 | 57 | 391.02 | 4.51 | 0.22 | A | 6.00 | 6.31 | 2.10 |
| 2012 | 49_27 | 6 | 49_13 | 6 | IA | P | 536 | 599.56 | 3.29 | 1814 | 127.14 | 5.12 | 66 | 383.21 | 2.65 | 0.21 | A | 6.00 | 6.08 | 2.03 |
| 2012 | 49_27 | 6 | 49_27 | 6 | S | P | 506 | 605.03 | 3.07 | 1483 | 129.53 | 5.49 | 91 | 387.30 | 3.49 | 0.21 | A | 6.00 | 5.94 | 1.98 |
| 2012 | 49_28 | 6 | 49_06 | 5 | IE | P | 564 | 594.77 | 3.06 | 1204 | 125.49 | 4.90 | 80 | 358.89 | 3.54 | 0.21 | A | 6.00 | 5.16 | 1.72 |
| 2012 | 49_28 | 6 | 49_24 | 6 | IA | P | 566 | 596.33 | 2.79 | 1893 | 126.63 | 4.80 | 99 | 387.00 | 3.22 | 0.21 | A | 6.00 | 6.34 | 2.11 |

Literature Cited

Greilhuber, J. & Ebert I. 1994. Genome size variation in *Pisum sativum*. Genome **37**: 646-655.
