# Supplementary material for: Asymmetric reproductive interference: The consequences of cross‐pollination on reproductive success in sexual–apomictic populations of Potentilla puberula (Rosaceae)
Source: Ecol Evol. 2017 Nov 28;8(1):365–81. doi: 10.1002/ece3.3684 (PMC5756837; doi:10.1002/ece3.3684)
Supplement: Supplementary file 3 [file ECE3-8-365-s003.docx]

**Online Resource 3** Fixed-effect coefficients of binomial Generalized Linear Mixed Models comparing seed set of A) tetraploid sexual pollen recipients and B) penta- to octoploid apomicts obtained in selfings and homoploid crosses in *Potentilla puberula*. Calculations were done separately for each ploidy level of the pollen recipient. Groups refer to the number of population and number of pollen recipients (individuals) nested within populations. Values in parentheses are the number of pollen donors. *P*-values given in bold indicate significant differences in selfed individuals, and open controls (pollen donor unknown) compared to homoploid crosses (representing the model intercept).

| **A) sexual pollen recipients** | coef ± SE | *z*-value | *p*-value | |
| --- | --- | --- | --- | --- |
|  | *N* = 112, groups = 3/12 | | |  |
| intercept (47) | –0.30 ± 0.24 | –1.23 | 0.218 | |
| selfing (12) | –3.23 ± 0.30 | –10.74 | **< 0.001** | |
| open control (12) | 0.17 ± 0.12 | 1.36 | 0.173 | |

| **B) apomictic pollen recipients** | coef ± SE | *z*-value | ***p*-value** | |  |
| --- | --- | --- | --- | --- | --- |
| pentaploids | *N* = 338, groups = 11/59 | | |  |  |
| intercept (153) | –1.96 ± 0.14 | –13.88 | **< 0.001** | | |
| selfing (129) | –0.38 ± 0.07 | –5.11 | **< 0.001** | | |
| open control (56) | 0.83 ± 0.07 | 12.33 | **< 0.001** | | |
| hexaploids | *N* = 108, groups = 5/20 | | |  |  |
| intercept (58) | –1.31 ± 0.19 | –6.99 | **< 0.001** | |  |
| selfing (30) | –0.41 ± 0.10 | –4.24 | **< 0.001** | |  |
| open control (20) | 0.40 ± 0.09 | 4.33 | **< 0.001** | |  |
| heptaploids | *N* = 91, groups = 5/19 | | |  |  |
| intercept (48) | –1.86 ± 0.30 | –6.21 | **< 0.001** | |  |
| selfing (24) | 0.06 ± 0.12 | 0.53 | 0.597 | |  |
| open control (19) | 0.24 ± 0.11 | 2.11 | 0.035 | |  |
| octoploids | *N* = 76, groups = 4/15 | | |  |  |
| intercept (12) | –1.92 ± 0.20 | –9.73 | **< 0.001** | |  |
| selfing (50) | –0.17 ± 0.18 | –0.98 | 0.328 | |  |
| open control (14) | 0.18 ± 0.20 | 0.93 | 0.355 | |  |
