# Supplementary material for: Asymmetric reproductive interference: The consequences of cross‐pollination on reproductive success in sexual–apomictic populations of Potentilla puberula (Rosaceae)
Source: Ecol Evol. 2017 Nov 28;8(1):365–81. doi: 10.1002/ece3.3684 (PMC5756837; doi:10.1002/ece3.3684)
Supplement: Supplementary file 4 [file ECE3-8-365-s004.docx]

**Online Resource 4** Generalised linear models comparing the proportion of viable pollen grains between tetraploids (intercept) and higher polyploids of *Potentilla puberula*. Pollen grains were pooled within populations for each cytotype to balance for the high individual variability of pollen viability. Tetra-, penta-, hexa-, hepta- and octoploids are represented by four, 11, five, five, and four populations, respectively. We assumed binomial error distribution and used the number of individuals within populations as weights in the regression analysis.

|  | coefficient ± SE | z value | p value |
| --- | --- | --- | --- |
| intercept | 0.33 ± 0.01 | 109.8 | < 0.001 |
| pentaploid | -0.21 ± 0.01 | -58.7 | < 0.001 |
| hexaploid | 0.42 ± 0.01 | 93.5 | < 0.001 |
| heptaploid | 0.76 ± 0.01 | 152.9 | < 0.001 |
| octoploid | -0.82 ± 0.01 | -160.3 | < 0.001 |
