# Supplementary material for: Asymmetric reproductive interference: The consequences of cross‐pollination on reproductive success in sexual–apomictic populations of Potentilla puberula (Rosaceae)
Source: Ecol Evol. 2017 Nov 28;8(1):365–81. doi: 10.1002/ece3.3684 (PMC5756837; doi:10.1002/ece3.3684)
Supplement: Supplementary file 5 [file ECE3-8-365-s005.docx]

**Online Resource 5** Fixed effect coefficients of binomial Generalized Linear Mixed Models relating seed set and germination rate of tetra- to octoploid individuals of *Potentilla puberula* (Rosaceae) to A) Δ *p* and B) Δ p_min_ in the endosperm. The left column represents the ploidy level of the pollen recipient and – in A) – its preferred reproductive mode of seed formation. Significant associations are given in bold. *N* represents the number of pollinated flowers.

| **A)** |  | seed set | | | | | germination | | | | |
| --- | --- | --- | --- | --- | --- | --- | --- | --- | --- | --- | --- |
|  | *N* | coef ± SE | z-value | | p-value | | *N* | coef ± SE | z-value | p-value | |
| sexuals |  |  |  | |  | |  |  |  |  | |
| tetraploid | 48 | –1.63 ± 0.37 | –4.36 | | **< 0.001** | | 46 | –0.50 ± 0.61 | –0.83 | 0.408 | |
| apomicts |  |  |  | |  | |  |  |  |  | |
| pentaploid | 118 | –0.22 ± 0.07 | –3.12 | | **0.002** | | 117 | –0.21 ± 0.20 | –1.07 | 0.287 | |
| hexaploids | 83 | 0.01 ± 0.11 | 0.11 | | 0.913 | | 80 | 0.46 ± 0.26 | 1.77 | 0.077 | |
| heptaploid | 58 | 0.19 ± 0.21 | 0.91 | | 0.364 | | 41 | 0.98 ± 0.76 | 1.29 | 0.197 | |
| oktoploid | 61 | 0.06 ± 0.09 | 0.65 | | 0.517 | | 46 | 0.11 ± 0.18 | 0.60 | 0.552 | |
| **B)** |  | seed set | | | | | germination | | | | |
|  | *N* | coef ± SE | | z-value | | p-value | *N* | coef ± SE | z-value | | p-value |
| pentaploid | 118 | –0.32 ±0.11 | | –3.011 | | **0.003** | 117 | 0.01 ±0.13 | 0.103 | | 0.918 |
| hexaploid | 83 | –0.11 ±0.16 | | –0.693 | | 0.488 | 80 | 0.46 ±0.26 | 1.766 | | 0.077 |
| heptaploid | 58 | –0.79 ±0.62 | | –1.278 | | 0.201 | 41 | 0.98 ±0.76 | 1.292 | | 0.196 |
| octoploid | 61 | 0.04 ±0.15 | | 0.280 | | 0.780 | 46 | 0.11 ±0.19 | 0.595 | | 0.552 |
